# Fluorinated Aminopiperidones as Non‐Glutarimide Thalidomide Analogs: Stereodivergent Synthesis and Validation

**DOI:** 10.1002/cmdc.70360

**Published:** 2026-07-07

**Authors:** Boštjan Adamlje, Tihomir Tomašič, Christian Steinebach, Sebastian Ebeling, Alexander Herrmann, Marcus D. Hartmann, Jessica L. Horner, Kinjal Bhadresha, Cindy H. Chau, William D. Figg, Izidor Sosič, Andrej Emanuel Cotman

**Affiliations:** ^1^ Faculty of Pharmacy University of Ljubljana Ljubljana Slovenia; ^2^ Institute of Pharmacy Pharmaceutical/Medicinal Chemistry University of Greifswald Greifswald Germany; ^3^ Max Planck Institute for Biology Tübingen Tübingen Germany; ^4^ Interfaculty Institute of Biochemistry University of Tübingen Tübingen Germany; ^5^ Molecular Pharmacology Section Genitourinary Malignancies Branch Center for Cancer Research National Cancer Institute NIH Bethesda Maryland USA

**Keywords:** bioisosterism, diastereoselectivity, drug design, fluorine, PROTAC

## Abstract

The glutarimide fragment is a key pharmacophore in cereblon‐binding immunomodulatory drugs and proteolysis‐targeting chimeras, yet exploration of non‐glutarimide scaffolds remains limited. Here, we report the design, synthesis, and evaluation of fluorinated aminopiperidones as three‐dimensional, non‐glutarimide thalidomide analogs. By replacing a single glutarimide carbonyl with a stereogenic C*–CF_3_ unit, we accessed a previously underexplored chemical space encompassing all regio‐ and diastereomeric variants of 3‐ and 5‐phthalimido‐6‐trifluoromethyl‐2‐piperidones. Stereocontrolled synthetic routes enabled reliable control over relative configurations, and two‐dimensional NMR provided unambiguous structural assignment. Experimental profiling of CF_3_‐piperidones relative to glutarimides revealed improved aqueous solubility despite increased log*D* and enhanced membrane interaction characteristics, without substantial changes in plasma protein binding, consistent with sp^3^ enrichment and escape‐from‐flatland principles. To assess whether cereblon recognition is retained after carbonyl‐to‐C*–CF_3_ replacement, the compounds were evaluated by microscale thermophoresis. No measurable cereblon binding was detected for the fluorinated aminopiperidones. Docking studies, performed to rationalize this lack of affinity, suggested disruption of key cereblon–ligand interactions and unfavorable binding poses following carbonyl‐to‐C*–CF_3_ substitution. Several CF_3_‐piperidones, particularly tetrafluorophthalimide derivatives, exhibited potent anti‐angiogenic activity in vitro and ex vivo, indicating a cereblon‐independent mechanism.

## Graphical abstract

Fluorinated aminopiperidones, designed as non‐glutarimide thalidomide analogs, were synthesized with full regio‐ and stereocontrol. The C^*^–CF_3_ used as carbonyl surrogate improved solubility and membrane interactions while abolishing cereblon binding. The resulting CF_3_‐piperidones displayed potent anti‐angiogenic activity, highlighting cereblon‐independent bioactivity and potential of trifluoroethylamines in drug design.

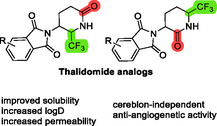

## 1 Introduction

The aminoglutarimide fragment is a part of thalidomide, initially developed as an antiemetic but later withdrawn due to teratogenicity [1]. Over the past two decades, its derivatives have emerged as clinically validated cereblon‐binding molecular glue degraders, exemplified by the approved immunomodulatory drugs thalidomide, lenalidomide, and pomalidomide, which reprogram the CRL4^CRBN^ E3 ligase to induce selective degradation of neosubstrates. This paradigm of small‐molecule–induced protein degradation has established cereblon as a central hub in targeted protein degradation and subsequently enabled the development of proteolysis‐targeting chimeras (PROTACs), in which cereblon ligands serve as key E3‐recruiting elements [2, 3, 4, 5]. Bioisosteres are valuable design elements that medicinal chemists use to tune the structural and pharmacokinetic properties of bioactive compounds, often with the goal of enhancing potency or selectivity, reducing metabolic liabilities, modifying toxicophores, or generating new intellectual property [6, 7, 8, 9, 10, 11, 12, 13, 14, 15]. In recent years, there has been increasing recognition that the structural complexity of drug candidates strongly influences their clinical success. While early drug candidates tend to be flat and noncomplex, approved drugs more often feature saturated frameworks and stereogenic centers (sp^3^‐carbons), indicating that targeting three‐dimensional chemical space is essential for successful drug development [16, 17]. Significant advances have been achieved in the development of three‐dimensional, sp^3^‐rich benzene ring surrogates, illustrating how synthetic methodology development, coupled with feedback from medicinal chemistry, can establish and validate new bioisosteric concepts [18, 19]. Considering the prevalence of planar, carbonyl‐containing functional groups such as amides, carboxylic acids, peptides, and α‐hydroxy acids in medicinal chemistry [20], there is a clear opportunity to explore their three‐dimensional bioisosteric replacements [12, 13, 14, 15].

Replacement of a carbonyl group by a stereogenic C*–CF_3_ motif represents an attractive design strategy. This substituent can mimic certain electronic features of the planar carbonyl group, retains hydrogen bond–donating properties while reducing NH basicity, and introduces a directional, highly polarized substituent that can approximate aspects of carbonyl functionality. Its sp^3^ hybridization can influence the conformation of adjacent substituents, potentially enhancing interactions within a target active site [21]. Furthermore, incorporation of fluorine into bioactive molecules profoundly affects their physicochemical and pharmacokinetic properties, contributing to the high prevalence of fluorinated motifs in blockbuster drugs and newly approved pharmaceuticals [22, 23, 24, 25, 26, 27, 28]. In line with the escape from flatland strategies [16, 29, 30], we are interested in exploring how replacing a carbonyl group with its isopolar three‐dimensional surrogate, C*–CF_3_, affects the properties and bioactivity of small molecules [31].

**FIGURE 1:**
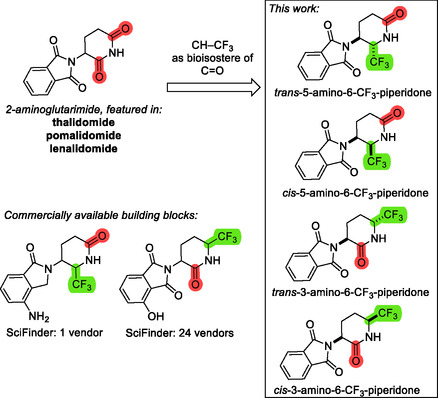
Design of fluorinated aminopiperidones as non‐glutarimide thalidomide analogs, and representative commercially available building blocks.

Replacement of a single carbonyl group in 2‐aminoglutarimide with a trifluoroethyl group affords regioisomeric 3‐ or 5‐amino‐6‐trifluoromethyl‐2‐piperidones, each existing as two diastereomers (Figure 1). 3‐Phthalimido‐6‐CF_3_‐piperidones have previously been described in one peer‐reviewed article and one patent as thalidomide analogs [32, 33], and SciFinder searches reveal that both 3‐ and 5‐amino‐6‐CF_3_‐piperidone‐derived compounds are already present in commercial protein degrader toolkits (Figure 1). We therefore pursued the synthesis of regio‐ and stereochemically defined fluorinated aminopiperidones to systematically examine the impact of this replacement on physicochemical properties and biological activity, including the influence on cereblon binding, relative to the parent glutarimide. Thalidomide analogs exhibit pronounced anti‐angiogenic activity, which can be substantially enhanced through targeted molecular modifications. In particular, tetrafluorinated derivatives have emerged as more potent inhibitors of angiogenesis, illustrating how fine‐tuning of the thalidomide scaffold can translate into strongly improved biological activity [34, 35, 36]. Notably, it was recently determined that fluorophthalimides show cereblon‐independent anti‐angiogenic activity [37]. Accordingly, we evaluated the anti‐angiogenic activity of this class of compounds using endothelial cell tube formation assay and rat aortic ring assay [38].

## 2 Results and Discussion

**SCHEME 1:**
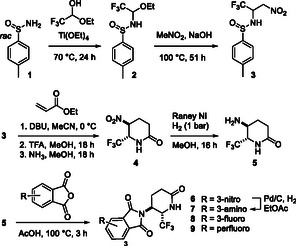
Synthesis of racemic *trans*‐6‐CF_3_‐5‐phthalimidopiperidones **6**–**9**.

For the preparation of *tra* *ns‐5‐amino‐6‐CF* *_3_* *‐* *piperidone* (Scheme 1), we employed the racemic tolylsulfinyl amide **1** as the starting material. Reaction with the ethyl hemiacetal of trifluoroacetaldehyde afforded a mixture of two diastereomers in a 1:1 ratio. Subsequently, an aza‐Henry reaction of the hemiaminal ether **2** with nitromethane was carried out in the presence of NaOH, requiring elevated temperature for efficient conversion. The resulting β‐nitroamine **3** underwent cyclization via Michael addition to ethyl acrylate, followed by acidolytic sulfinyl deprotection and ring closure to afford **4** as a single diastereomer [39]. Consistent with the literature report [39], starting from **3** as a ~65:35 mixture of diastereomers, four diastereomers of the Michael addition product in a 32:27:23:18 ratio were detected in the crude reaction mixture. Upon sulfinyl deprotection, the two remaining chiral centers are both substituted with strong electron‐withdrawing groups, which makes them prone to epimerization. The *trans* selectivity observed during the formation of **4** might therefore involve dynamic kinetic resolution during cyclization, followed by additional thermodynamically driven *cis*–*trans* epimerization [40]. The diastereomerically pure 5‐nitro‐2‐piperidone **4** was then subjected to Raney nickel–catalyzed hydrogenation, providing the corresponding primary amine **5**. Finally, **5** was coupled with the appropriate phthalic anhydride in the presence of acetic acid and sodium acetate to afford compounds **6**–**9**.

**SCHEME 2:**
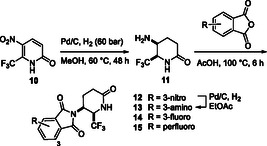
Synthesis of *cis*‐6‐CF_3_‐5‐phthalimidopiperidones **12**–**15**.

The *cis*‐5‐amino‐6‐CF_3_‐piperidones were prepared by total reduction of the corresponding 5‐nitro‐2‐pyridinone **10** (Scheme 2). The reaction was carried out in a steel reactor under elevated hydrogen pressure and temperature in the presence of palladium on carbon as a catalyst. The reaction was *cis*‐stereospecific, delivering the piperidone **11** in an unoptimized 47% isolated yield after purification. **11** was then readily converted to the phthalimides using the above‐described strategy **12**–**15**.

**SCHEME 3:**
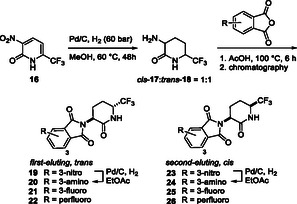
Synthesis of 6‐CF_3_‐3‐phthalimidopiperidones. The diastereomers were separated by normal‐phase chromatography on silica.

The 3‐amino‐6‐CF_3_‐piperidones were obtained starting from the corresponding 3‐nitropyridine **16** (Scheme 3). As in the case of the *cis*‐5‐amino‐6‐CF_3_‐piperidone, the reduction was performed in a steel hydrogenator under elevated hydrogen pressure and temperature to afford the corresponding piperidone. In this instance, a mixture of diastereomers was formed in 1:1 ratio, because the product is configurationally labile. The formation of the *trans* isomer can be explained by its higher thermodynamic stability compared to the cis isomer [32]. For this reason, no separation was carried out at this stage; instead, the mixture was directly reacted with the appropriate phthalic acid derivative in the presence of acetic acid and sodium acetate. The resulting mixtures of both diastereomers were then successfully separated by flash chromatography, where the *trans* isomers eluted first and the *cis* isomers second.

**FIGURE 2:**
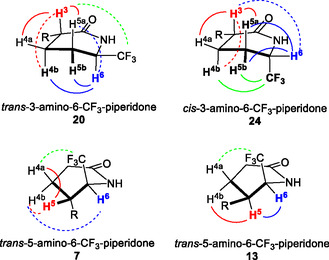
Determination of the relative configurations by ^1^H–^1^H NOESY and ^1^H–^19^F HOESY NMR experiments. Blue and red marks represent the observed nuclear Overhauser effect interactions with protons next to the CF_3_ and R = 3‐aminophthalimido, respectively. Green marks represent the observed heteronuclear Overhauser effect interactions with CF_3_. Solid line represents strong interaction, and dashed line represents weak interaction.

For the 3‐phthalimido‐6‐CF_3_ regioisomers, the pure diastereomers were obtained by chromatographic separation at the final stage, and it was therefore important to establish the relative configurations of the first‐ and second‐eluting diastereomer. Unambiguous assignment was achieved by means of ^1^H–^1^H NOESY and ^1^H–^19^F HOESY NMR experiments, which indicated a *trans* configuration of the first‐eluting isomer and a *cis* configuration of the second‐eluting isomer. The key NMR correlations for the 3‐aminophthalimido derivatives **20** and **24** are shown in Figure 2; for a detailed discussion of the assignment of relative configurations for compounds **19**–**26**, see the Supporting Information. On the other hand, the respective diastereomers of the 5‐phthalimido regioisomers **6**–**15** were obtained via diastereoselective synthesis of the corresponding aminopiperidone precursors **5** and **11** without erosion of stereomeric purity during the phthalimide synthesis step. Their relative configurations were confirmed by two‐dimensional NMR experiments (Figure 2, bottom).

To evaluate the effect of the carbonyl replacement, a set of ADME‐related physicochemical properties was experimentally determined for the parent glutarimides **27**–**30** and the fluorinated aminopiperidones (Table 1). The most relevant comparisons are between analogs bearing the same phthalimide substitution but differing in the aliphatic moiety. In the 3‐aminophthalimide series, our determination of the thermodynamic solubility of pomalidomide (**28**) in phosphate‐buffered saline (PBS, pH 7.4) at 25 °C (13.2 µM) is consistent with the low PBS solubility reported in the literature at 37 °C (53.0 µM) [45]. The corresponding fluorinated aminopiperidones **7**, **13**, **20**, and **24** all exhibit higher solubility, with the *cis*‐5‐amino‐6‐CF_3_‐piperidone **13** being the most soluble member of this series (Table 1). Interestingly, the *cis* diastereomers tended to be more soluble than the corresponding *trans* isomers in the 5‐amino‐6‐CF_3_ series, whereas no clear stereochemical effect on solubility was observed for the 3‐amino‐6‐CF_3_ series.

**TABLE 1:** Evaluation of ADME‐related physicochemical properties and inhibition of angiogenesis.

Structure^a^	R =	Cpd ID	Solubility^a^, μM	MP, °C	Log*D*_7.4_ b	eLog*D*_7.4_ c	CHI_IAM_ d	PPB^e^, %	Tube formation, inh. @10 μM	RAR assay, inh. @100 μM
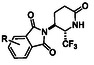	3‐Nitro	**6**	896	228–232	−0.06	n.d.^g^	n.d.	n.d.	9.5	47.2
	3‐Amino	**7**	80	231–235	1.42	n.d.	n.d.	n.d.	35.3	69.5
	3‐Fluoro	**8**	147	241–245	0.98	n.d.	n.d.	n.d.	n.i.	62.8
	Perfluoro	**9**	265	225–226	4.46	2.0	22.87	40	99.1	63.8
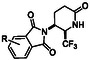	3‐Nitro	**12**	818	249–252	0.25	1.1	19.36	54	12.0	47.9
	3‐Amino	**13**	228	238–241	1.28	0.9	20.65	74	13.4	57.8
	3‐Fluoro	**14**	466	218–222	1.00	1.1	19.30	56	24.3	17.6
	Perfluoro	**15**	948	224–229	0.94	1.9	23.72	53	97.7	68.4
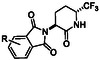	3‐Nitro	**19**	1028	238–241	0.51	1.7	20.86	51	3.2	47.7
	3‐Amino	**20**	34	285–286	1.32	1.4	22.22	67	3.3	33.4
	3‐Fluoro	**21**	305	220–223	1.15	1.6	19.95	45	32.1	30.2
	Perfluoro	**22**	220	241–246	1.13	2.2	22.29	52	98.5	48.1
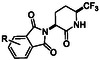	3‐Amino	**24**	42	296–300	1.16	1.3	23.06	70	8.4	57
	3‐Fluoro	**25**	66	275–283	1.06	1.5	21.38	50	33.0	27.8
	Perfluoro	**26**	192	273–280	1.10	2.2	24.75	66	98.8	35.4
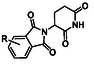	3‐Nitro	**27**	<1	>250 [41]	1.87	n.d.	n.d.	n.d.		
	3‐Amino	**28**	13	293–295	1.01	0.5 [42]	−4.0 [42]	51 [42]	n.i.^f^	n.i.^g^
	3‐Fluoro	**29**	67	236–238 [41]	1.02	n.d.	n.d.	n.d.		
	Perfluoro	**30**	8.2	249–259	2.30	1.4	17.76	44	94.5	65.3

a Thermodynamic solubility in phosphate‐buffered saline (PBS) pH 7.4.

b Octanol/phosphate buffer pH 7.4 distribution coefficient determined by shake‐flask method.

c Distribution coefficient estimated by the HPLC‐based method.

d Chromatographic hydrophobicity index values referring to IAM chromatography, an estimate for drug–membrane interactions and permeability.

e Plasma protein binding; experimentally determined percentage of compound bound to human serum albumin. MP, melting point; n.d., not determined; n.i., no inhibition.

f No inhibition at 220 μM [43].

g No inhibition at 50 μM [44].

**FIGURE 3:**
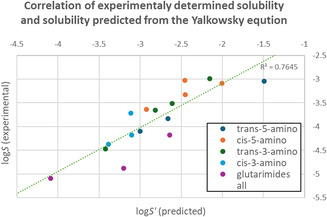
Correlation of experimentally determined solubility, and solubility predicted from the Yalkowsky general solubility equation log*S′* = −0.01(*T*_m_ − 25) − log*D* + 0.5; *S′* is the predicted aqueous solubility; *T*_m_ is the Celsius melting point.

Lipophilicity was assessed by octanol/phosphate buffer pH 7.4 distribution coefficient (log*D*_7.4_) determined by shake‐flask method and also estimated (elog*D*_7.4_) by an high‐performance liquid chromatography (HPLC)‐based method (Table 1). The fluorinated aminopiperidones **7**, **13**, **20**, and **24** have higher lipophilicity than pomalidomide regardless of the method used. Improved solubility coinciding with increased lipophilicity may be counterintuitive, but the observed trend can be explained by fitting the solubility, melting point, and log*D* data to the Yalkowsky general solubility equation, log*S′* = −0.01(*T*_m_ − 25) − log*D* + 0.5, wherein *S′* is the estimated aqueous solubility and *T*_m_ is the compound's Celsius melting point (Figure 3) [46, 47]. According to this relationship, the lower melting points of the CF_3_‐piperidones, reflecting reduced crystal lattice stability, can outweigh the unfavorable effect of increased lipophilicity and result in higher aqueous solubility. Improved solubility due to decreased melting point of the sp^3^‐enriched compounds is central to the *escape from flatland* paradigm [16].

The membrane permeability and interactions with the membranes were estimated by the chromatographic hydrophobicity index obtained with the immobilized artificial membrane (IAM) column. All fluorinated aminopiperidones appear to possess better membrane permeability compared to the analogous glutarimides. The plasma protein binding (PPB) was estimated by the chromatographic method using a column containing human serum albumin immobilized on silica gel. The PPB values for glutarimides and the CF_3_‐piperidones were in the same range.

**FIGURE 4:**
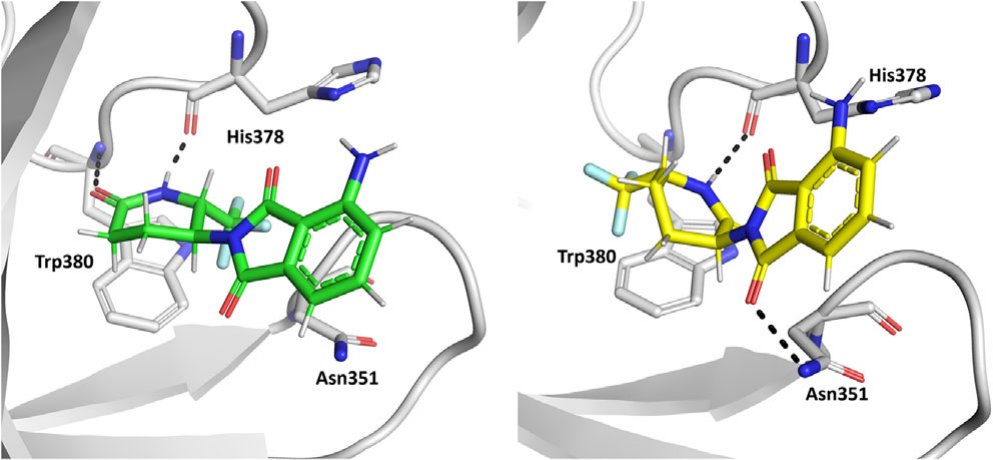
Docking poses of (*S*,*S*)‐**7** (in green sticks) and (3*S*,6*R*)‐**20** (in yellow sticks) in the binding pocket of cereblon (in gray cartoon). The ligand and the neighboring protein side chains are shown as stick models, colored according to the chemical atom type (blue, N; red, O; cyan, F). The enantiomers of **7** and **20** exhibiting the more favorable docking poses are shown. For clarity, only key amino acids are shown. H‐bonds are indicated by black dotted lines.

To assess whether cereblon recognition is retained following carbonyl‐to‐C–CF_3_ replacement, we evaluated the compounds **6**–**9**, **12**–**15**, **19**–**22**, and **24**–**26** using a competitive microscale thermophoresis (MST) assay. The fluorinated aminopiperidones showed no detectable affinity for this E3 ligase substrate receptor at the tested concentrations (up to 500 μM), as determined by a competitive MST assay employing a BODIPY–uracil reporter and the human thalidomide binding domain [42]. For comparison, pomalidomide exhibits an IC_50_ value of 13 μM in the same assay [42]. To rationalize the absence of measurable cereblon binding, we analyzed the interactions between the fluorinated aminopiperidones and cereblon by molecular docking. Docking studies were performed using a high‐resolution structure of cereblon in complex with thalidomide (PDB entry: 7BQU) [48]. In this structure, the glutarimide motif engages in a defined hydrogen‐bonding network, with the 6‐carbonyl group interacting with Trp380 and the NH group forming a hydrogen bond with His378. In addition, one of the phthalimide carbonyls interacts with Asn351 (Figure S1). Docking calculations for the representative 5‐phthalimido‐6‐CF_3_‐piperidones **7** and **13** and the 3‐phthalimido‐6‐CF_3_‐piperidones **20** and **24** revealed that the canonical hydrogen‐bonding network required for cereblon binding is disrupted in all cases. Specifically, the *trans* pomalidomide analog **7** loses the hydrogen bond with Asn351, while preserving the Trp380 and His378 interactions (Figure 4, left), whereas its *cis* counterpart **13** cannot be sterically accommodated within the cereblon‐binding site. Similarly, *trans* compound **20** fails to form a hydrogen bond with Trp380 (Figure 4, right), while its *cis* analog **24** is unable to fit sterically into the binding pocket.

Given that fluorophthalimide derivatives can exhibit cereblon‐independent anti‐angiogenic activity, we next evaluated the biological activity of the fluorinated aminopiperidones using established angiogenesis models. Human umbilical vein endothelial cells (HUVECs) can form hollow tube‐like structures when cultured upon biological basement membranes, such as ECMatrix. The formation of the tubules can then be used as an in vitro measurement of angiogenesis, with the extent of inhibition compared to a vehicle control corresponding to the potency of the anti‐angiogenic effects of the compounds. We have previously optimized the lattice assay for screening IMiD compounds and selected 10 and 100 μM as the screening doses to determine which compounds exhibit potent anti‐angiogenic activity in vitro [49]. All fluorinated aminopiperidones were tested at 10 μM, and compounds that did not exhibit potent anti‐angiogenic activity at 10 μM were further tested at 100 μM (Table 1, Figures S2–S3, Table S1). The glutarimide **30** [50] and *N*‐cyclohexyltetrafluorophthalimide **Gü1215** [51] were used as positive controls.

The CF_3_‐piperidones with tetrafluophthalimide fragments **9**, **15**, **22,** and **26** were found to significantly inhibit tube formation at 10 μM (>97% inhibition), benchmarked against the positive controls **30** (94.5% inhibition) and **Gü1215** (91.2%). Compounds with nitro‐, amino‐, and monofluoro‐phthalimide fragments exhibited low to modest antiangiogenetic activity in the tube formation assay regardless of the CF_3_‐piperidone regio‐ or stereoisomer (Table 1, Figures S2–S3, Table S1).

In the rat aorta ring assay, all tested compounds exhibited a clear concentration‐dependent suppression of angiogenic sprouting, with 15%–55% inhibition observed at 50 μM and 30%–70% inhibition at 100 μM (Table 1, Figures S4–S7, Table S2). Notably, compound **15** demonstrated potent anti‐angiogenic activity, achieving >50% inhibition at both tested concentrations. Relative to the vehicle control (0.5% DMSO), significant inhibition of microvessel outgrowth at 100 μM was observed for the tetrafluorophthalimide positive control **30** (65.3%), which is consistent with the previous report [50], and for both diastereomers of the 5‐(tetrafluorophthalimide)‐6‐CF_3_‐piperidone: **9** (63.8%) and **15** (68.4%). On the other hand, inhibition of microvessel outgrowth was lower for the diastereomeric 3‐(tetrafluorophthalimide)‐6‐CF_3_‐piperidones **22** and **26**. Notably, significant inhibition of microvessel outgrowth was observed for the amino and monofluoro analogs of *cis*‐5‐phthalimido‐6‐CF_3_‐piperidones **7** (69.5%) and **8** (62.8%). Overall, these results demonstrate that substantial anti‐angiogenic activity is retained across the fluorinated aminopiperidone series despite the absence of measurable cereblon binding. The underlying mechanism of activity of polyfluorinated compounds is still not completely understood, and further experiments are needed to pinpoint their pharmacological target(s). Beside the possibility of fluorine atoms to alter binding to potential targets, increased lipophilicity of these compounds and their enhanced membrane affinity might further aid the inhibition of tube formation via improved passive diffusion.

## 3 Conclusion

We designed, synthesized, and systematically evaluated fluorinated aminopiperidones as three‐dimensional, non‐glutarimide analogs of thalidomide. By replacing a single glutarimide carbonyl group with a stereogenic C*–CF_3_ unit, we accessed a previously underexplored chemical space encompassing all regio‐ and diastereomeric variants of 3‐ and 5‐phthalimido‐6‐trifluoromethyl‐2‐piperidones. The developed synthetic routes enabled reliable control over relative stereochemistry, and comprehensive NMR analyses allowed unambiguous structural assignment of all final compounds.

Experimental profiling revealed that carbonyl‐to‐C*–CF_3_ replacement leads to improvements in ADME‐related physicochemical properties, including increased aqueous solubility despite higher log*D* and enhanced membrane interaction characteristics, without substantially increasing PPB. These effects are consistent with sp^3^ enrichment and highlight the utility of this bioisosteric strategy for escaping planar chemical space. The glutarimide fragment is a highly conserved feature among reported cereblon binders, reflecting the stringent structural requirements for productive engagement with the cereblon binding pocket [52, 53]. Consistent with this, replacement of the glutarimide motif with a CF_3_‐piperidone scaffold disrupts the canonical hydrogen‐bonding network required for cereblon recognition, and the resulting fluorinated aminopiperidones did not exhibit measurable cereblon binding in MST assays. Accordingly, commercially available CF_3_‐piperidone building blocks should not be assumed to function as cereblon‐binding tool compounds without experimental validation. Importantly, several CF_3_‐piperidone derivatives, particularly those bearing tetrafluorophthalimide substituents, exhibited strong anti‐angiogenic activity in both in vitro and ex vivo assays, comparable to established glutarimide‐based controls. These findings support a cereblon‐independent mechanism of angiogenesis inhibition and underscore the potential of fluorinated thalidomide‐inspired scaffolds beyond targeted protein degradation [50].

## 4 Experimental Section

### 4.1 Chemistry

#### 4.1.1 General

The starting chemicals and solvents were obtained from vendors (ABCR, Acros, Apollo, BLDPharm, Fluorochem, Sigma–Aldrich, TCI), and were used without further purification. In particular, the compounds **27**–**29** were purchased from BLDPharm, and **30** was synthesized as previously described [50]. Reactions were conducted under an inert atmosphere using anhydrous solvents when required. For reactions that require heating, flask heating blocks were used. High‐pressure reactions were conducted using a Parr Instruments Model 4790 reactor under the specified temperature and pressure conditions. Analytical thin‐layer chromatography (TLC) was performed on Silica Gel 60F254 plates. Flash column chromatography was performed using Silica Gel 60 (40−63 μm). ^1^H NMR (400 MHz; internal Me_4_Si = 0 ppm), ^13^C NMR (100 MHz; internal Chloroform‐*d* = 77.16 ppm, DMSO‐*d*_6_ = 39.52 ppm), and ^19^F NMR (380 MHz, external CCl_3_F = 0 ppm) spectra were recorded on a Bruker AVANCE III 400 spectrometer (Bruker Corporation, Billerica, MA, USA) or Bruker AVANCE NEO 400 MHz NMR spectrometer. HRMS were obtained using Exactive Plus Orbitrap mass spectrometer (Thermo Fisher Scientific, Waltham, MA, USA). Purity was determined with UHPLC Thermo Scientific UltiMate 3000. The general method used a Waters Acquity UPLC HSS C18 SB column (2.1 × 50 mm, 1.8 μm) thermostated at 40 °C, with injection volume, 1 μL; sample, 0.2 mg/mL in MeCN; flow rate, 0.4 mL/min; ultraviolet (UV) detector λ, 220 or 254 nm; mobile phase A, 0.1% TFA (v/v) in water; and mobile phase B, MeCN. Gradient: 0–2 min 30% B, 2–5 min, 30%–90% B; 5–8 min, 90% B. Melting points were determined on Kofler heating stage microscope from Laica or Metler Toledo Melting Point System MP30.

#### 4.1.2 4‐Methylbenzenesulfinamide (1)

Oxalyl chloride (11.4 g, 89.2 mmol) was added dropwise to racemic *p*‐toluenesulfinic acid sodium salt (15.0 g, 84.0 mmol) in toluene at 0 °C [54]. After stirring for 1 h at room temperature, the reaction mixture was added to 25% aqueous solution of ammonia (85 mL) and EtOAc (85 mL) at 0 °C and then stirred at room temperature. After 1 h, the reaction mixture was diluted with EtOAc (85 mL), and the two layers were separated. The aqueous layer was extracted with EtOAc (2 × 85 mL). The combined EtOAc layers were washed with brine (85 mL), dried over Na_2_SO_4_, and concentrated under reduced pressure to afford the title compound as a white powder (8.73 g, 66.8% yield). ^1^H NMR (400 MHz, CDCl_3_‐*d*) δ 7.64 (d, *J* = 8.3 Hz, 2H), 7.32 (d, J = 8.0 Hz, 3H), 4.24 (s, 3H), 2.42 (s, 4H). The spectral data are in agreement with those previously reported in the literature [54].

#### 4.1.3 N‐(1‐Ethoxy‐2,2,2‐Trifluoroethyl)‐4‐Methylbenzenesulfinamide (2)

Tetraisopropyl orthotitanate (82.7 mL, 281 mmol) was added to a mixture of 1‐ethoxy‐2,2,2‐trifluoro‐ethanol (6.7 mL, 56.0 mmol) and **1** (8.73 g, 56.3 mmol), and the mixture was stirred at 70 °C for 24 h. EtOAc (160 mL) was added, and the resulting solution was added to a flask containing brine (400 mL) and stirred for 10 min [39]. The white sticky precipitate was removed by filtration through celite. The aqueous phase was extracted with EtOAc. The combined organic layers were concentrated under vacuum to afford yellow oil. Crude was purified by flash chromatography on silica, eluent EtoAc:Hex = 3:1, to afford the title compound (12.4 g, 78.5% yield). ^1^H NMR (400 MHz, CDCl_3_‐*d*) δ 7.64–7.54 (m, 2H), 7.33 (d, *J* = 8.0 Hz, 2H), 4.94 (ddd, *J* = 10.8, 7.7, 3.1 Hz, 1H), 4.56 (td, *J* = 9.3, 4.6 Hz, 1H), 4.20 (dq, *J* = 12.2, 6.1 Hz, 1H), 2.43 (d, *J* = 1.6 Hz, 3H), 1.31–1.19 (m, 5H), 1.13 (d, *J* = 6.0 Hz, 1H). ^19^F NMR (376 MHz, CDCl_3_‐*d*) δ −80.64 (d, *J* = 4.7 Hz), −81.02 (d, *J* = 4.6 Hz). The spectral data are in agreement with those previously reported in the literature [39].

#### 4.1.4 4‐Methyl‐N‐(1,1,1‐Trifluoro‐3‐Nitropropan‐2‐yl)benzenesulfinamide (3)

Freshly powdered NaOH (1.60 g, 40.4 mmol) was added to a slurry of **2** (2.27 g, 8.07 mmol) and 2.30 g of 4 Å molecular sieves in MeNO_2_ (100 mL) [39]. The reaction mixture was stirred at 110 °C for 46 h in a round bottom flask, equipped with a reflux condenser and chlorcalcium tube. Then, the solvent was evaporated, and the residue was suspended in EtOAc and filtered through a short pad of silica gel and then additionally purified by flash chromatography (EtOAc:Hex = 3:1) to afford the title compound, a 65:35 mixture of diastereomers, as a yellow oil (1.88 g, 78.6% yield). ^1^H NMR (400 MHz, CDCl_3_‐*d*) δ 7.58 (d, *J* = 8.0, 2H), 7.36 (d, *J* = 8.0 Hz, 2H), 5.21 (d, *J* = 10.8 Hz, 0.35H), 4.87–4.56 (m, 3.3H), 4.35 (ddd, *J* = 12.5, 10.9, 5.5 Hz, 0.35H), 2.44 (s, 3H). ^19^F NMR (376 MHz, CDCl_3_‐*d*) δ −74.05 (d, *J* = 7.3 Hz, 35%, minor), −74.48 (d, *J* = 6.7 Hz, 65%, major). The spectral data are in agreement with those previously reported in the literature [39].

#### 4.1.5 trans‐5‐Nitro‐6‐(trifluoromethyl)piperidin‐2‐One (4)

DBU (340 μL, 2.30 mmol) was added to a solution of **3** (1.70 g, 5.76 mmol) and ethyl acrylate (3.06 mL, 28.8 mmol) in acetonitrile (45 mL) at 0 °C [39]. The reaction mixture was stirred 24 h at 0 °C. After completion, the reaction mixture was filtered through a short pad of silica gel, and the silica gel was thoroughly washed with excess EtOAc, after which the solvent was changed to methanol (45 mL). Trifluoroacetic acid (2.24 mL, 28.5 mmol) was added, and the reaction mixture was stirred at room temperature overnight. The solvent was evaporated under reduced pressure, the residue was dissolved a 7N solution of NH_3_ in MeOH (45 mL), and the mixture was stirred at room temperature overnight. The volatiles were evaporated, and the crude was purified by flash chromatography (EtOAc:Hex = 1:5 → 1:2) to afford a yellow oil (259 mg, 21.2% yield). ^1^H NMR (400 MHz, CDCl_3_‐*d*) δ 7.82 (s, 1H), 4.98–4.84 (m, 2H), 2.76–2.63 (m, 1H), 2.59–2.34 (m, 3H). ^19^F NMR (376 MHz, CDCl_3_‐*d*) δ −75.88 (d, *J* = 7.2 Hz). The spectral data are in agreement with those previously reported in the literature [39].

#### 4.1.6 5‐Amino‐6‐(trifluoromethyl)piperidin‐2‐One (5)

Raney‐Ni slurry in water (1 mL) was dried by filtration through a sintered glass frit. The solid catalyst thus obtained was suspended in MeOH (5 mL) and added to a solution of **4** (259 mg, 1.22 mmol) in MeOH (5 mL). The solution was stirred in H_2_ atmosphere (1 atm) at room temperature for 24 h. The reaction mixture was filtered through celite and concentrated under reduced pressure to afford yellow‐brown oil (199 mg, 89% yield). ^1^H NMR (400 MHz, CDCl_3_‐*d*) δ 6.06 (s, 1H), 3.71 (ddd, *J* = 7.2, 5.2, 2.0 Hz, 1H), 3.45 (ddd, *J* = 8.9, 5.5, 3.7 Hz, 1H), 2.70–2.32 (m, 2H), 2.18–1.91 (m, 1H), 1.94–1.77 (m, 1H). ^19^F NMR (376 MHz, CDCl_3_‐*d*) δ −75.87 (d, *J* = 7.3 Hz). ^13^C NMR (101 MHz, CDCl_3_‐*d*) δ 171.3, 123.0 (q, *J* = 284.0 Hz), 61.0 (q, *J* = 27.2 Hz), 44.3, 28.1, 27.5. HRMS calcd. for C_6_H_9_F_3_N_2_O [M + H]^+^ 183.07397, found 183.07381.

#### 4.1.7 trans‐5‐(3‐Nitrophthalimido)‐6‐Trifluoromethyl‐2‐Piperidone (6)

A mixture of 3‐nitrophthalic anhydride (295 mg, 1.52 mmol), **5** (186 mg, 1,10 mmol), and sodium acetate (110 mg, 1.32 mmol) in acetic acid (4.4 mL) was refluxed at 110 °C for 3 h. After cooling, the reaction mixture was poured into water, resulting in the formation of a solid, which was then filtered. Crude was purified by flash chromatography (EtOAc:Hex = 2:1) to afford a white‐yellow solid (178 mg, 45.0% yield). ^1^H NMR (400 MHz, DMSO‐*d*_6_) δ 8.38 (d, *J* = 3.0 Hz, 1H), 8.31 (dd, *J* = 8.1, 0.8 Hz, 1H), 8.21 (dd, *J* = 7.5, 0.8 Hz, 1H), 8.13–8.05 (m, 1H), 4.72–4.48 (m, 2H), 2.44–2.23 (m, 3H), 2.05–1.96 (m, 1H). ^19^F NMR (376 MHz, DMSO‐*d*_6_) δ −76.21 (d, *J* = 7.4 Hz). ^13^C NMR (101 MHz, DMSO‐*d*_6_) δ 171.3, 165.2, 162.6, 144.4, 136.4, 133.2, 128.4, 127.0, 125.6 (q, *J* = 283.5 Hz), 122.7, 53.9 (q, *J* = 29.7 Hz), 43.5, 29.5, 23.1. HRMS calcd. for C_14_H_10_F_3_N_3_O_5_ [M + H]^+^ 358.06453, found 358.06410. *T*_m_ = 227.9 °C–232.0 °C.

#### 4.1.8 trans‐5‐(3‐Aminophthalimido)‐6‐Trifluoromethyl‐2‐Piperidone (7)

**6** (87 mg, 0.25 mmol) was dissolved in EtOAc (15 mL). The mixture was purged with argon. Pd/C (9 mg, 10 wt %) was then added to the solution, and the mixture was bubbled with hydrogen for 2 h. The reaction mixture was filtered through celite and concentrated under vacuum to afford a greenish yellow solid (73 mg, 89% yield). ^1^H NMR (400 MHz, DMSO‐*d*_6_) δ 8.30 (s, *J* = 8.5 Hz, 1H), 7.46 (dd, *J* = 8.4, 7.0 Hz, 1H), 7.00 (dd, *J* = 7.7, 3.9 Hz, 2H), 6.55 (s, 2H), 4.61–4.48 (m, 2H), 2.46–2.23 (m, 3H), 1.97–1.89 (m, 1H). ^19^F NMR (376 MHz, DMSO‐*d*_6_) δ −76.06 (d, *J* = 6.4 Hz). ^13^C NMR (101 MHz, DMSO‐*d*_6_) δ 171.0, 168.5, 167.3, 146.2, 135.4, 131.9, 124.8 (q, *J* = 282.3 Hz), 121.7, 111.0, 108.4, 53.8 (q, *J* = 29.0 Hz), 42.8, 29.9, 23.6. HRMS calcd. for C_14_H_12_F_3_N_3_O_3_ [M − H]^−^ 326.07580, found 326.07601. *T*_m_ = 231.2 °C–234.6 °C.

#### 4.1.9 trans‐5‐(3‐Fluorophthalimido)‐6‐Trifluoromethyl‐2‐Piperidone (8)

A mixture of 3‐fluorophthalic anhydride (94 mg, 0.57 mmol), **5** (69 mg, 0.38 mmol), and sodium acetate (37 mg, 0.46 mmol) in acetic acid (1.5 mL) was refluxed at 110 °C 6 h. After cooling, the reaction mixture was poured into water, resulting in the formation of a solid, which was then filtered. Crude was purified by flash chromatography to afford a white solid (48 mg, 54% yield). ^1^H NMR (400 MHz, CDCl_3_‐*d*_6_) δ 7.79 (ddd, *J* = 8.3, 7.4, 4.3 Hz, 1H), 7.72 (d, *J* = 7.0 Hz, 1H), 7.44 (td, *J* = 8.6, 0.8 Hz, 1H), 6.22 (s, 1H), 4.90–4.35 (m, 2H), 2.83–2.38 (m, 3H), 1.98 (ddd, *J* = 12.0, 5.8, 3.1 Hz, 1H). ^19^F NMR (376 MHz, CDCl_3_‐*d*_6_) δ −77.20 (d, *J* = 5.6 Hz), −111.72 (dd, *J* = 8.6, 4.3 Hz). ^13^C NMR (101 MHz, CDCl_3_‐*d*_6_) δ 170.7, 166.2 (d, *J* = 2.9 Hz), 164.1 (d, *J* = 1.3 Hz), 158.0 (d, *J* = 267.3 Hz), 137.4 (d, *J* = 7.7 Hz), 133.5, 124.1 (q, *J* = 282.8 Hz), 123.2 (d, *J* = 19.6 Hz), 120.2 (d, *J* = 3.8 Hz), 117.3 (d, *J* = 12.5 Hz), 54.2 (q, *J* = 29.8 Hz), 44.3, 30.3, 24.3. HRMS calcd. for C_14_H_10_F_4_N_2_O_3_ [M + H]^+^ 331.07003, found 331.06921. *T*_m_ = 241.3 °C–244.6 °C.

#### 4.1.10 trans‐5‐(3,4,5,6‐Tetrafluorophthalimido)‐6‐Trifluoromethyl‐2‐Piperidone (9)

A mixture of 4,5,6,7‐tetrafluoro phthalic anhydride (180 mg, 0.812 mmol), **5** (100 mg, 0.55 mmol), and sodium acetate (54 mg, 0.66 mmol) in acetic acid (2.2 mL) was refluxed at 110 °C for 3 h. After cooling, the reaction mixture was poured into water, resulting in the formation of a solid, which was then filtered to afford a white solid (140 mg, 67% yield). ^1^H NMR (400 MHz, DMSO‐*d*_6_) δ 8.38 (d, *J* = 3.3 Hz, 1H), 4.70–4.42 (m, 2H), 2.44–2.19 (m, 3H), 1.99 (tt, *J* = 7.0, 3.2 Hz, 1H). ^19^F NMR (376 MHz, DMSO‐*d*_6_) δ −76.22 (d, *J* = 7.3 Hz), −139.18 (d, *J* = 12.8 Hz), −144.08 (d, *J* = 12.7 Hz). ^13^C NMR (101 MHz, DMSO‐*d*_6_) δ 171.2, 161.8, 145.5, 143.8, 142.9, 141.2, 124.8 (q, *J* = 282.6 Hz), 113.7, 53.8 (q, *J* = 29.3 Hz), 43.7, 29.5, 23.0. HRMS calcd. for C_14_H_8_O_3_N_2_F_7_ [M + H]^+^ 385.04177, found 385.04133. *T*_m_ = 225.1 °C–226.4 °C.

#### 4.1.11 cis‐5‐Amino‐6‐(trifluoromethyl)piperidin‐2‐One (11)

To a solution of 5‐nitro‐6‐(trifluoromethyl)pyridin‐2(1*H*)‐one (100 mg, 0.48 mmol) in MeOH (5 mL), Pd/C (10 mg, 10 wt %) was added. Solution was hydrogenated for 2 days in pressure reactor (80 °C, 60 atm H_2_). The reaction mixture was filtered through celite and concentrated under reduced pressure. Crude was purified by flash chromatography (DCM:MeOH = 6:1) to afford a white solid (41 mg, 47% yield). ^1^H NMR (400 MHz, CDCl_3_‐*d*) δ 5.78 (s, 1H), 4.01–3.90 (m, 1H), 3.62 (dd, *J* = 7.5, 3.8 Hz, 1H), 2.64 (ddd, *J* = 18.4, 8.8, 6.7 Hz, 1H), 2.46 (ddd, *J* = 18.3, 6.9, 5.4 Hz, 1H), 2.10–1.89 (m, 2H). ^19^F NMR (376 MHz, CDCl_3_‐*d*) δ −71.91 (d, *J* = 7.6 Hz). ^13^C NMR (101 MHz, CDCl_3_‐*d*) δ 171.6, 126.0 (d, *J* = 281.4 Hz), 58.3 (q, J = 27.7 Hz), 44.8, 27.5, 27.4.

#### 4.1.12 cis‐5‐(3‐Nitrophthalimido)‐6‐Trifluoromethyl‐2‐Piperidone (12)

A mixture of 3‐nitrophthalic anhydride (160 mg. 0.82 mmol), **11** (100 mg, 0.55 mmol), and sodium acetate (54 mg, 0.66 mmol) in acetic acid (2.2 mL) was refluxed at 110 °C for 3 h. After cooling, the reaction mixture was poured into water which was extracted with EtOAc. The organic phase was washed with brine and dried over Na_2_SO_4_. The combined organic layers were concentrated under a vacuum. Crude was purified by flash chromatography to afford a white solid (90 mg, 45% yield). ^1^H NMR (400 MHz, CDCl_3_‐*d*) δ 8.16 (ddd, *J* = 7.7, 3.2, 0.9 Hz, 2H), 7.98 (t, *J* = 7.8 Hz, 1H), 7.11 (d, *J* = 3.8 Hz, 1H), 4.88–4.74 (m, 1H), 4.33–4.16 (m, 1H), 3.39 (qd, *J* = 13.3, 6.3 Hz, 1H), 2.72 (ddd, *J* = 18.3, 6.4, 2.2 Hz, 1H), 2.55 (ddd, *J* = 18.5, 11.6, 7.4 Hz, 1H), 2.24–2.12 (m, 1H). ^19^F NMR (376 MHz, CDCl_3_‐*d*) δ −71.80 (d, *J* = 7.4 Hz). ^13^C NMR (101 MHz, CDCl_3_‐*d*) δ 165.3, 162.7, 145.4, 136.1, 133.6, 129.3, 127.6, 125.7 (q, *J* = 283.7 Hz), 122.9, 53.4 (q, *J* = 29.3 Hz), 47.6, 30.7, 20.2. HRMS calcd. for C_14_H_10_F_3_N_3_O_5_ [M − H]^−^ 356.04998, found 356.05026, *T*_m_ = 248.6 °C–252.0 °C.

#### 4.1.13 cis‐5‐(3‐Aminophthalimido)‐6‐Trifluoromethyl‐2‐Piperidone (13)

**12** (60 mg, 0.17 mmol) was dissolved in EtOAc (15 mL). The mixture was purged with argon. Pd/C (6 mg, 10 wt %) was then added to the solution, and the mixture was bubbled with hydrogen for 2 h. Pd/C was filtered over celite and concentrated under vacuum. Crude was purified by flash chromatography (EtOAc:Hex = 2:1) and then with reverse phase chromatography to afford a yellow solid (15 mg, 27% yield). ^1^H NMR (400 MHz, DMSO‐*d*_6_) δ 8.32 (d, *J* = 4.4 Hz, 1H), 7.44 (dd, *J* = 8.4, 7.0 Hz, 1H), 7.05–6.94 (m, 2H), 6.54 (s, 2H), 4.68 (dd, *J* = 12.4, 5.0 Hz, 1H), 4.10 (dq, *J* = 13.2, 6.6 Hz, 1H), 3.15–3.00 (m, 1H), 2.51–2.39 (m, 1H), 2.07 (d, *J* = 7.9 Hz, 1H). ^19^F NMR (376 MHz, DMSO‐*d*_6_) δ −70.51 (d, *J* = 8.2 Hz). ^13^C NMR (101 MHz, DMSO‐*d*_6_) δ 169.8, 169.1, 167.8, 146.7, 135.4, 131.7, 124.9 (q, J = 284.3 Hz), 121.6, 110.8, 108.3, 52.4 (q, J = 27.5 Hz), 46.0, 30.3, 19.7. HRMS calcd. for C_14_H_11_F_3_N_3_O_2_ [M − H]^−^ 326.07580, found 326.07593, *T*_m_ = 238 °C–241 °C.

#### 4.1.14 cis‐5‐(3‐Fluorophthalimido)‐6‐Trifluoromethyl‐2‐Piperidone (14)

A mixture of 3‐fluorophthalic anhydride (91 mg, 0.82 mmol), **11** (100 mg, 0.55 mmol), and sodium acetate (54 mg, 0.66 mmol) in acetic acid (2.2 mL) was refluxed at 110 °C 3 h. After cooling, the reaction mixture was poured into water which was extracted with EtOAc. The organic phase was washed with brine and dried over Na_2_SO_4_. The combined organic layers were concentrated under a vacuum. Crude was purified by flash chromatography (DCM:MeOH = 25:1) to afford a white solid (72 mg, 40% yield). ^1^H NMR (400 MHz, CDCl_3_‐*d*) δ 7.78 (ddd, *J* = 8.2, 7.3, 4.3 Hz, 1H), 7.72–7.68 (m, 1H), 7.43 (td, *J* = 8.5, 0.9 Hz, 1H), 6.64 (d, *J* = 3.8 Hz, 1H), 4.79 (dddd, *J* = 13.3, 5.4, 3.9, 1.6 Hz, 1H), 4.20 (dd, *J* = 11.1, 6.2 Hz, 1H), 3.48–3.34 (m, 1H), 2.77 (ddd, *J* = 18.3, 6.3, 2.1 Hz, 1H), 2.57 (ddd, *J* = 18.6, 11.7, 7.4 Hz, 1H), 2.16 (dd, *J* = 12.2, 5.3 Hz, 1H). ^19^F NMR (376 MHz, CDCl_3_‐*d*) δ −71.89 (d, *J* = 7.7 Hz), −112.08 (dd, *J* = 8.6, 4.4 Hz). ^13^C NMR (101 MHz, CDCl_3_‐*d*) δ 170.5, 166.7 (d, *J* = 3.2 Hz), 164.6, 157.9 (d, *J* = 267.1 Hz), 137.3 (d, *J* = 7.7 Hz), 133.6, 127.2 (q, *J* = 283.7 Hz), 123.1 (d, *J* = 19.8 Hz), 120.1 (d, *J* = 3.7 Hz), 117.3 (d, *J* = 12.4 Hz), 53.6 (q, *J* = 29.0 Hz), 47.1, 30.9, 20.2. HRMS calcd. For C_14_H_10_F_4_N_2_O_3_ [M + H]^+^ 331.07003, found 331.06956, *T*_m_ = 218 °C–222 °C.

#### 4.1.15 cis‐5‐(3,4,5,6‐Tetrafluorophthalimido)‐6‐Trifluoromethyl‐2‐Piperidone (15)

A mixture of 4,5,6,7‐tetrafluoro phthalic anhydride (114 mg, 0.52 mmol), **11** (63 mg, 0.34 mmol), and sodium acetate (34 mg, 0.41 mmol) in acetic acid (1.4 mL) was refluxed at 110 °C for 4 h. After cooling, the reaction mixture was poured into water, resulting in the formation of a solid, which was then filtered to afford a white solid (110 mg, 55% yield). ^1^H NMR (400 MHz, DMSO‐*d*_6_) δ 8.39 (d, *J* = 4.5 Hz, 1H), 4.81–4.70 (m, 1H), 4.17 (qt, *J* = 8.3, 4.7 Hz, 1H), 2.99 (qd, *J* = 12.8, 7.1 Hz, 1H), 2.61–2.53 (m, 1H), 2.52–2.40 (m, 4H), 2.09 (dd, *J* = 12.3, 6.6 Hz, 1H). ^19^F NMR (376 MHz, DMSO‐*d*_6_) δ −70.45 (d, *J* = 7.9 Hz, 3 F), (−138.52)–(−139.12) (m, 2F), (−143.46)–(−143.98) (m, 2F). ^13^C NMR (101 MHz, DMSO‐*d*_6_) δ 169.7, 162.23, 145.8, 143.9, 143.1, 141.3, 124.7 (q, *J* = 284.1 Hz), 113.2, 52.3 (q, *J* = 28.0 Hz), 46.8, 30.0, 19.3. HRMS calcd. for C_14_H_7_F_7_N_2_O_3_ [M + H]^+^ 385.04177, found 385.04141 *T*_m_ = 224.2 °C–228.6 °C.

#### 4.1.16 3‐Amino‐6‐(trifluoromethyl)piperidin‐2‐One (17,18)

To a solution of 3‐nitro‐6‐(trifluoromethyl)pyridin‐2(1*H*)‐one (2.25 g, 10.8 mmol) in MeOH (10 mL), Pd/C (225 mg, 10 wt %) was added [32]. The solution was stirred for 2 days in a pressure reactor (80 °C, 60 atm). The reaction mixture was filtered through celite and concentrated under reduced pressure. The residue was purified by flash chromatography on silica (DCM:MeOH:NH_3_(aq) = 6:1:0.1) to afford a white solid (1.67 g, 85.0% yield). Isolated as a 67:33 mixture of diastereomers. ^1^H NMR (400 MHz, CDCl_3_‐*d*) δ 7.11 (s, 0.67H), 6.91 (s, 0.37H), 3.97 (dt, *J* = 10.5, 6.2 Hz, 0.37H), 3.94–3.87 (m, 0.67H), 3.34 (dt, *J* = 11.0, 6.0 Hz, 1H), 2.33–2.05 (m, 3.H), 1.97 (s, 2H), 1.91–1.79 (m, 1.2H), 1.66 (ddd, *J* = 14.2, 11.5, 2.9 Hz, 0.4H). ^19^F NMR (376 MHz, CDCl_3_‐*d*) δ −76.66 (d, *J* = 7.6 Hz, 67% major), −78.73 (d, *J* = 6.3 Hz, 33% minor). ^13^C NMR (101 MHz, CDCl_3_‐*d*) δ 175.3 (major), 175.2 (minor), 125.0 (q, *J* = 282.5 Hz, major), 123.0 (q, *J* = 280.5 Hz, minor), 54.7 (q, *J* = 31.3 Hz, major), 53.1 (q, *J* = 30.5 Hz, minor), 51.3, 51.2 (major), 27.8, 26.1 (major), 20.6 (d, *J* = 2.2 Hz, minor), 20.0 (d, *J* = 1.3 Hz major).

#### 4.1.17 trans‐3‐(3‐Nitrophthalimido)‐6‐Trifluoromethyl‐2‐Piperidone (19)

A mixture of 3‐nitrophthalic anhydride (158 mg. 0.82 mmol), the above mixture of diastereomers **17** and **18** (100 mg, 0.55 mmol), and sodium acetate (54 mg, 0.66 mmol) in acetic acid (2.2 mL) was refluxed at 110 °C for 6 h. After cooling, the reaction mixture was poured into water, resulting in the formation of a solid, which was then filtered. The aqueous phase was extracted with EtOAc. The organic phase was washed with brine and dried over Na_2_SO_4_. The combined organic layers were concentrated under a vacuum. The crude mixture, containing both diastereomers in a 41:59 ratio, was purified by flash chromatography (DCM:MeOH = 25:1) to afford a first‐eluting compound as white solid (66 mg, 34% yield). ^1^H NMR (400 MHz, DMSO‐*d*_6_) δ 8.67 (s, 1H), 8.34 (dd, *J* = 8.1, 0.9 Hz, 1H), 8.23 (dd, *J* = 7.6, 0.9 Hz, 1H), 8.16–8.00 (m, 1H), 4.73 (dd, *J* = 12.0, 5.8 Hz, 1H), 4.39–4.27 (m, 1H), 2.36 (qd, *J* = 12.8, 3.4 Hz, 1H), 2.26–2.09 (m, 1H), 2.09 (dq, *J* = 13.2, 4.3 Hz, 1H), 1.96 (tdd, *J* = 13.5, 10.3, 3.4 Hz, 1H).^19^F NMR (376 MHz, DMSO‐*d*_6_) δ −76.41 (d, *J* = 6.9 Hz). ^13^C NMR (101 MHz, DMSO‐*d*_6_) δ 167.6, 165.4, 162.7, 144.4, 136.78, 133.1, 128.8, 127.2, 123.7 (q, *J* = 282.0 Hz), 122.6, 53.0 (q, *J* = 30.3 Hz), 49.1, 22.9, 20.3. HRMS calcd. for C_14_H_11_O_5_N_3_F_3_ [M + H]^+^ 358.0645; found 358.0636, *T*_m_ = 237.5 °C–240.9 °C.

#### 4.1.18 trans‐3‐(3‐Aminophthalimido)‐6‐Trifluoromethyl‐2‐Piperidone (20)

**19** (80 mg, 0.22 mmol) was dissolved in EtOAc (15 mL). The mixture was purged with argon. Pd/C (8 mg, 10 wt %) was then added to the solution, and the mixture was bubbled with hydrogen for 2 h. The reaction mixture was filtered through celite and concentrated under reduced pressure to afford a yellow solid (71 mg, 99% yield). ^1^H NMR (400 MHz, DMSO‐*d*_6_) δ 8.55 (s, 1H), 7.46 (dd, *J* = 8.4, 7.0 Hz, 1H), 7.11–6.92 (m, 2H), 6.49 (s, 2H), 4.58 (dd, *J* = 12.0, 5.7 Hz, 1H), 4.41–4.22 (m, 1H), 2.39 (qd, *J* = 12.8, 3.4 Hz, 1H), 2.24–2.16 (m, 1H), 2.04 (dt, *J* = 13.1, 4.7 Hz, 1H), 1.93 (tdd, *J* = 13.5, 10.3, 3.5 Hz, 1H). ^19^F NMR (376 MHz, DMSO‐*d*_6_) δ −76.49 (d, *J* = 7.0 Hz). ^13^C NMR (101 MHz, DMSO‐*d*_6_) δ 168.8, 168.3, 167.6, 146.6, 135.4, 132.1, 126.3 (q, *J* = 282.0 Hz), 121.55, 110.9, 108.7, 53.0 (q, *J* = 30.4 Hz), 48.2, 23.3, 20.5. HRMS calcd. for C_14_H_12_F_3_N_3_O_3_ [M + H]^+^ 328.09035, found 328.09013, *T*_m_ = 284.6 °C–286.0 °C.

#### 4.1.19 trans‐3‐(3‐Fluorophthalimido)‐6‐Trifluoromethyl‐2‐Piperidone (21)

A mixture of 3‐fluorophthalic anhydride (166 mg, 1.64 mmol), the above mixture of diastereomers **17** and **18** (200 mg, 1.10 mmol), and sodium acetate (108 mg, 1.30 mmol) in acetic acid (4.4 mL) was refluxed at 110 °C 12 h. After cooling, the reaction mixture was poured into water, resulting in the formation of a solid, which was then filtered. The crude mixture, containing both diastereomers in a 18:82 ratio, was purified by flash chromatography (DCM:MeOH = 30:1) to afford a the first‐eluting diastereomer as a white solid (114 mg, 32% yield). ^1^H NMR (400 MHz, DMSO‐*d*_6_) δ 8.63 (s, 1H), 7.93 (ddd, *J* = 8.4, 7.3, 4.5 Hz, 1H), 7.81–7.67 (m, 2H), 4.68 (dd, *J* = 12.1, 5.7 Hz, 1H), 4.34 (dt, *J* = 10.3, 6.1 Hz, 1H), 2.38 (qd, *J* = 12.8, 3.3 Hz, 1H), 2.25–2.16 (m, 1H), 2.07 (dt, *J* = 13.2, 4.8 Hz, 1H), 1.95 (tdd, *J* = 13.5, 10.4, 3.5 Hz, 1H).^19^F NMR (376 MHz, DMSO‐*d*_6_) δ −76.45 (d, *J* = 7.0 Hz), −114.87 (dd, *J* = 9.3, 4.8 Hz). ^13^C NMR (101 MHz, DMSO‐*d*_6_) δ 167.8, 166.3 (d, *J* = 2.9 Hz), 164.1, 156.8 (d, *J* = 262.1 Hz), 137.9 (d, *J* = 8.0 Hz), 133.5, 126.2 (q, *J* = 276.5 Hz), 122.9 (d, *J* = 19.7 Hz), 119.9 (d, *J* = 3.4 Hz), 117.1 (d, *J* = 12.5 Hz), 52.9 (q, *J* = 30.2 Hz), 48.8, 23.0, 20.3. HRMS calcd. for C_14_H_10_F_4_N_2_O_3_ [M + H]^+^ 331.07003, found 331.06949, *T*_m_ = 219.5 °C–222.6 °C.

#### 4.1.20 trans‐3‐(3,4,5,6‐Tetrafluorophthalimido)‐6‐Trifluoromethyl‐2‐Piperidone (22)

A mixture of 4,5,6,7‐tetrafluoro phthalic anhydride (361 mg, 1.64 mmol), the above mixture of diastereomers **17** and **18** (200 mg, 1.10 mmol), and sodium acetate (108 mg, 1.30 mmol) in acetic acid (4.4 mL) was refluxed at 110 °C for 12 h. After cooling, the reaction mixture was poured into water, resulting in the formation of a solid, which was then filtered. The crude mixture, containing both diastereomers in a 47:58 ratio, was purified by flash chromatography (DCM:MeOH = 40:1 to 30:1) to afford a first‐eluting compound as white solid (156 mg, 37% yield). ^1^H NMR (400 MHz, DMSO‐*d*_6_) δ 8.68 (s, 1H), 4.71 (dd, *J* = 12.1, 5.8 Hz, 1H), 4.33 (q, *J* = 7.8 Hz, 1H), 2.34 (qd, *J* = 12.7, 3.3 Hz, 1H), 2.27–2.13 (m, 1H), 2.09 (dt, *J* = 8.0, 3.9 Hz, 1H), 1.97 (tdd, *J* = 13.5, 10.4, 3.4 Hz, 1H). ^19^F NMR (376 MHz, DMSO‐*d*_6_) δ −76.45 (d, *J* = 6.9 Hz), −138.51 (d, *J* = 13.5 Hz), −143.99 (d, *J* = 13.6 Hz). ^13^C NMR (101 MHz, DMSO‐*d*_6_) δ 167.3, 162.0, 145.7, 144.0, 143.1, 141.4, 124.8 (q, J = 280.9 Hz), 113.4 (d, J = 10.1 Hz), 52.9 (q, J = 30.4 Hz), 49.3, 22.8, 20.2. HRMS calcd. for C_14_H_7_F_7_N_2_O_3_ [M + H]^+^ 385.04177, found 385.04130, *T*_m_ = 241 °C–246 °C.

#### 4.1.21 cis‐3‐(3‐Nitrophthalimido)‐6‐Trifluoromethyl‐2‐Piperidone (23)

A mixture of 3‐nitrophthalic anhydride (158 mg, 0.82 mmol), the above mixture of diastereomers **17** and **18** (100 mg, 0.55 mmol), and sodium acetate (54 mg, 0.66 mmol) in acetic acid (2.2 mL) was refluxed at 110 °C for 6 h. After cooling, the reaction mixture was poured into water, resulting in the formation of a solid, which was then filtered. The aqueous phase was extracted with EtOAc. The organic phase was washed with brine and dried over Na_2_SO_4_. The combined organic layers were concentrated under a vacuum. Crude was purified by flash chromatography (DCM:MeOH = 25:1). The compound was not isolated in sufficient quantities for full characterization. ^1^H NMR (400 MHz, DMSO‐*d*_6_) δ 8.68 (d, *J* = 3.5 Hz, 1H), 8.34 (dd, *J* = 8.1, 0.9 Hz, 1H), 8.22 (dd, *J* = 7.5, 0.9 Hz, 1H), 8.11 (dd, *J* = 8.1, 7.5 Hz, 1H), 4.80 (dd, *J* = 11.8, 6.3 Hz, 1H), 4.14 (t, *J* = 8.9 Hz, 1H), 2.50 (d, *J* = 1.9 Hz, 1H), 2.39–2.26 (m, 1H), 2.17–2.08 (m, 1H), 1.94 (dd, *J* = 12.9, 6.1 Hz, 1H). ^19^F NMR (376 MHz, DMSO‐*d*_6_) δ −74.07 (d, *J* = 8.3 Hz). ^13^C NMR (101 MHz, DMSO) δ 167.8, 165.7, 163.1, 144.9, 137.2, 133.6, 129.2, 127.6, 124.6 (q, *J* = 285.6 Hz), 123.0, 51.6 (q, *J* = 29.7 Hz), 49.5, 21.8, 20.1.

#### 4.1.22 cis‐3‐(3‐Aminophthalimido)‐6‐Trifluoromethyl‐2‐Piperidone (24)

A 41:59 mixture of diastereomers **19** and **23** (132 mg, 0.37 mmol) was dissolved in EtOAc (15 mL). The mixture was purged with argon. Pd/C (13 mg, 10 wt %) was then added to the solution, and the mixture was bubbled with hydrogen for 2.5 h. The reaction mixture filtered over celite and concentrated under vacuum. The crude mixture, containing both diastereomers in a 68:32 ratio, was purified by flash chromatography (DCM:MeOH:NH_3_(aq) = 40:1:0.1) to afford a second‐eluting compound as yellow solid (45 mg, 37% yield). ^1^H NMR (400 MHz, DMSO‐*d*_6_) δ 8.55 (s, 1H), 7.45 (dd, *J* = 8.4, 7.0 Hz, 1H), 6.98 (dd, *J* = 9.7, 7.6 Hz, 2H), 6.49 (s, 2H), 4.64 (dd, *J* = 12.0, 6.2 Hz, 1H), 4.12 (s, 1H), 2.49–2.40 (m, 1H), 2.31 (s, 1H), 2.10 (d, *J* = 15.0 Hz, 1H), 1.92–1.84 (m, 1H). ^19^F NMR (376 MHz, DMSO‐*d*_6_) δ −74.10 (d, *J* = 8.4 Hz). ^13^C NMR (101 MHz, DMSO‐*d*_6_) δ 168.7, 168.0, 167.5, 146.6, 135.3, 132.2, 127.0 (q, *J* = 282.5 Hz), 121.5, 110.8, 108.8, 51.3 (q, *J* = 30.0 Hz), 48.1, 21.8, 19.9. HRMS calcd. For C_14_H_12_F_3_N_3_O_3_ [M + H]^+^ 328.09035, found 328.08985, *T*_m_ = 295.7 °C–299.6 °C.

#### 4.1.23 cis‐3‐(3‐Fluorophthalimido)‐6‐Trifluoromethyl‐2‐Piperidone (25)

A mixture of 4‐fluorophthalic anhydride (166 mg, 1.64 mmol), the above mixture of diastereomers **17** and **18** (200 mg, 1.10 mmol), and sodium acetate (108 mg, 1.30 mmol) in acetic acid (4.4 mL) was refluxed at 110 °C 12 h. After cooling, the reaction mixture was poured into water, resulting in the formation of a solid, which was then filtered. The crude mixture, containing both diastereomers, was purified by flash chromatography (DCM:MeOH = 30:1) to afford a second‐eluting compound as white solid (96 mg, 27% yield). ^1^H NMR (400 MHz, DMSO‐*d*_6_) δ 8.64 (d, *J* = 3.6 Hz, 1H), 7.93 (ddd, *J* = 8.4, 7.3, 4.5 Hz, 1H), 7.86–7.65 (m, 2H), 4.75 (dd, *J* = 11.9, 6.3 Hz, 1H), 4.15 (d, *J* = 9.5 Hz, 1H), 2.44 (d, *J* = 12.6 Hz, 1H), 2.40–2.26 (m, 1H), 2.11 (d, *J* = 14.7 Hz, 1H), 1.99–1.85 (m, 1H). ^19^F NMR (376 MHz, DMSO‐*d*_6_) δ −74.07 (d, *J* = 8.5 Hz), −114.96. ^13^C NMR (101 MHz, DMSO‐*d*_6_) δ 167.5, 166.2 (d, *J* = 2.8 Hz), 164.0, 156.8 (d, *J* = 262.2 Hz), 138.0 (d, *J* = 7.8 Hz), 133.6, 125.6 (q, *J* = 283.6 Hz), 122.9 (d, *J* = 19.6 Hz), 120.0, 117.1 (d, *J* = 12.5 Hz), 51.2 (q, *J* = 29.9 Hz), 48.7, 21.5, 19.7. HRMS calcd. For C_14_H_10_F_4_N_2_O_3_ [M + H]^+^ 331.07003, found 331.06955, *T*_m_ = 274.9 °C–283.2 °C.

#### 4.1.24 cis‐3‐(3,4,5,6‐Tetrafluorophthalimido)‐6‐Trifluoromethyl‐2‐Piperidone (26)

A mixture of 4,5,6,7‐tetrafluoro phthalic anhydride (361 mg, 1.64 mmol), the above mixture of diastereomers **17** and **18** (200 mg, 1.10 mmol), and sodium acetate (108 mg, 1.30 mmol) in acetic acid (4.4 mL) was refluxed at 110 °C for 12 h. After cooling, the reaction mixture was poured into water, resulting in the formation of a solid, which was then filtered. The crude mixture, containing both diastereomers was purified by flash chromatography (DCM:MeOH = 40:1 to 30:1) to afford a second‐eluting compound as white solid. (138 mg, 33% yield). ^1^H NMR (400 MHz, DMSO‐*d*_6_) δ 8.68 (d, *J* = 3.6 Hz, 1H), 4.78 (dd, *J* = 11.6, 6.2 Hz, 1H), 4.15 (d, *J* = 9.2 Hz, 1H), 2.49–2.23 (m, 2H), 2.18–2.07 (m, 1H), 1.93 (dd, *J* = 12.2, 6.6 Hz, 1H). ^19^F NMR (376 MHz, DMSO‐*d*_6_) δ −74.09 (d, *J* = 8.2 Hz), −138.59, −144.07 (d, *J* = 13.8 Hz). ^13^C NMR (101 MHz, DMSO‐*d*_6_) δ 167.0, 161.9, 145.67, 144.1, 143.1, 141.4, 125.5 (q, *J* = 283.9 Hz), 113.4, 51.2 (q, *J* = 30.1 Hz), 49.1, 21.3, 19.6. HRMS calcd. for C_14_H_7_F_7_N_2_O_3_ [M + H]^+^ 385.04177, found 385.04141, *T*_m_ = 273.2 °C–279.9 °C.

### 4.2 Determination of Physicochemical Properties and Biological Assays

#### 4.2.1 Thermodynamic Solubility

Determinations of solubility were performed by a contract research organization WuXi AppTec, China. Two‐milligram samples were weighed into lower chamber of Whatman Mini‐UniPrep vials (Cytiva), and 450 µL of buffer (10 mM PBS, pH 7.4) was added into each chamber. After buffer addition, filter pistons of Mini‐UniPrep vials were placed and compressed to the position of the liquid level to allow for contact of buffer and compound with the filter during incubation. The thermodynamic solubility samples were vortexed for 2 min and incubated at room temperature (25 °C ± 2 °C) for 24 h with shaking at 600 rpm. Mini‐UniPreps were compressed to prepare the filtrates for injection into HPLC system. All vials were inspected for visible undissolved material before filtering and for leakage after filtering. The supernatant was diluted with buffer by a factor of 100 to make diluents. UV calibration standard solutions (1 μM, 20 μM, 200 μM) were injected from low to high concentration, followed by the diluents and thermodynamic solubility supernatant. Testing samples were injected in duplicate. The UV chromatograms were integrated, the calibration equation was simulated, and thermodynamic solubility was calculated. HPLC chromatograms were acquired on Agilent 1200 instrument, equipped with diode array detector, using Xbridge C18 column.

#### 4.2.2 Distribution Coefficient, logD

Determinations of log*D* were performed by a contract research organization WuXi AppTec, China. 1‐Octanol‐saturated buffer was prepared by vigorously shaking a mixture of 1‐octanol (10 mL) and 100 mM phosphate buffer, pH 7.4 (100 mL), and letting it stand overnight at room temperature. The buffer layer was separated and stored at room temperature for a maximum of 1 week. Buffer‐saturated 1‐octanol was prepared by vigorously shaking a mixture of 100 mM phosphate buffer, pH 7.4 (10 mL), and 1‐octanol (100 mL) and letting it stand overnight at room temperature. The octanol layer was separated and stored at room temperature for a maximum of 1 week. Internal standard (IS) was prepared as a 1.0 mg/mL solution in acetonitrile. Four hundred two microliters of IS solution and 50 mL of 1‐octanol‐saturated buffer were dissolved in 949 mL of methanol/water 1:1 to get 1‐octanol IS solution. Four hundred twenty‐one microliters of IS solution and 5.263 mL of buffer‐saturated 1‐octanol were dissolved in 994 mL of methanol/water 1:1 to get buffer (pH 7.4) solution. Test compounds (10 mM in DMSO; 2 μL/well) were transferred in duplicate from storage tubes to the 96‐well polypropylene cluster tubes. Buffer‐saturated 1‐octanol (150 µL/well) and 1‐octanol saturated buffer (150 µL/well) were added to each well. Each of the tubes was vigorously mixed on their sides for 1 min, shaken 1 h at a speed of 600 rpm at 28 °C, and then centrifuged at 4000 rpm for 10 min. The buffer layer sample was diluted by a factor of X‐fold and 1‐octanol layer sample by a factor of Y‐fold with IS solution. Usually, X and Y correspond to 20 and 200, respectively. The concentration of the test compounds was determined using LCMSMS. The LC/MS/MS parameters of the IS were as follows: precursor m/z 329.0; product m/z 311.2; Q1 Pre Bias (v) −20.0; CE −15; Q3 Pre Bias (v) −20.0. Sample analysis was performed using a triple quadrupole mass spectrometer. Diluted samples were injected onto a Xbridge RP C18 (2.1 × 50 mm, 5 µm) HPLC column using an aqueous isocratic loading solvent (water/formic acid 1000:1) and then flushed into the mass spectrometer with a fast gradient to organic elution solvent (acetonitrile/formic acid 1000:1). Peak areas were corrected by dilution factors and incorporating IS, and the ratio of the corrected peak areas was used to calculate the results (log*D* value). The log*D* value for each compound was calculated using the following equation:

$$\text{Log}D_{1-\text{Octanol}/\text{buffer}}=\log_{10}\left(\frac{{\left[Y-\text{fold dilution of compound}\right]}_{1-\text{octanol}}\times \;Y}{{\left[X-\text{fold compound}\right]}_{\text{buffer}}\times \;X}\right)$$

#### 4.2.3 Chromatographic elog*D*_7.4_ Values

The determination of the log*D*_7.4_ values was performed by a chromatographic method as described previously [55]. Briefly, the system was calibrated by plotting the retention times of six different drugs (atenolol, metoprolol, labetalol, diltiazem, triphenylene, permethrin) versus their literature known log*D*_7.4_ values to obtain a calibration line (*R* ^2^ ≥ 0.95). Subsequently, the mean retention times of the analytes were taken to calculate their log*D*_7.4_ values with the aid of the calibration line.

#### 4.2.4 IAM Chromatography

Drug–membrane interactions were assessed and characterized by a high‐throughput HPLC method on an IAM column that consists of monolayers of phospholipids covalently bound to silica particles [56]. Particularly, the column was a Regis IAM.PC.DD2 column (100 × 4.6 mm, 10 μm, 300 Å) equipped with a guard cartridge. The column oven was set to 25 °C. Mobile phase A was 50 mM NH_4_Ac adjusted to *p*H 7.4 with ammonia, while mobile phase B was MeCN. The retention times were measured with a gradient of 0 to 95% MeCN from 0 to 6 min, which was kept at 95% until 6.5 min, and then dropped to 0% from 6.5 to 7 min, and finally kept at 0% until 9 min. The mobile phase flow rate was 1.5 mL/min. For the conversion of gradient retention times to chromatographic hydrophobicity index values referring to IAM chromatography (CHI_IAM_ values), a calibration was performed by plotting the retention times of an IAM standard solution of paracetamol, acetanilide, acetophenone, propiophenone, butyrophenone, valerophenone, and octanophenone against their literature known CHI values [56].

#### 4.2.5 Plasma Protein Binding

PPB was assessed as previously described [42]. CHIRALPAK HSA 50 × 3 mm, 5 μm column with the literature known %PPB values (converted into log *K* values) of the following drugs: warfarin, ketoprofen, budesonide, nizatidine, indomethacin, acetylsalicylic acid, carbamazepine, piroxicam, nicardipine, and cimetidine. Samples were dissolved in MeCN/DMSO 9:1 to achieve a final concentration of 0.5 mg/mL. The mobile phase A was 50 mM NH_4_Ac adjusted to pH 7.4 with ammonia, while mobile phase B was *i*PrOH. The flow rate was set to 1.0 mL/min, the UV detector was set to 254 nm, and the column temperature was kept at 30 °C. After injecting 3 μL of the sample, a linear gradient from 100% A to 30% *i*PrOH in 5.4 min was applied. From 5.4 to 18 min, 30% *i*PrOH was kept, followed by switching back to 100% A in 1.0 min and a re‐equilibration time of 6 min. For enantiomeric mixtures resolved on the chiral stationary phase, the mean retention time of the enantiomeric peaks was used for evaluation. With the aid of the calibration line (*R* ^2^ ≥ 0.92), the log *K* values of new substances were calculated and converted to their %PPB values.

#### 4.2.6 MST

MST measurements and data analysis were performed as previously described using the human thalidomide binding domain (hTBD) [57, 58]. In brief, a 16‐point 1:1 dilution series of the compound in DMSO was diluted 1:100 in ddH_2_O and then mixed with protein:reporter stock to final concentrations of 10 μM hTBD and 200 nM BODIPY‐uracil. The final concentration for the compounds in the assay ranged from 250 µM to 15 nM. Measurements were performed on a Monolith NT.115 with a Nano BLUE detector (NanoTemper Technologies), using 20% excitation power, MST power set to medium, and temperature control at 25 °C. The obtained normalized fluorescence (*F*_norm_) values at an MST on‐time of 20 s were baseline‐corrected to the mean of the normalized fluorescence values for the lowest concentration of ligand (Δ*F*_norm_), plotted against the compound concentration and fitted to a nonlinear four‐parameter equation using GraphPad Prism 9.

#### 4.2.7 Angiogenesis Assays

##### 4.2.7.1 Cell Culture

HUVEC cells (Lonza, Walkersville, MD) were grown in EBM‐II Endothelial Cell Growth Basal Medium (Lonza, Basel, Switzerland) plus EGM Plus Endothelial SingleQuot Kit (Lonza, Basel, Switzerland) and used until passage 8 post‐thawing. Cells were maintained in collagen coated flasks and used before passage 11. To split, the cells were detached using TryplE Express (Thermo Fisher Scientific, Waltham, MA), spun at 1200 rpm, and resuspended in full media. Cells were cultured in 5% CO_2_ and 95% air at 37 °C.

##### 4.2.7.2 Endothelial Cell Tube Formation Assay

The endothelial cell tube formation assay was conducted to test the anti‐angiogenic effects of the compounds. ECMatrix (60 μL/well) from in vitro angiogenesis assay kits (Millipore Sigma, Rockville, MD) was plated in a 96‐well plate and incubated at 37 °C for 30 min. HUVECs were split and plated atop the basement membrane at a density of 35,000 cells/well. The wells were treated with 0.1% DMSO (vehicle control), 10 μM Gü1215 (positive control), or test compounds. Thalidomide analogs were tested at 10 and 100 μM. The wells were incubated for 16–18 h and then imaged. Tubule formation was quantified using ImageJ software based on total mesh size. Three independent experiments were performed, with *n* = 3 replicates per experiment.

##### 4.2.7.3 Rat Aortic Ring Assay

The three‐dimensional ex vivo aortic ring model recapitulates the complexities of angiogenesis and combines the advantages of in vitro and in vivo models. The anti‐angiogenic effects of the test compounds were evaluated in the rat aortic ring assay as previously described [34, 59]. Briefly, 96‐well tissue culture plates were covered with 60 μL of Matrigel (Corning: #354234) and allowed to set for 1 h at room temperature. Six‐ to 8‐week‐old male Sprague Dawley rats were euthanized, and the descending aortas were dissected. Following excision of fibroadipose tissue, the aortic sections were cut into 1‐mm cross‐sections, placed on Matrigel‐coated wells, and layered with additional Matrigel (60 μL). These were then allowed to set, after which the cross‐sectional rings were covered with endothelial cell growth media (EGM‐II, Lonza, Walkersville, MD) and incubated under 5% CO_2_ at 37 °C overnight. EGM‐II consists of endothelial cell basal medium (EBM‐II) and endothelial cell growth factors. The next day, rings were treated with either the vehicle control (0.5% DMSO), known angiogenesis inhibitors as the positive controls (30 μM carboxyamidotriazole (CAI) or 50 μM TNP‐470), or the test compounds at a range of concentrations. Rings were incubated for 4 days and then imaged on day 5 using an EVOS scope. This was independently replicated three times using aortas from three to four different rats. The area of angiogenic sprouting, reported in square pixels, was quantified using Adobe Photoshop. Data was presented as percent growth based on the negative control (vehicle), which was normalized to 100% growth.

### 4.3 Molecular Docking

Molecular docking of pomalidomide analogs **7**, **13**, **20**, and **24** to cereblon was performed using Schrödinger Release 2024‐3 (Schrödinger, LLC, New York, NY, USA, 2024). First, crystal structure of cereblon in complex with thalidomide (PDB entry: 7BQU) was prepared using Protein Preparation Wizard with the default settings: Bond orders were assigned using CCD database, missing hydrogens were added, termini were capped, missing side chains were modeled with Prime, and het protonation states (pH 7.0 ± 2.0) were modeled with Epik [60]. Sal‐like protein 4 and water molecules were deleted prior to receptor grid calculation for the ligand‐binding site. Compound structures were optimized using LigPrep module and ionized with Epik at pH = 7.4 using OPLS4 force field. Ligands were then docked using the Glide XP protocol as implemented in Schrödinger Release 2024‐3 (Glide, Schrödinger, LLC, New York, NY, USA, 2024). The highest ranked docking pose was used for analysis and visualization in PyMOL.

## Author Contributions

**Boštjan Adamlje:** methodology, investigation, writing – original draft, visualization, writing – review and editing, data curation, conceptualization. **Tihomir Tomašič:** methodology, investigation, writing – review and editing, data curation, visualization. **Christian Steinebach:** investigation, writing – review and editing, methodology, data curation, resources. **Sebastian Ebeling:** investigation, data curation. **Alexander Herrmann:** investigation, data curation. **Marcus D. Hartmann:** investigation, methodology, supervision, writing – review and editing, resources. **Jessica L. Horner:** investigation, visualization, data curation, formal analysis. **Kinjal Bhadresha:** investigation, visualization, data curation, formal analysis. **Cindy H. Chau:** investigation, visualization, formal analysis, data curation, writing – review and editing. **William D. Figg:** methodology, resources, supervision. **Izidor Sosič:** conceptualization, writing – review and editing, project administration, resources, funding acquisition. **Andrej Emanuel Cotman:** writing – review and editing, project administration, resources, funding acquisition, conceptualization, writing – original draft, methodology, supervision, visualization, data curation.

## Funding

This study was supported by Javna Agencija za Raziskovalno Dejavnost RS (P1‐0208, J1‐50023, J1‐50037, SN‐ZRD/22‐27/510), Deutsche Forschungsgemeinschaft (530691087, 552374678), and the National Institutes of Health (ZIA SC 006538).

## Conflicts of Interest

The authors declare no conflicts of interest.

## Supporting information

Supplementary Material

## Data Availability

The data that supports the findings of this study are available in the supplementary material of this article.
