# Supplementary material for: Fluorinated Aminopiperidones as Non‐Glutarimide Thalidomide Analogs: Stereodivergent Synthesis and Validation
Source: ChemMedChem. 2026 Jul 7;21(13):e70360. doi: 10.1002/cmdc.70360 (PMC13342437; doi:10.1002/cmdc.70360)
Supplement: Supplementary file 1 — Supplementary Material [file CMDC-21-e70360-s001.pdf]

Fluorinated Aminopiperidones as Non-Glutarimide Thalidomide Analogs: Stereodivergent Synthesis and Validation

Boštjan Adamlje,[a] Tihomir Tomašič,[a] Christian Steinebach,[b] Sebastian Ebeling,[c,d] Alexander Herrmann,[c,d] Marcus D. Hartmann,[c,d] Jessica L. Horner,[e] Kinjal Bhadresha,[e] Cindy J. Chau,[e] William D. Figg,[e] Izidor Sosič,[a],*, Andrej Emanuel Cotman[a],*

[a] B. Adamlje, T. Tomašič, I. Sosič, A. E. Cotman
Faculty of Pharmacy, University of Ljubljana
SI-1000 Ljubljana, Slovenia
E-mail: izidor.sosic@ffa.uni-lj.si, Andrej.emanuel.cotman@ffa.uni-lj.si

[b] C. Steinebach
Institute of Pharmacy, Pharmaceutical/Medicinal Chemistry, University of Greifswald
D-17489 Greifswald, Germany

[c] S. Ebeling, A. Herrmann, M. Hartmann
Max Planck Institute for Biology Tübingen
D-72076 Tübingen, Germany

[d] S. Ebeling, A. Herrmann, M. D. Hartmann
Interfaculty Institute of Biochemistry, University of Tübingen
D-72076 Tübingen, Germany

[e] J. L. Horner K. Bhadresha, C. J. Chau, W. D. Figg
Molecular Pharmacology Section, Genitourinary Malignancies Branch, Center for Cancer Research, National Cancer Institute, NIH
Bethesda, MD, 20892, USA

Contents

1D NMR spectra 2

2D NMR spectra 31

HPLC traces 61

Docking (Figure S1) 77

Angiogenesis assays (Figure S2–S7, Table S1–S2) 78

1D NMR spectra

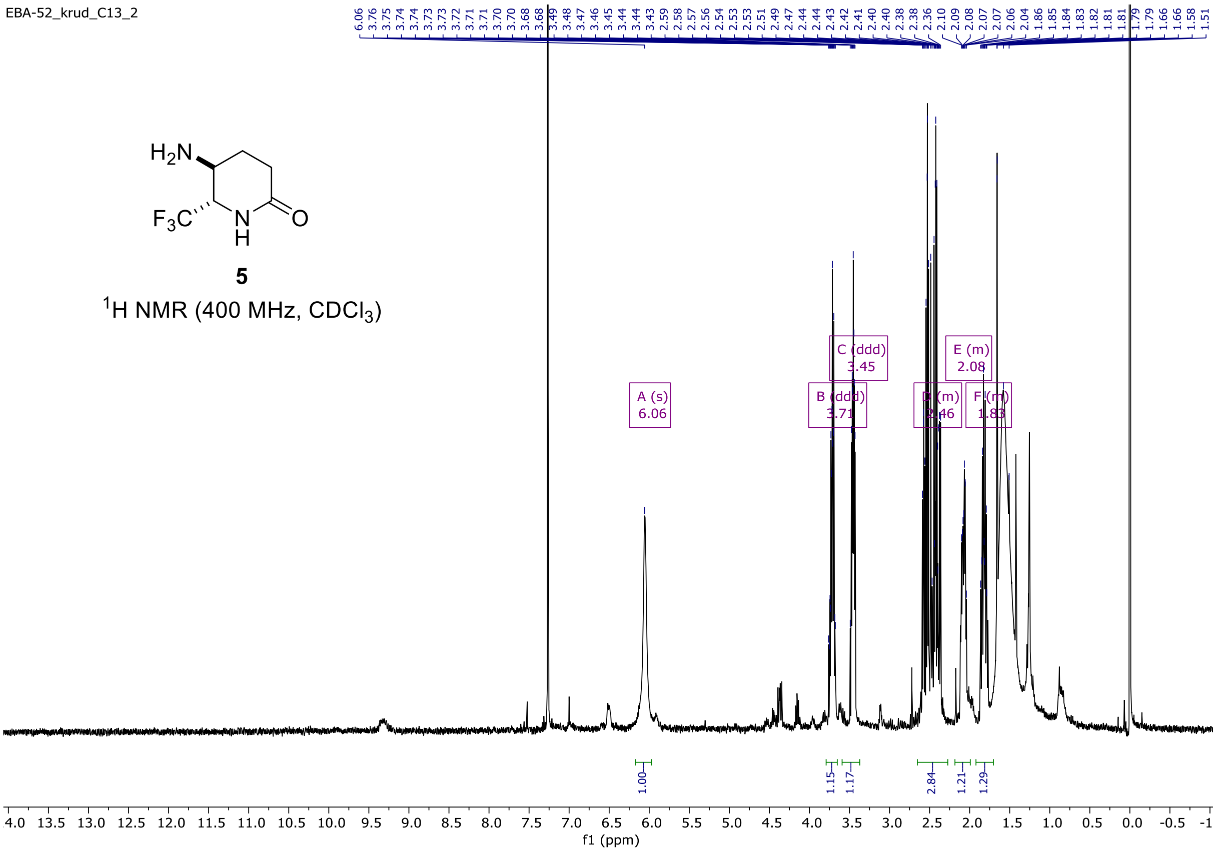

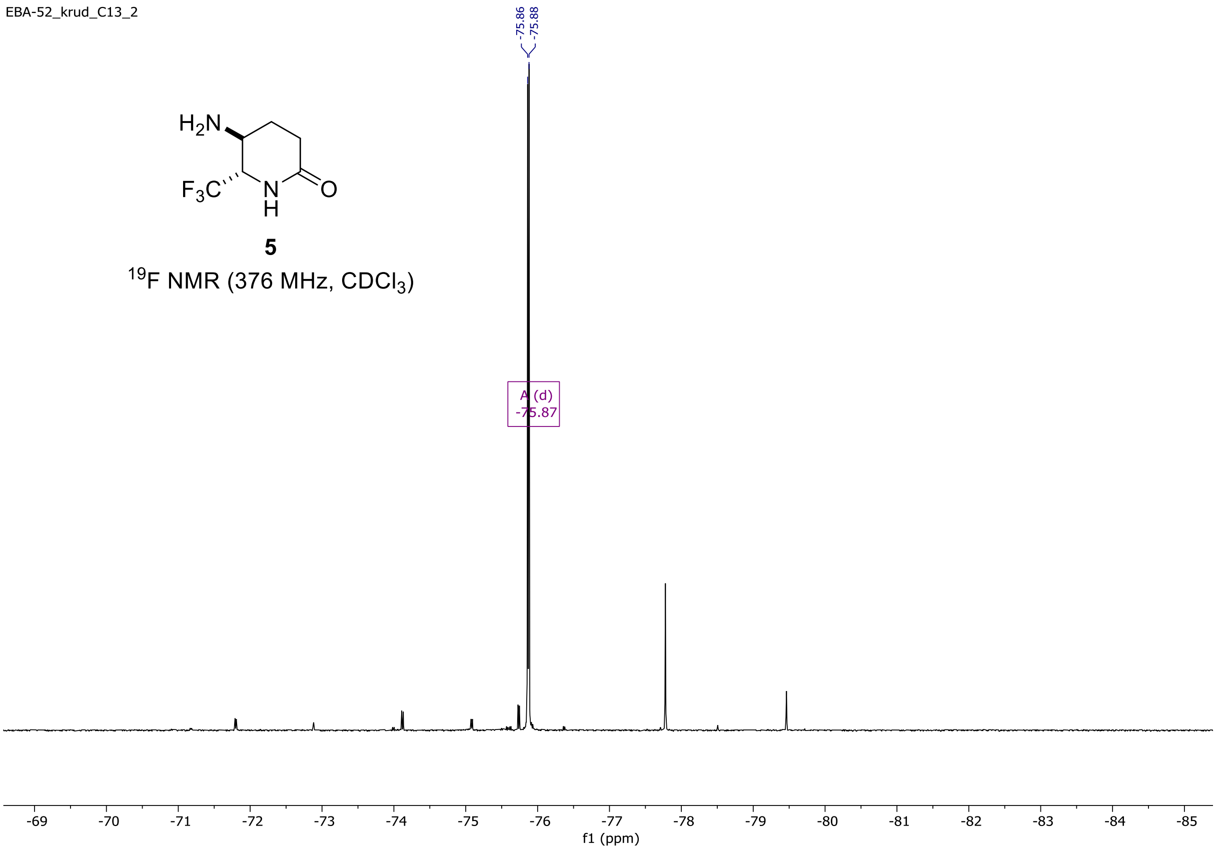

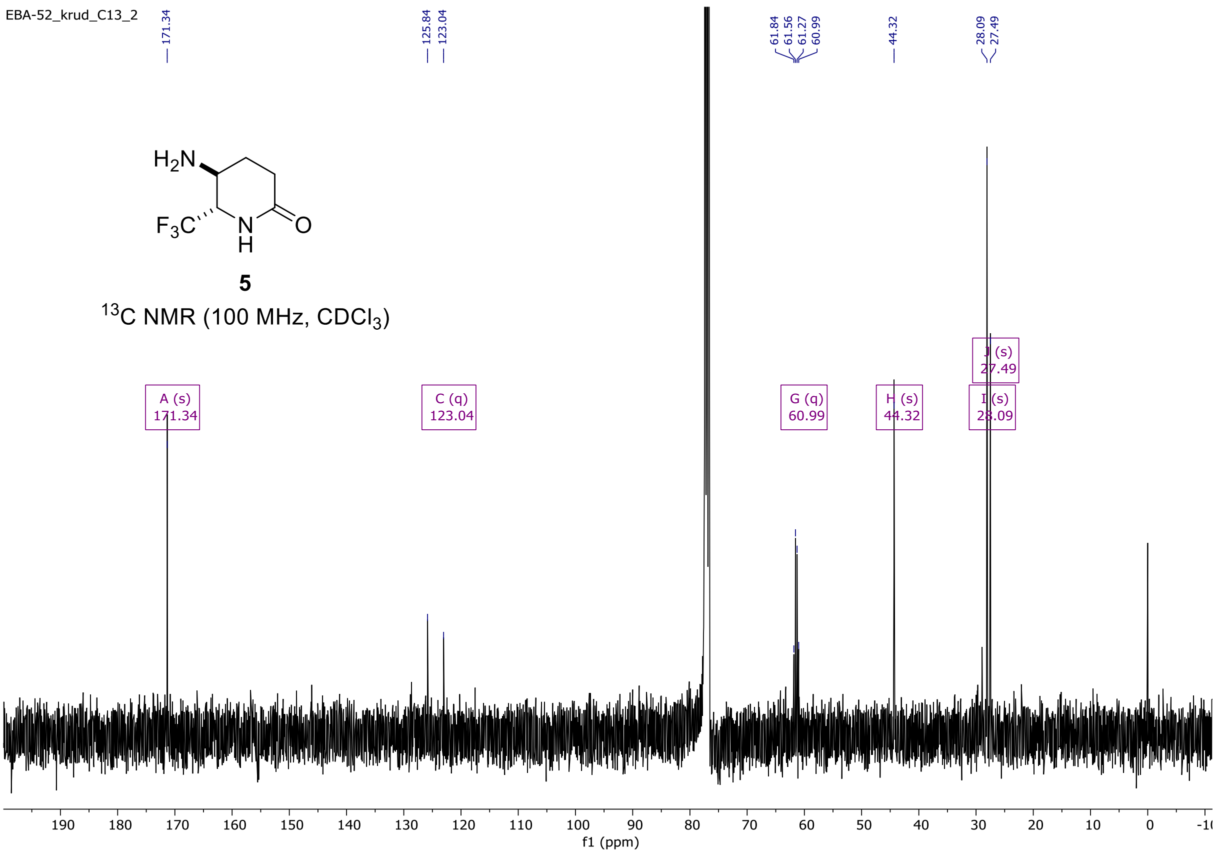
EBA-52_krud_C13_2
5
^{13}C NMR (100 MHz, CDCl$_3$)
A (s) 171.34
C (q) 123.04
G (q) 60.99
H (s) 44.32
I (s) 28.09
J (s) 27.49
f1 (ppm)

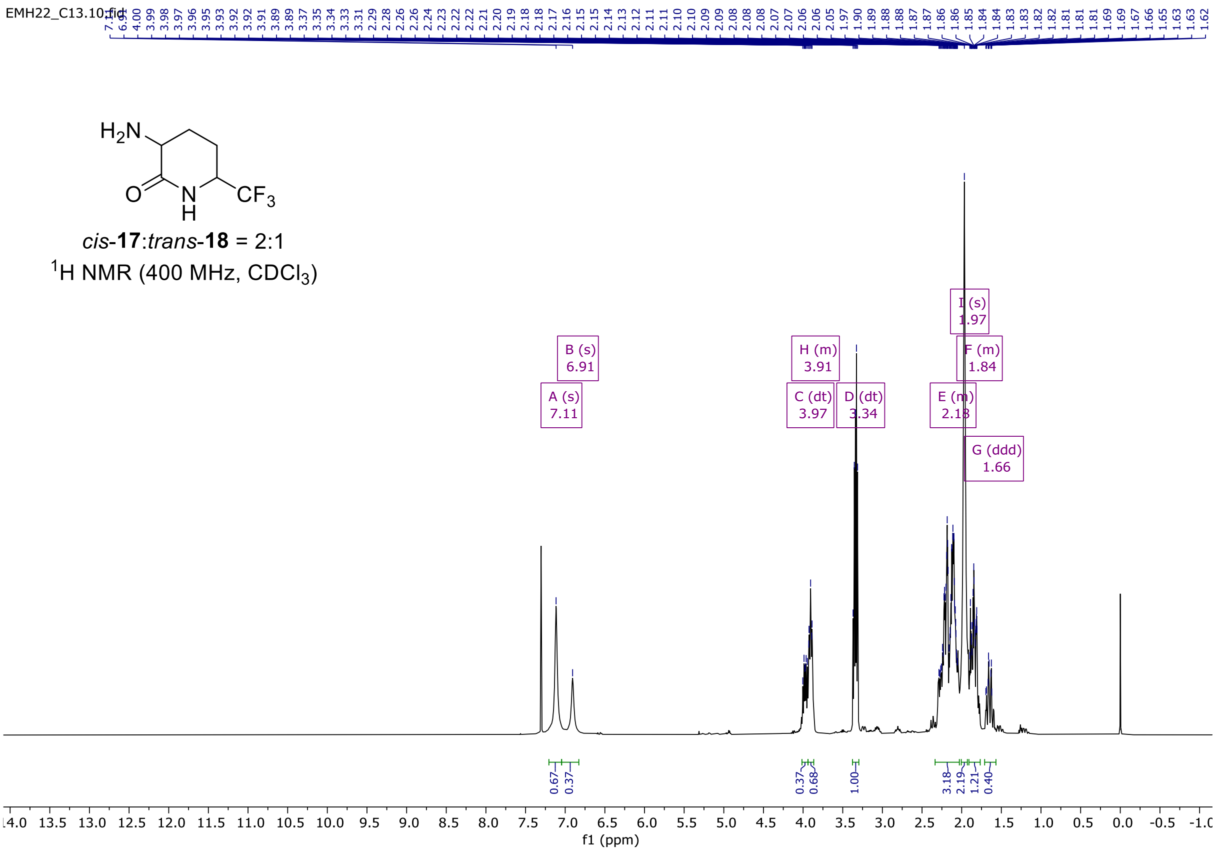
EMH22_C13.10.fid
cis-17:trans-18 = 2:1
^{1}H NMR (400 MHz, CDCl$_3$)
A (s) 7.11
B (s) 6.91
C (dt) 3.97
H (m) 3.91
D (dt) 3.34
E (m) 2.18
I (s) 1.97
F (m) 1.84
G (ddd) 1.66
f1 (ppm)

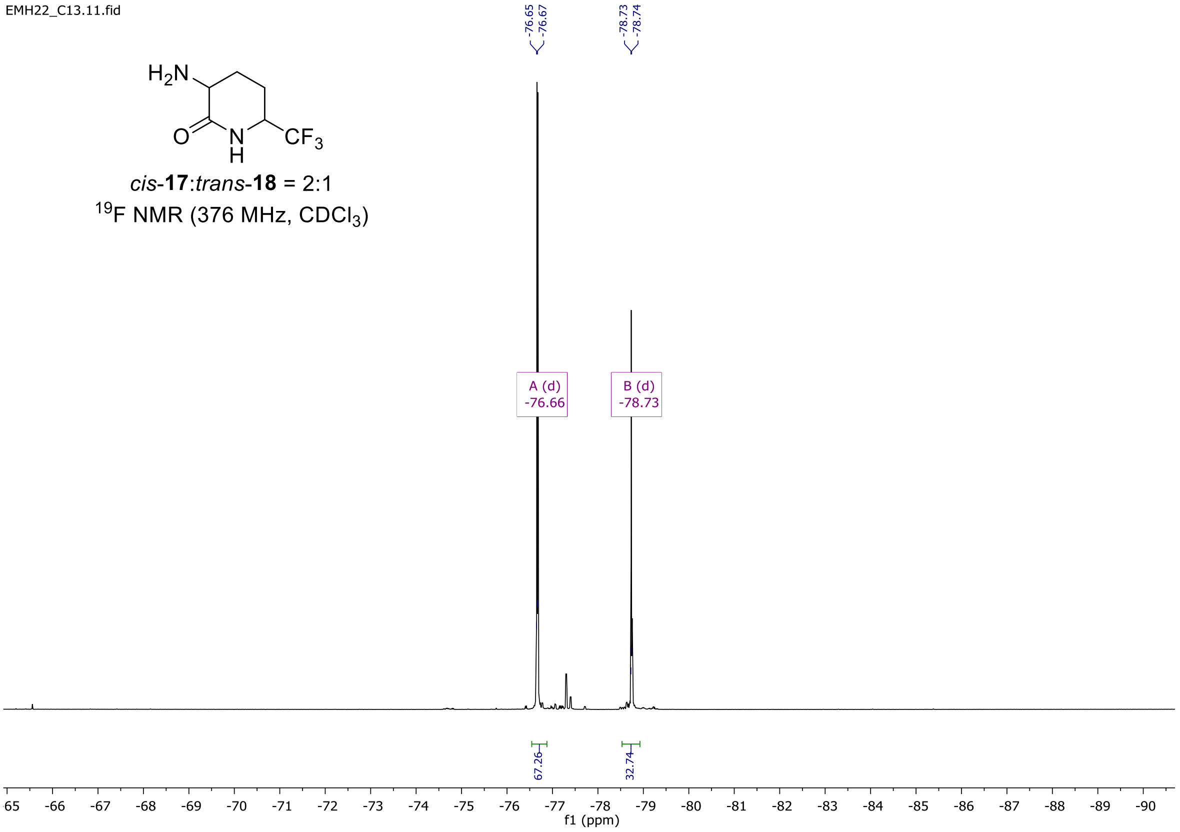
EMH22_C13.11.fid
H2N
O
N
H
CF3
cis-17:trans-18 = 2:1
19F NMR (376 MHz, CDCl3)
-76.65
-76.67
-78.73
-78.74
A (d)
-76.66
B (d)
-78.73
67.26
32.74
f1 (ppm)

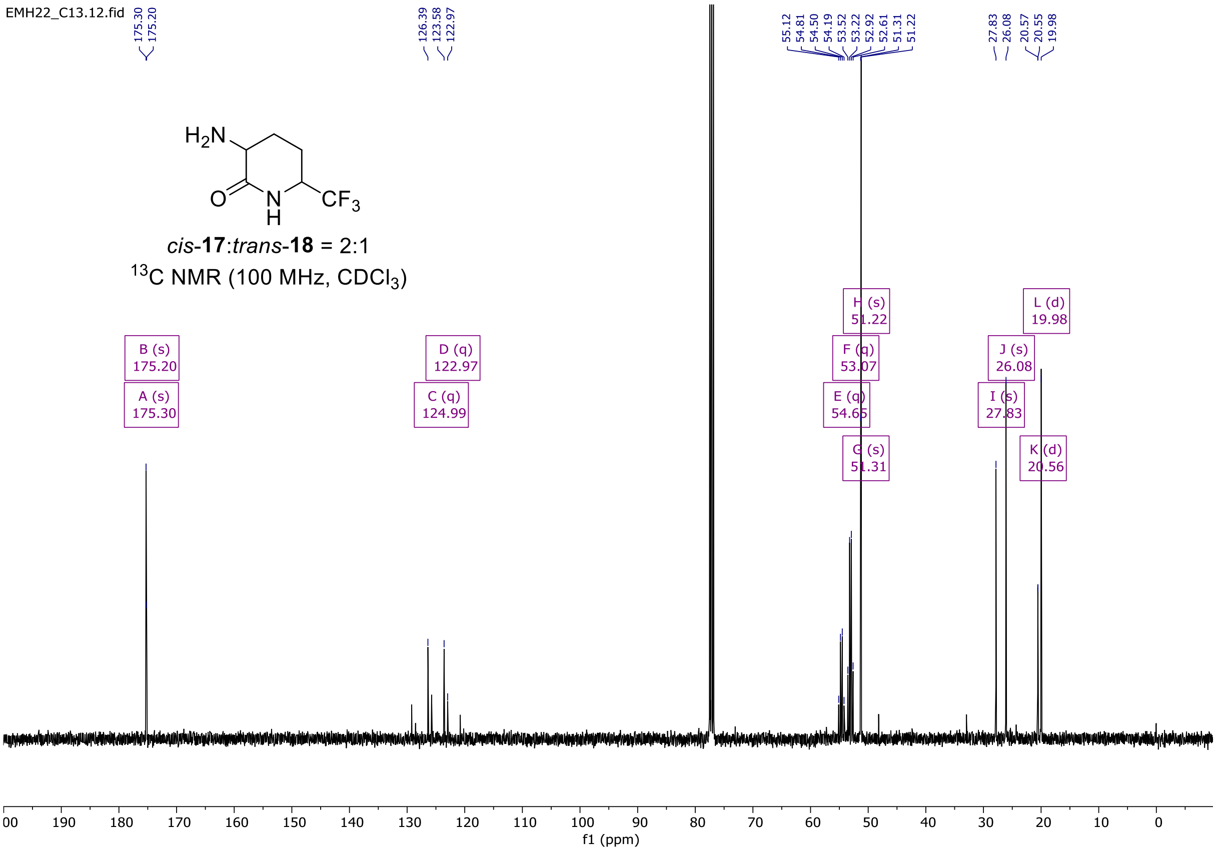
EMH22_C13.12.fid
175.30
175.20
126.39
123.58
122.97
55.12
54.81
54.50
54.19
53.52
53.22
52.92
52.61
51.31
51.22
27.83
26.08
20.57
20.55
19.98
H2N
O
N
H
CF3
cis-17:trans-18 = 2:1
13C NMR (100 MHz, CDCl3)
B (s)
175.20
A (s)
175.30
D (q)
122.97
C (q)
124.99
H (s)
51.22
F (q)
53.07
E (q)
54.65
G (s)
51.31
L (d)
19.98
J (s)
26.08
I (s)
27.83
K (d)
20.56
f1 (ppm)

EMH23_C13.10.fid

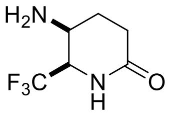

11

^{1}H NMR (400 MHz, $CDCl_3$-d_6)

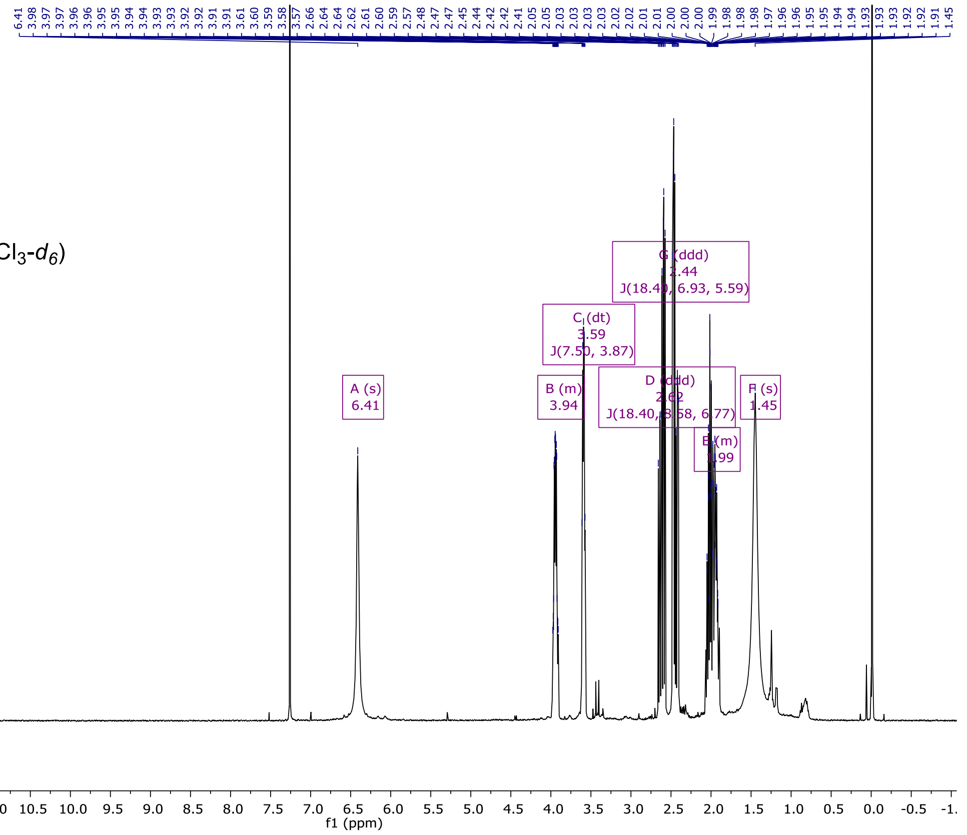

EMH23_C13.11.fid

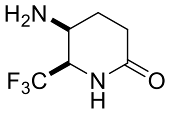

11

^{19}F NMR (376 MHz, $CDCl_3$-d_6)

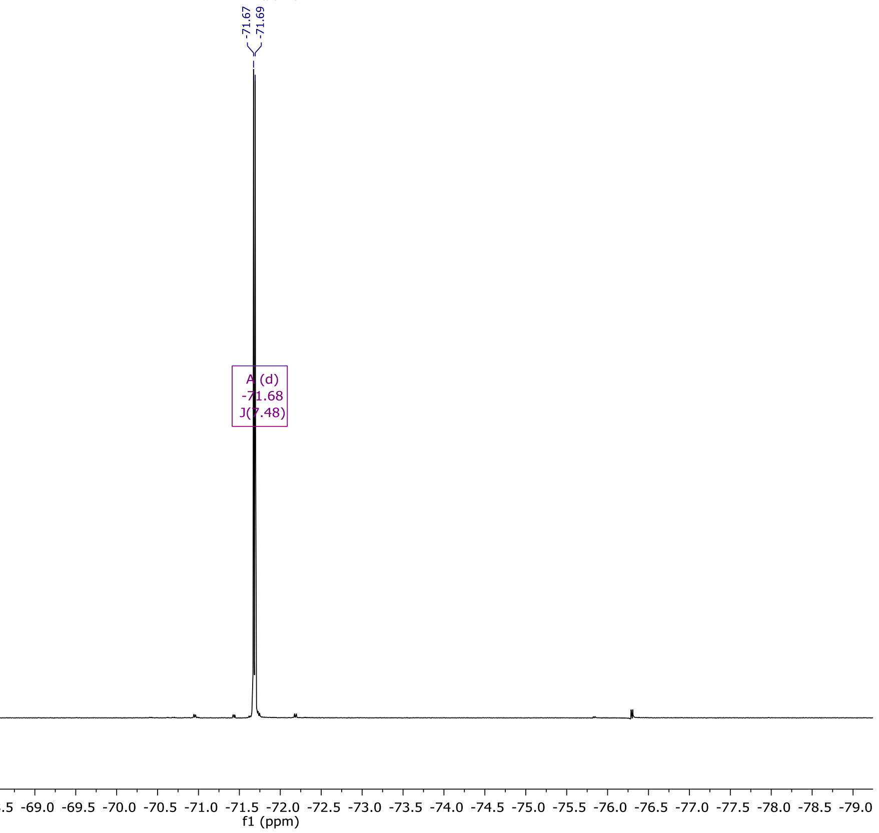

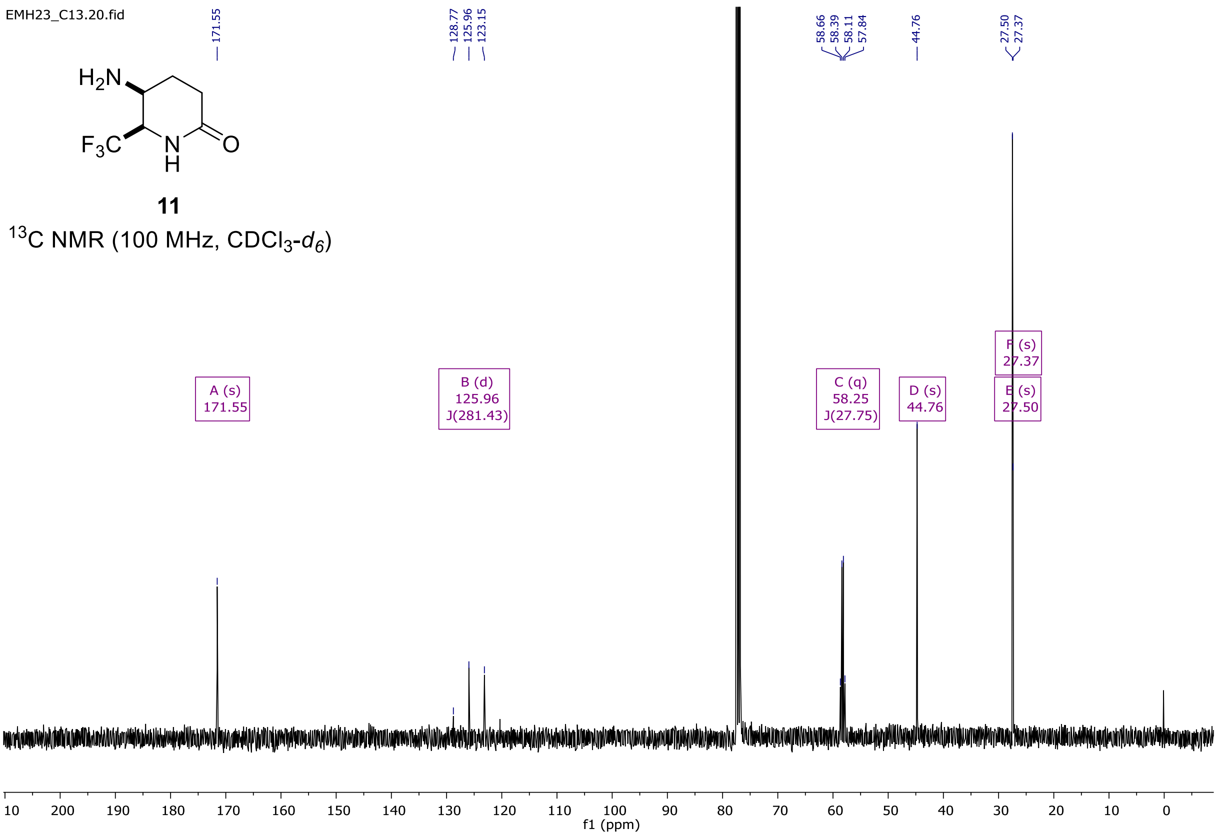
EMH23_C13.20.fid
H2N
F3C
N
H
O
11
13C NMR (100 MHz, CDCl3-d6)
171.55
128.77
125.96
123.15
58.66
58.39
58.11
57.84
44.76
27.50
27.37
A (s) 171.55
B (d) 125.96 J(281.43)
C (q) 58.25 J(27.75)
D (s) 44.76
E (s) 27.50
F (s) 27.37
f1 (ppm)

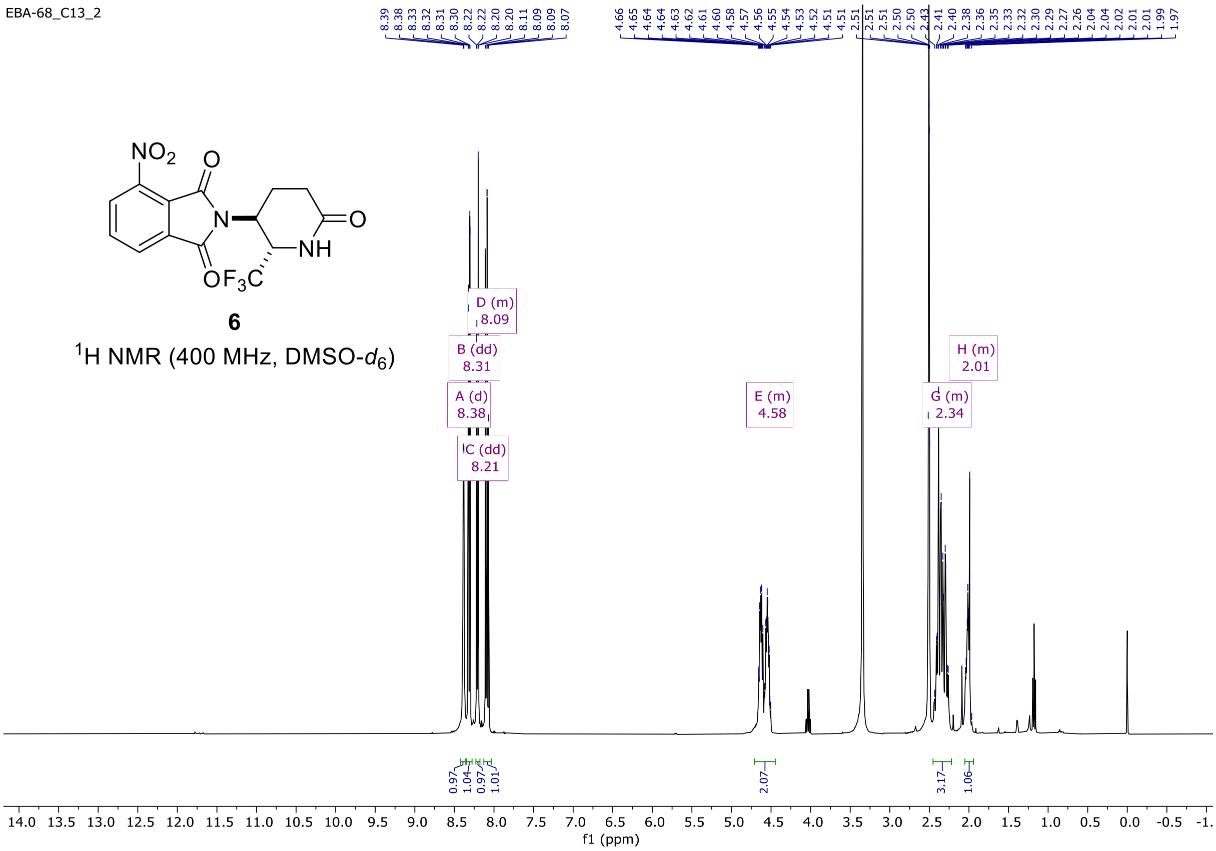
EBA-68_C13_2
NO2
O
N
O
F3C
NH
O
6
1H NMR (400 MHz, DMSO-d6)
8.39 8.38 8.33 8.32 8.31 8.30 8.22 8.22 8.20 8.20 8.11 8.09 8.09 8.07
4.66 4.65 4.64 4.63 4.62 4.61 4.60 4.58 4.57 4.56 4.55 4.54 4.53 4.52 4.51 4.51
2.51 2.51 2.51 2.50 2.50 2.42 2.41 2.40 2.38 2.36 2.35 2.33 2.32 2.30 2.29 2.27 2.26 2.04 2.02 2.02 2.01 2.01 1.99 1.97
A (d) 8.38
B (dd) 8.31
C (dd) 8.21
D (m) 8.09
E (m) 4.58
G (m) 2.34
H (m) 2.01
0.97
1.04
0.97
1.01
2.07
3.17
1.06
f1 (ppm)

EBA-68_C13_2

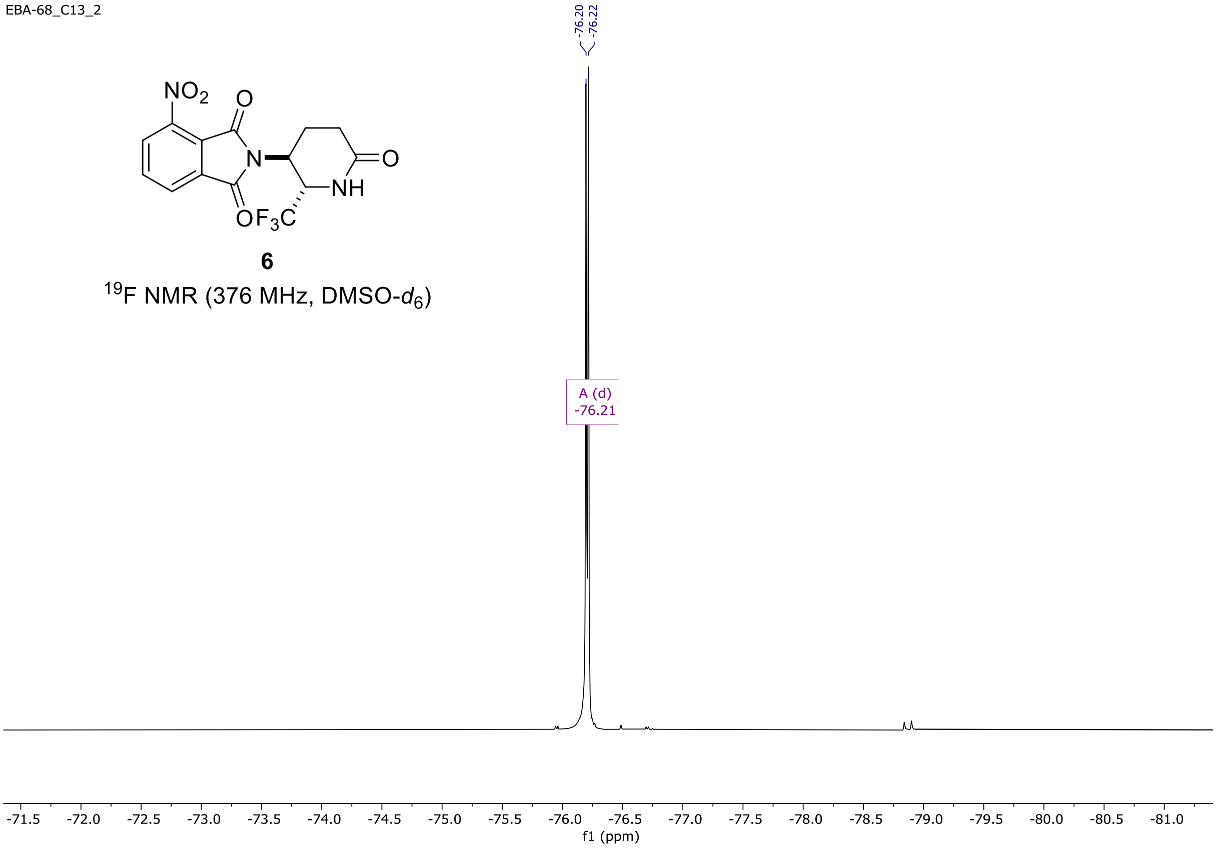

6

^{19}F NMR (376 MHz, DMSO-d_6)

EBA-68_C13_2

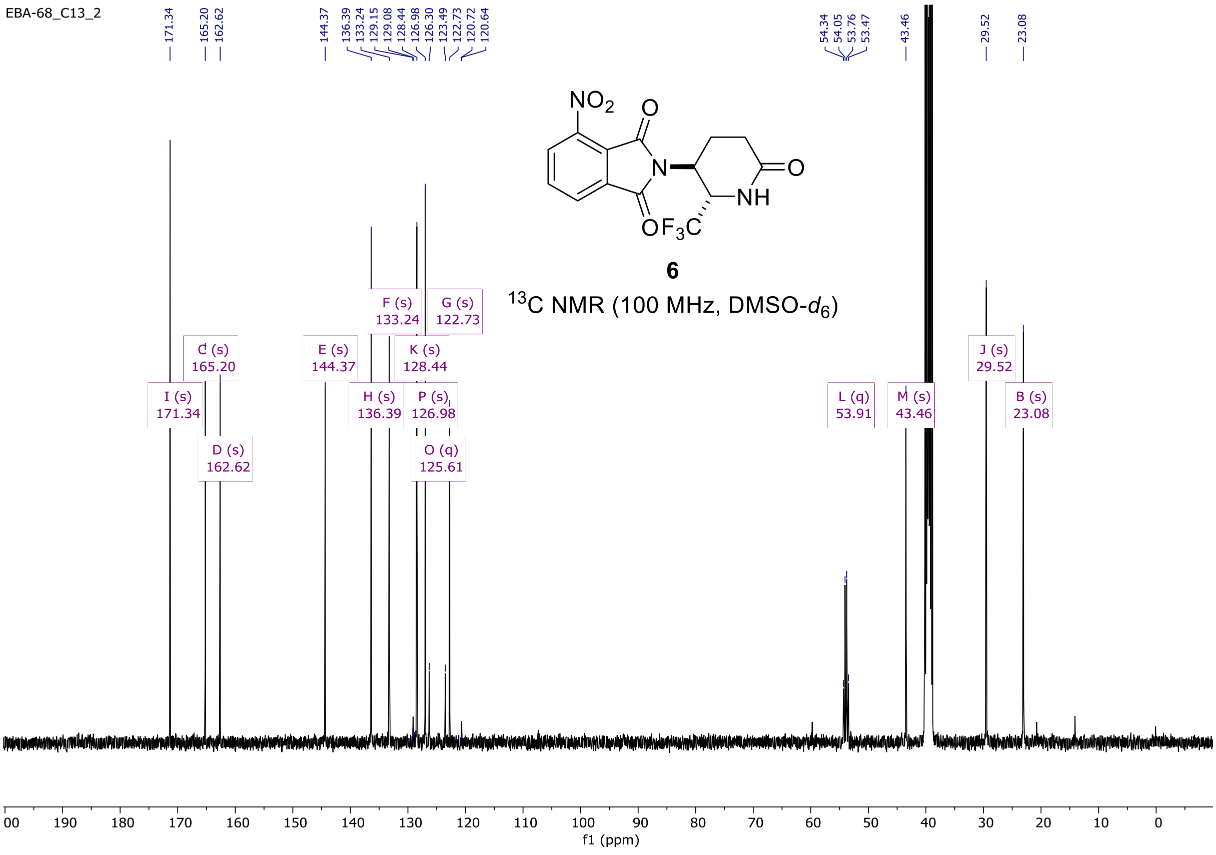

6

^{13}C NMR (100 MHz, DMSO-d_6)

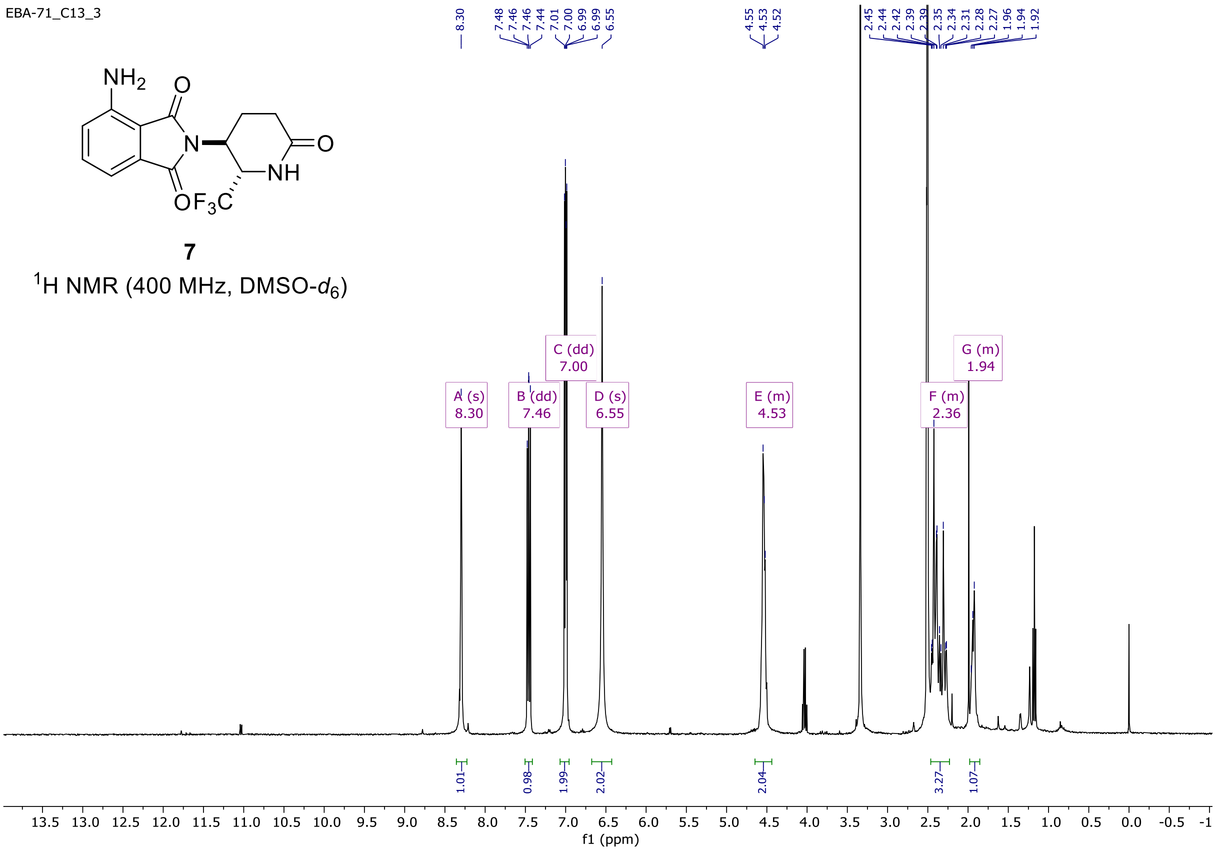
EBA-71_C13_3
7
^{1}H NMR (400 MHz, DMSO-d_6)
A (s) 8.30
B (dd) 7.46
C (dd) 7.00
D (s) 6.55
E (m) 4.53
F (m) 2.36
G (m) 1.94
f1 (ppm)

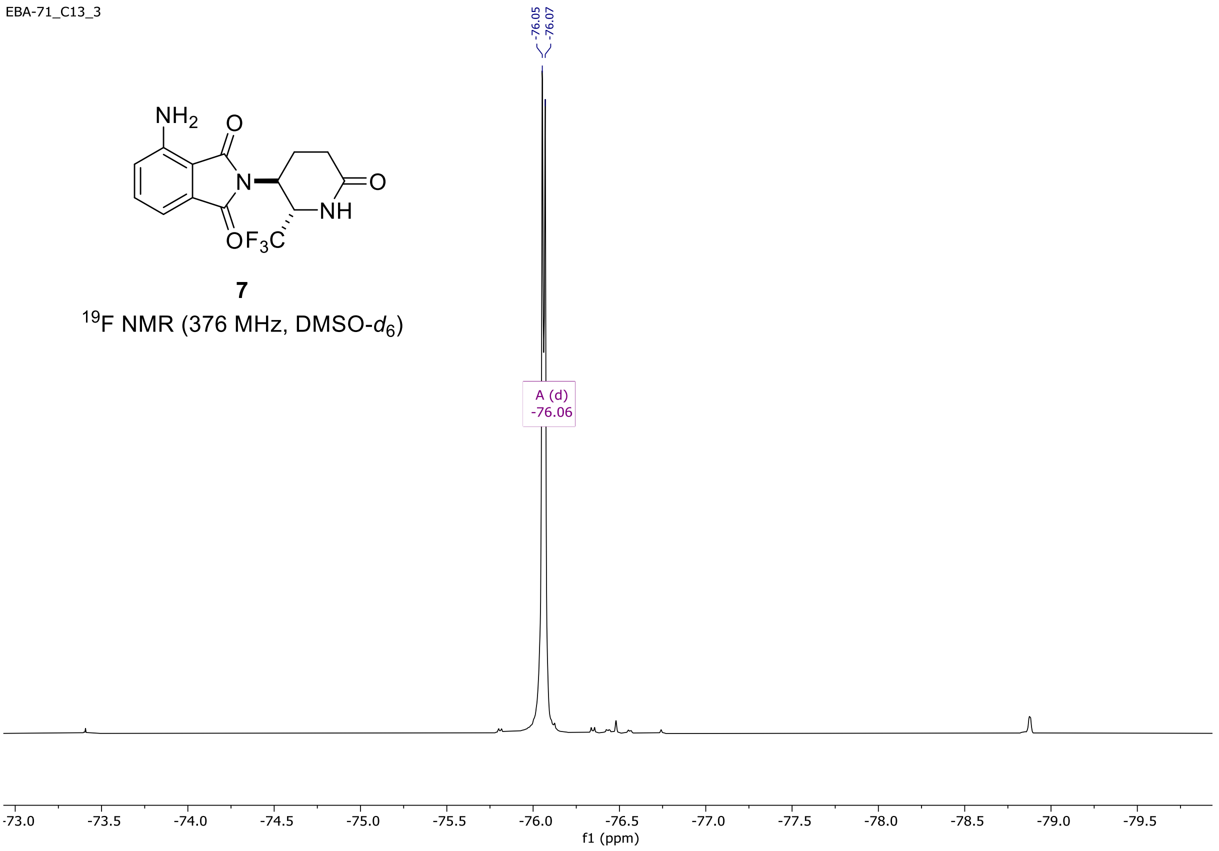
EBA-71_C13_3
7
^{19}F NMR (376 MHz, DMSO-d_6)
A (d) -76.06
f1 (ppm)

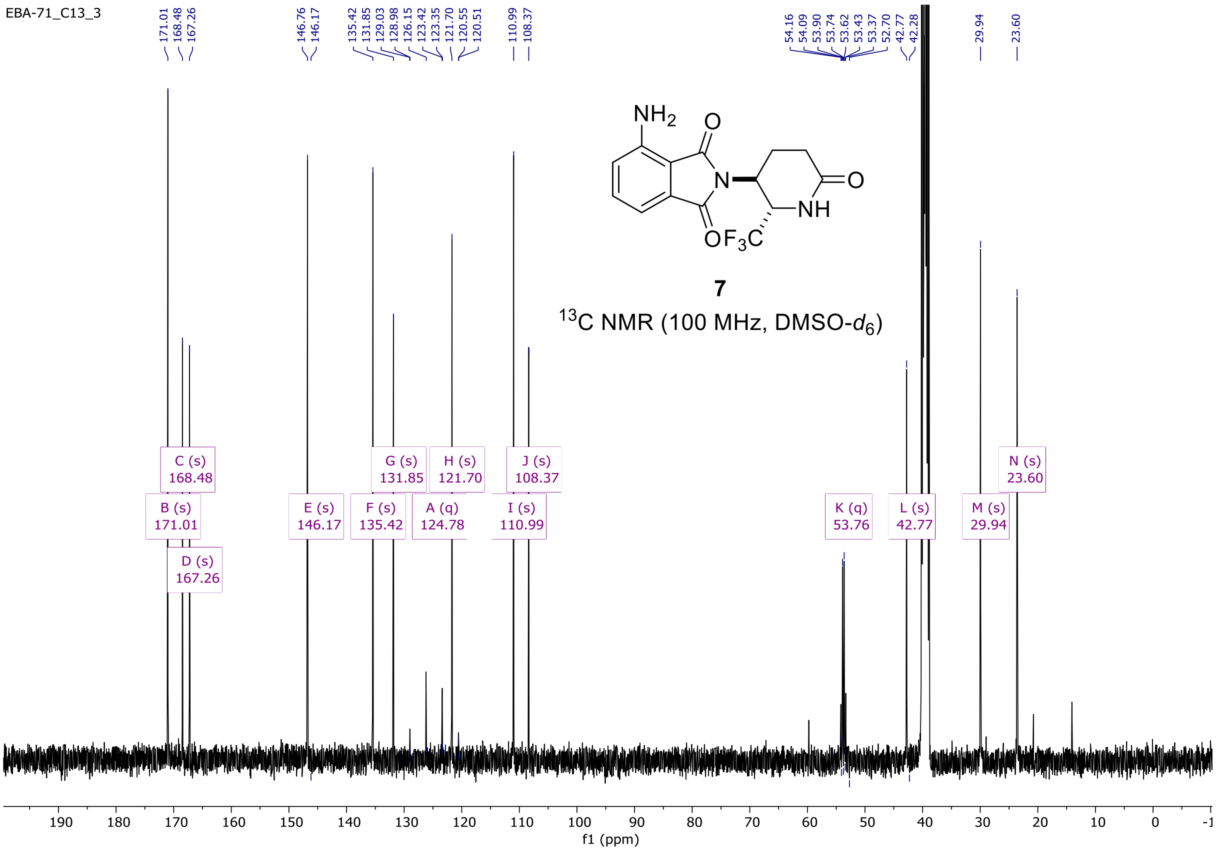
EBA-71_C13_3
7
^{13}C NMR (100 MHz, DMSO-d_6)
B (s) 171.01
C (s) 168.48
D (s) 167.26
E (s) 146.17
F (s) 135.42
G (s) 131.85
A (q) 124.78
H (s) 121.70
I (s) 110.99
J (s) 108.37
K (q) 53.76
L (s) 42.77
M (s) 29.94
N (s) 23.60
f1 (ppm)

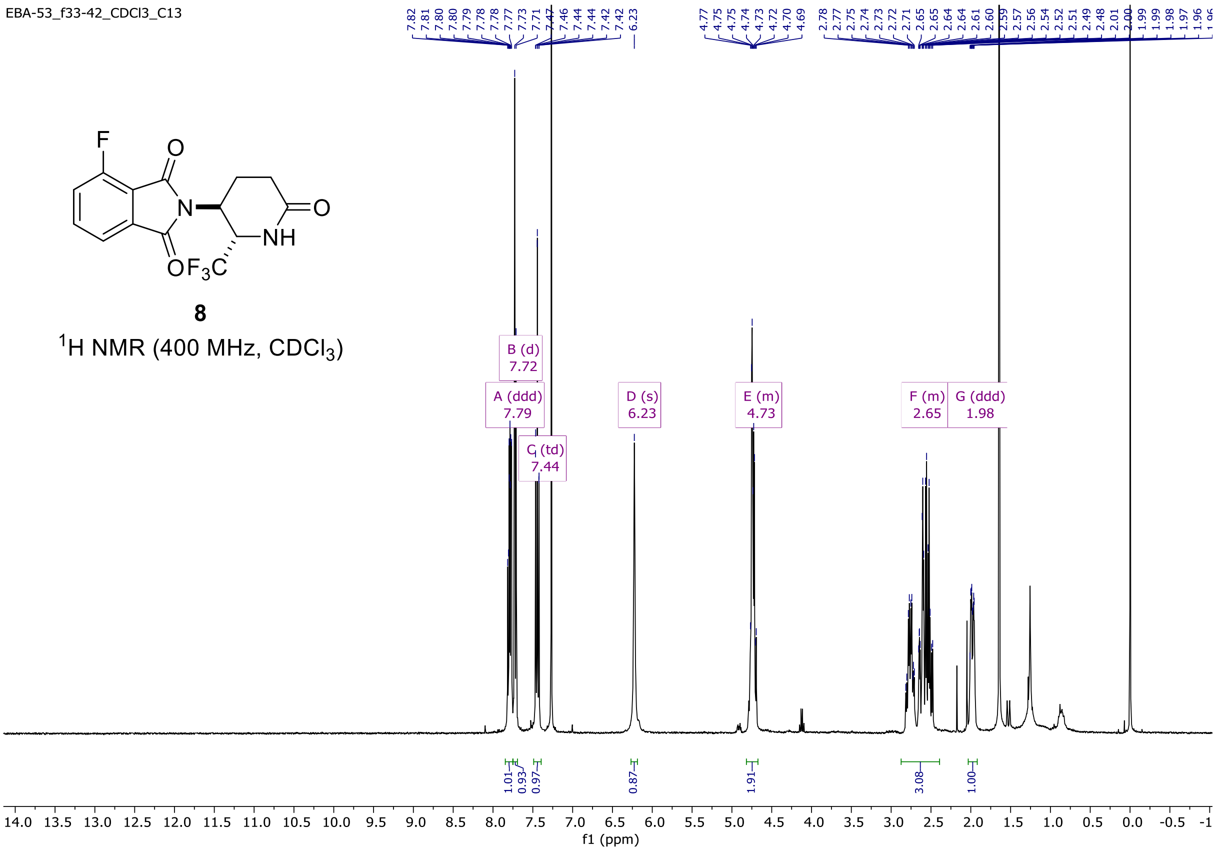
EBA-53_f33-42_CDCl3_C13
8
^{1}H NMR (400 MHz, CDCl$_3$)
B (d) 7.72
A (ddd) 7.79
C (td) 7.44
D (s) 6.23
E (m) 4.73
F (m) 2.65
G (ddd) 1.98
f1 (ppm)

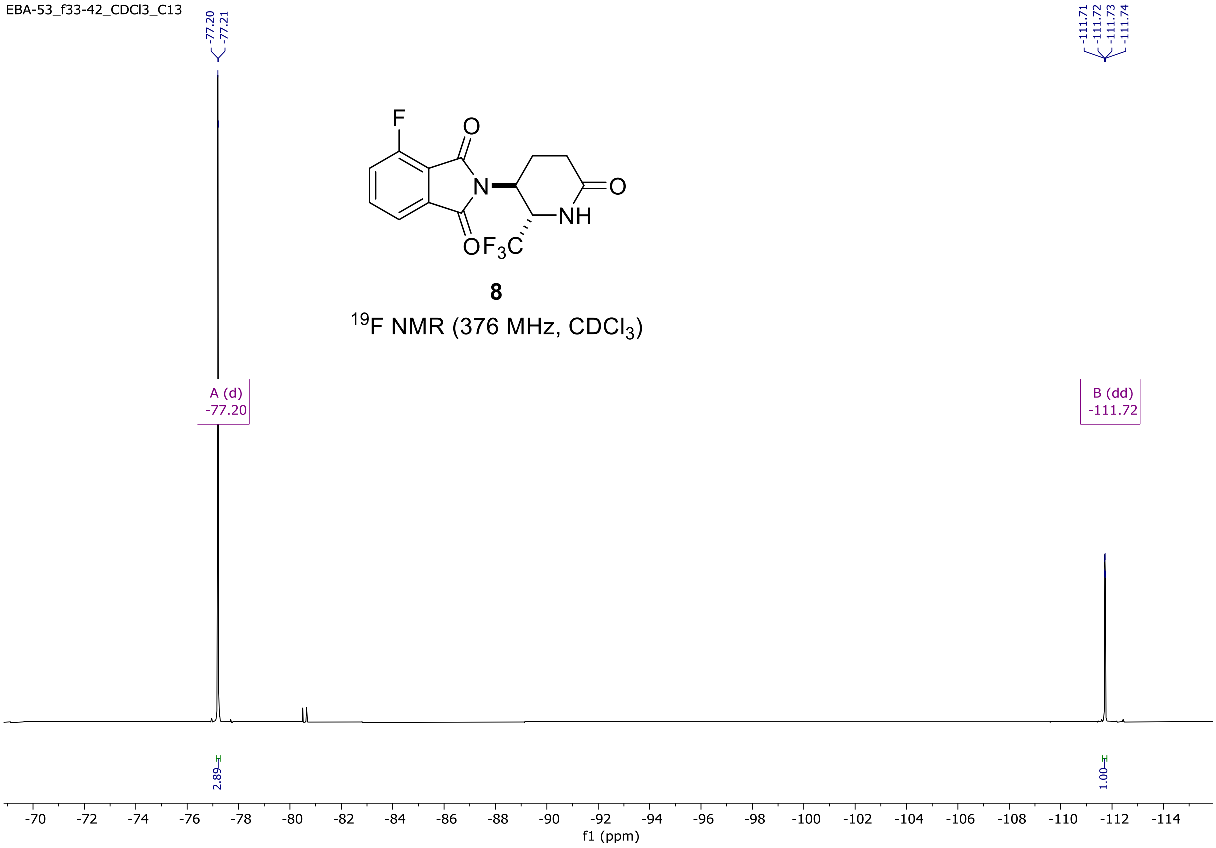
EBA-53_f33-42_CDCl3_C13
-77.20
-77.21
-111.71
-111.72
-111.73
-111.74
F
O
N
O
NH
O
F3C
8
19F NMR (376 MHz, CDCl3)
A (d)
-77.20
B (dd)
-111.72
2.89
1.00
-70 -72 -74 -76 -78 -80 -82 -84 -86 -88 -90 -92 -94 -96 -98 -100 -102 -104 -106 -108 -110 -112 -114
f1 (ppm)

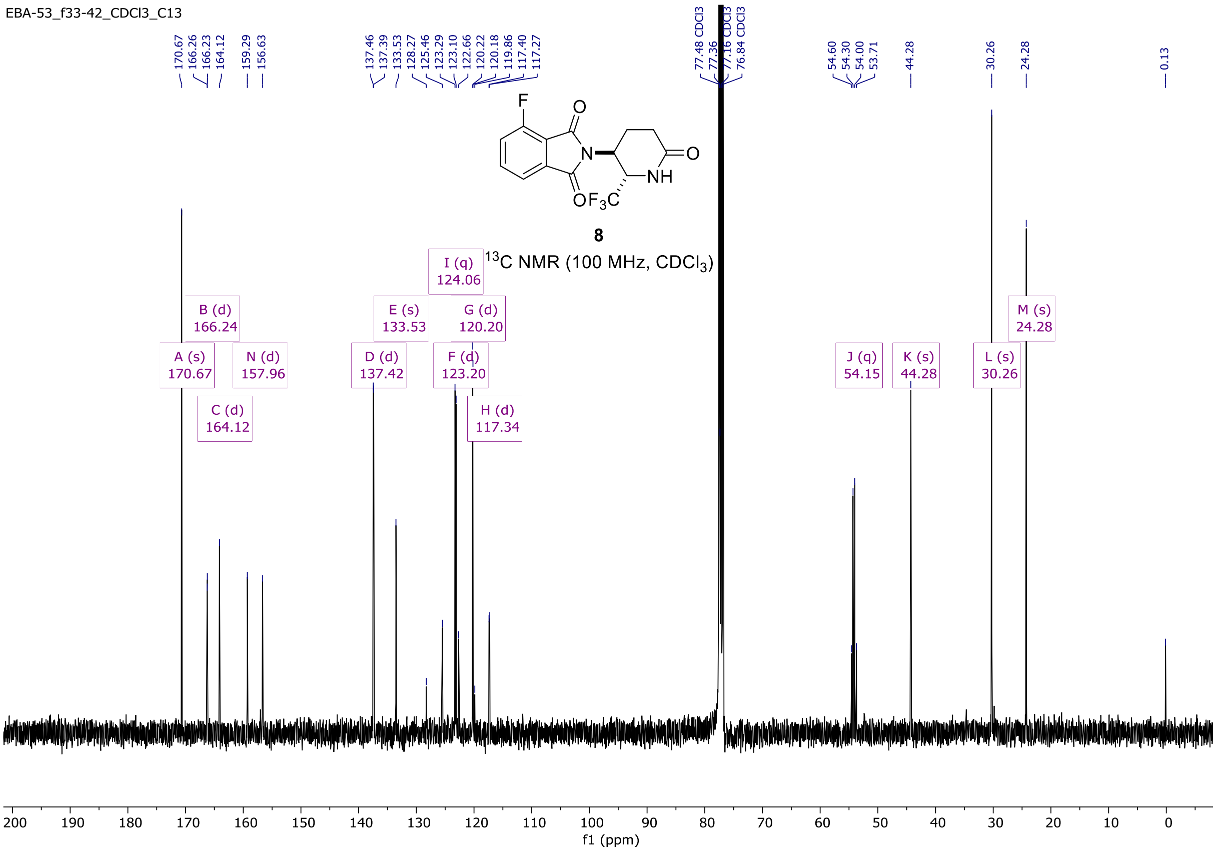
EBA-53_f33-42_CDCl3_C13
170.67
166.26
166.23
164.12
159.29
156.63
137.46
137.39
133.53
128.27
125.46
123.29
123.10
122.66
120.22
120.18
119.86
117.40
117.27
77.48 CDCl3
77.36
77.16 CDCl3
76.84 CDCl3
54.60
54.30
54.00
53.71
44.28
30.26
24.28
0.13
F
O
N
O
NH
O
F3C
8
13C NMR (100 MHz, CDCl3)
I (q)
124.06
B (d)
166.24
E (s)
133.53
G (d)
120.20
M (s)
24.28
A (s)
170.67
N (d)
157.96
D (d)
137.42
F (d)
123.20
J (q)
54.15
K (s)
44.28
L (s)
30.26
C (d)
164.12
H (d)
117.34
200 190 180 170 160 150 140 130 120 110 100 90 80 70 60 50 40 30 20 10 0
f1 (ppm)

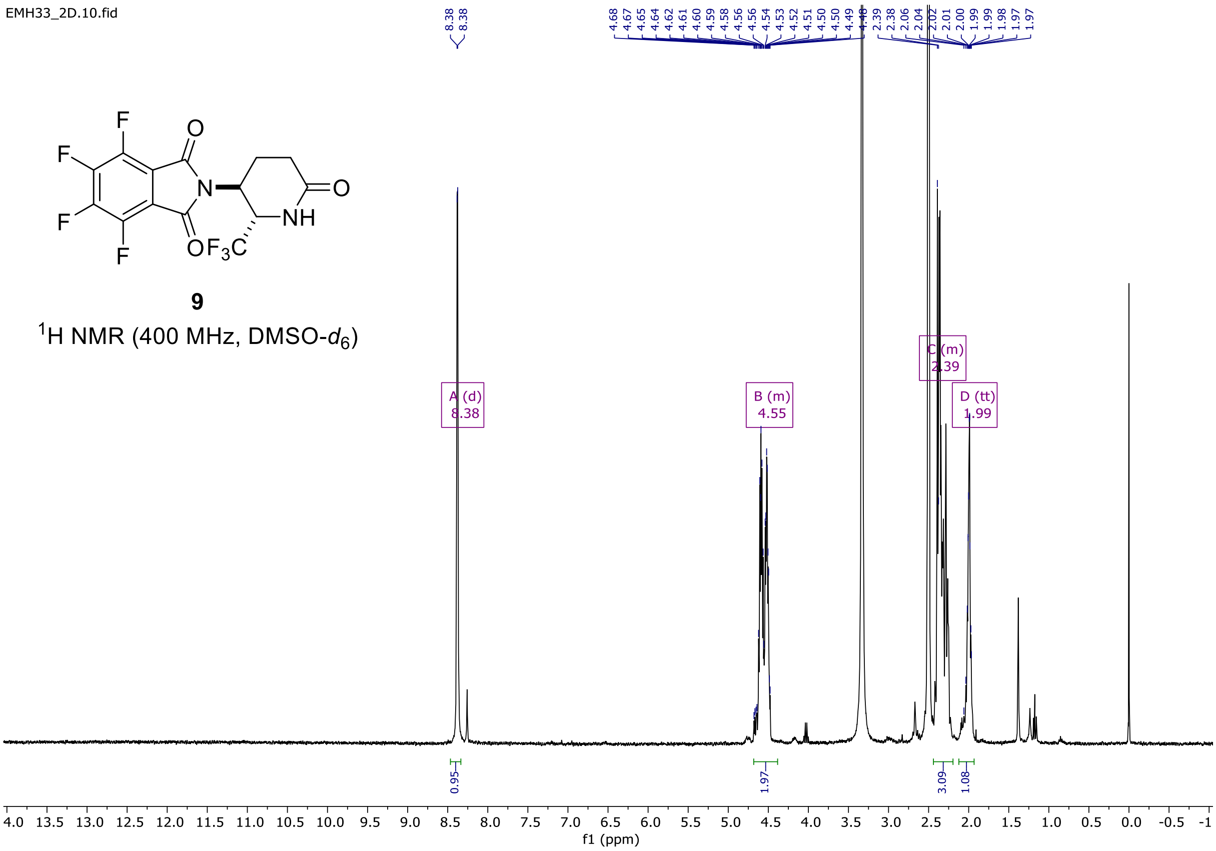
EMH33_2D.10.fid
9
^{1}H NMR (400 MHz, DMSO-d_6)
8.38
8.38
4.68
4.67
4.65
4.64
4.62
4.61
4.60
4.59
4.58
4.56
4.54
4.53
4.52
4.51
4.50
4.49
2.39
2.38
2.06
2.04
2.02
2.01
2.00
1.99
1.99
1.98
1.97
1.97
A (d) 8.38
B (m) 4.55
C (m) 2.39
D (tt) 1.99
0.95
1.97
3.09
1.08
f1 (ppm)

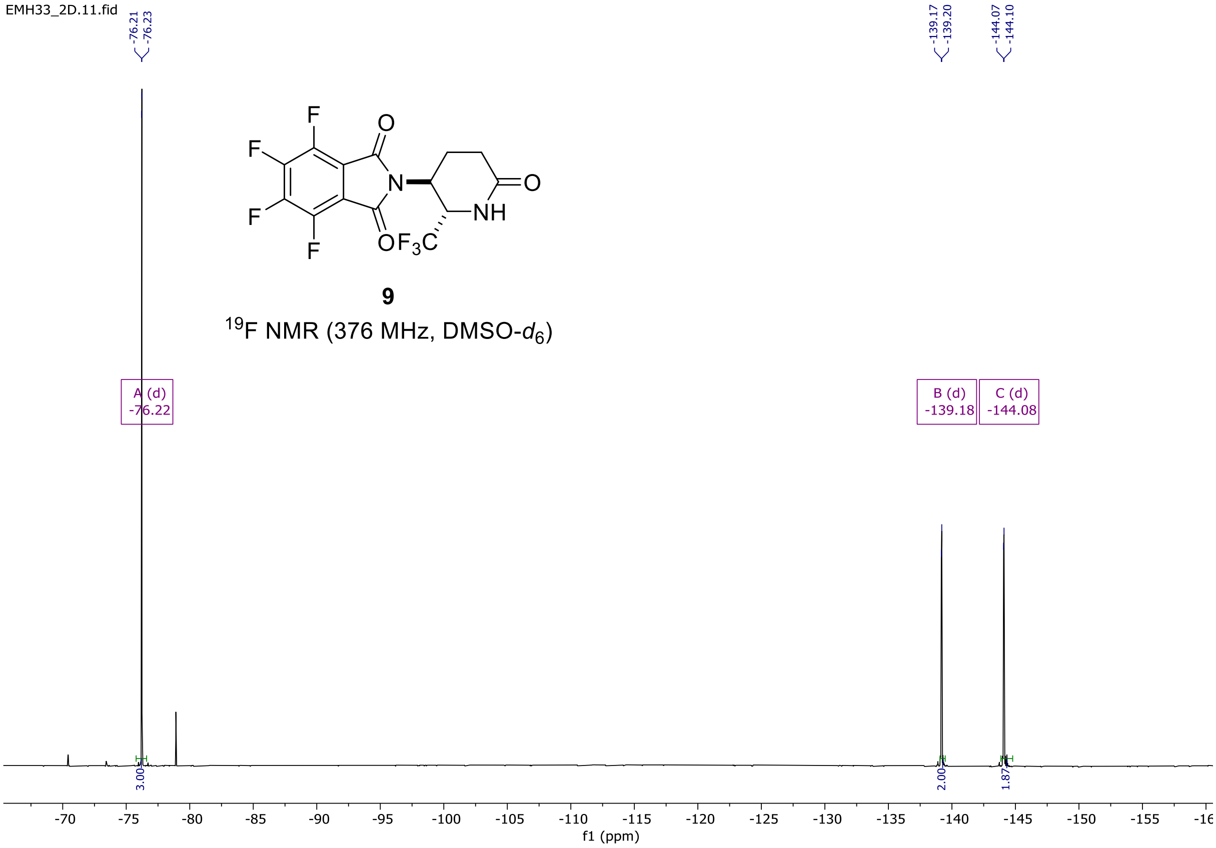
EMH33_2D.11.fid
-76.21
-76.23
-139.17
-139.20
-144.07
-144.10
9
^{19}F NMR (376 MHz, DMSO-d_6)
A (d) -76.22
B (d) -139.18
C (d) -144.08
3.00
2.00
1.87
f1 (ppm)

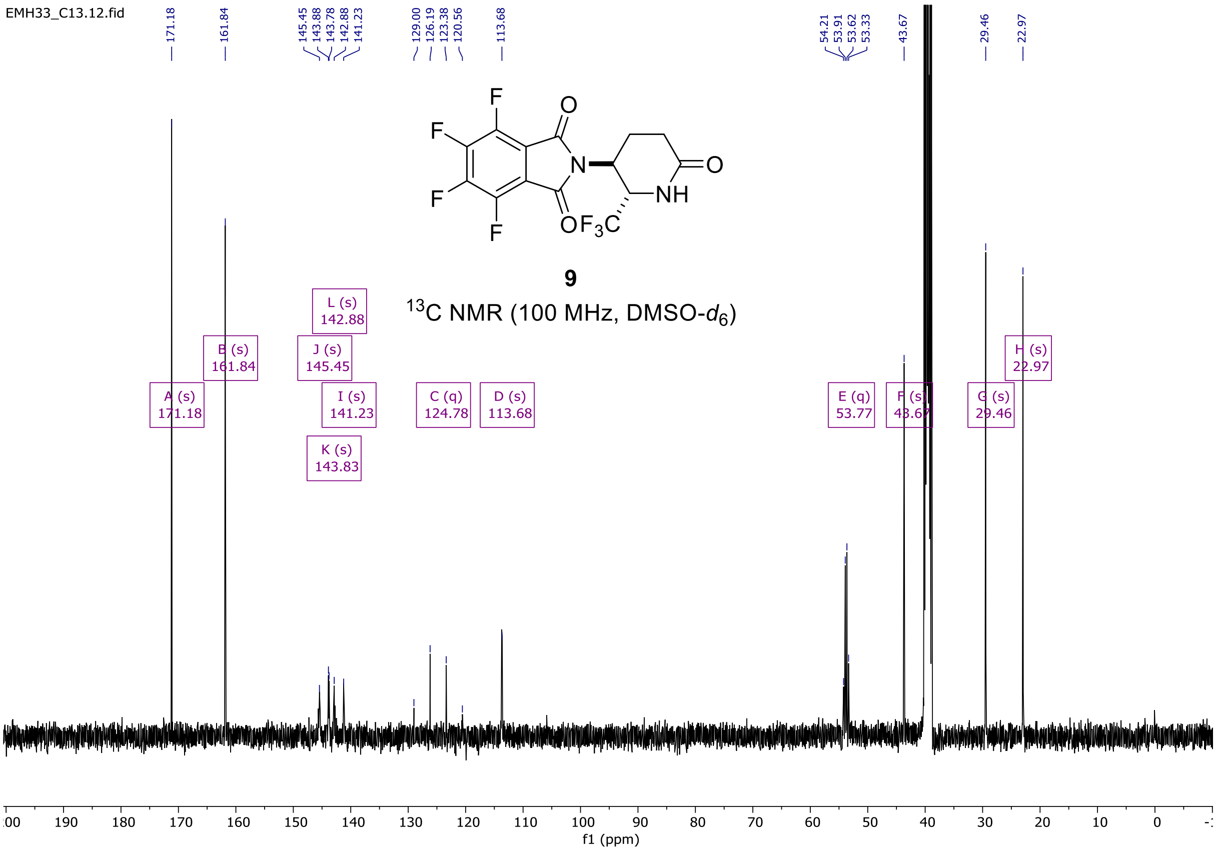
EMH33_C13.12.fid
171.18
161.84
145.45
143.88
143.78
142.88
141.23
129.00
126.19
123.38
120.56
113.68
54.21
53.91
53.62
53.33
43.67
29.46
22.97
9
13C NMR (100 MHz, DMSO-d6)
A (s) 171.18
B (s) 161.84
C (q) 124.78
D (s) 113.68
E (q) 53.77
F (s) 43.67
G (s) 29.46
H (s) 22.97
I (s) 141.23
J (s) 145.45
K (s) 143.83
L (s) 142.88
f1 (ppm)

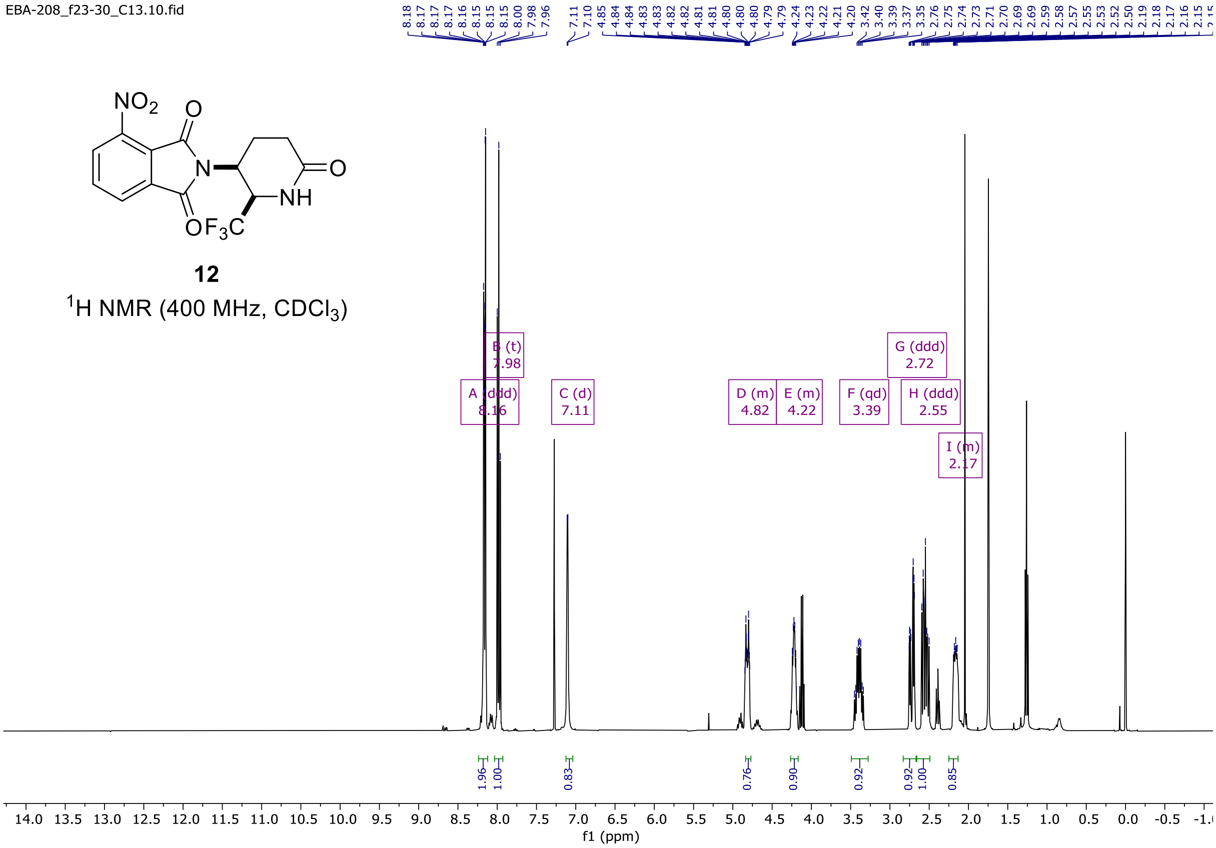
EBA-208_f23-30_C13.10.fid
12
1H NMR (400 MHz, CDCl3)
A (ddd) 8.16
B (t) 7.98
C (d) 7.11
D (m) 4.82
E (m) 4.22
F (qd) 3.39
G (ddd) 2.72
H (ddd) 2.55
I (m) 2.17
1.96
1.00
0.83
0.76
0.90
0.92
0.92
1.00
0.85
f1 (ppm)

EBA-208_f23-30_C13.11.fid

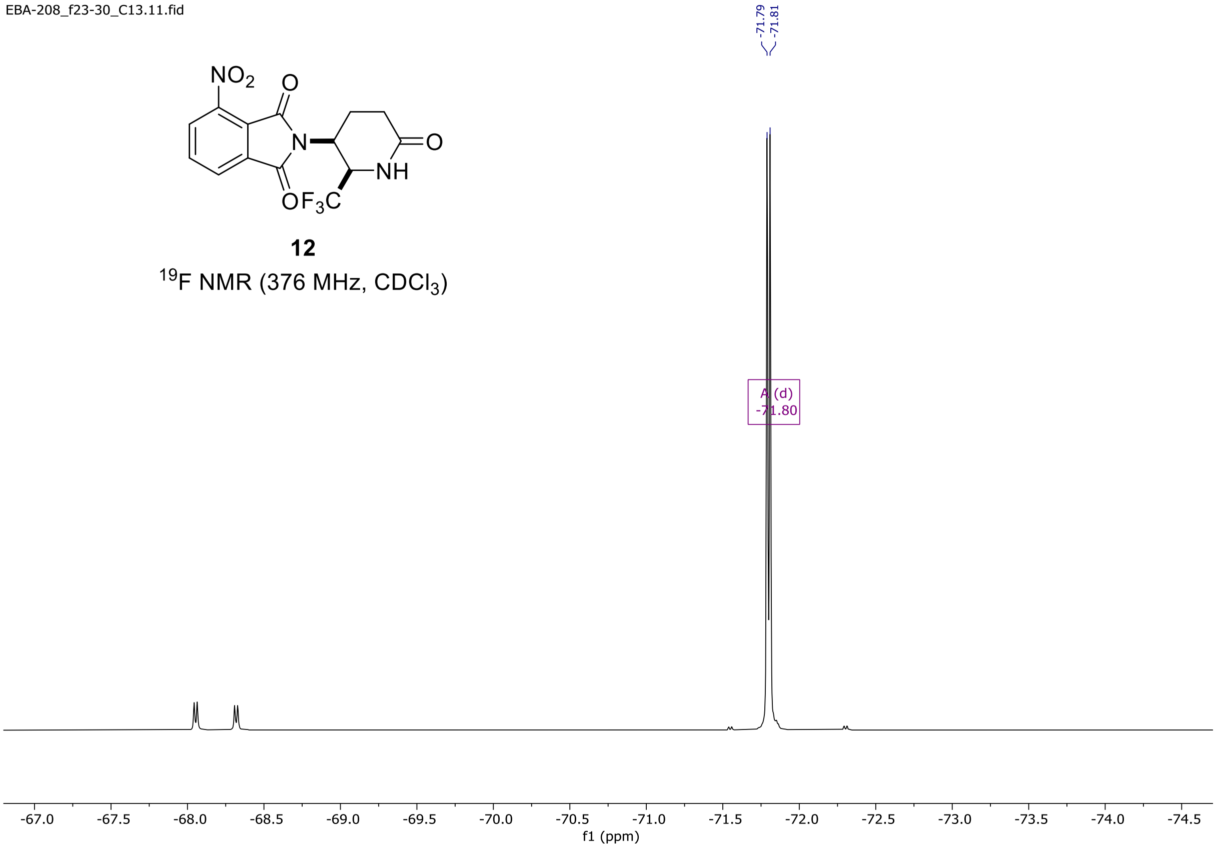

EBA-208_f23-30_C13.20.fid

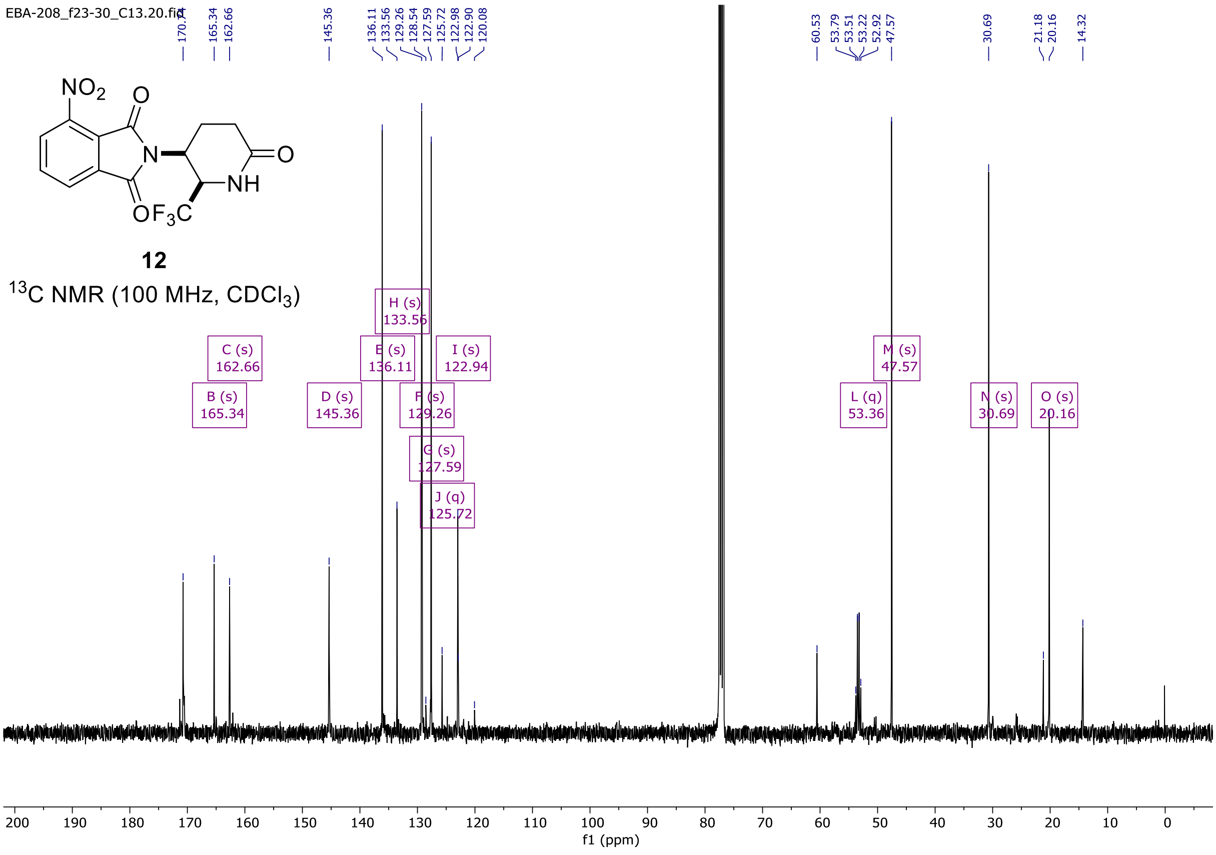

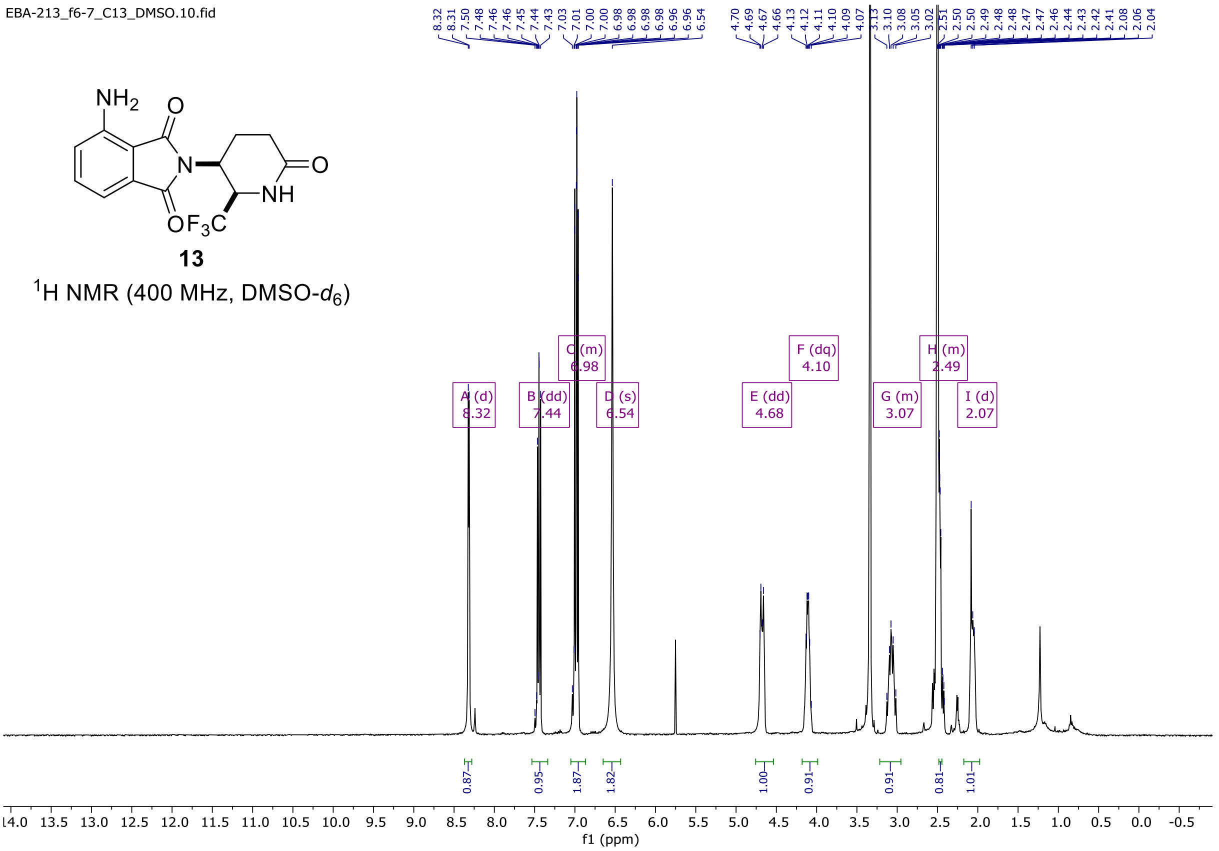
EBA-213_f6-7_C13_DMSO.10.fid
8.32 8.31 7.50 7.48 7.46 7.46 7.45 7.44 7.43 7.03 7.01 7.00 7.00 6.98 6.98 6.98 6.98 6.96 6.96 6.54
4.70 4.69 4.67 4.66 4.13 4.12 4.11 4.10 4.09 4.07 3.13 3.10 3.08 3.05 3.02 2.51 2.50 2.50 2.49 2.48 2.48 2.47 2.47 2.46 2.44 2.43 2.42 2.41 2.08 2.06 2.04
NH2
O
N
O
NH
O
F3C
13
1H NMR (400 MHz, DMSO-d6)
A (d) 8.32
B (dd) 7.44
C (m) 6.98
D (s) 6.54
E (dd) 4.68
F (dq) 4.10
G (m) 3.07
H (m) 2.49
I (d) 2.07
0.87
0.95
1.87
1.82
1.00
0.91
0.91
0.81
1.01
f1 (ppm)

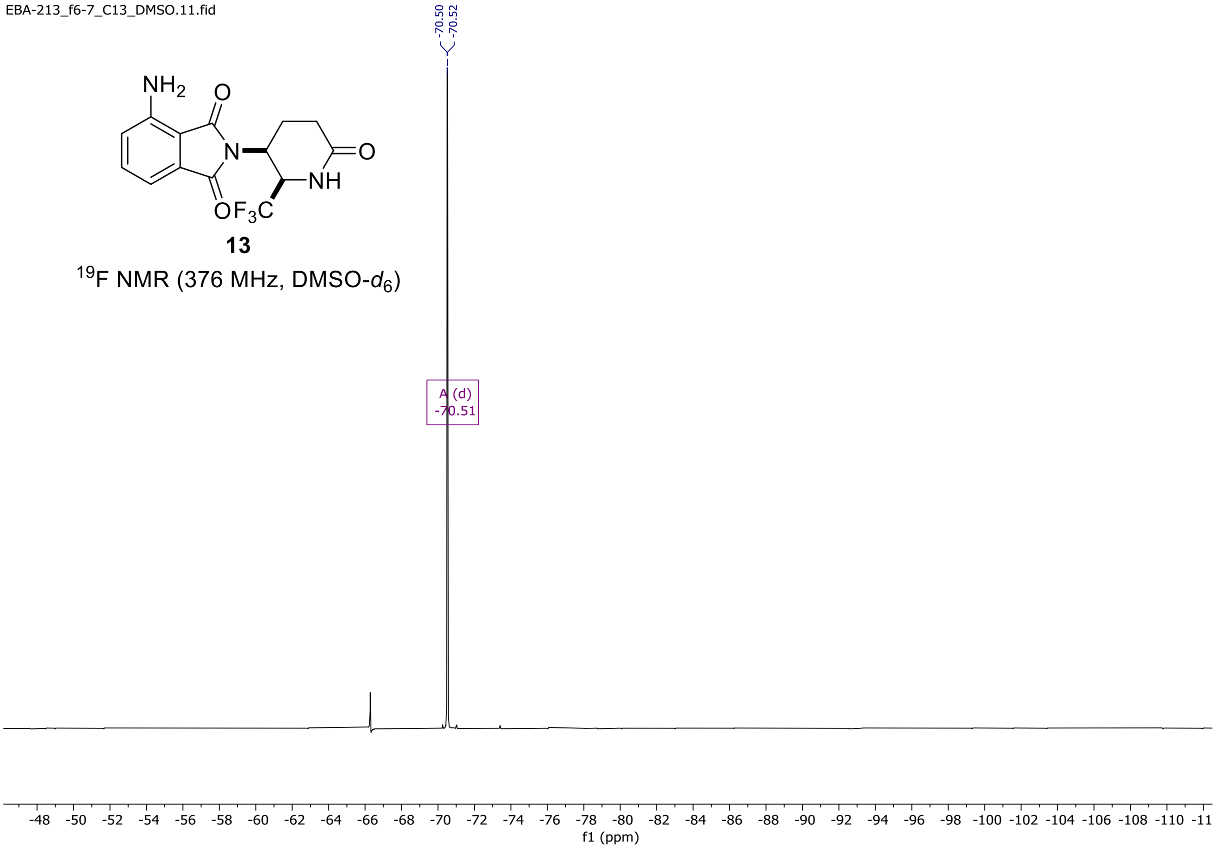
EBA-213_f6-7_C13_DMSO.11.fid
-70.50
-70.52
NH2
O
N
O
NH
O
F3C
13
19F NMR (376 MHz, DMSO-d6)
A (d) -70.51
f1 (ppm)

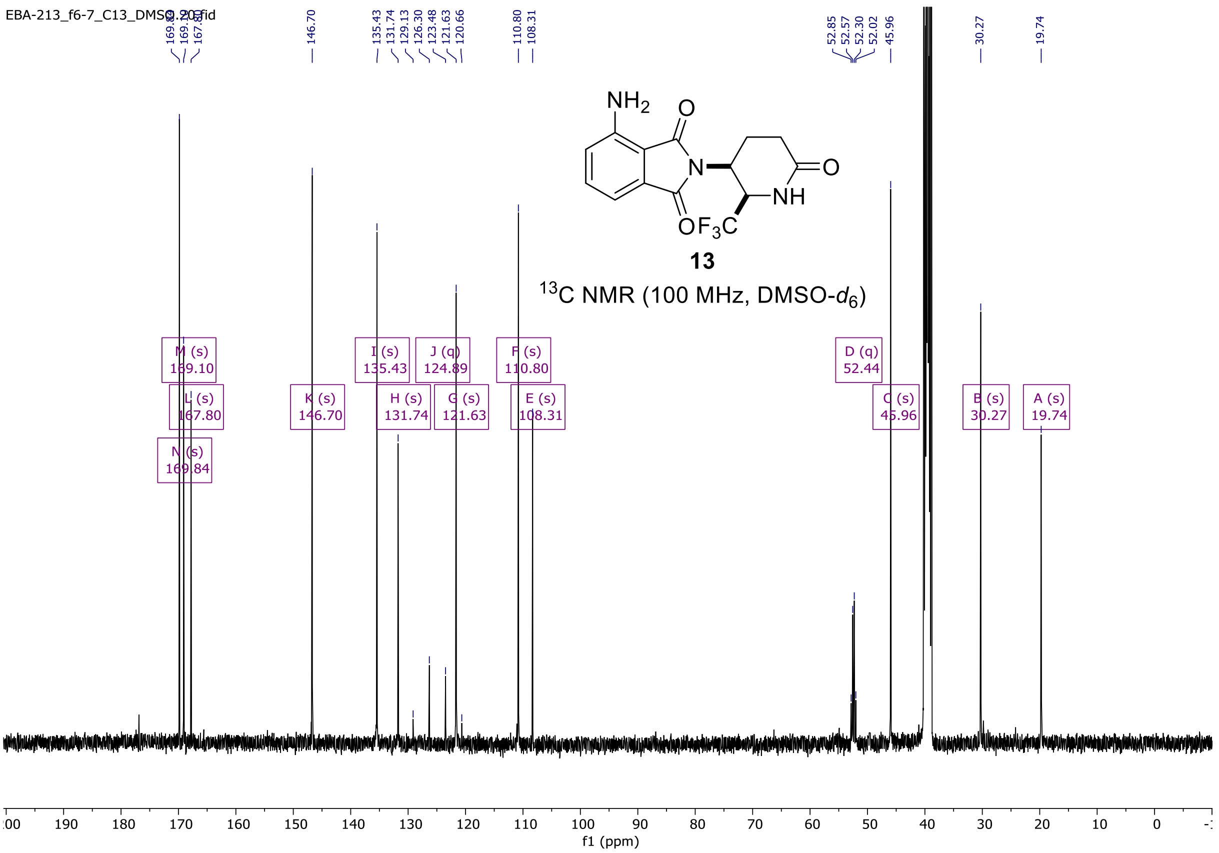
EBA-213_f6-7_C13_DMSO.20.fid
13
^{13}C NMR (100 MHz, DMSO-d_6)
f1 (ppm)

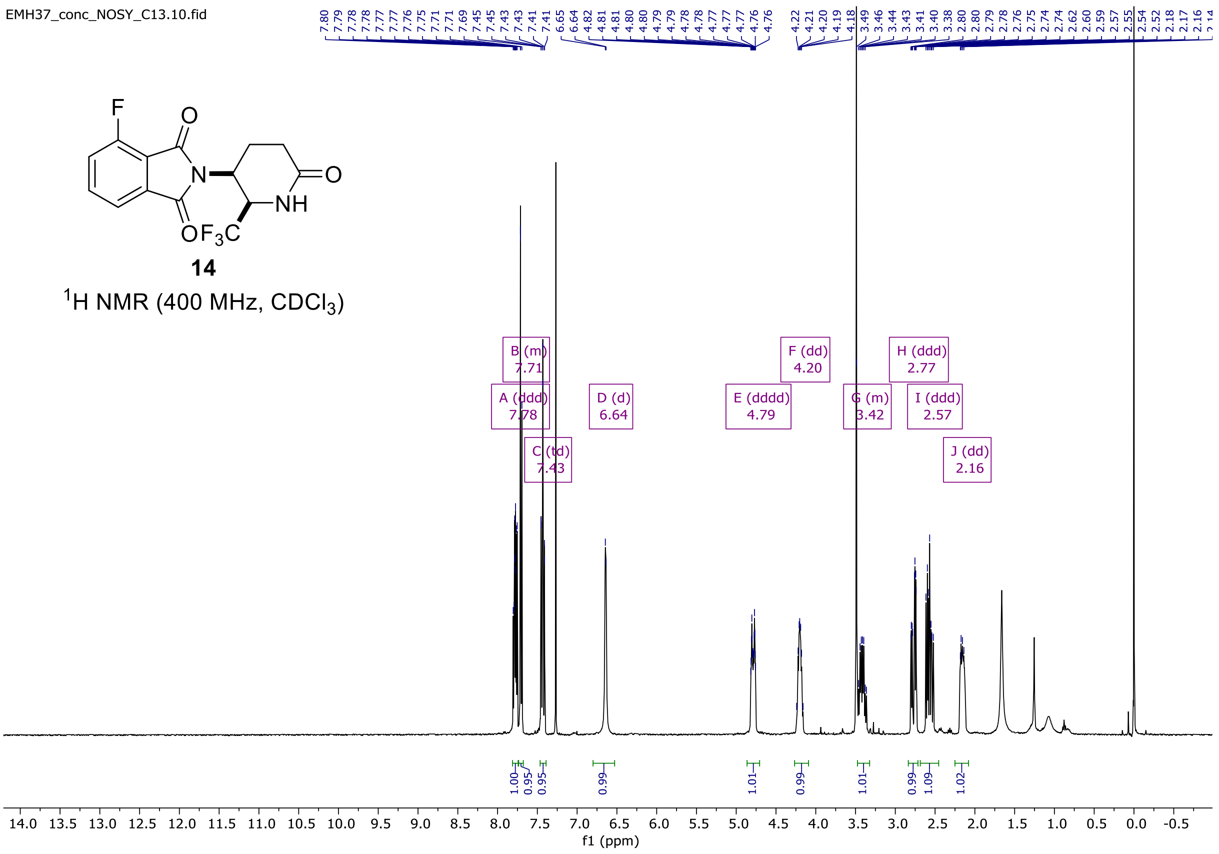
EMH37_conc_NOSY_C13.10.fid
14
^{1}H NMR (400 MHz, CDCl$_3$)
f1 (ppm)

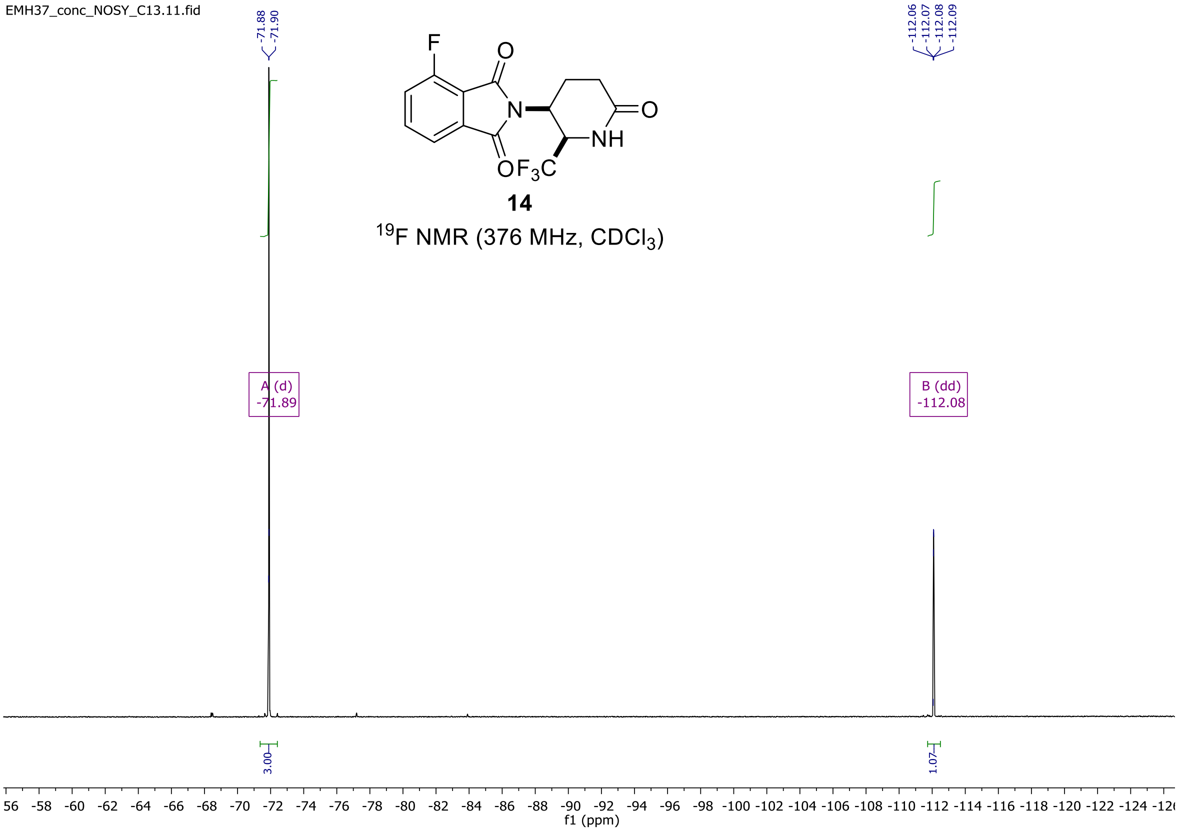
EMH37_conc_NOSY_C13.11.fid
-71.88
-71.90
-112.06
-112.07
-112.08
-112.09
F
O
N
O
NH
O
F3C
14
19F NMR (376 MHz, CDCl3)
A (d)
-71.89
B (dd)
-112.08
3.00
1.07
56 -58 -60 -62 -64 -66 -68 -70 -72 -74 -76 -78 -80 -82 -84 -86 -88 -90 -92 -94 -96 -98 -100 -102 -104 -106 -108 -110 -112 -114 -116 -118 -120 -122 -124
f1 (ppm)

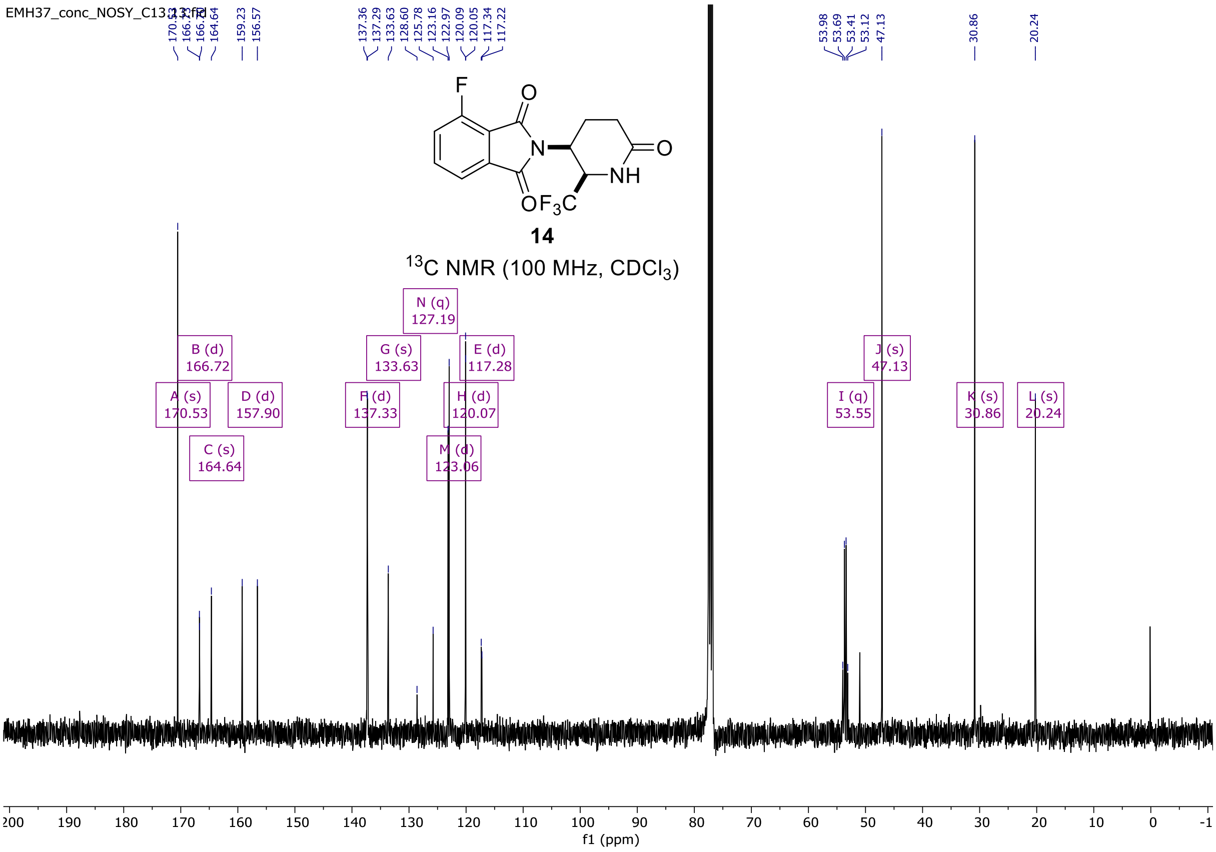
164.64
159.23
156.57
137.36
137.29
133.63
128.60
125.78
123.16
122.97
120.09
120.05
117.34
117.22
53.98
53.69
53.41
53.12
47.13
30.86
20.24
F
O
N
O
NH
O
F3C
14
13C NMR (100 MHz, CDCl3)
N (q)
127.19
B (d)
166.72
G (s)
133.63
E (d)
117.28
J (s)
47.13
A (s)
170.53
D (d)
157.90
F (d)
137.33
H (d)
120.07
I (q)
53.55
K (s)
30.86
L (s)
20.24
C (s)
164.64
M (d)
123.06
200 190 180 170 160 150 140 130 120 110 100 90 80 70 60 50 40 30 20 10 0
f1 (ppm)

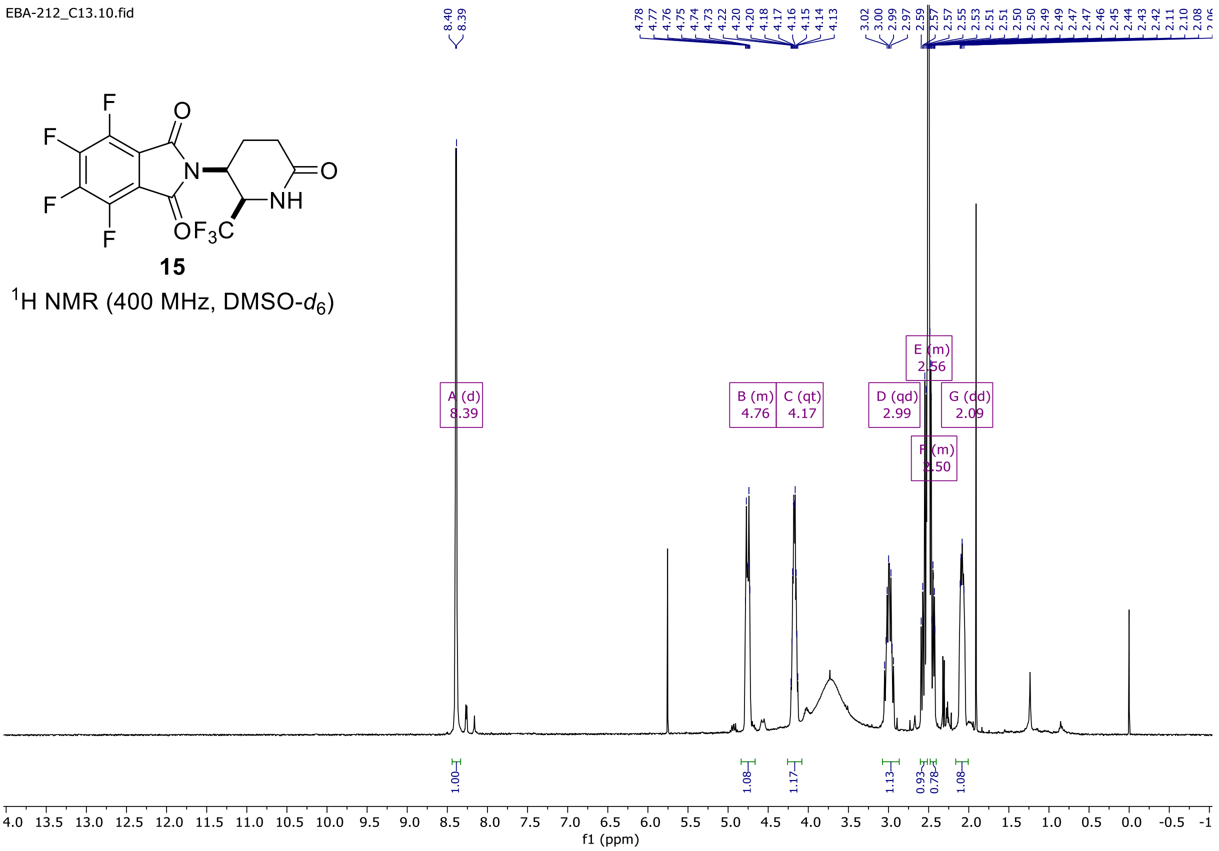

EBA-212_C13.10.fid
15
^{1}H NMR (400 MHz, DMSO-d_6)
A (d) 8.39
B (m) 4.76
C (qt) 4.17
D (qd) 2.99
E (m) 2.56
F (m) 2.50
G (dd) 2.09
f1 (ppm)

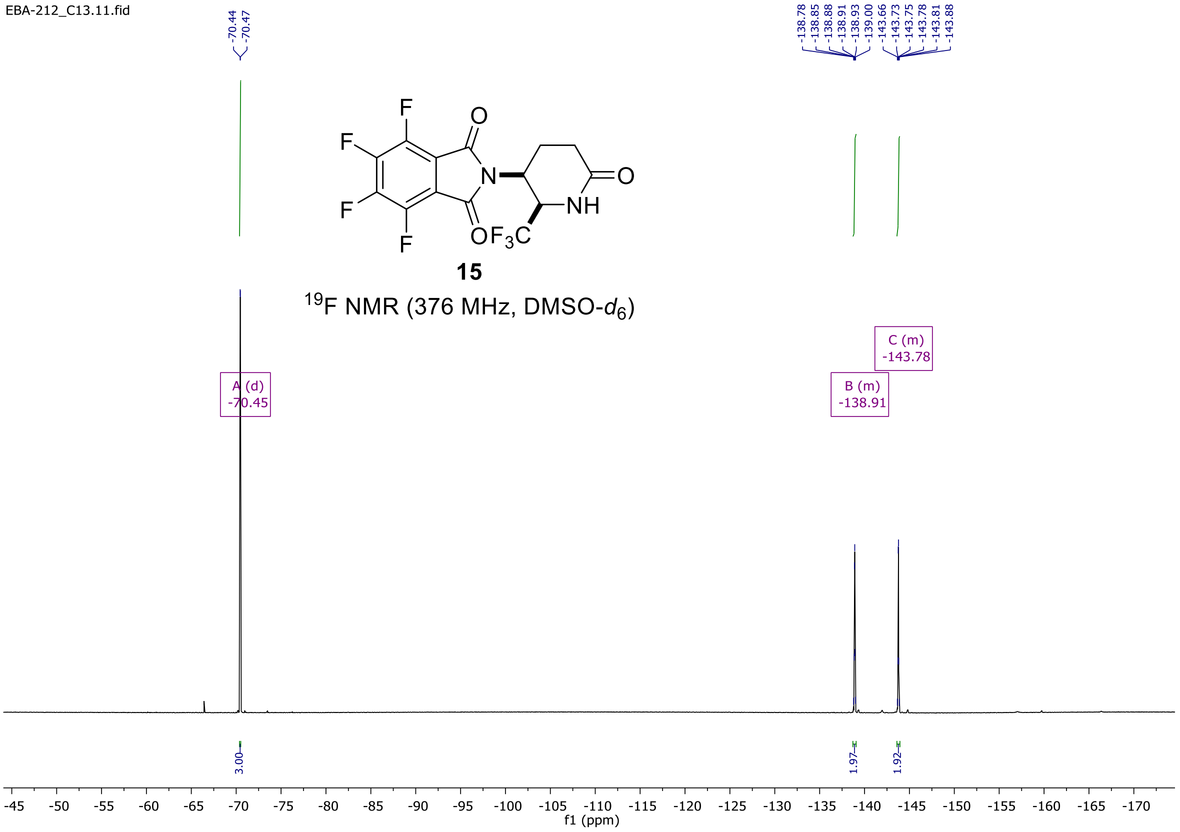

EBA-212_C13.11.fid
15
^{19}F NMR (376 MHz, DMSO-d_6)
A (d) -70.45
B (m) -138.91
C (m) -143.78
f1 (ppm)

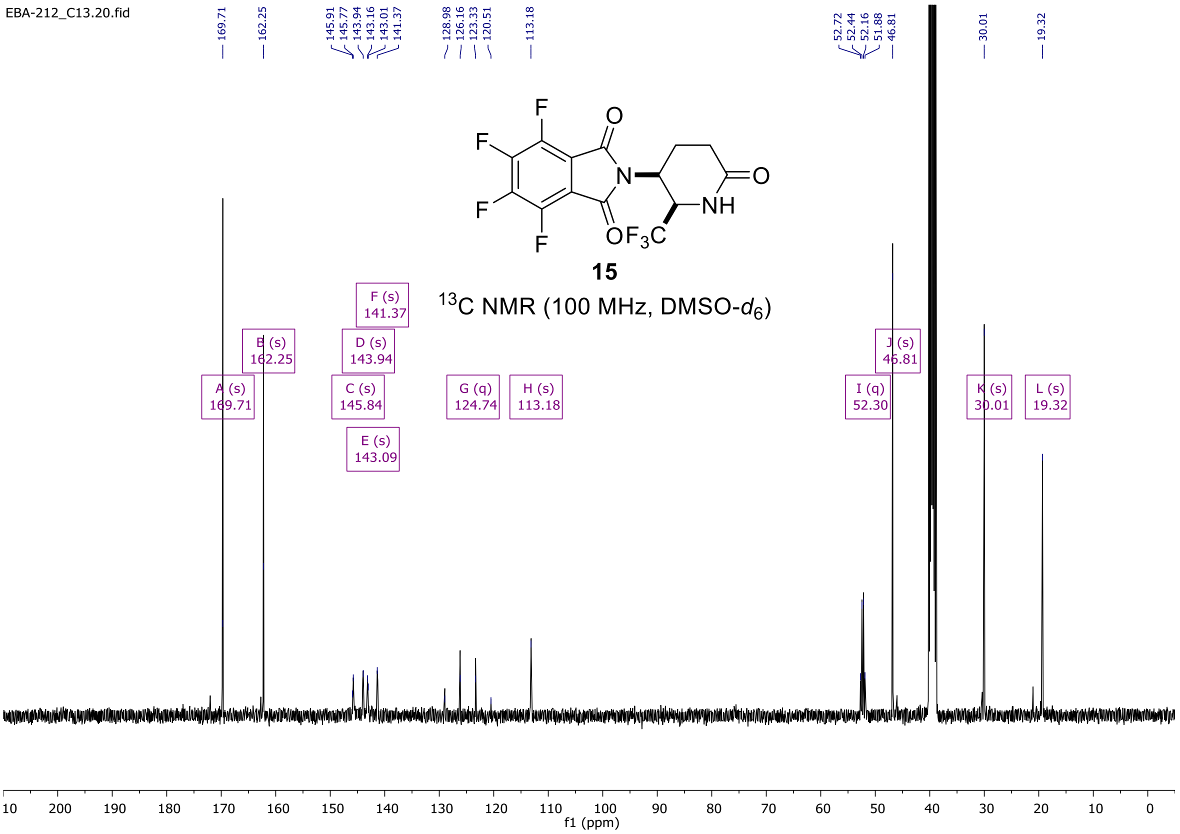
EBA-212_C13.20.fid
169.71
162.25
145.91
145.77
143.94
143.16
143.01
141.37
128.98
126.16
123.33
120.51
113.18
52.72
52.44
52.16
51.88
46.81
30.01
19.32
15
^{13}C NMR (100 MHz, DMSO-d_6)
A (s) 169.71
B (s) 162.25
C (s) 145.84
D (s) 143.94
E (s) 143.09
F (s) 141.37
G (q) 124.74
H (s) 113.18
I (q) 52.30
J (s) 46.81
K (s) 30.01
L (s) 19.32
f1 (ppm)

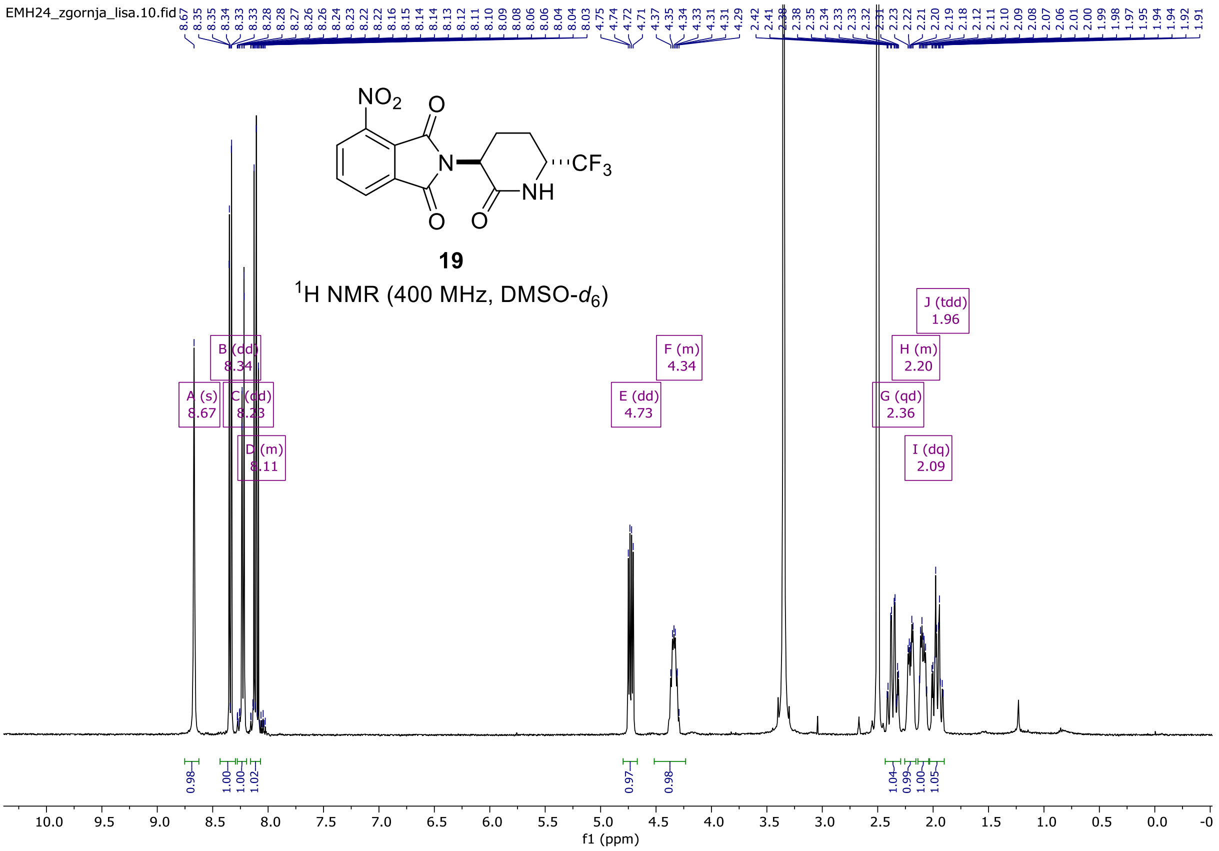
EMH24_zgornja_lisa.10.fid
19
^{1}H NMR (400 MHz, DMSO-d_6)
A (s) 8.67
B (dd) 8.34
C (dd) 8.23
D (m) 8.11
E (dd) 4.73
F (m) 4.34
G (qd) 2.36
H (m) 2.20
I (dq) 2.09
J (tdd) 1.96
f1 (ppm)

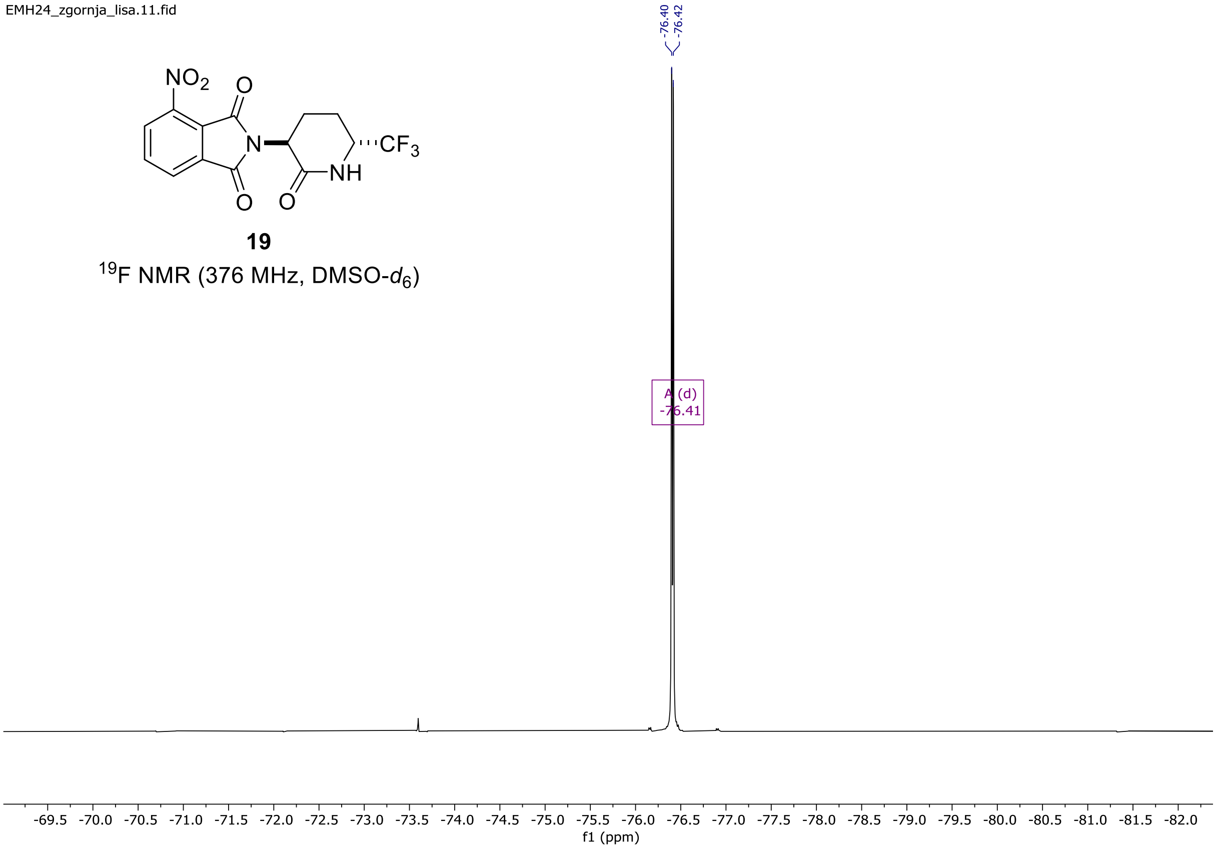
EMH24_zgornja_lisa.11.fid
-76.40
-76.42
NO2
CF3
NH
19
19F NMR (376 MHz, DMSO-d6)
A (d)
-76.41
-69.5 -70.0 -70.5 -71.0 -71.5 -72.0 -72.5 -73.0 -73.5 -74.0 -74.5 -75.0 -75.5 -76.0 -76.5 -77.0 -77.5 -78.0 -78.5 -79.0 -79.5 -80.0 -80.5 -81.0 -81.5 -82.0
f1 (ppm)

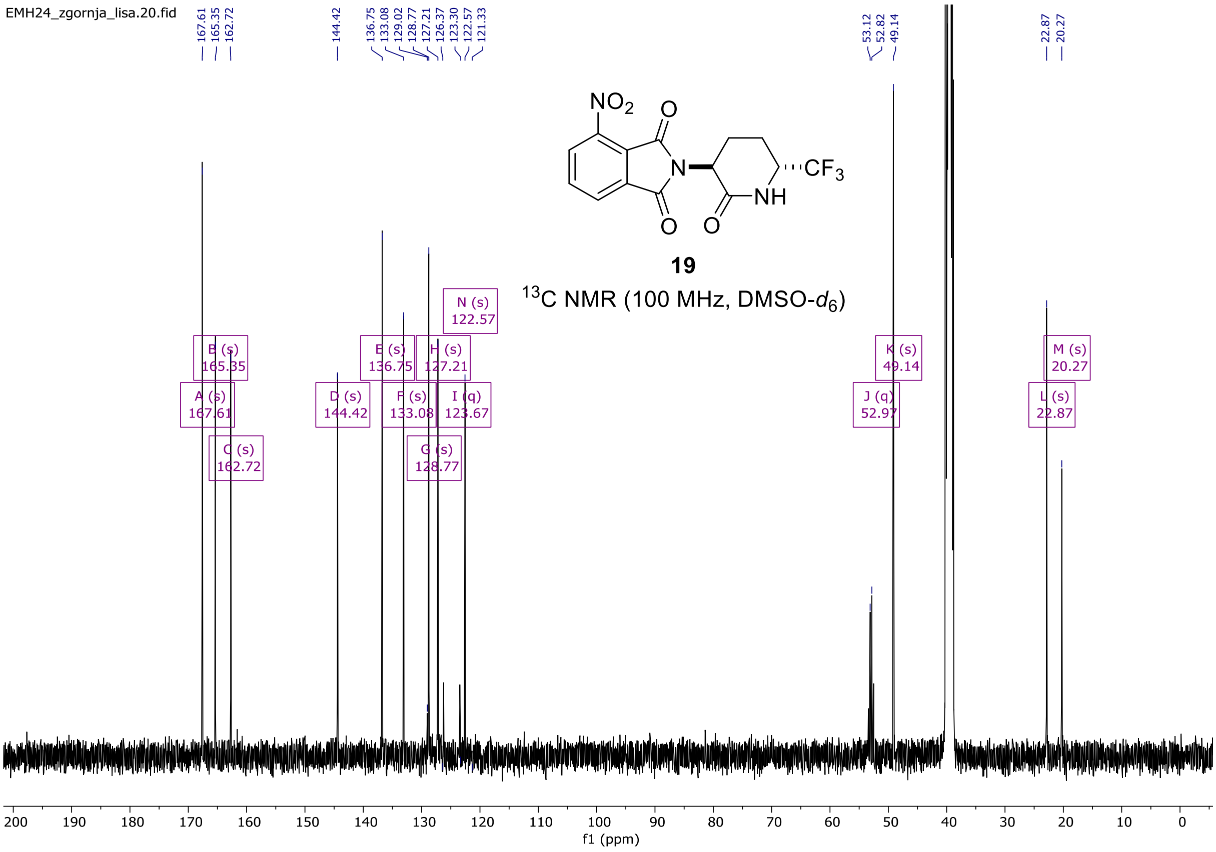
EMH24_zgornja_lisa.20.fid
167.61
165.35
162.72
144.42
136.75
133.08
129.02
128.77
127.21
126.37
123.30
122.57
121.33
53.12
52.82
49.14
22.87
20.27
NO2
CF3
NH
19
13C NMR (100 MHz, DMSO-d6)
N (s)
122.57
B (s)
165.35
E (s)
136.75
H (s)
127.21
K (s)
49.14
M (s)
20.27
A (s)
167.61
D (s)
144.42
F (s)
133.08
I (q)
123.67
J (q)
52.97
L (s)
22.87
C (s)
162.72
G (s)
128.77
200 190 180 170 160 150 140 130 120 110 100 90 80 70 60 50 40 30 20 10 0
f1 (ppm)

EMH28-conc.10.fid

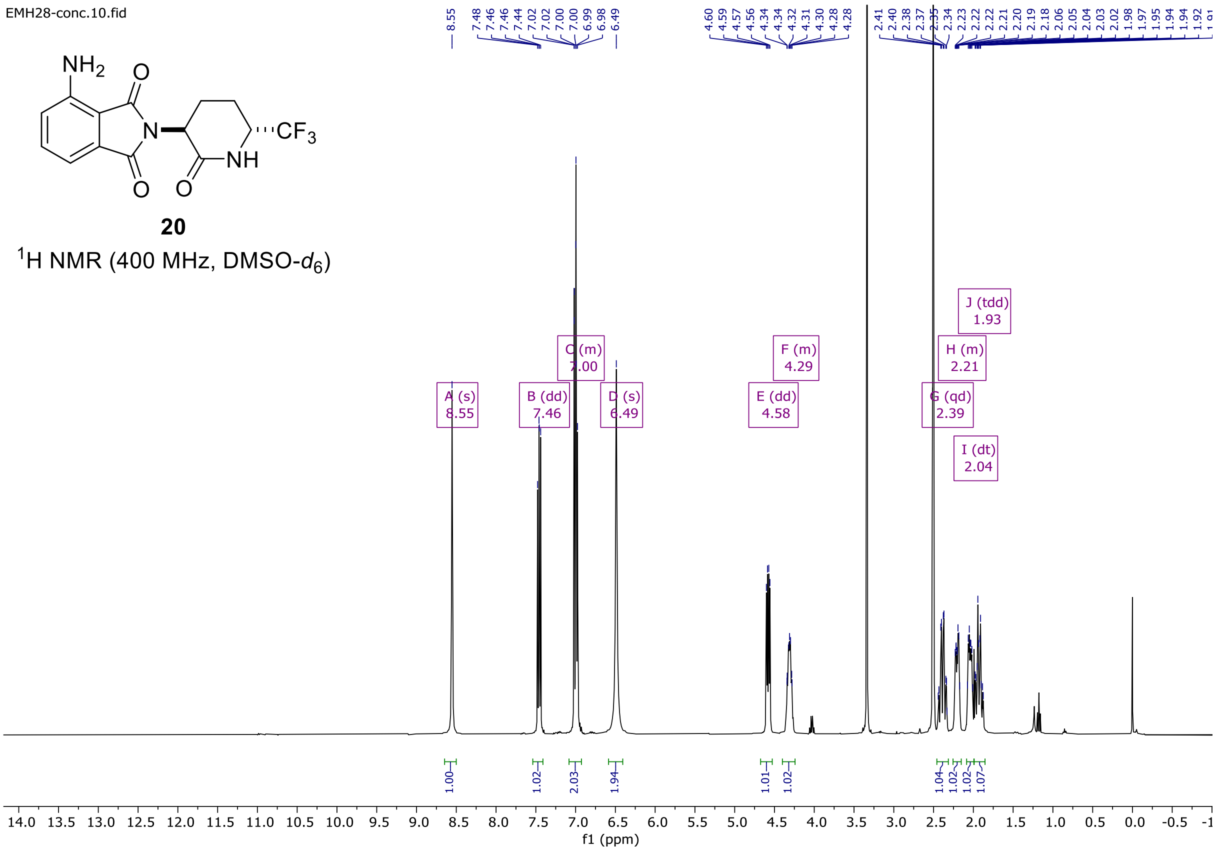

20

^{1}H NMR (400 MHz, DMSO-d_6)

EMH28-conc.11.fid

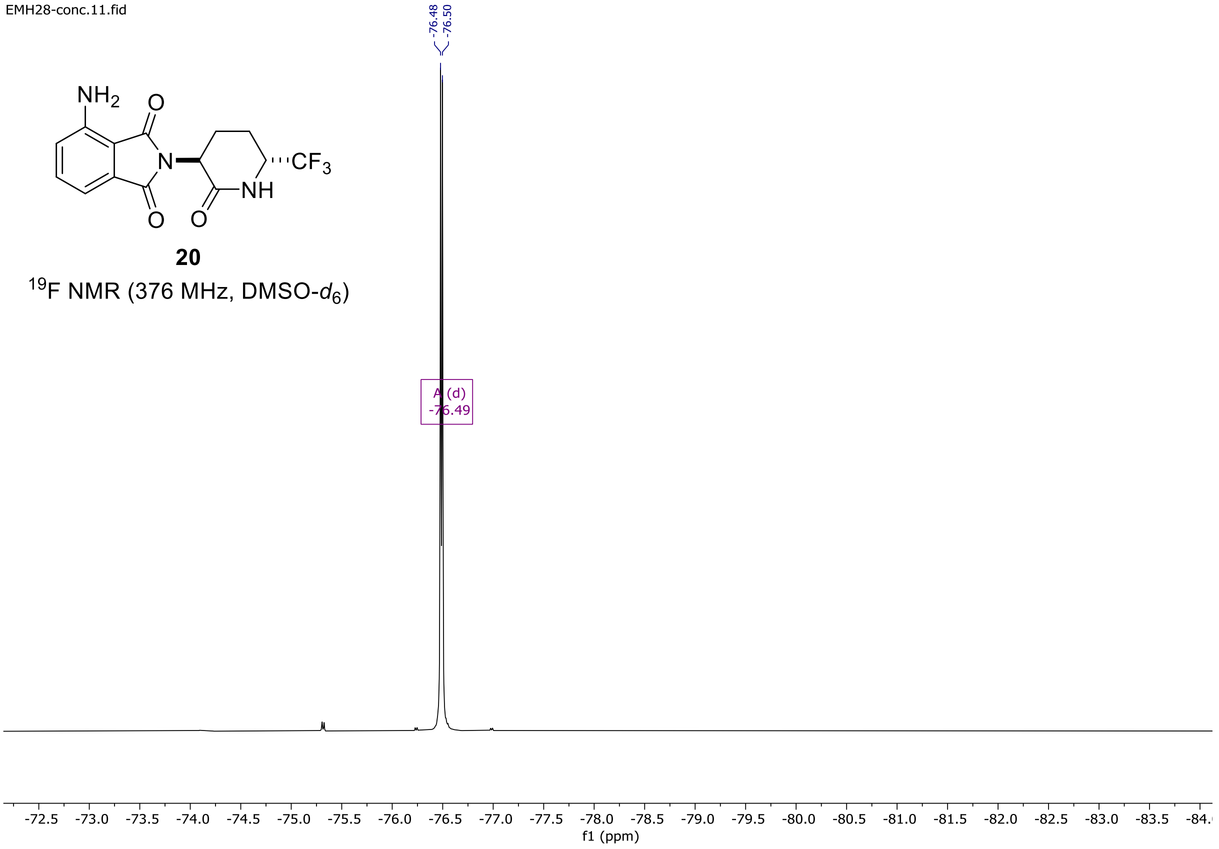

20

^{19}F NMR (376 MHz, DMSO-d_6)

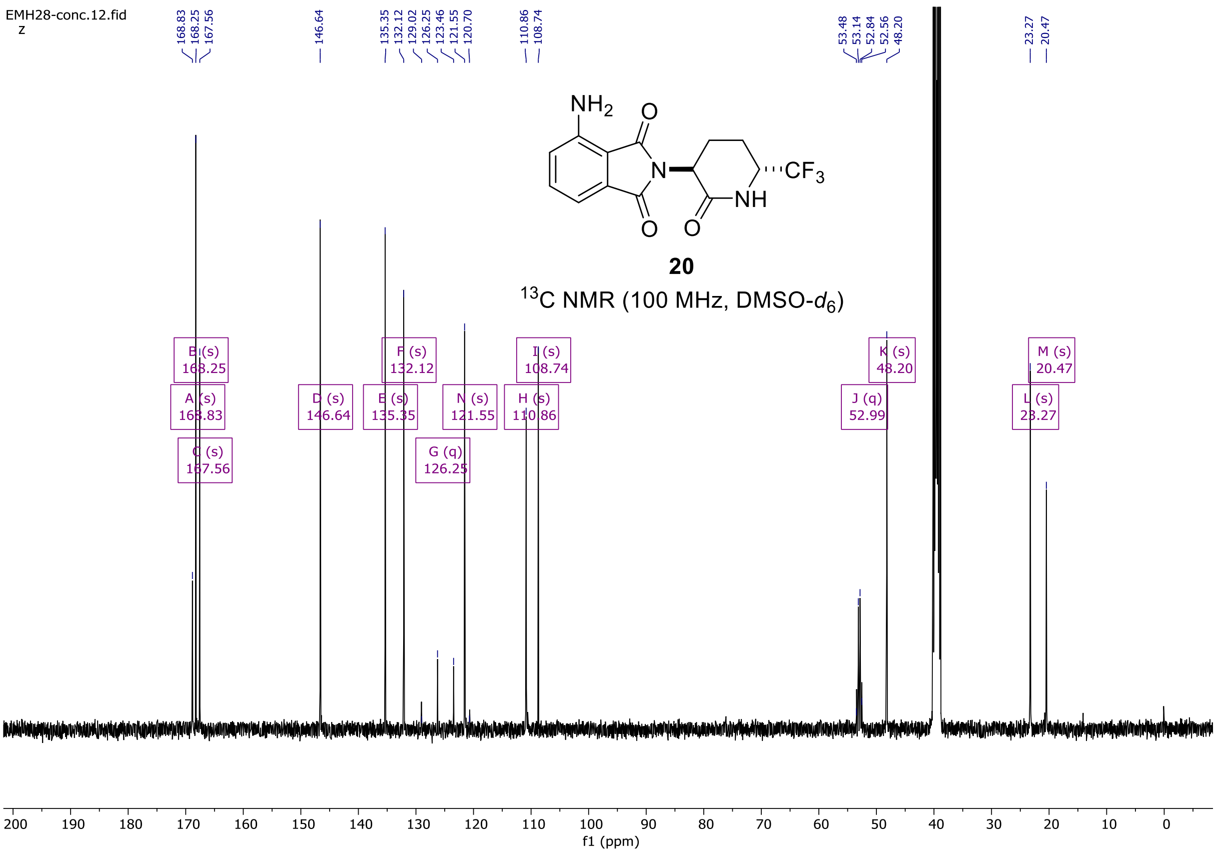
EMH28-conc.12.fid
z
168.83
168.25
167.56
146.64
135.35
132.12
129.02
126.25
123.46
121.55
120.70
110.86
108.74
53.48
53.14
52.84
52.56
48.20
23.27
20.47
NH2
O
N
CF3
NH
O
O
20
13C NMR (100 MHz, DMSO-d6)
B (s) 168.25
A (s) 168.83
C (s) 167.56
D (s) 146.64
E (s) 135.35
F (s) 132.12
G (q) 126.25
N (s) 121.55
H (s) 110.86
I (s) 108.74
J (q) 52.99
K (s) 48.20
L (s) 23.27
M (s) 20.47
f1 (ppm)

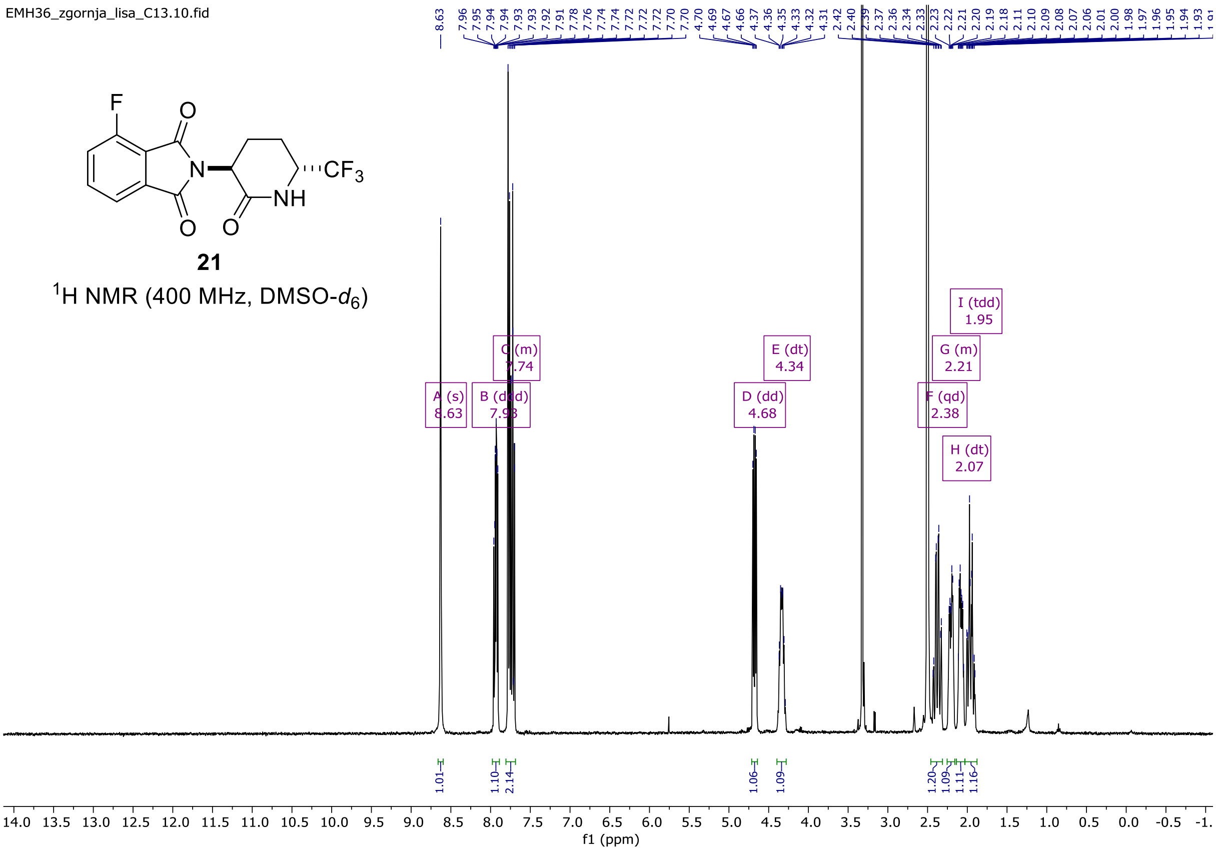
EMH36_zgornja_lisa_C13.10.fid
F
O
N
CF3
NH
O
O
21
1H NMR (400 MHz, DMSO-d6)
A (s) 8.63
B (ddd) 7.93
C (m) 7.74
D (dd) 4.68
E (dt) 4.34
F (qd) 2.38
G (m) 2.21
H (dt) 2.07
I (tdd) 1.95
1.01
1.10
2.14
1.06
1.09
1.20
1.09
1.11
1.16
f1 (ppm)

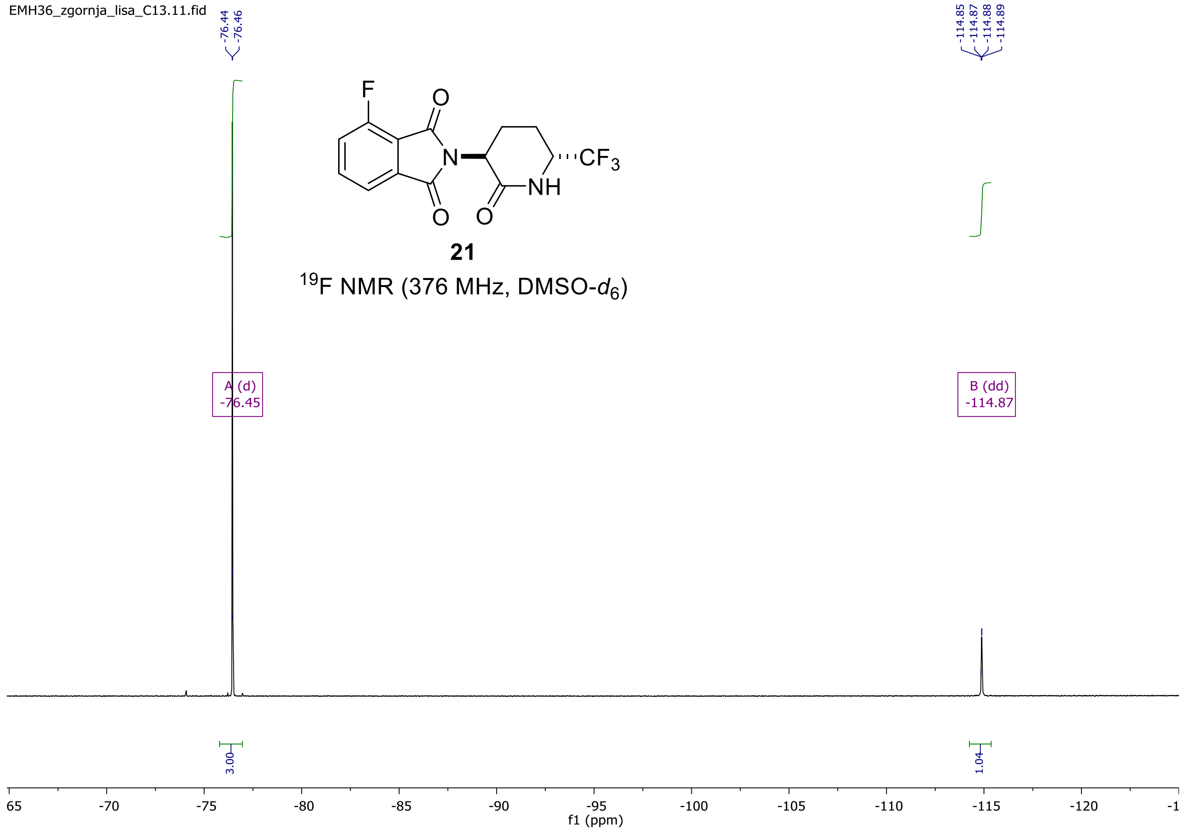
EMH36_zgornja_lisa_C13.11.fid
-76.44
-76.46
-114.85
-114.87
-114.88
-114.89
21
^{19}F NMR (376 MHz, DMSO-d_6)
A (d)
-76.45
B (dd)
-114.87
3.00
1.04
f1 (ppm)

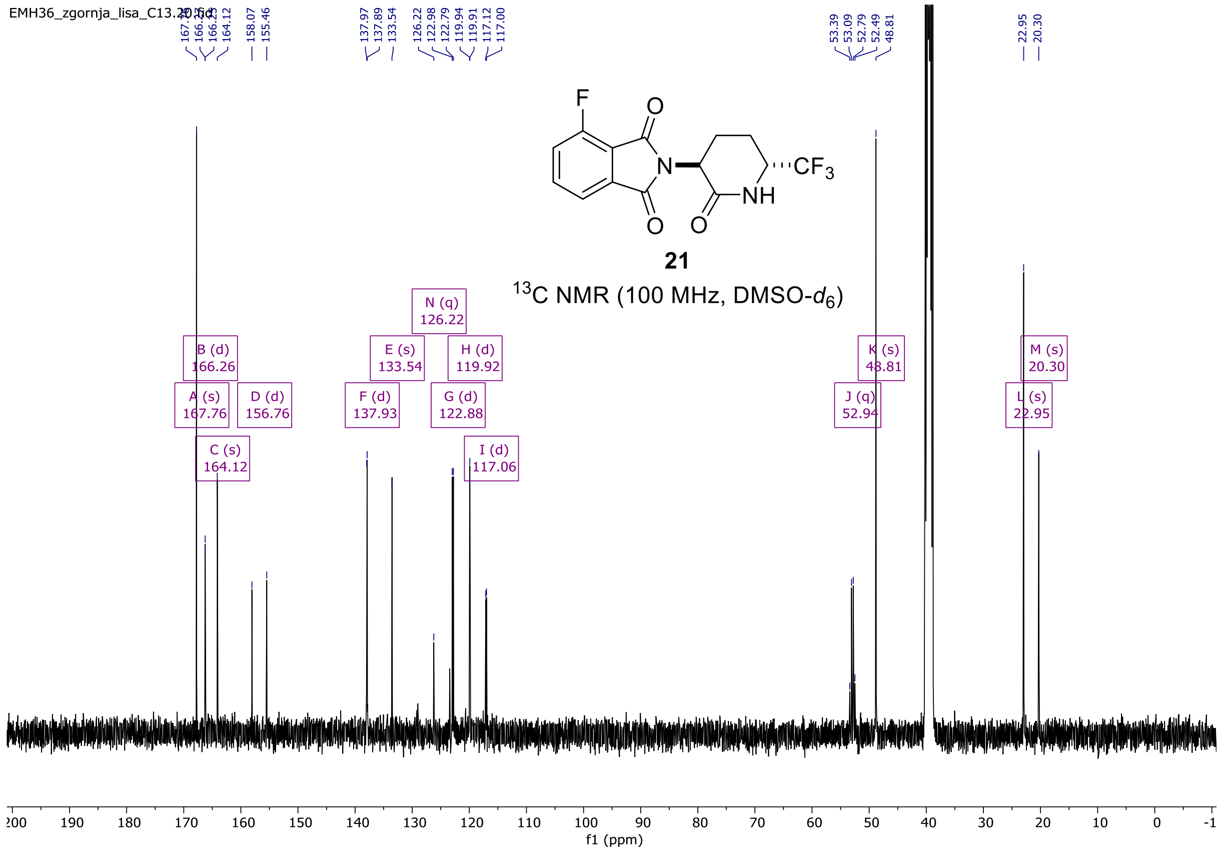
167.76
166.26
164.12
158.07
155.46
137.97
137.89
133.54
126.22
122.98
122.79
119.94
119.91
117.12
117.00
53.39
53.09
52.79
52.49
48.81
22.95
20.30
21
^{13}C NMR (100 MHz, DMSO-d_6)
N (q)
126.22
B (d)
166.26
E (s)
133.54
H (d)
119.92
K (s)
48.81
M (s)
20.30
A (s)
167.76
D (d)
156.76
F (d)
137.93
G (d)
122.88
J (q)
52.94
L (s)
22.95
C (s)
164.12
I (d)
117.06
f1 (ppm)

EMH31_zgornja_lisa_C13.10.fid

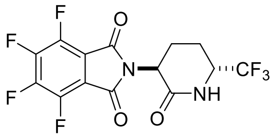

22

^{1}H NMR (400 MHz, DMSO-d_6)

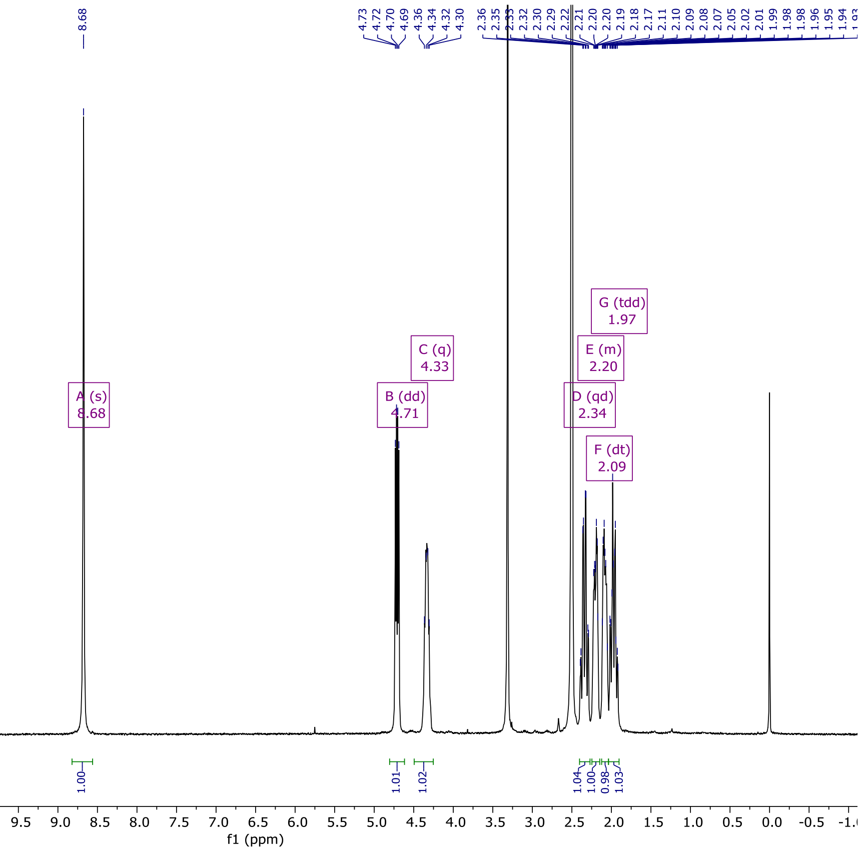

EMH31_zgornja_lisa_C13.11.fid

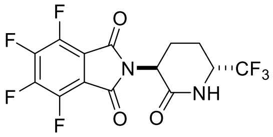

22

^{19}F NMR (376 MHz, DMSO-d_6)

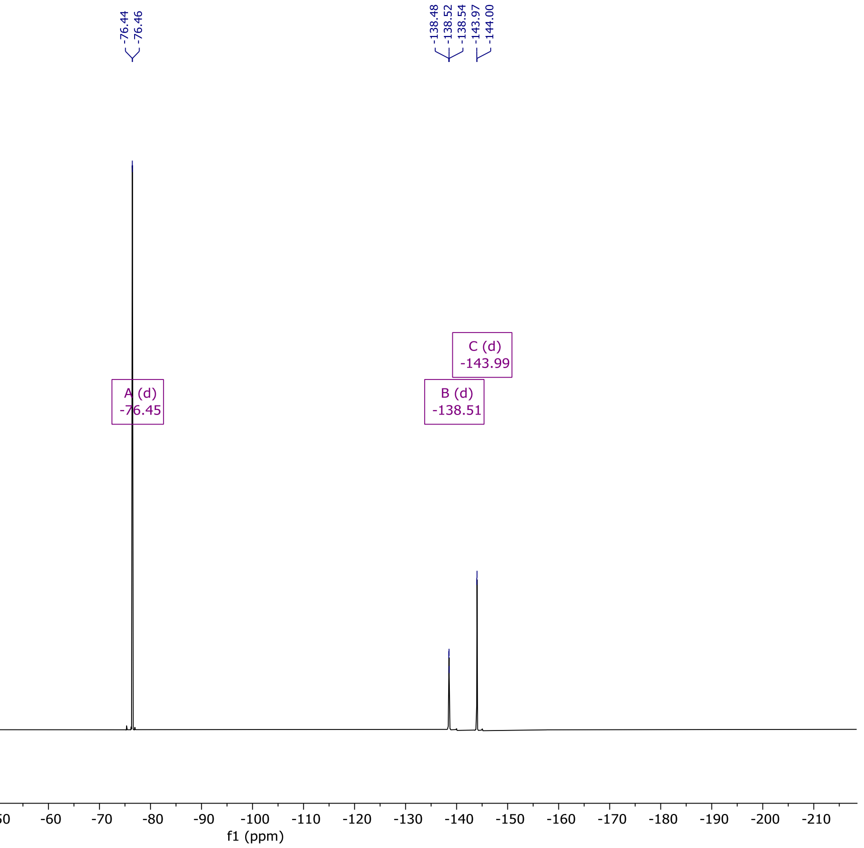

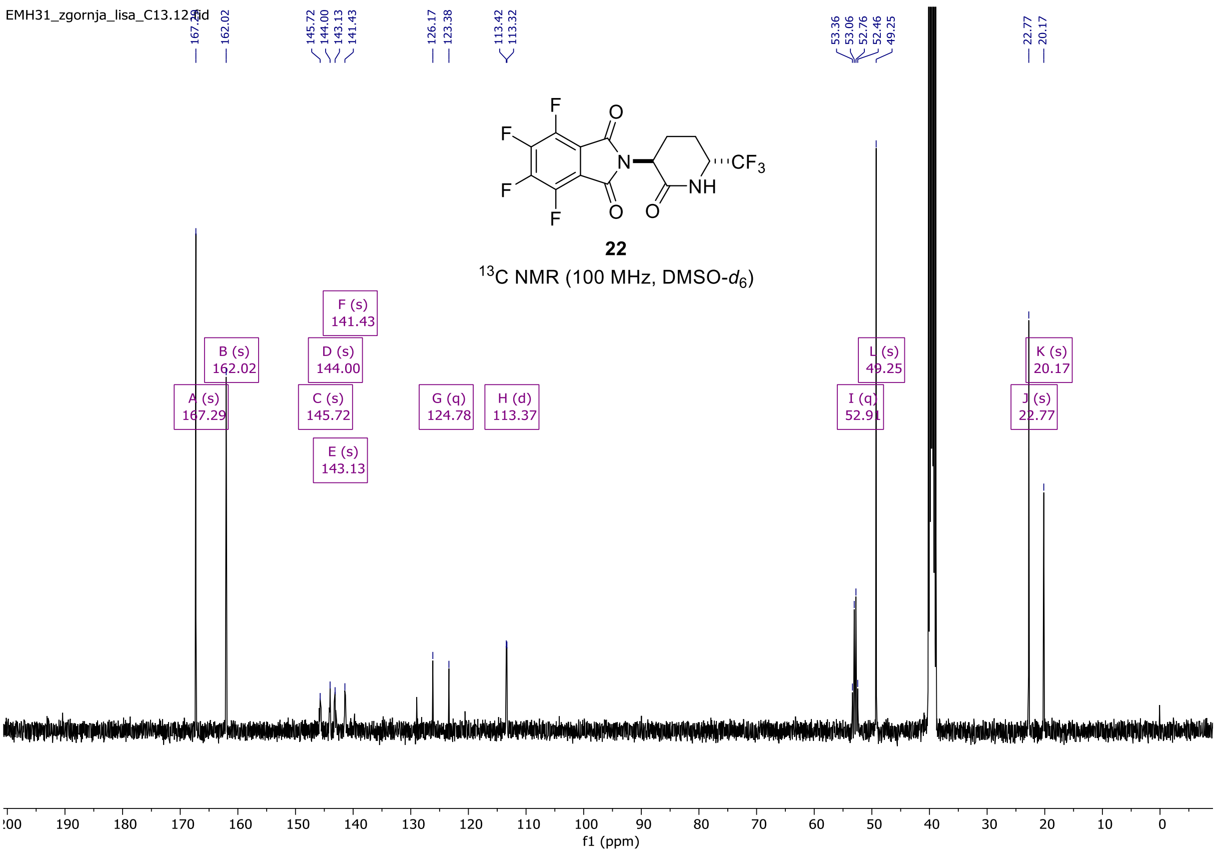
EMH31_zgornja_lisa_C13.12.fid
22
^{13}C NMR (100 MHz, DMSO-d_6)
A (s) 167.29
B (s) 162.02
C (s) 145.72
D (s) 144.00
E (s) 143.13
F (s) 141.43
G (q) 124.78
H (d) 113.37
I (q) 52.91
L (s) 49.25
J (s) 22.77
K (s) 20.17
f1 (ppm)

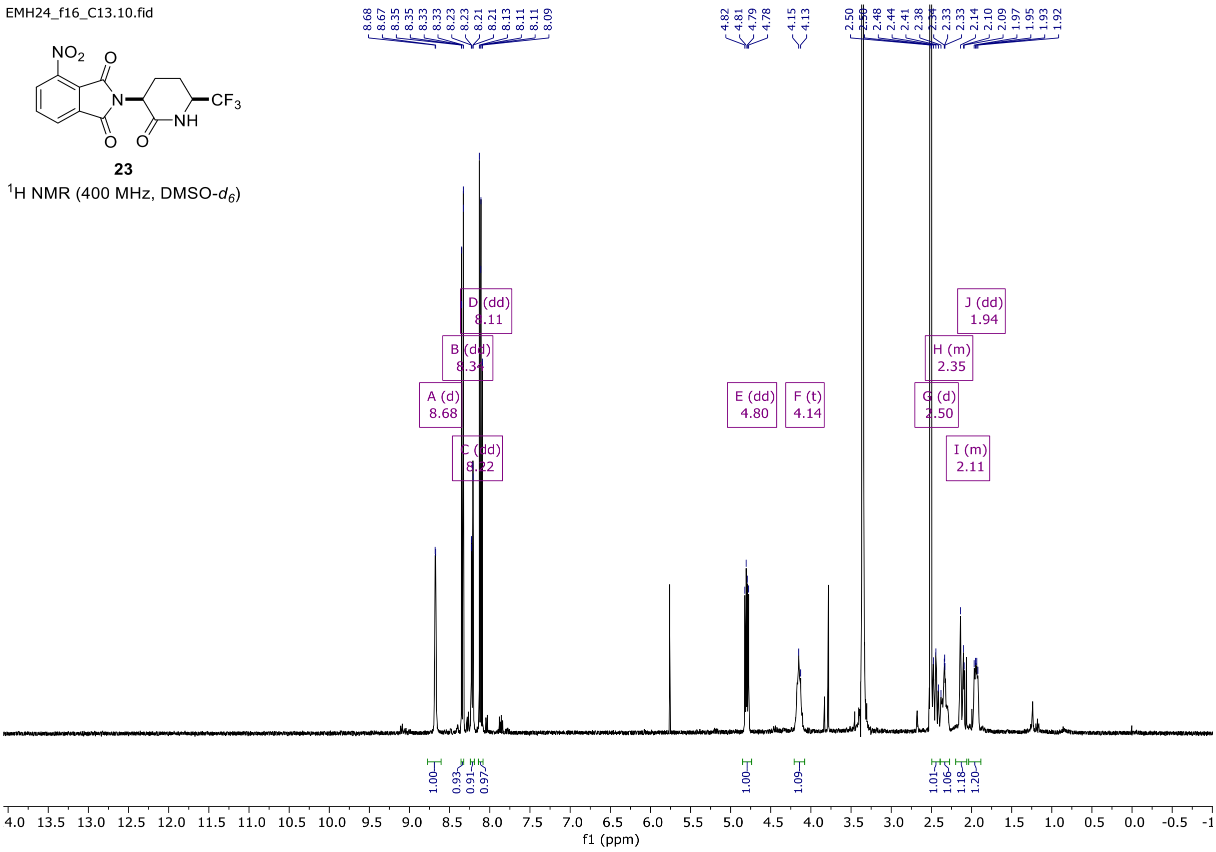
EMH24_f16_C13.10.fid
23
^{1}H NMR (400 MHz, DMSO-d_6)
A (d) 8.68
B (dd) 8.34
C (dd) 8.22
D (dd) 8.11
E (dd) 4.80
F (t) 4.14
G (d) 2.50
H (m) 2.35
I (m) 2.11
J (dd) 1.94
f1 (ppm)

EMH24_f16_C13.11.fid

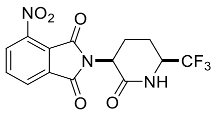

23

^{19}F NMR (376 MHz, DMSO-d_6)

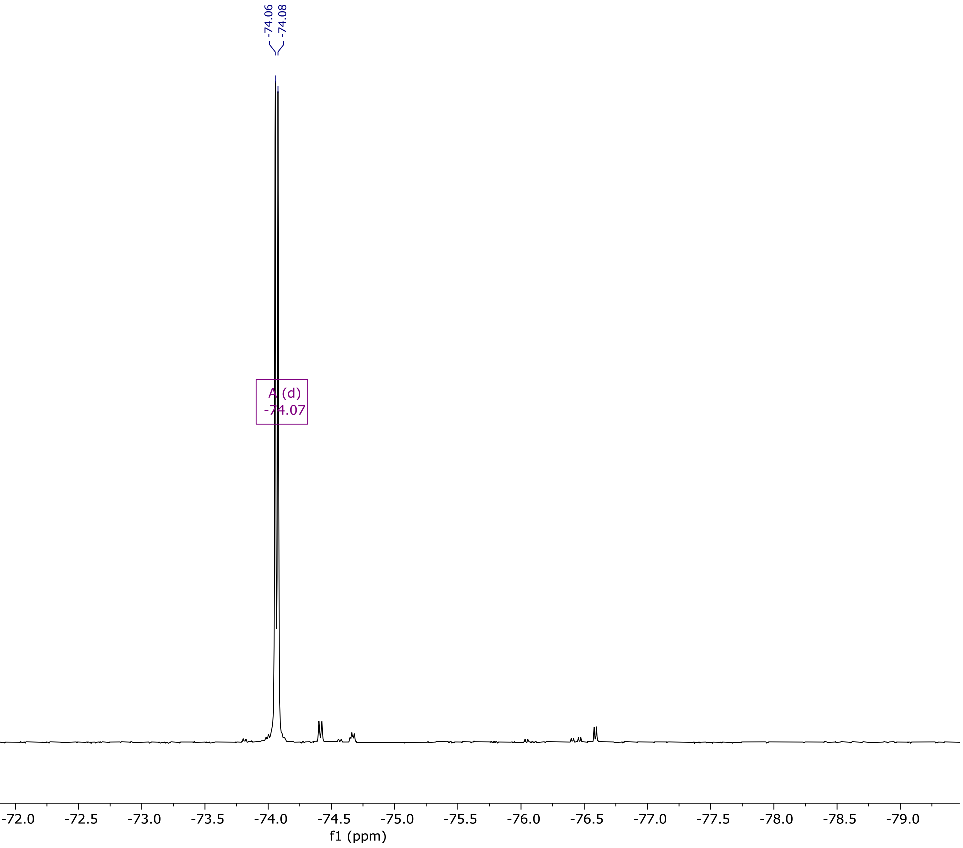

EMH24_f16_C13.20.fid

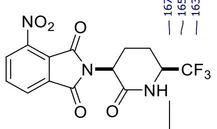

23

^{13}C NMR (100 MHz, DMSO-d_6)

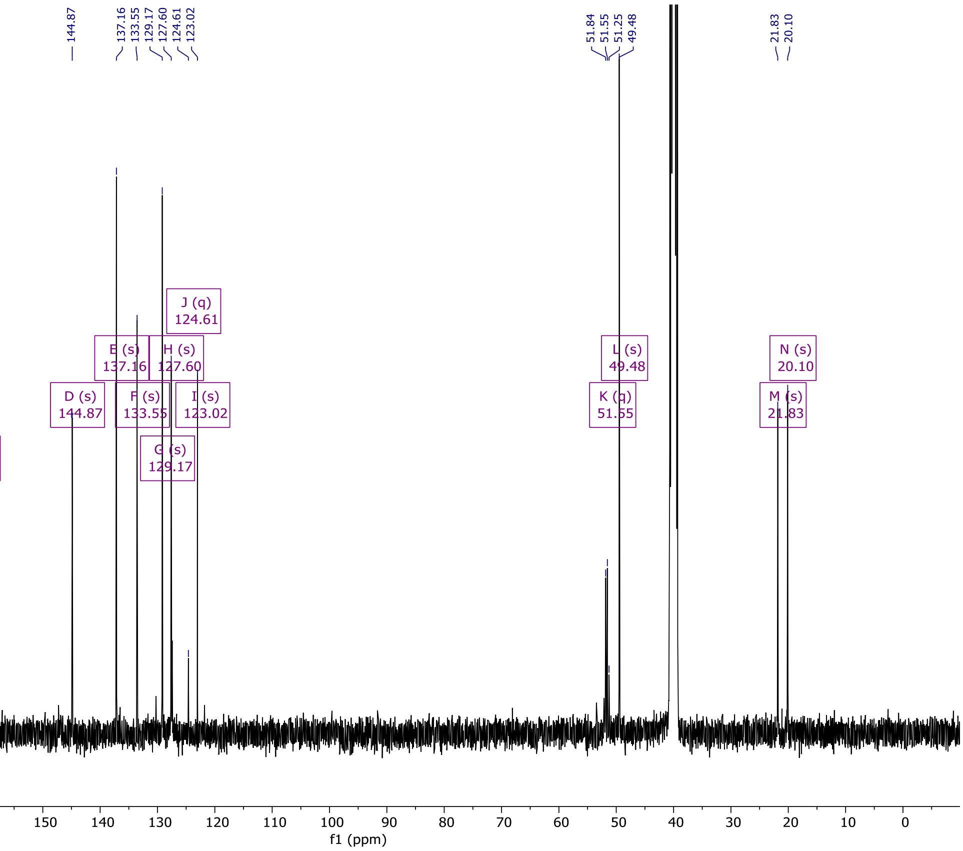

EMH29_C13.10.fid

24

^{1}H NMR (400 MHz, DMSO-d_6)

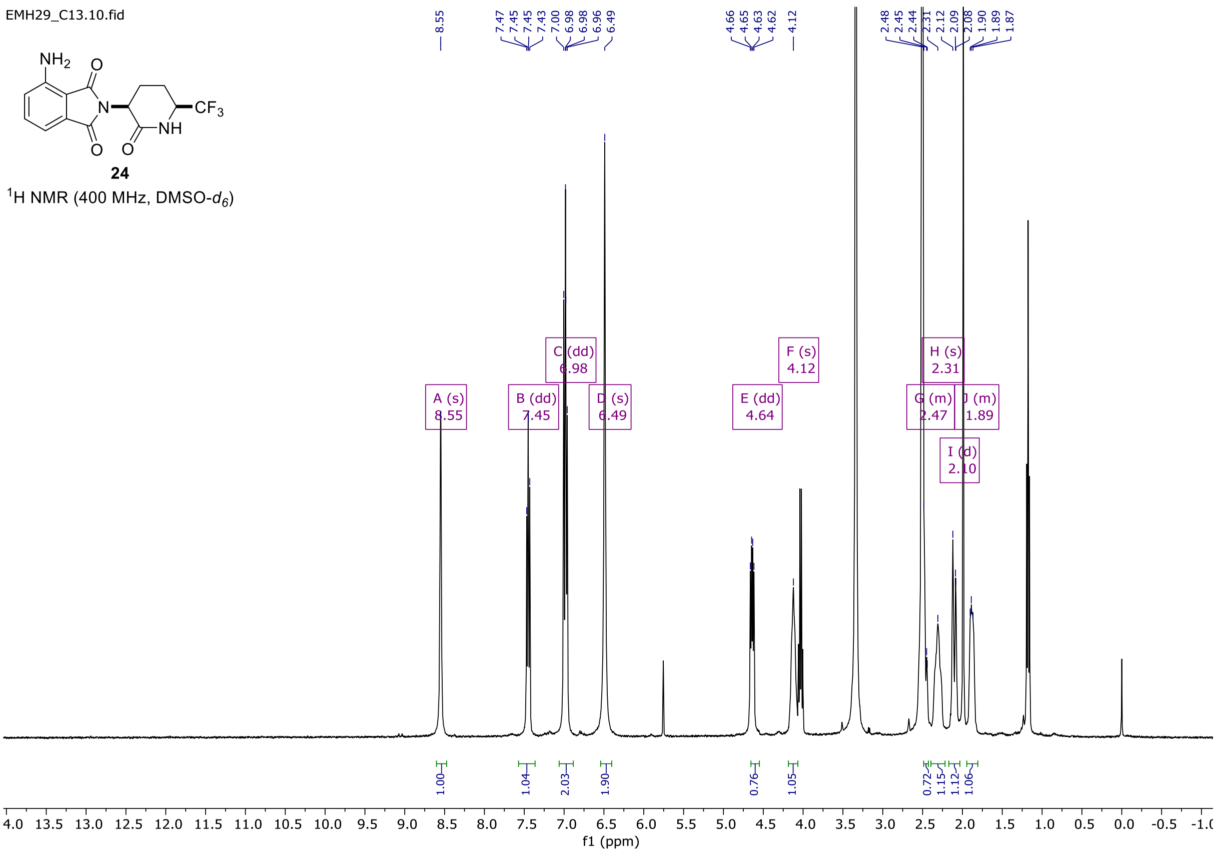

EMH29_C13.11.fid

24

^{19}F NMR (376 MHz, DMSO-d_6)

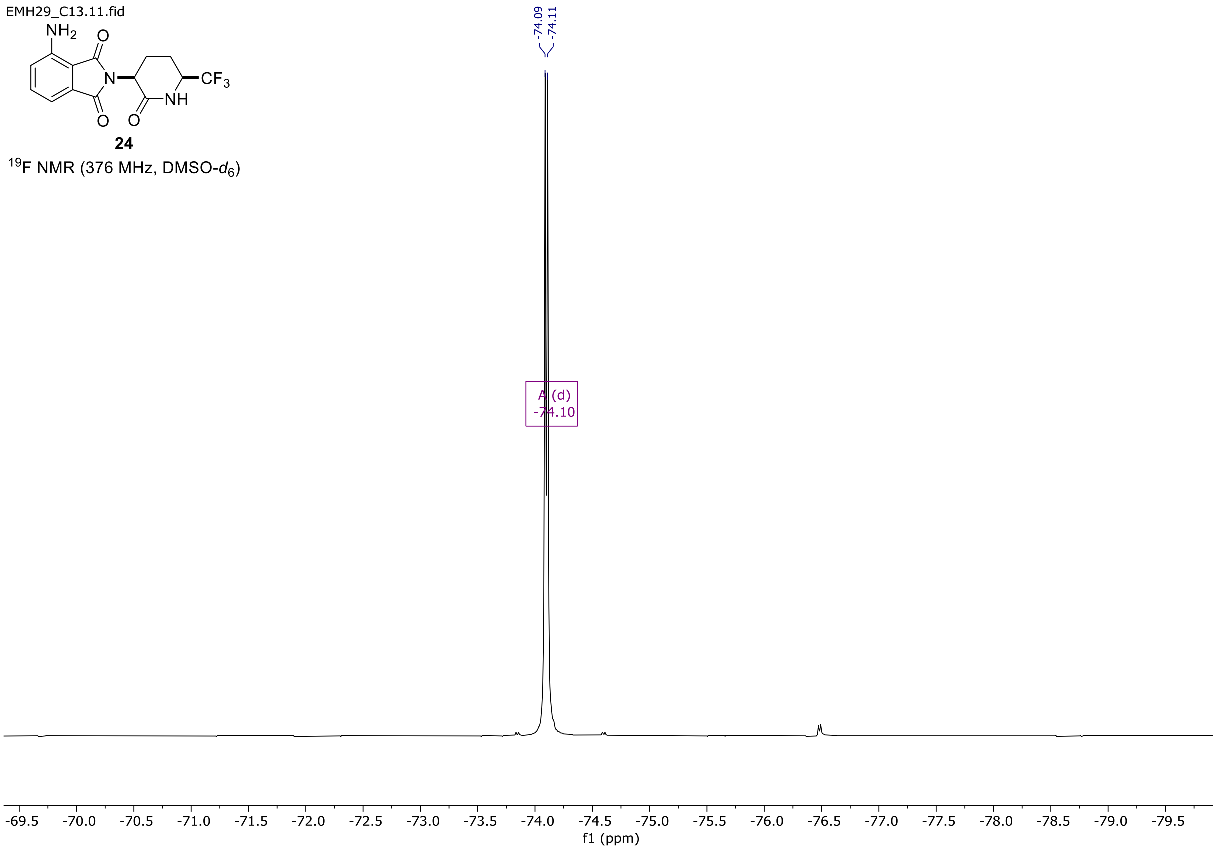

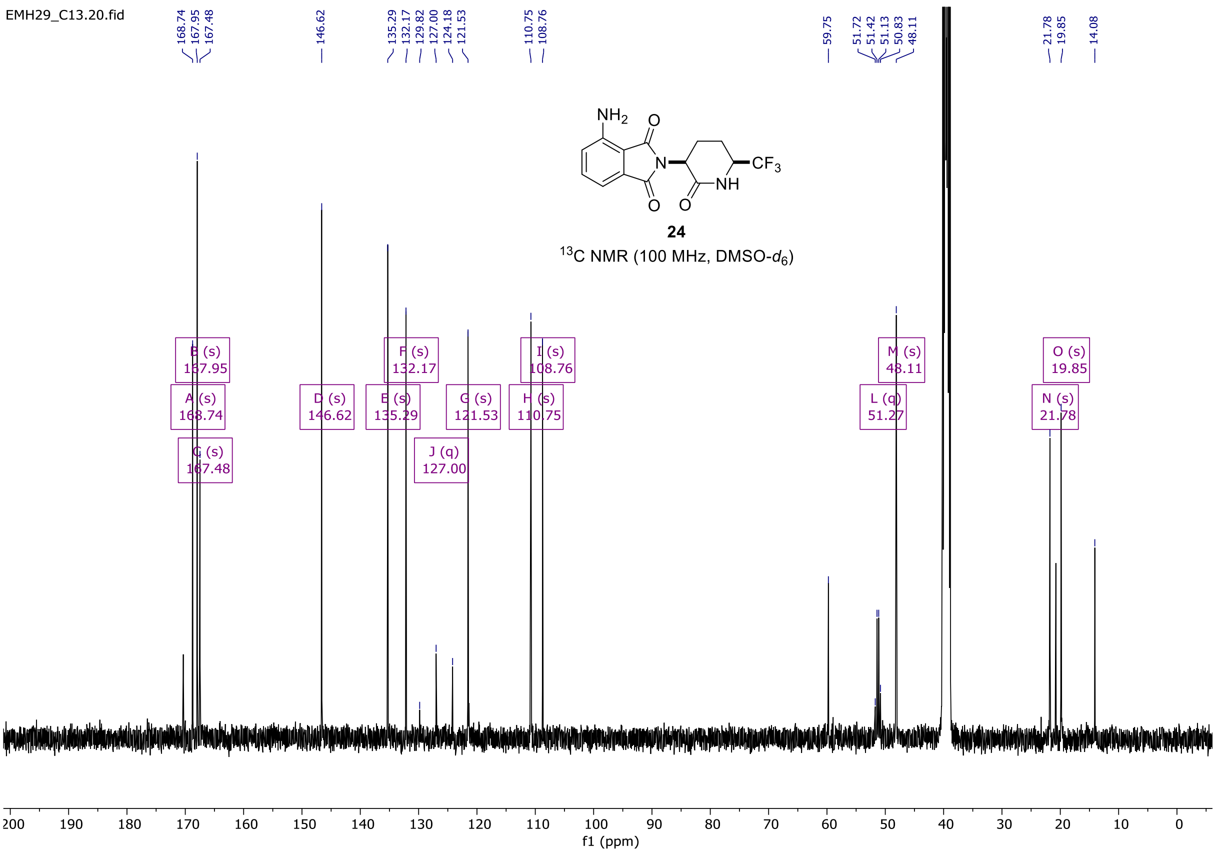
EMH29_C13.20.fid
168.74
167.95
167.48
146.62
135.29
132.17
129.82
127.00
124.18
121.53
110.75
108.76
59.75
51.72
51.42
51.13
50.83
48.11
21.78
19.85
14.08
NH2
O
N
CF3
NH
O
O
24
13C NMR (100 MHz, DMSO-d6)
A (s) 168.74
B (s) 167.95
C (s) 167.48
D (s) 146.62
E (s) 135.29
F (s) 132.17
G (s) 121.53
H (s) 110.75
I (s) 108.76
J (q) 127.00
L (q) 51.27
M (s) 48.11
N (s) 21.78
O (s) 19.85
200
190
180
170
160
150
140
130
120
110
100
90
80
70
60
50
40
30
20
10
0
f1 (ppm)

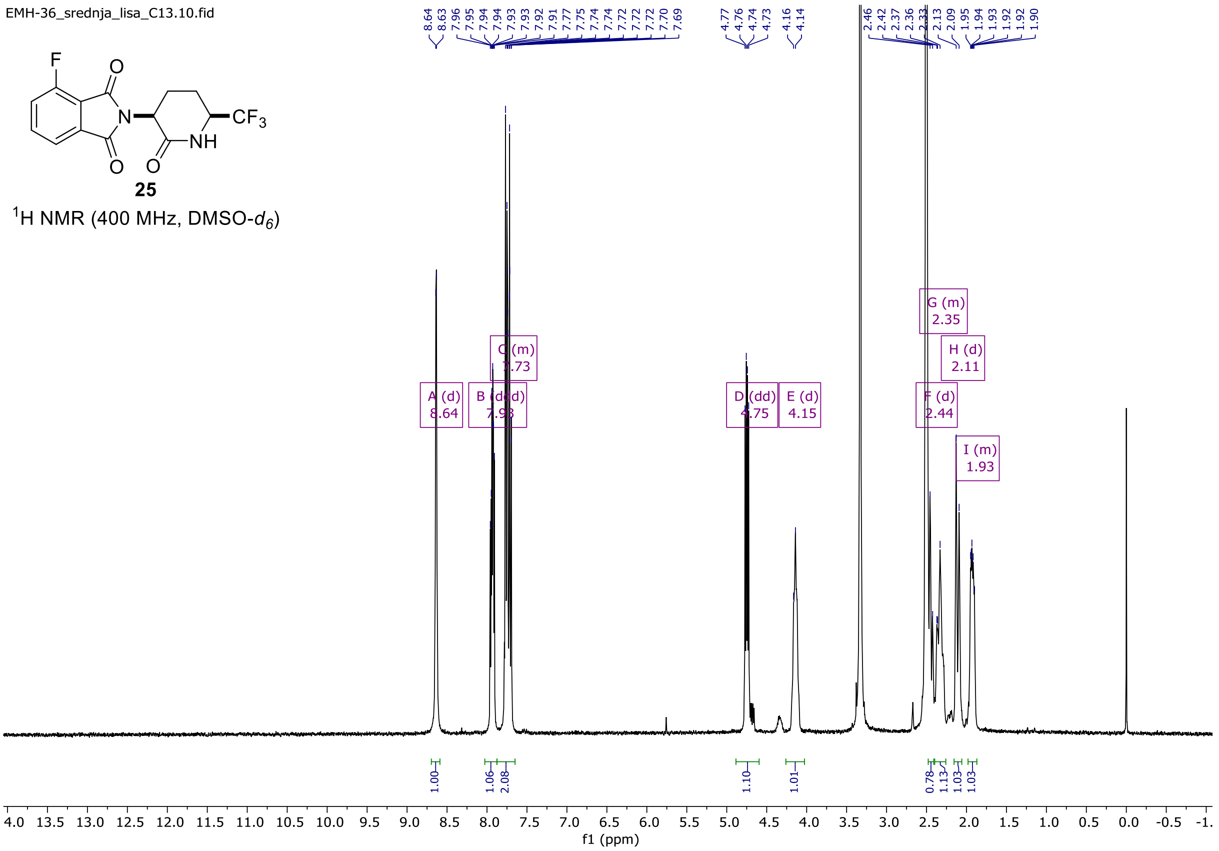
EMH-36_srednja_lisa_C13.10.fid
F
O
N
CF3
NH
O
O
25
1H NMR (400 MHz, DMSO-d6)
A (d)
8.64
B (ddd)
7.93
C (m)
7.73
D (dd)
4.75
E (d)
4.15
F (d)
2.44
G (m)
2.35
H (d)
2.11
I (m)
1.93
f1 (ppm)

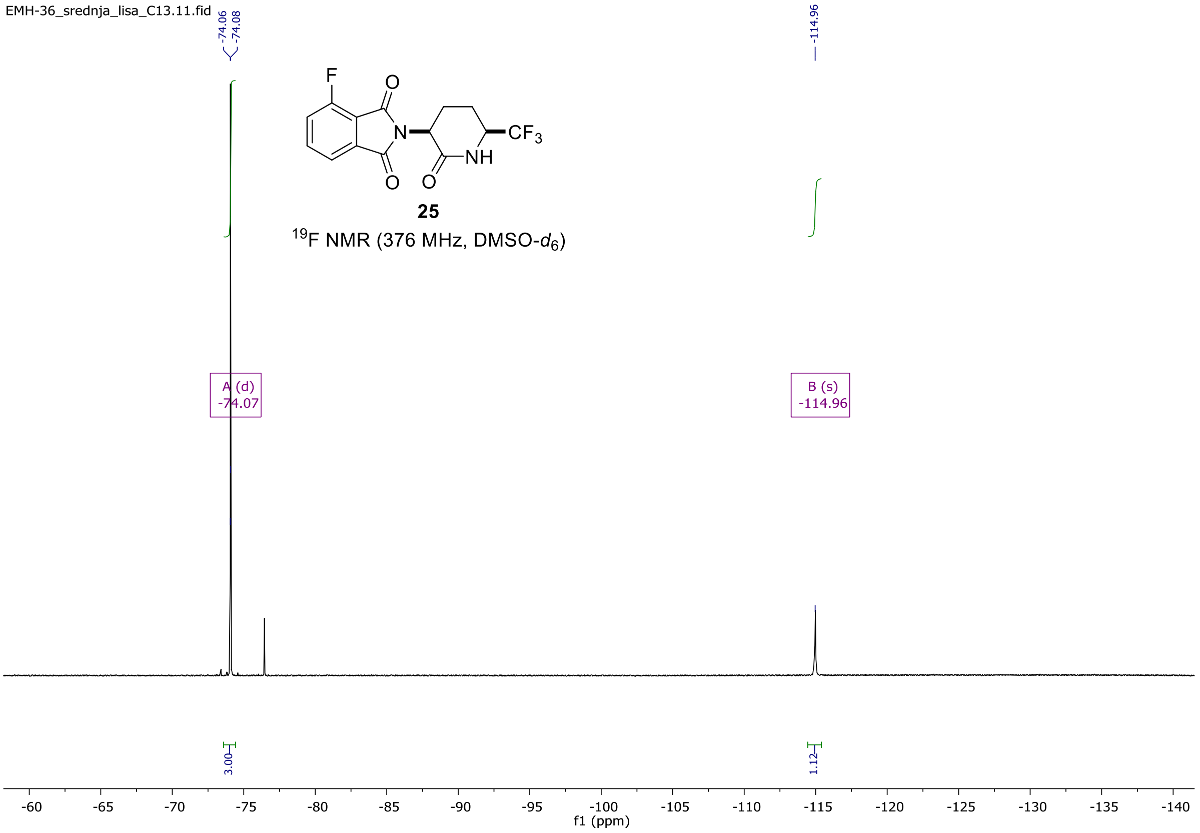
EMH-36_srednja_lisa_C13.11.fid
-74.06
-74.08
-114.96
F
O
N
CF3
NH
O
O
25
19F NMR (376 MHz, DMSO-d6)
A (d)
-74.07
B (s)
-114.96
3.00
1.12
f1 (ppm)

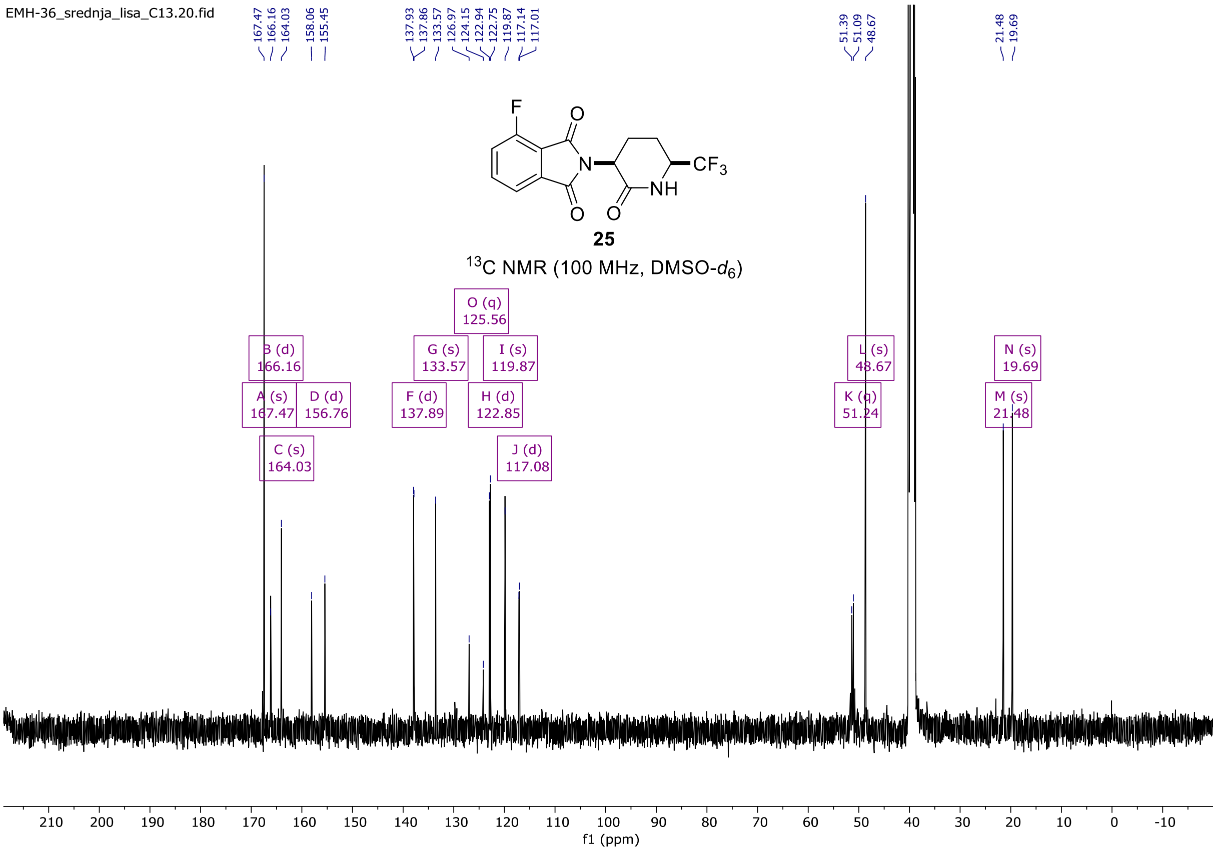
EMH-36_srednja_lisa_C13.20.fid
25
^{13}C NMR (100 MHz, DMSO-d_6)
A (s) 167.47
B (d) 166.16
C (s) 164.03
D (d) 156.76
F (d) 137.89
G (s) 133.57
O (q) 125.56
H (d) 122.85
I (s) 119.87
J (d) 117.08
K (q) 51.24
L (s) 48.67
M (s) 21.48
N (s) 19.69
f1 (ppm)

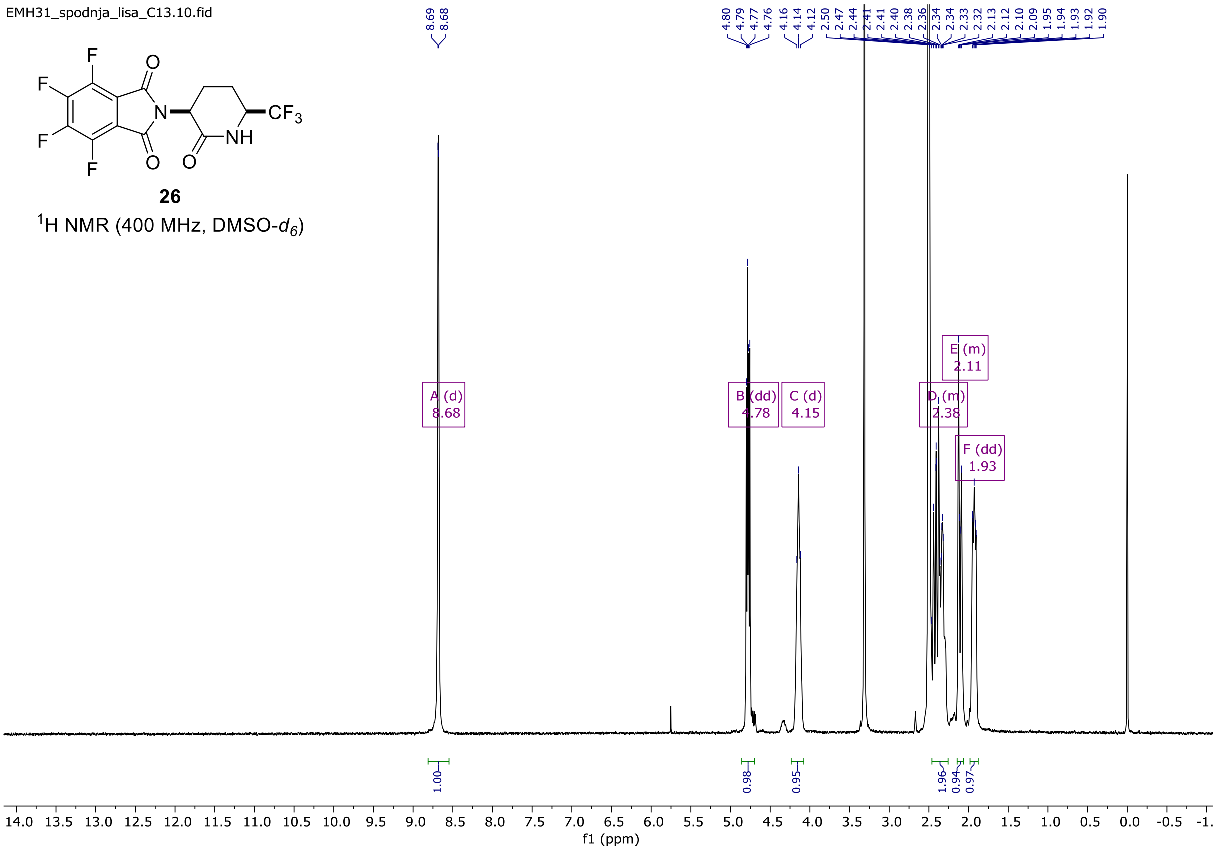
EMH31_spodnja_lisa_C13.10.fid
26
^{1}H NMR (400 MHz, DMSO-d_6)
A (d) 8.68
B (dd) 4.78
C (d) 4.15
D (m) 2.38
E (m) 2.11
F (dd) 1.93
f1 (ppm)

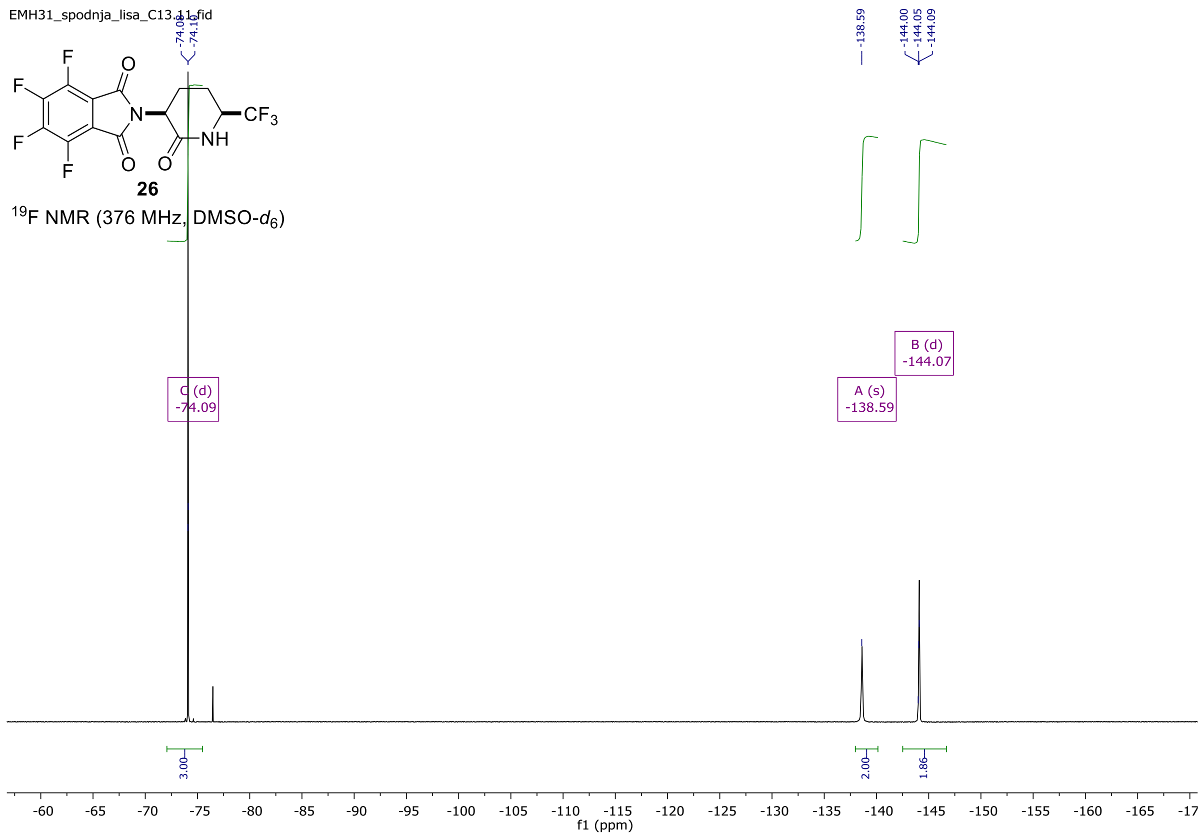
EMH31_spodnja_lisa_C13.11.fid
26
^{19}F NMR (376 MHz, DMSO-d_6)
-74.08
-74.10
-138.59
-144.00
-144.05
-144.09
C (d) -74.09
A (s) -138.59
B (d) -144.07
3.00
2.00
1.86
f1 (ppm)

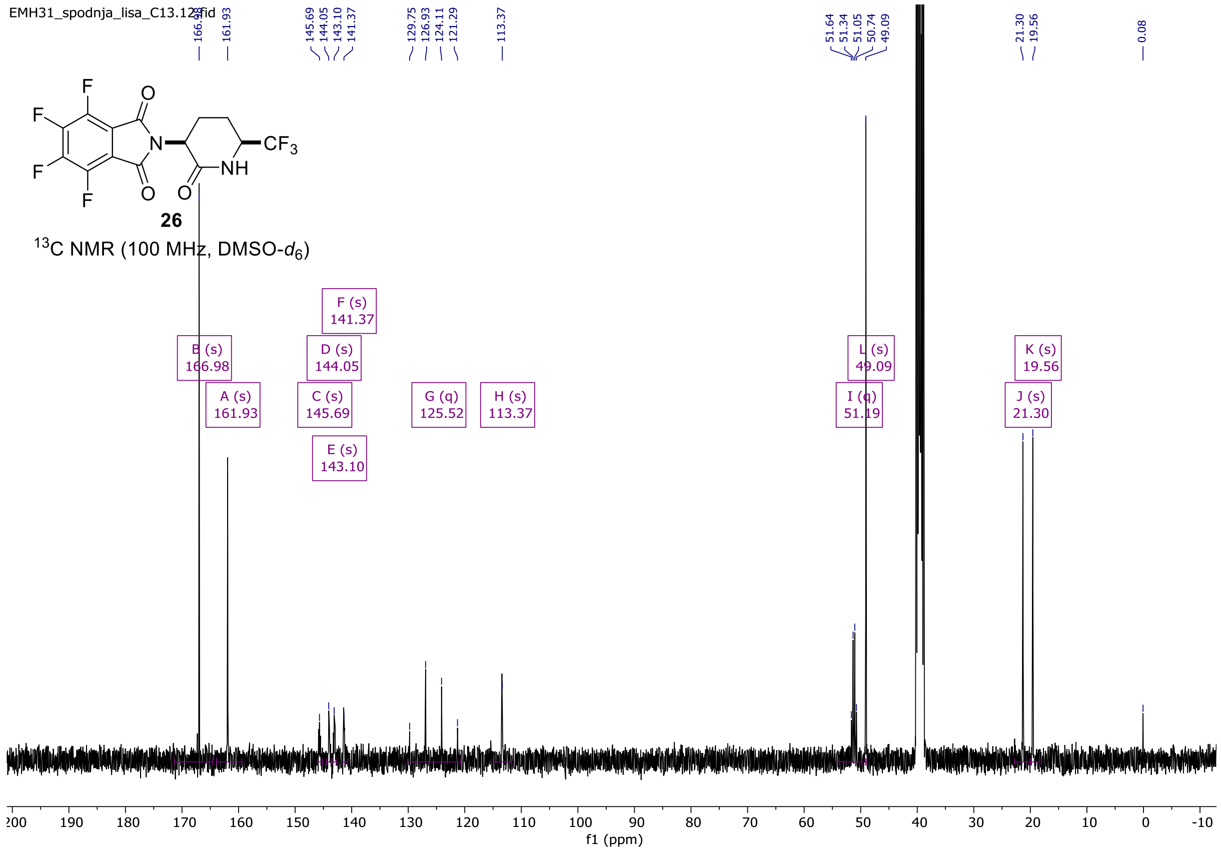
EMH31_spodnja_lisa_C13.12.fid
26
^{13}C NMR (100 MHz, DMSO-d_6)
166.98
161.93
145.69
144.05
143.10
141.37
129.75
126.93
124.11
121.29
113.37
51.64
51.34
51.05
50.74
49.09
21.30
19.56
0.08
B (s) 166.98
A (s) 161.93
C (s) 145.69
D (s) 144.05
E (s) 143.10
F (s) 141.37
G (q) 125.52
H (s) 113.37
I (q) 51.19
L (s) 49.09
J (s) 21.30
K (s) 19.56
f1 (ppm)

2D NMR spectra

Compound 20

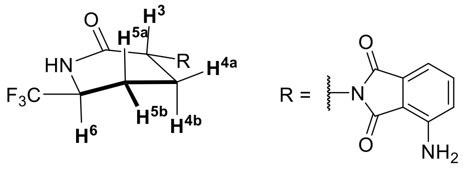

^{1}H–^{1}H COSY

In the COSY spectrum, cross-peaks indicate scalar couplings ($^3J_{HH}$) between the following pairs of protons: H3–H4a, H3–H4b, H6–H5a, H6–H5b, H5a–H4b, H5a–H4b, H4a–H5b, H4b–H5b; and geminal coupling ($^2J_{HH}$) between H5a–H5b, H4a–H4b.

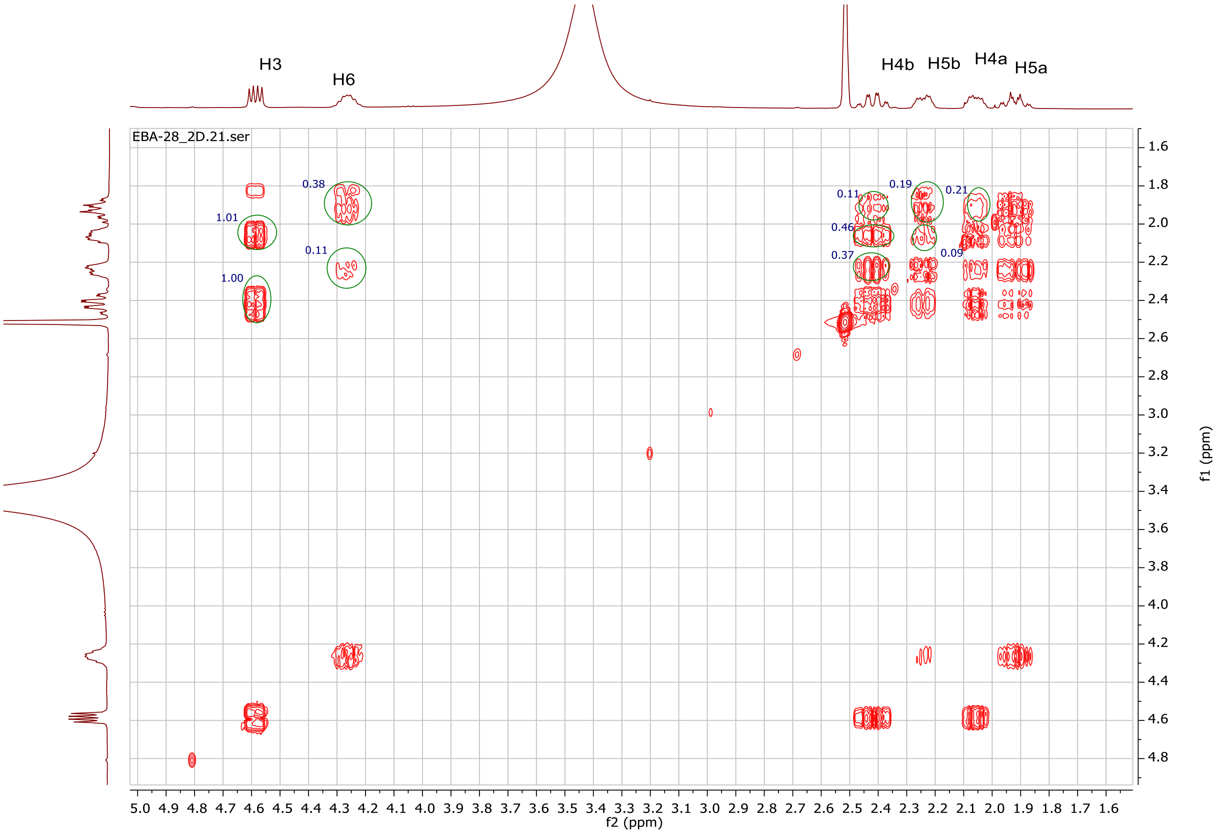

^{1}H–^{13}C HSQC

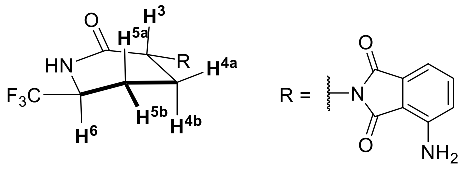

In the HSQC spectrum, H3–C3, H6–C6, H4b-C4, H5b-C5, H4a-C4 and H5a-C5 correlations confirm that these protons are directly attached to their respective carbons.

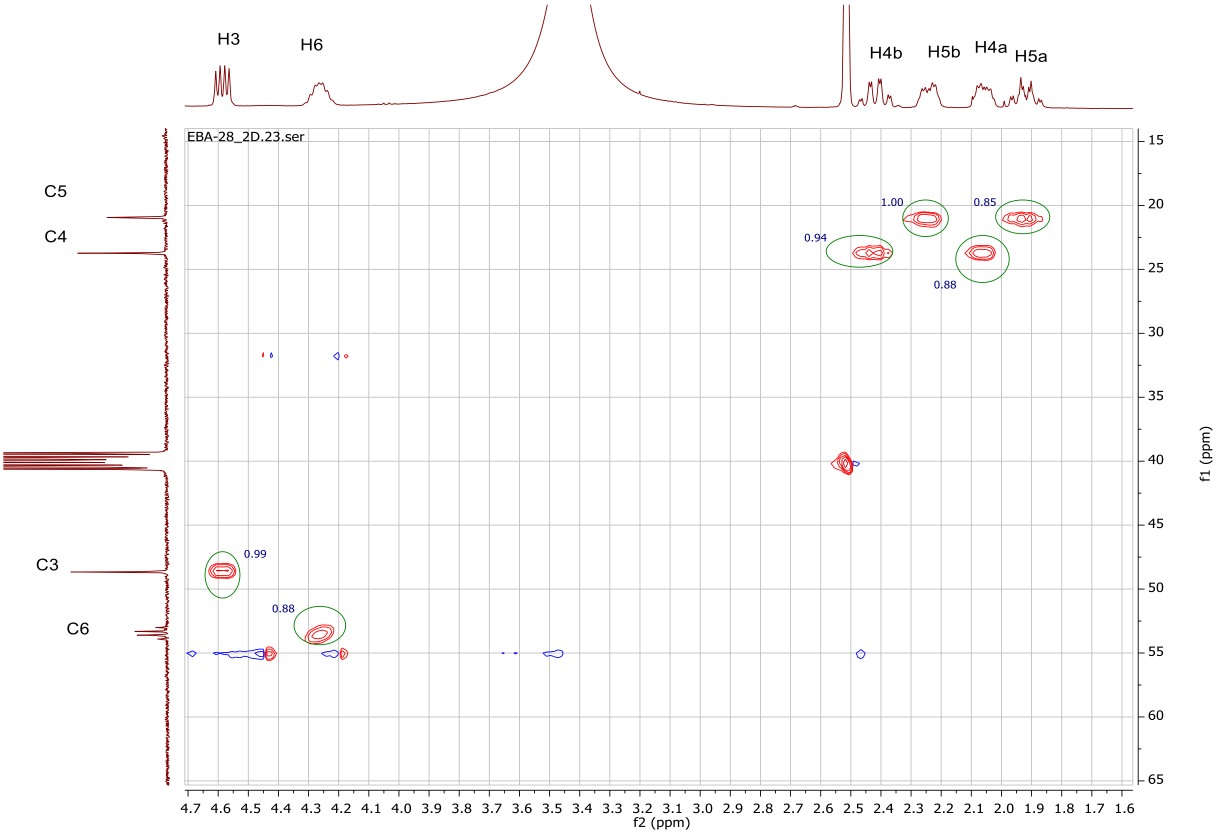

^{1}H–^{1}H NOESY

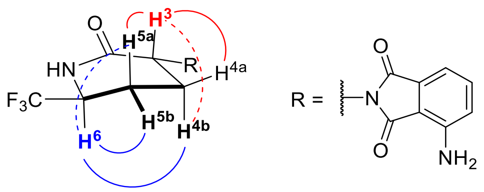

A strong nuclear Overhauser effect (nOe) is observed between H6 and H5b, and a weak nOe between H6 and its other vicinal proton, H5a. A strong nOe is observed between H6 and H4b. On the other hand, a weak nOe is observed between H3 and H4b, and a strong nOe between H3 and its other vicinal proton H4a, and with H5a. This confirms the *trans* relative configuration of the protons H3 and H6 at the chiral centers.

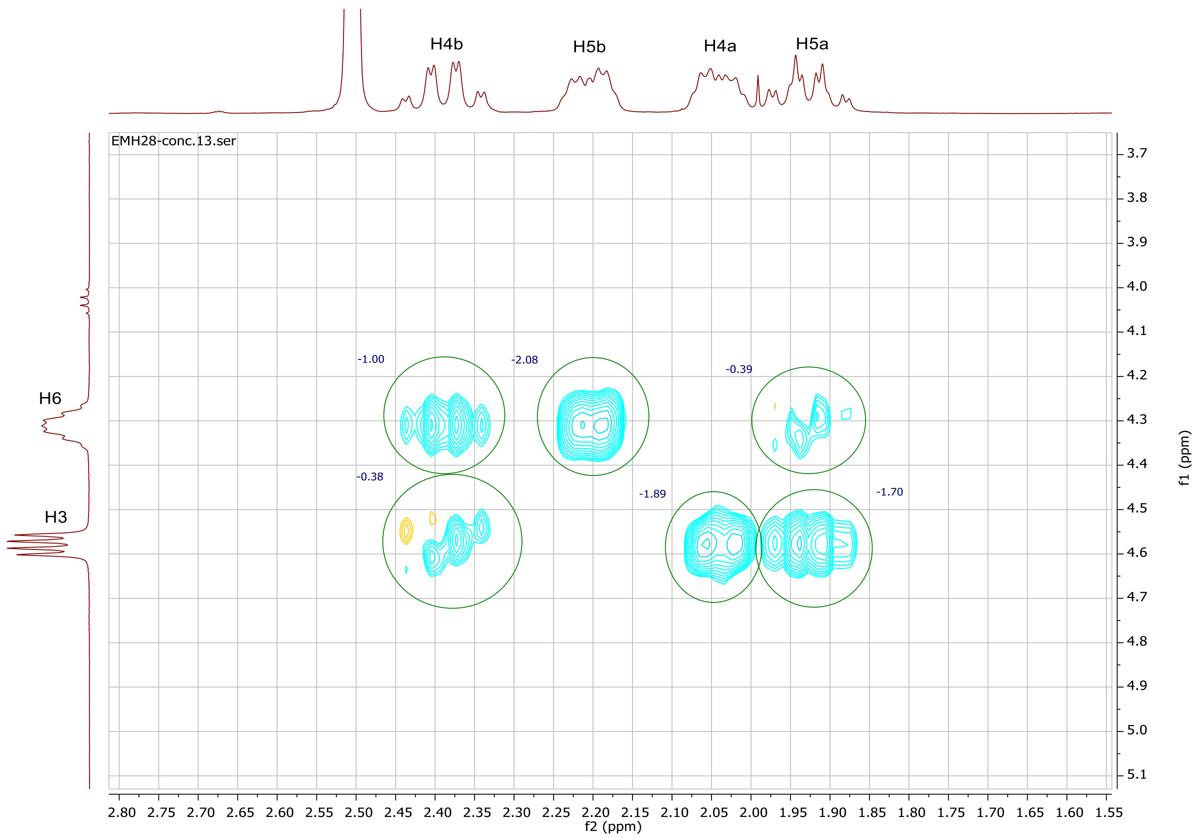

^{1}H–^{19}F HOESY

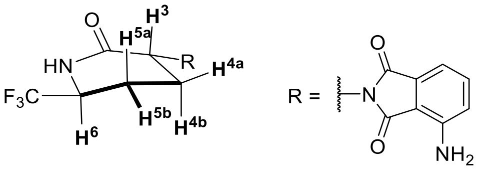

A strong correlation is observed between CF_3 and H6, CF_3 and H5a and a less intense correlation between CF_3 and H5b. There is also weak nOe between CF_3 and H3 and CF_3 and H4a. The presence of a weak CF_3-H3 correlation and the lack of CF_3-H4b correlation additionally confirms the *trans* relative configuration.

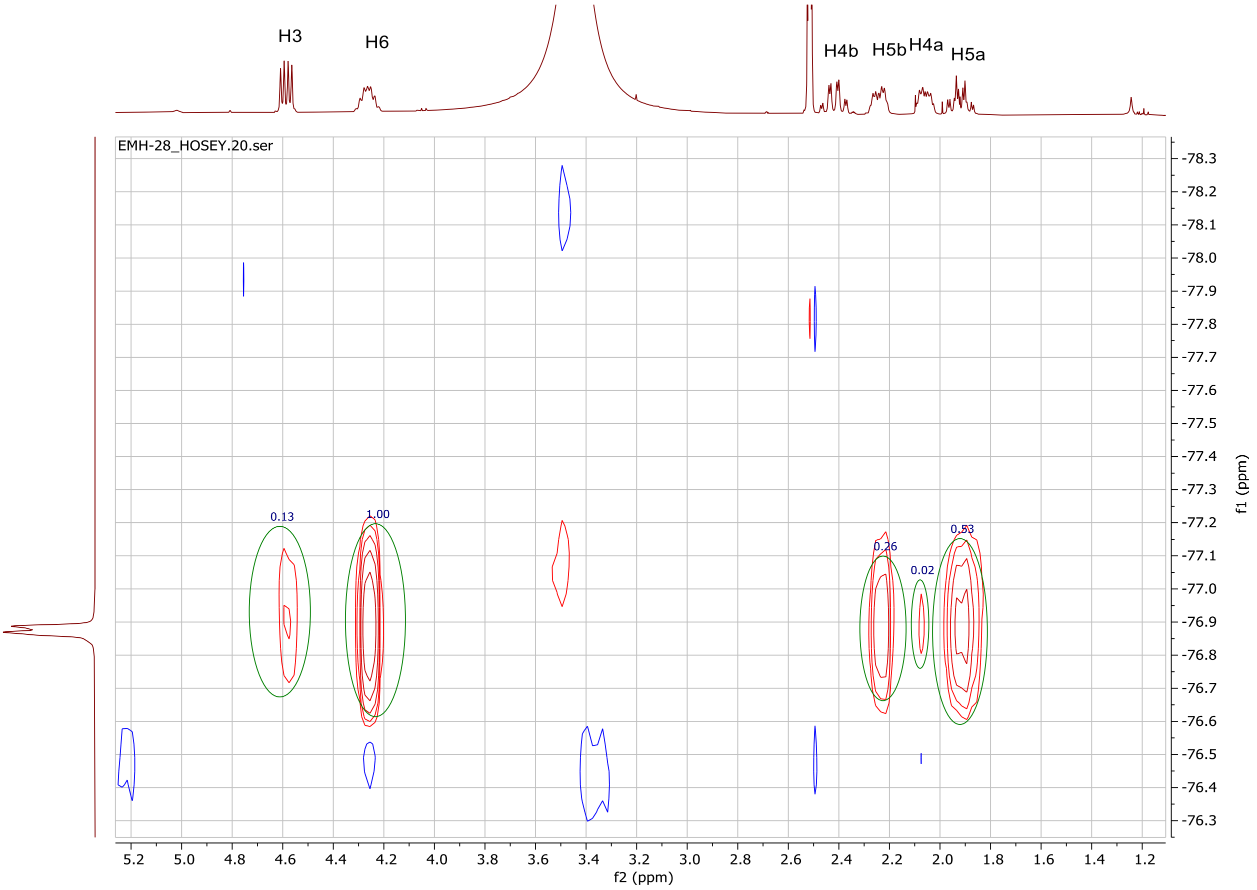

Compound 24

^1H–^1H COSY

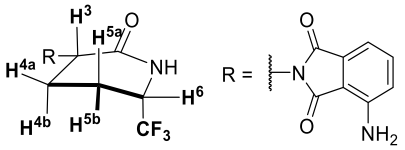

In the COSY spectrum, cross-peaks indicate scalar couplings ($^3J_{HH}$) between the following pairs of protons: H3–H4a, H3–H4b, H6–H5a, H6–H5b, H4a–H5a, H4a–H5b, H5b–H4b, H5a–H4b, and geminal couplings H4a–H4b, H5b–H5a.

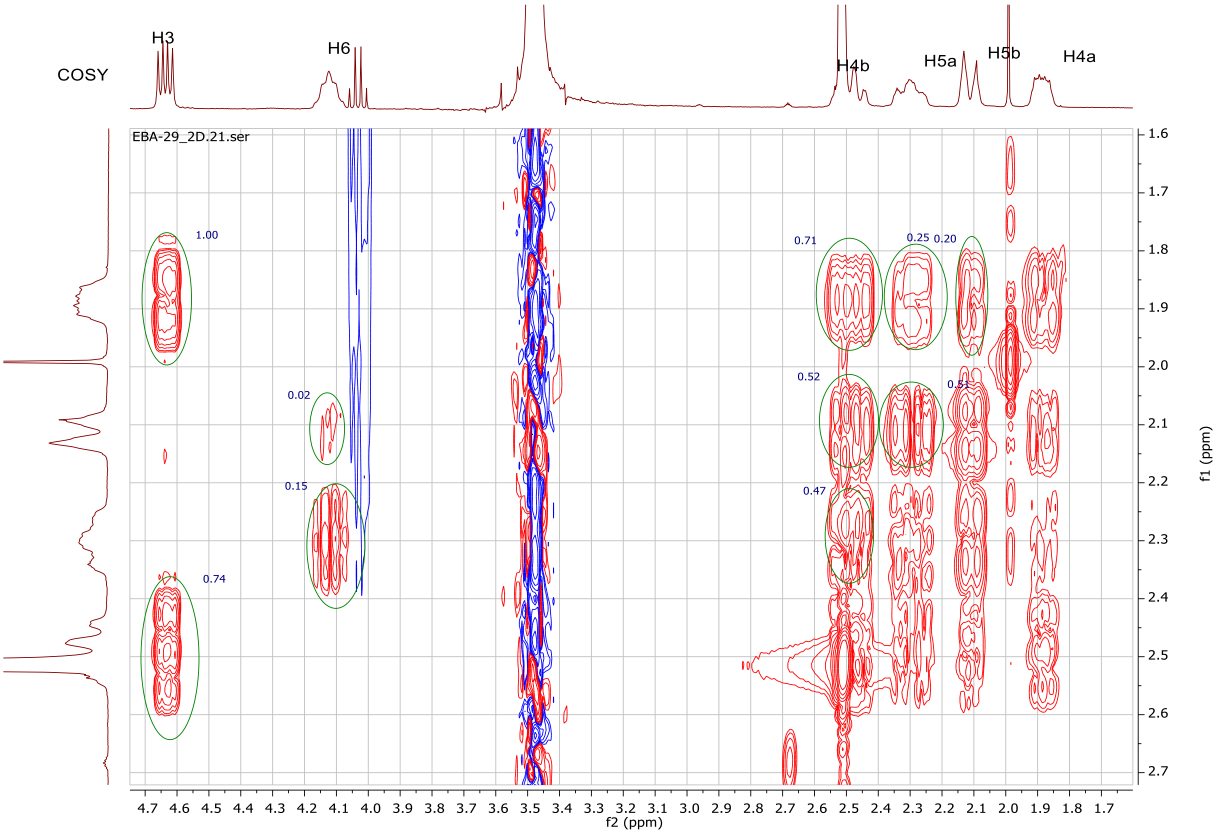

$^{1}H–^{13}C$ HSQC

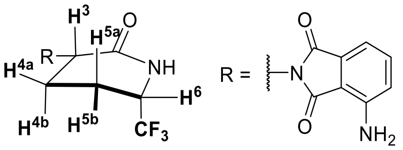

In the HSQC spectrum, H3–C3, H6–C6, H4b-C4, H5b-C5, H4a-C4 and H5a-C5 correlations confirm that these protons are directly attached to their respective carbons.

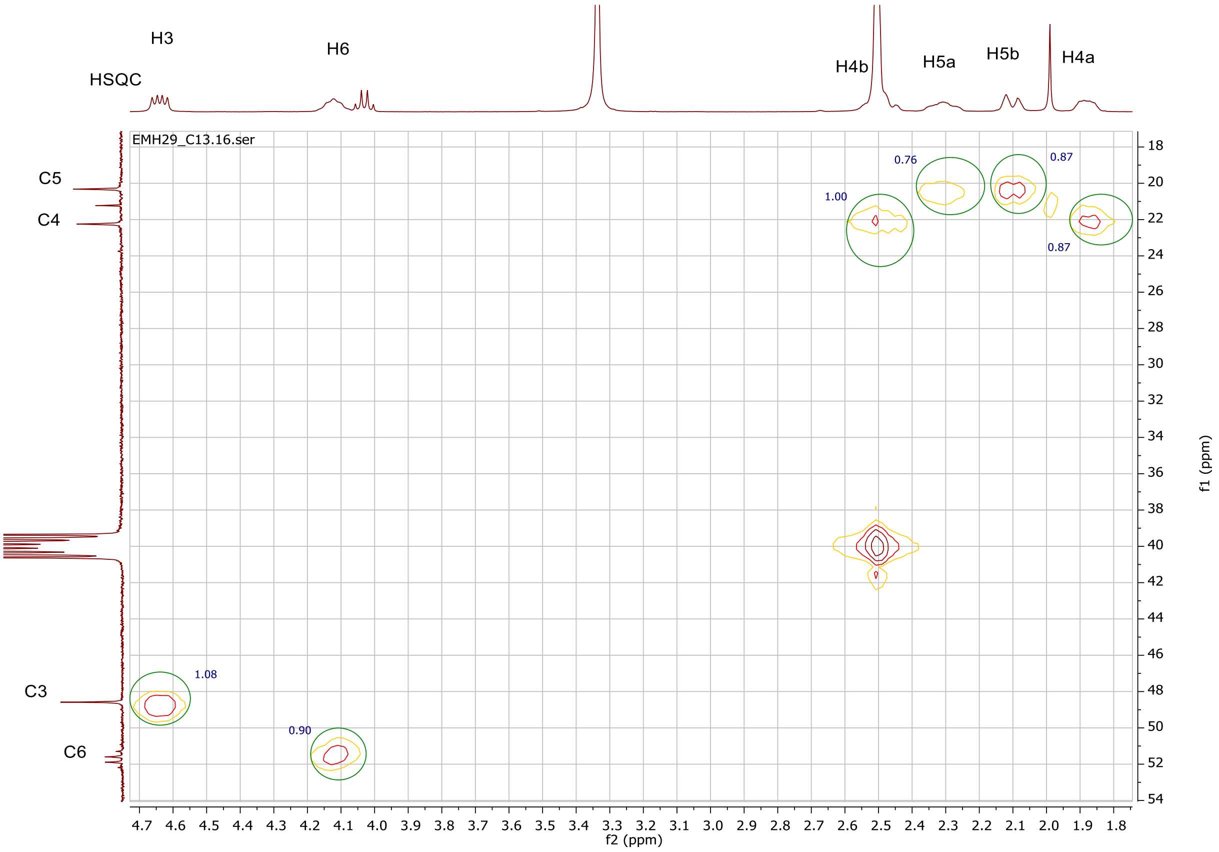

^{1}H–^{1}H NOESY

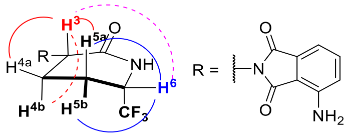

A strong nOe is observed between H6 and H5b, as well as between H6 and H5b, and in particular, a weak nOe appears between H6 and H3. On the other hand, a weak nOe is detected between H3 and H4b, along with two strong ones between H4a and H5a. This confirms the *cis* relative configuration of the protons H3 and H6 at the chiral centers.

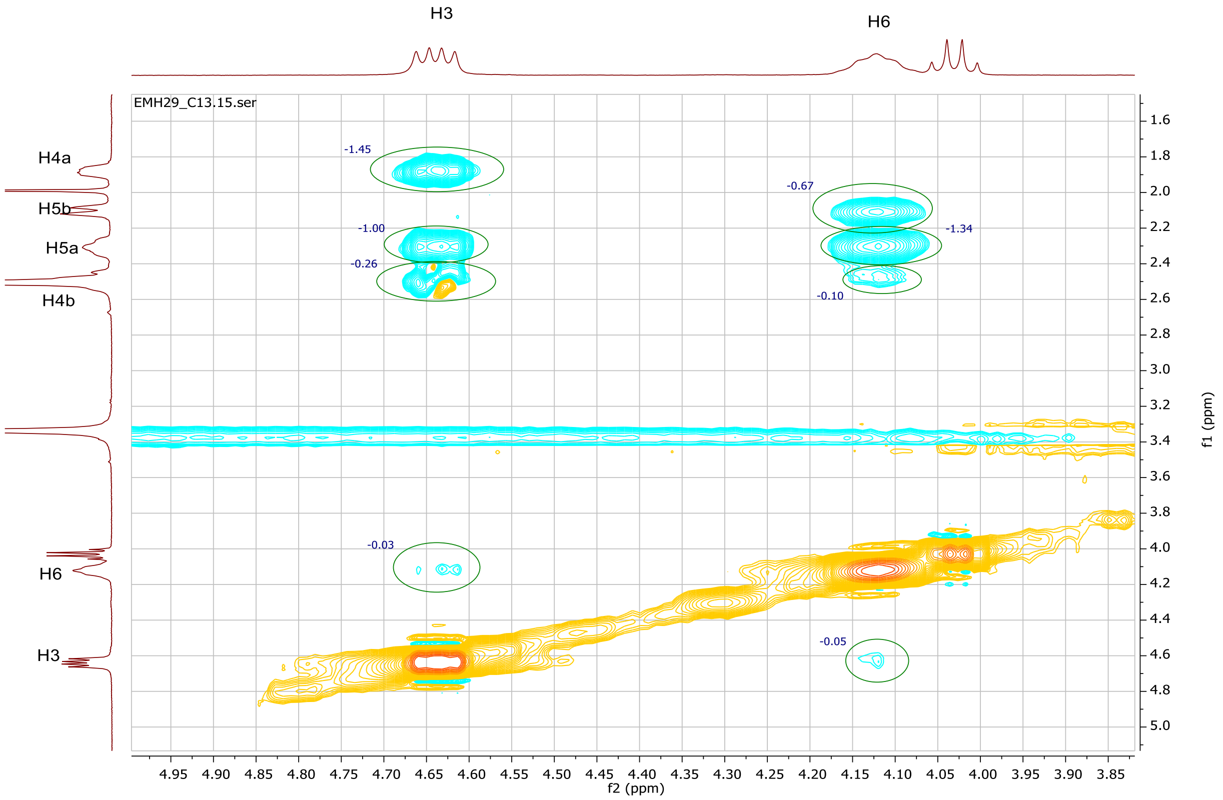

[1]H–[19]F HOESY

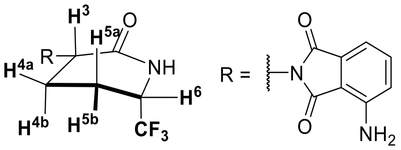

A strong correlation is observed between CF_3 and H6, CF_3 and H5b and CF_3 and H4b. There is also no correlation between CF_3 and H3, which confirms the *cis* relative configuration.

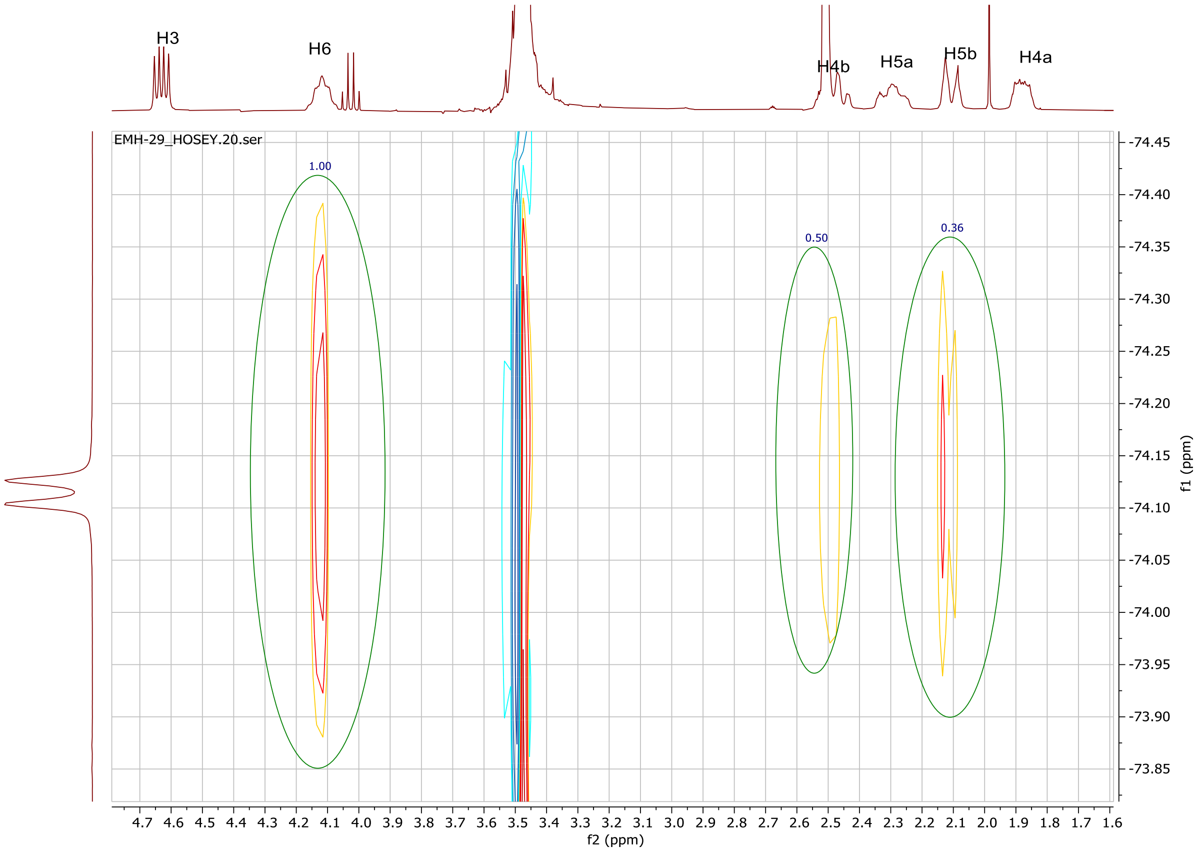

Compound 7

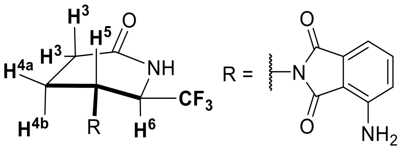

^{1}H–^{1}H COSY

In the COSY spectrum, crosspeaks indicate scalar couplings between the following pairs of protons:

H5–H4a, H5–H4b, H4a–H3a/b, H4a–H4b, and H4b–H3a/b. The NH–C6 crosspeak additionally establishes the regioisomeric structure.

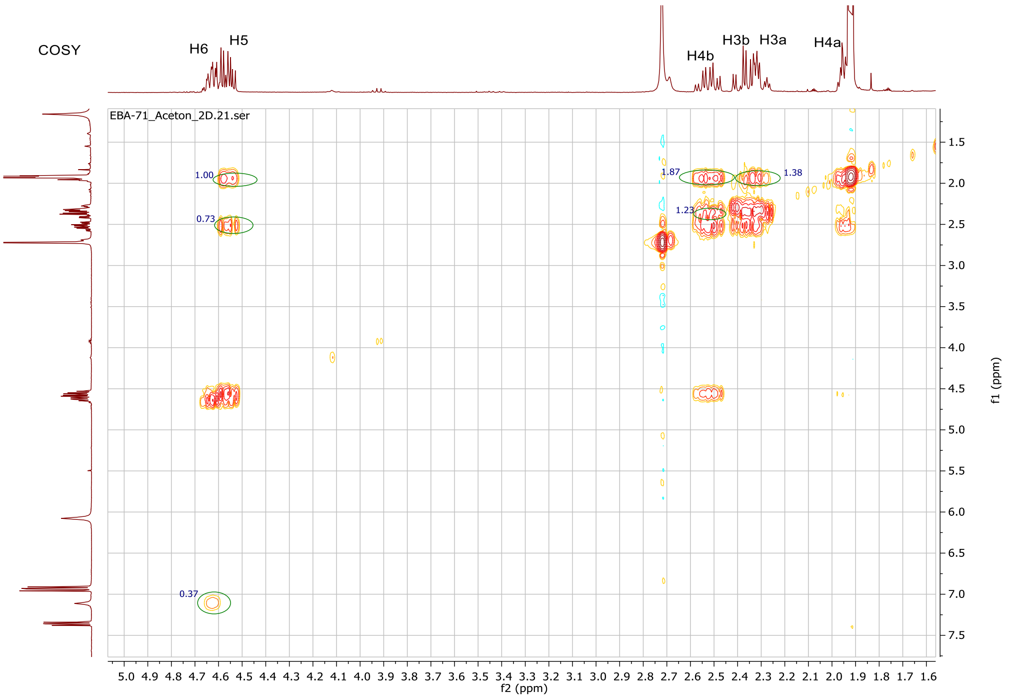

^{1}H–^{13}C HSQC

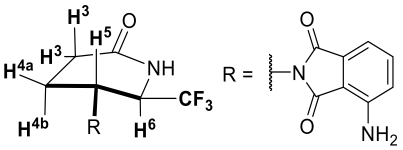

In the HSQC spectrum H6–C6, H5-C5, H4b–C4, H3b–C3, H3a–C3 and H4a-C4 correlations confirm that these protons are directly attached to their respective carbons. In particular, C5 and C6 were distinguished by the quartet pattern of C6.

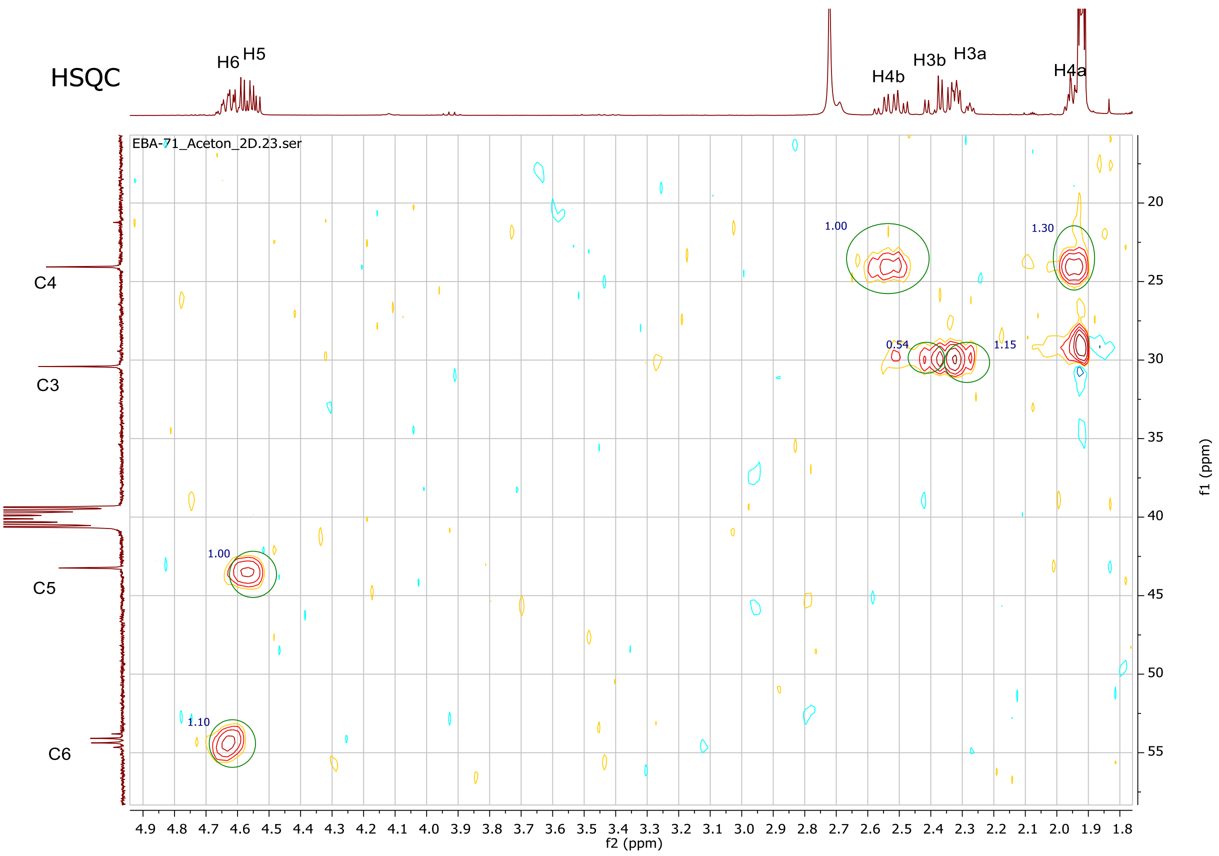

^{1}H–^{1}H NOESY

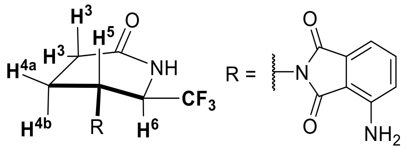

A nOe is observed between H5 and H4a, H4b and H3. Notably, the H5–H4a correlation is stronger than H5–H4a correlation, but this observation is compromised by the zero-quantum artefact. H6 exhibits a nOe only with NH. A lack of nOe between H5 and H6 is inconclusive due to low resolution of their ^{1}H NMR signals. These observations together with the ^{1}H–^{19}F HOESY spectrum support the *trans* relative configuration if the spectral data are compared to the *cis* diastereomer below.

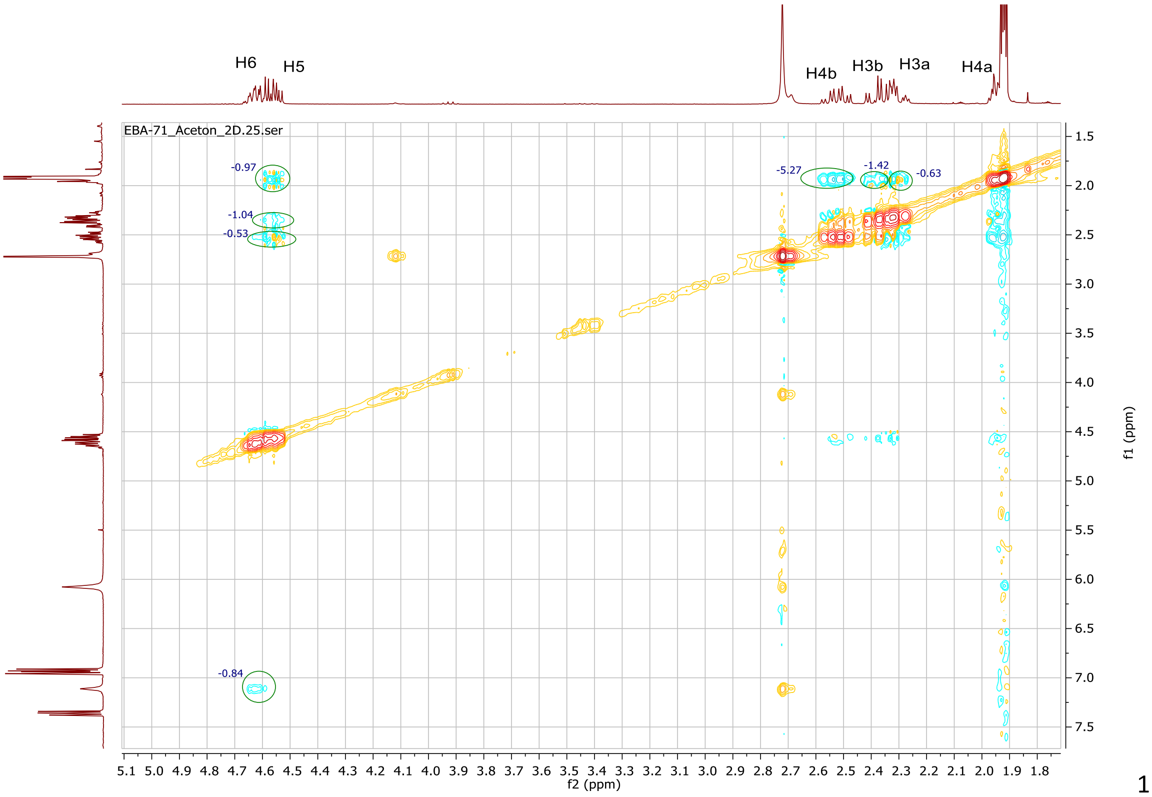

1H-

^{1}H–^{19}F HOESY

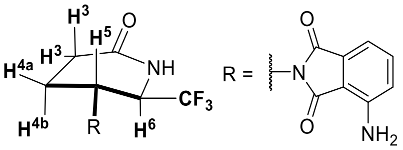

A strong correlation is observed between CF_3 and H5 and CF_3 and H6. There is also weak correlation between CF_3 H4a, and a lack of CF_3–H4b correlation. These observations, together with the above ^{1}H–^{1}H NOESY spectrum, support the *trans* relative configuration.

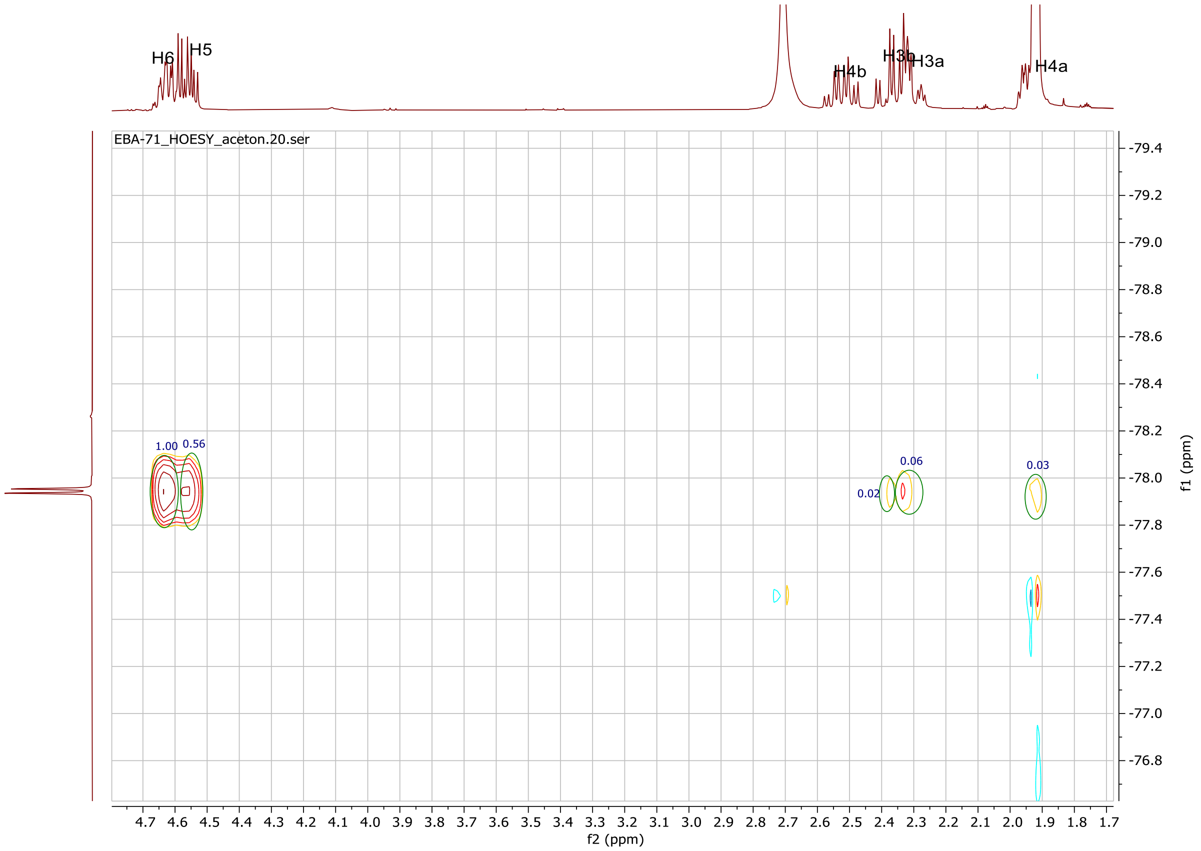

Compound 13

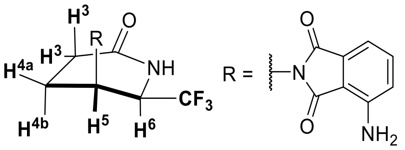

^{1}H–^{1}H COSY

In the COSY spectrum, cross-peaks indicate scalar couplings between the following pairs of protons:

H5–H4b, H5–H4a, H5–H6, H6–NH, H4b–H3, H4b–H4a, and H3–H4a. These observations confirm the indicated regioisomer.

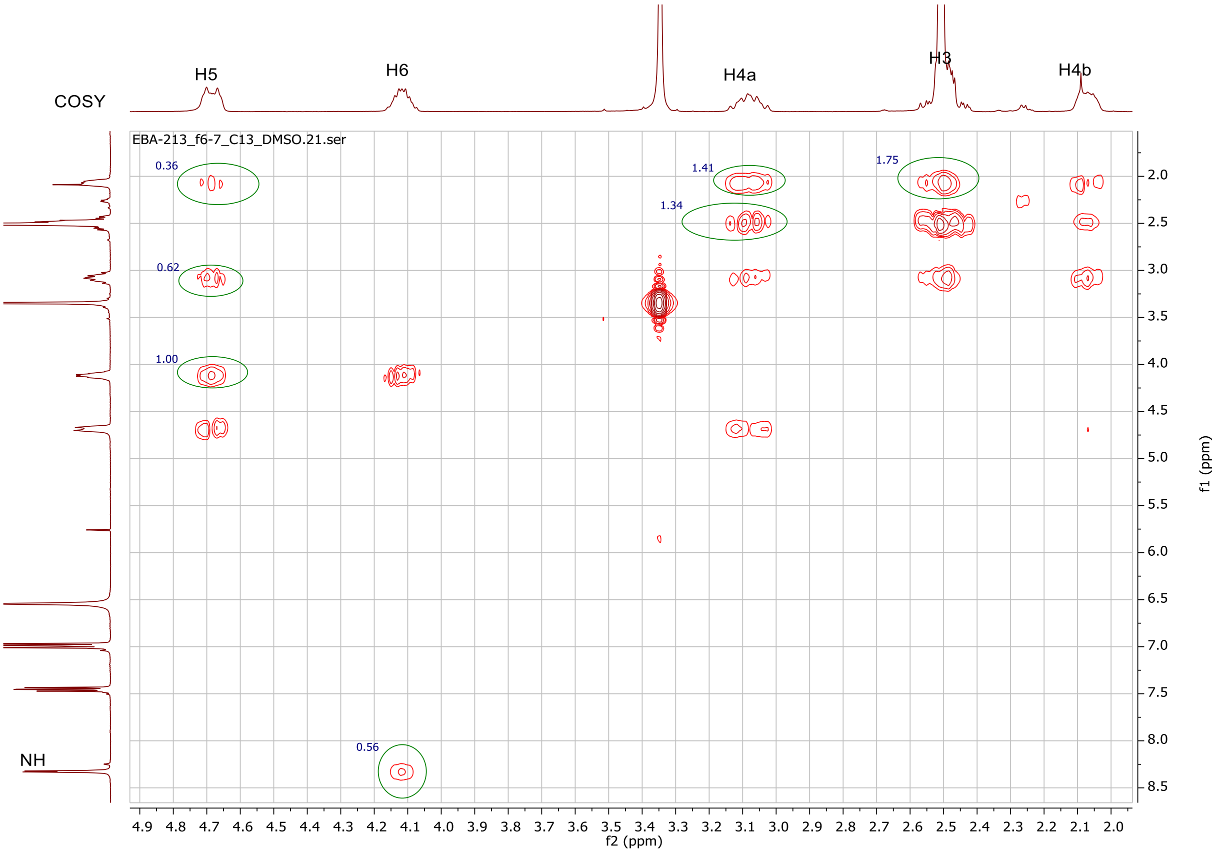

^{1}H–^{13}C HSQC

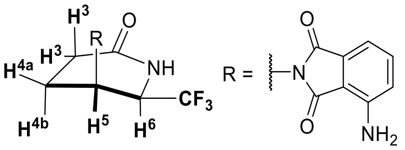

In the HSQC spectrum, H3–C3, H6–C6, H4b-C4, H4a–C4 and H5–C5 correlations confirm that these protons are directly attached to their respective carbons. In particular, C5 and C6 were distinguished by the quartet pattern of C6.

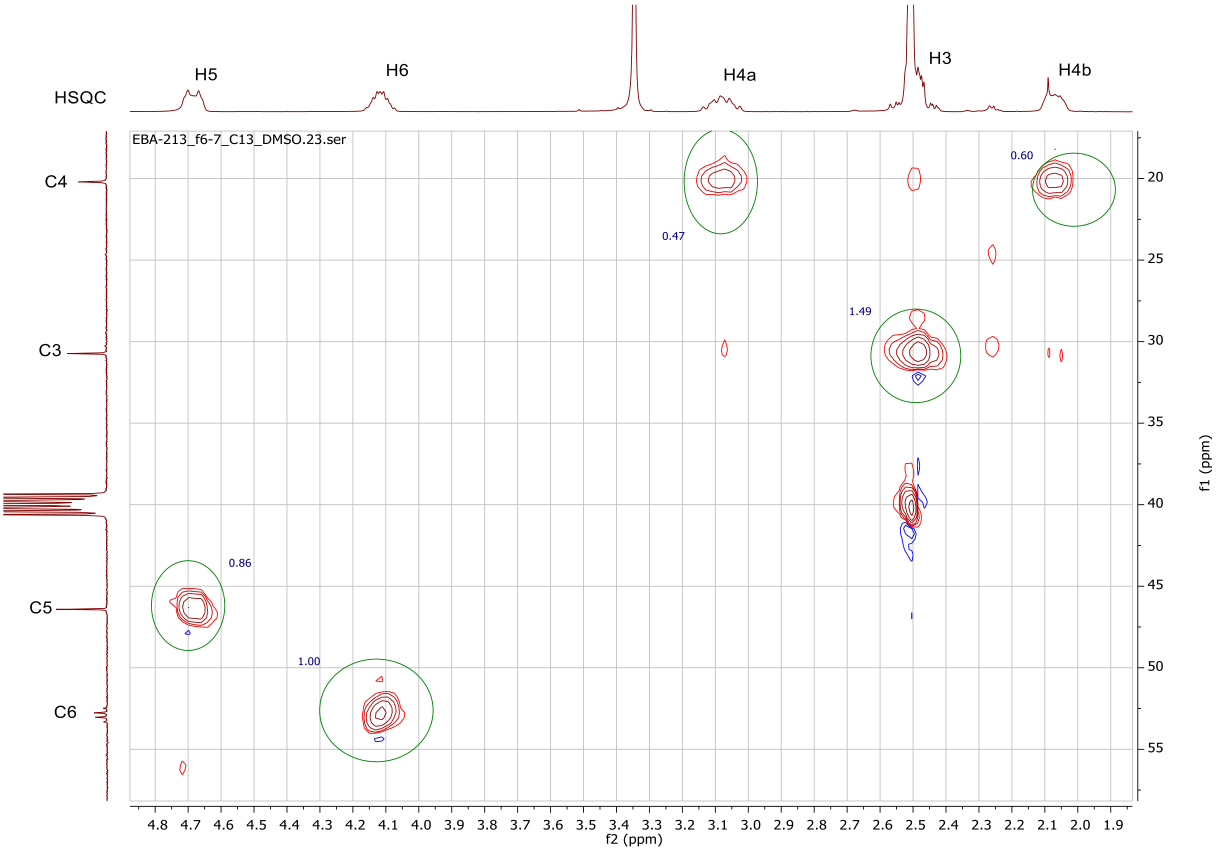

^1H–^1H NOESY

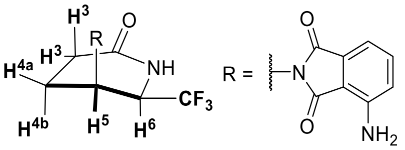

A strong nOe is observed between H5 and H6, indicating their *cis* relative configuration.

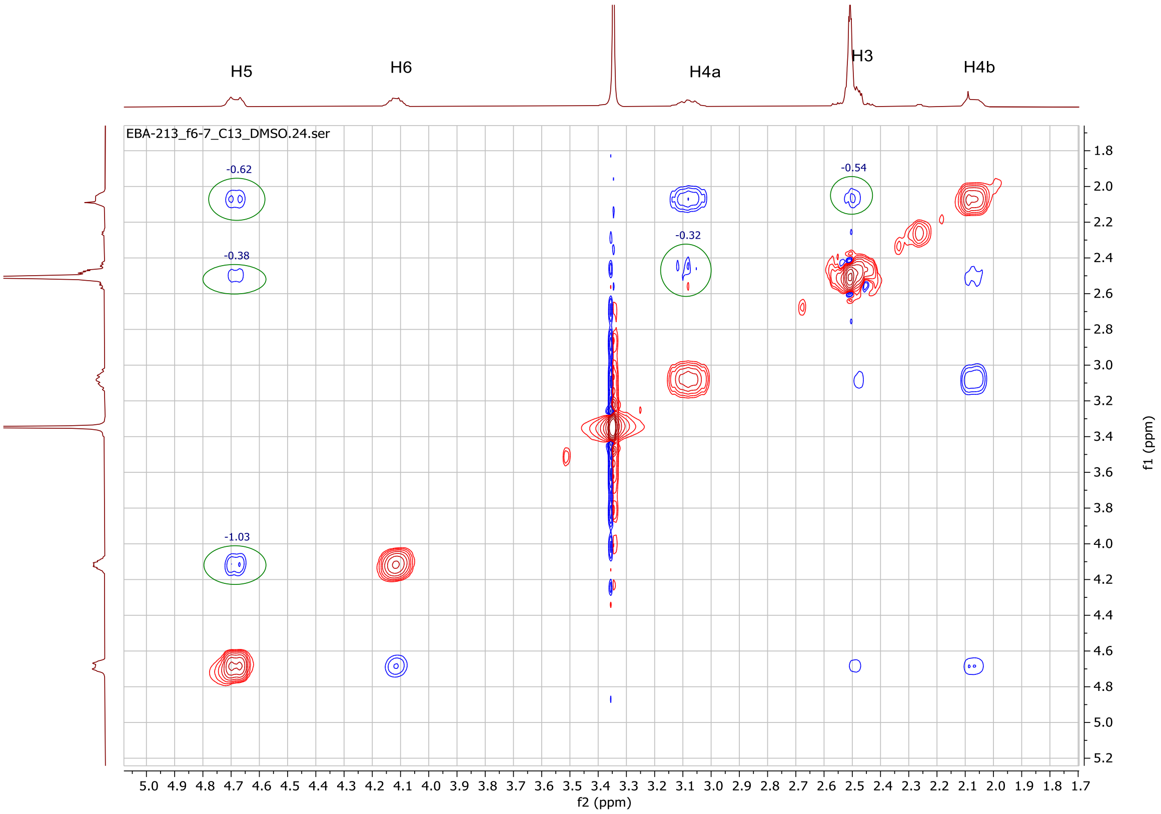

^1H–^{19}F HOESY

A strong correlation is observed between CF_3 and NH, CF_3 and H6, and CF_3 and H4a. Correlation between CF_3 and H5 is week which indicates their orientation on the opposite site of the ring, which is in agreement with the observations in the ^1H–^1H NOESY spectrum, and confirms the *cis* relative configuration.

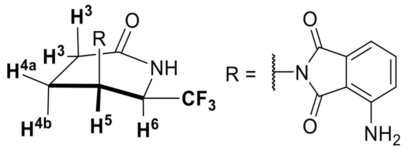

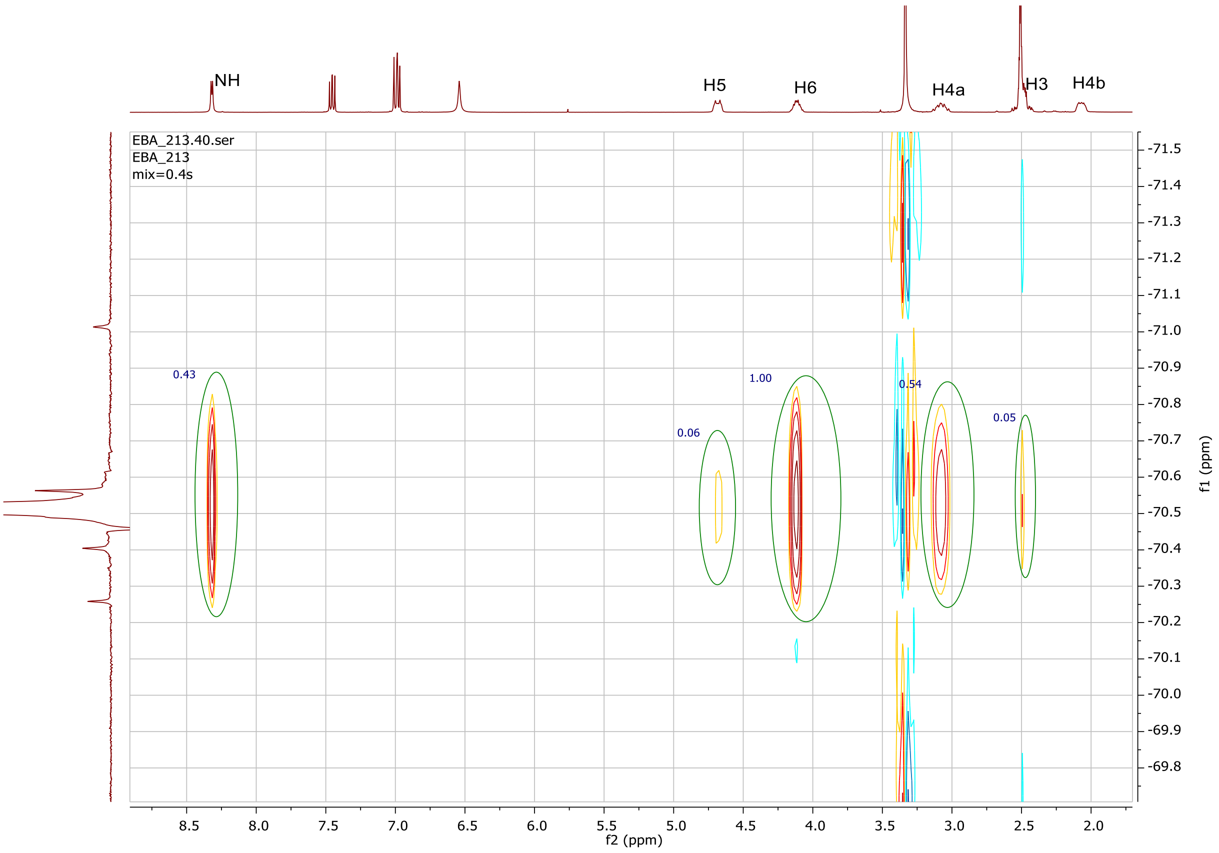

Compound 21

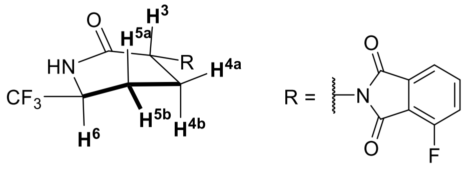

^{1}H–^{1}H COSY

In the COSY spectrum, cross-peaks indicate scalar couplings ($^3J_{HH}$) between the following pairs of protons: H3–H4a, H3–H4b, H6–H5a, H6–H5b, H5a–H4b, H5a–H4b, H4a–H5b, H4b–H5b; and geminal coupling ($^2J_{HH}$) between H5a–H5b, H4a–H4b.

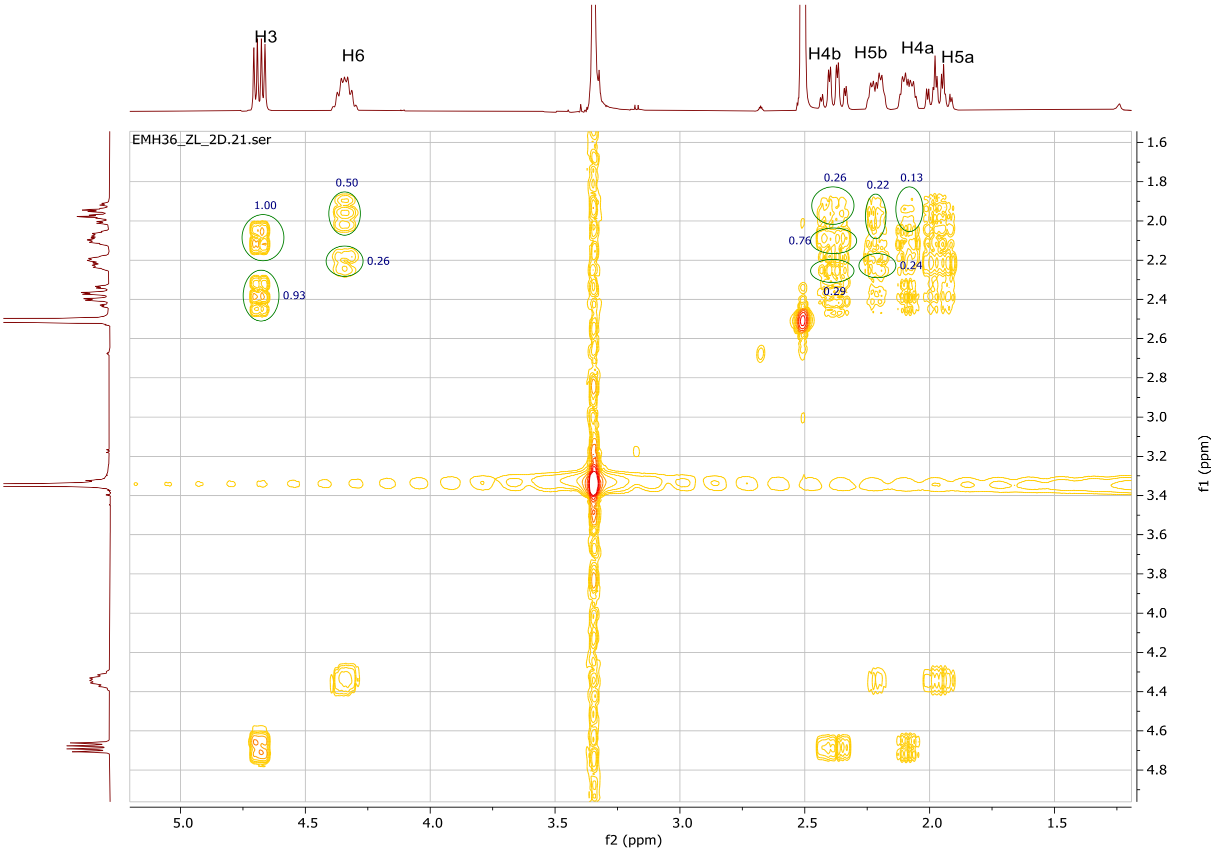

^{1}H–^{13}C HSQC

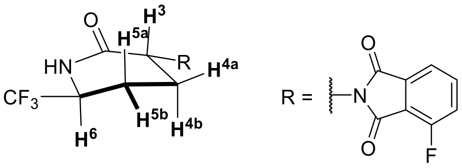

In the HSQC spectrum, H3–C3, H6–C6, H4b-C4, H5b-C5, H4a-C4 and H5a-C5 correlations confirm that these protons are directly attached to their respective carbons.

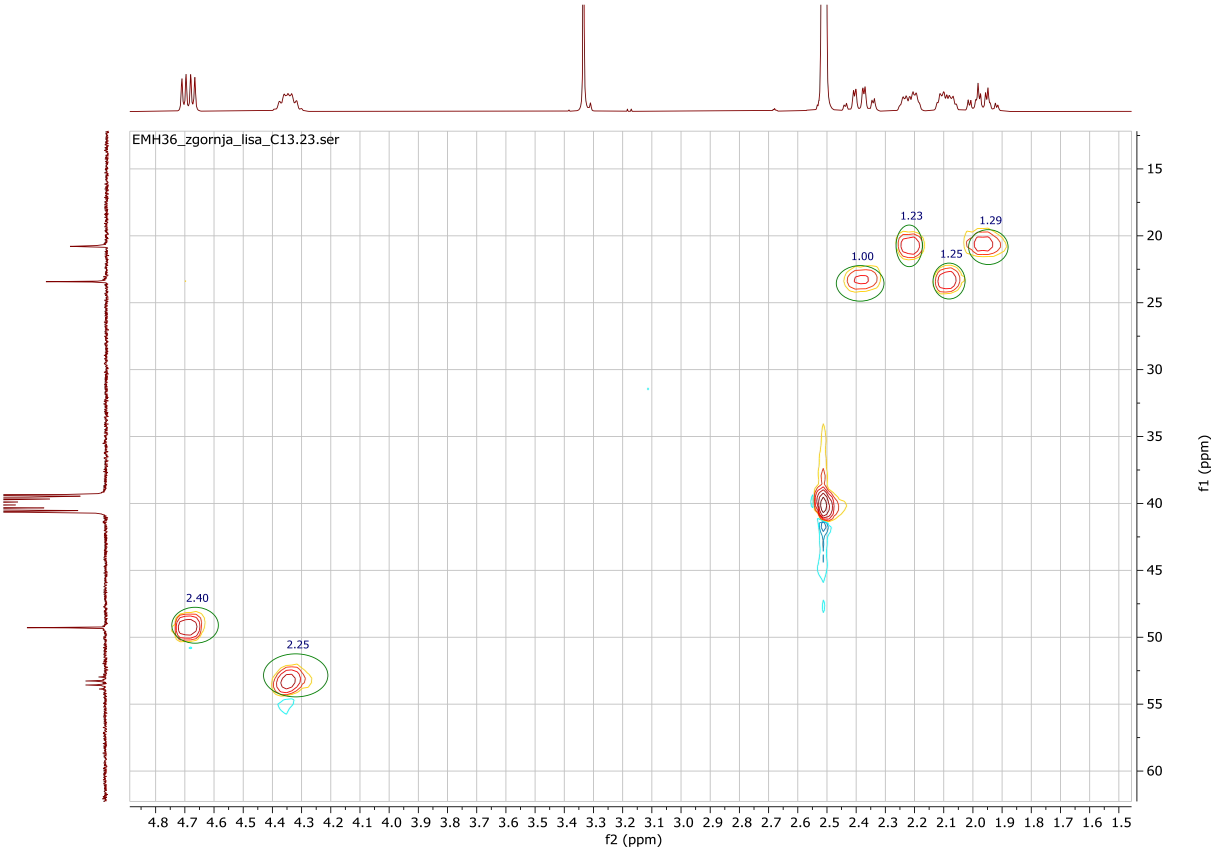

^1H–^1H NOESY

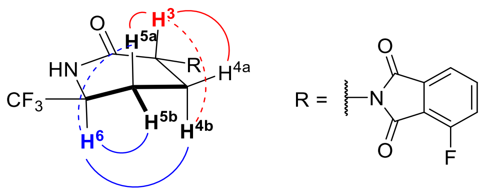

A strong nuclear Overhauser effect (nOe) is observed between H6 and H5b, and a weak nOe between H6 and its other vicinal proton, H5a. A strong nOe is observed between H6 and H4b. On the other hand, a weak nOe is observed between H3 and H4b, and a strong nOe between H3 and its other vicinal proton H4a, and with H5a. This confirms the *trans* relative configuration of the protons H3 and H6 at the chiral centers.

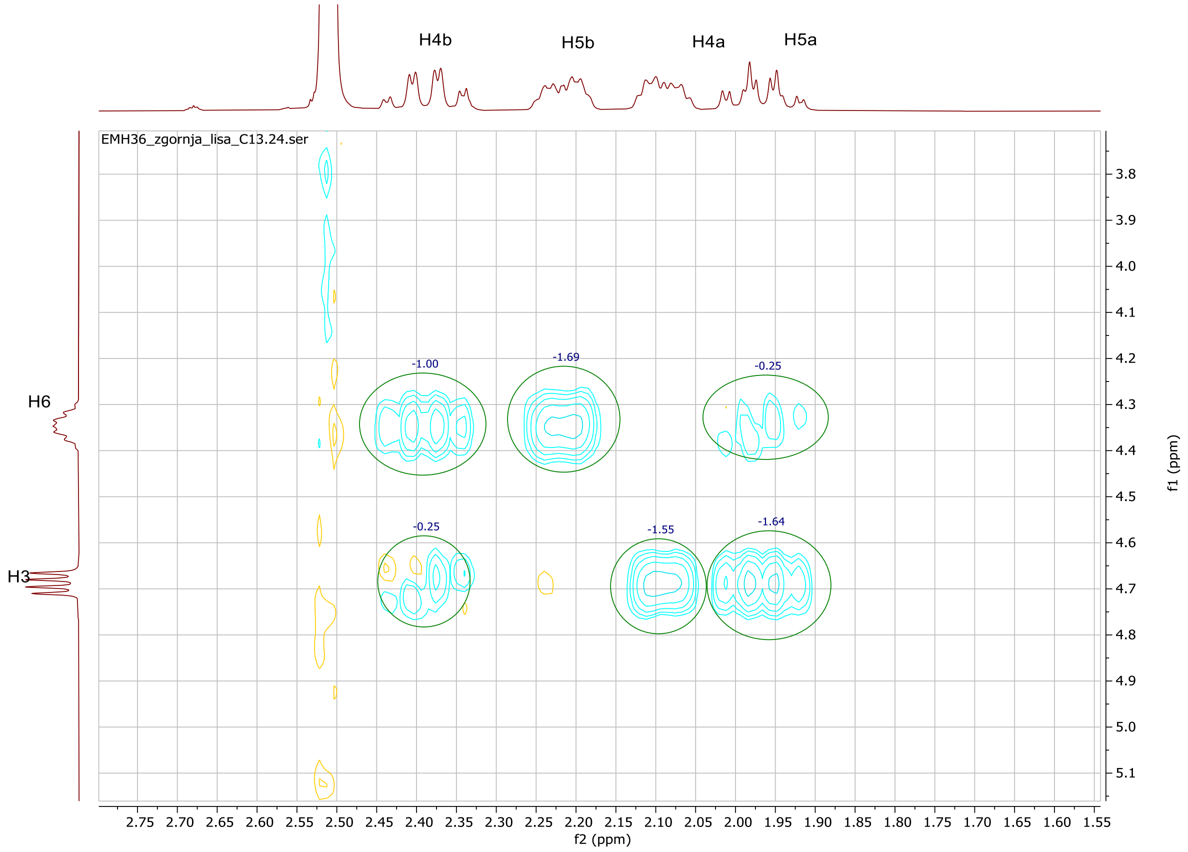

$^{1}H–^{19}F$ HOESY

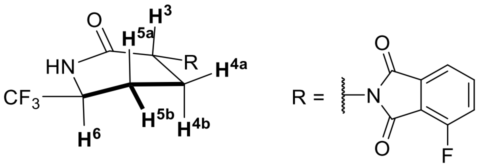

A strong correlation is observed between CF_3 and H6, CF_3 and H5a and a less intense correlation between CF_3 and H5b. There is also weak nOe between CF_3 and H3 and CF_3 and H4a. The presence of a weak CF_3-H3 correlation and the lack of CF_3-H4b correlation additionally confirms the *trans* relative configuration.

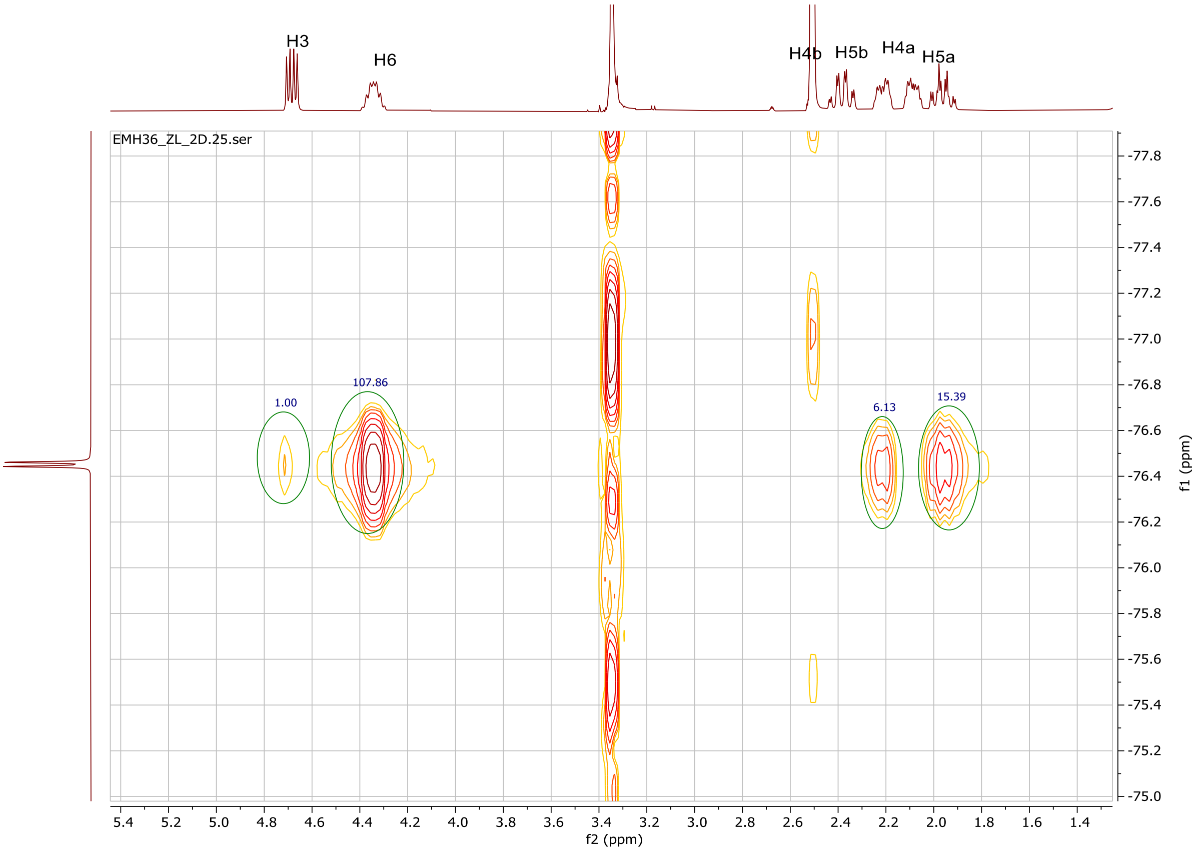

Compound 22

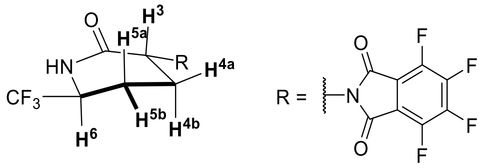

^1H–^1H COSY

In the COSY spectrum, cross-peaks indicate scalar couplings ($^3J_{HH}$) between the following pairs of protons: H3–H4a, H3–H4b, H6–H5a, H6–H5b, H5a–H4b, H5a–H4b, H4a–H5b, H4b–H5b; and geminal coupling ($^2J_{HH}$) between H5a–H5b, H4a–H4b.

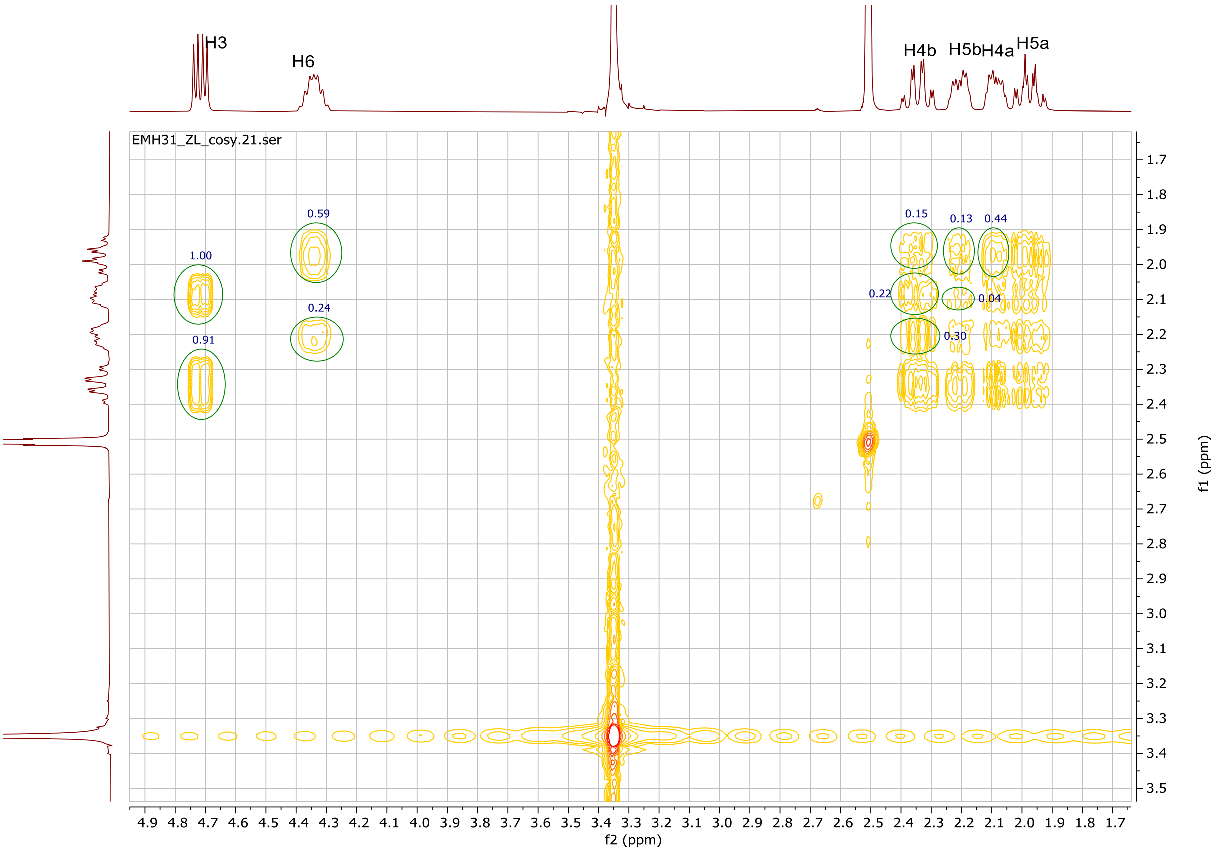

^{1}H–^{13}C HSQC

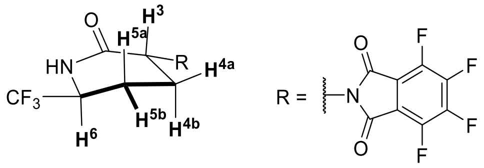

In the HSQC spectrum, H3–C3, H6–C6, H4b-C4, H5b-C5, H4a-C4 and H5a-C5 correlations confirm that these protons are directly attached to their respective carbons.

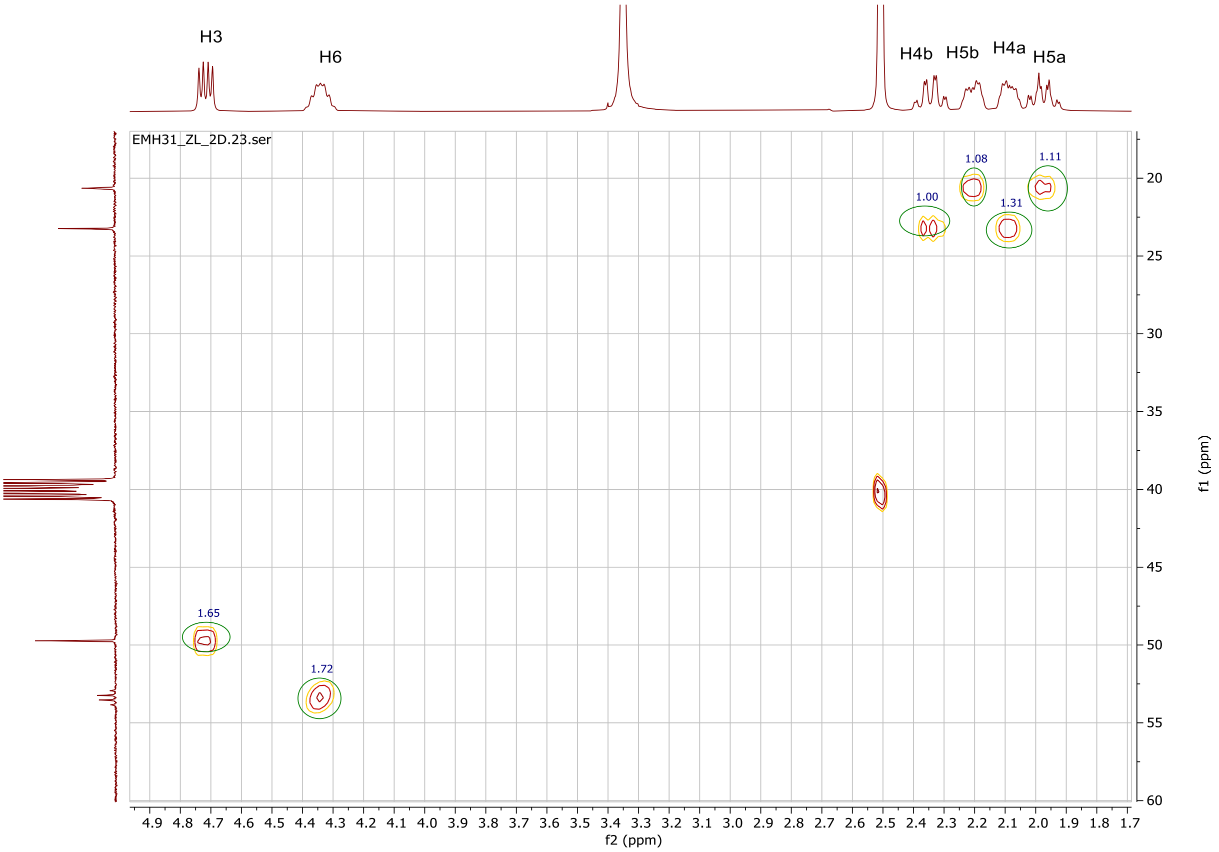

^{1}H–^{1}H NOESY

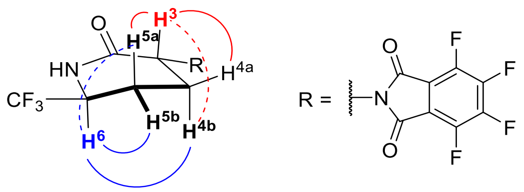

A strong nuclear Overhauser effect (nOe) is observed between H6 and H5b, and a weak nOe between H6 and its other vicinal proton, H5a. A strong nOe is observed between H6 and H4b. On the other hand, a weak nOe is observed between H3 and H4b, and a strong nOe between H3 and its other vicinal proton H4a, and with H5a. This confirms the *trans* relative configuration of the protons H3 and H6 at the chiral centers.

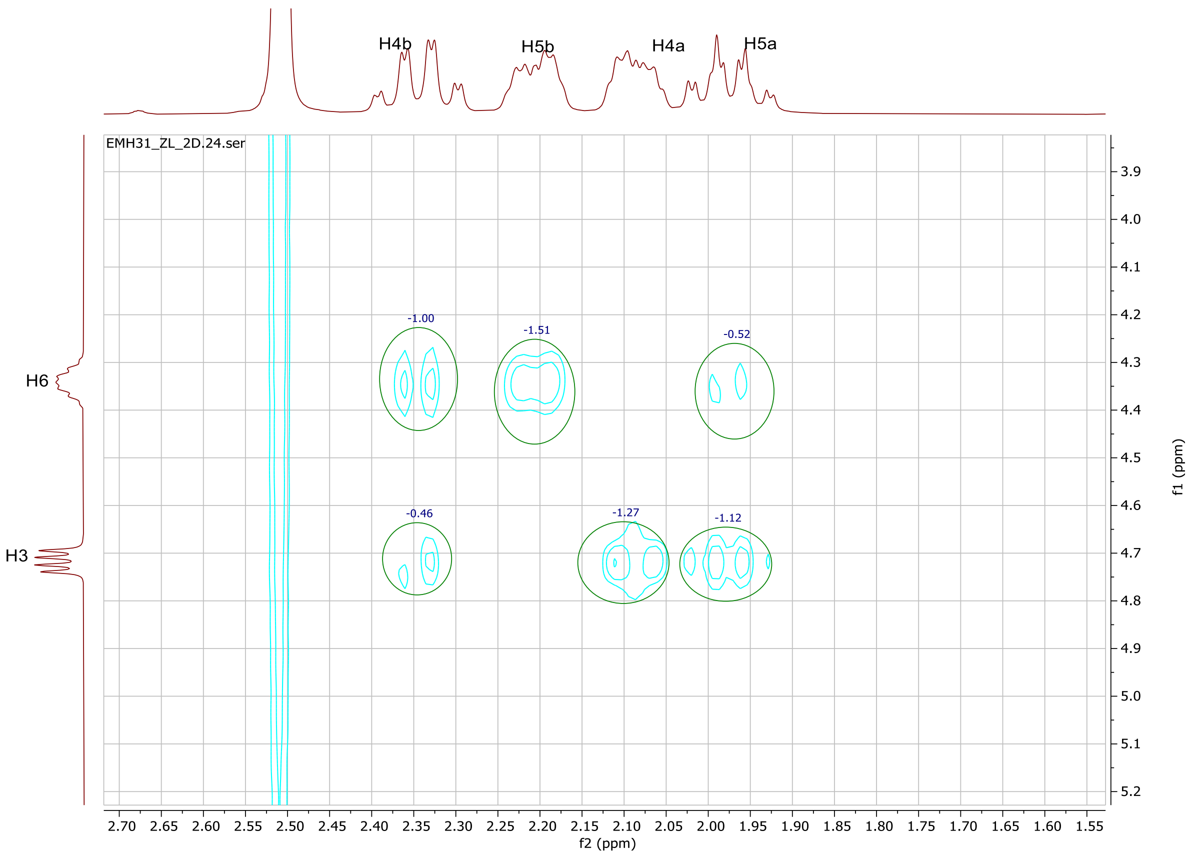

^{1}H–^{19}F HOESY

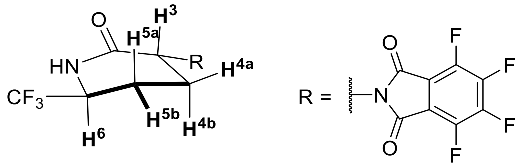

A strong correlation is observed between CF_3 and H6, CF_3 and H5a and a less intense correlation between CF_3 and H5b. There is also weak nOe between CF_3 and H3 and CF_3 and H4a. The presence of a weak CF_3-H3 correlation and the lack of CF_3-H4b correlation additionally confirms the *trans* relative configuration.

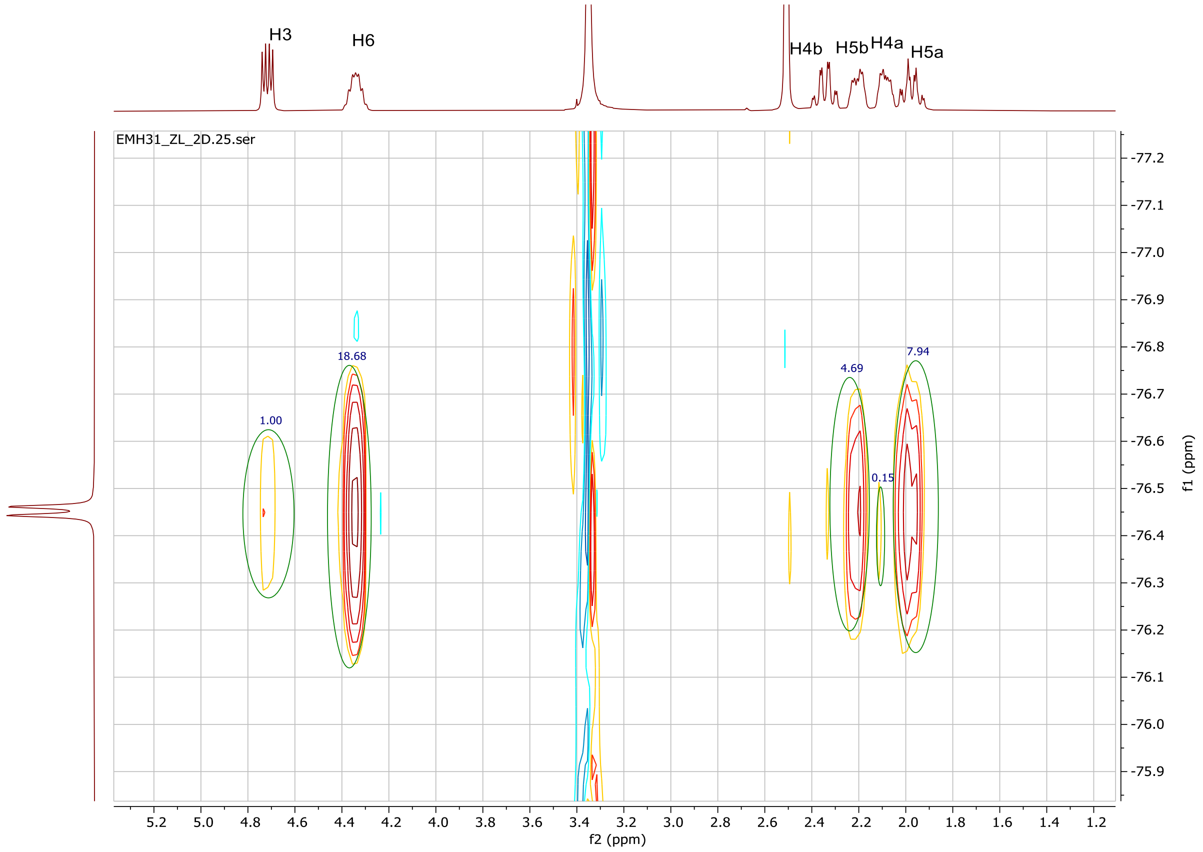

Compound 25

$^1H–^1H$ COSY

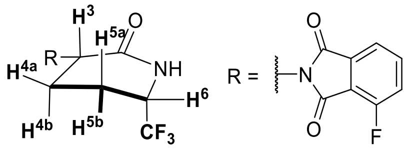

In the COSY spectrum, cross-peaks indicate scalar couplings ($^3J_{HH}$) between the following pairs of protons: H3–H4a, H3–H4b, H6–H5a, H6–H5b, H4a–H5a, H4a–H5b, H5b–H4b, H5a–H4b, and geminal couplings H4a–H4b, H5b–H5a.

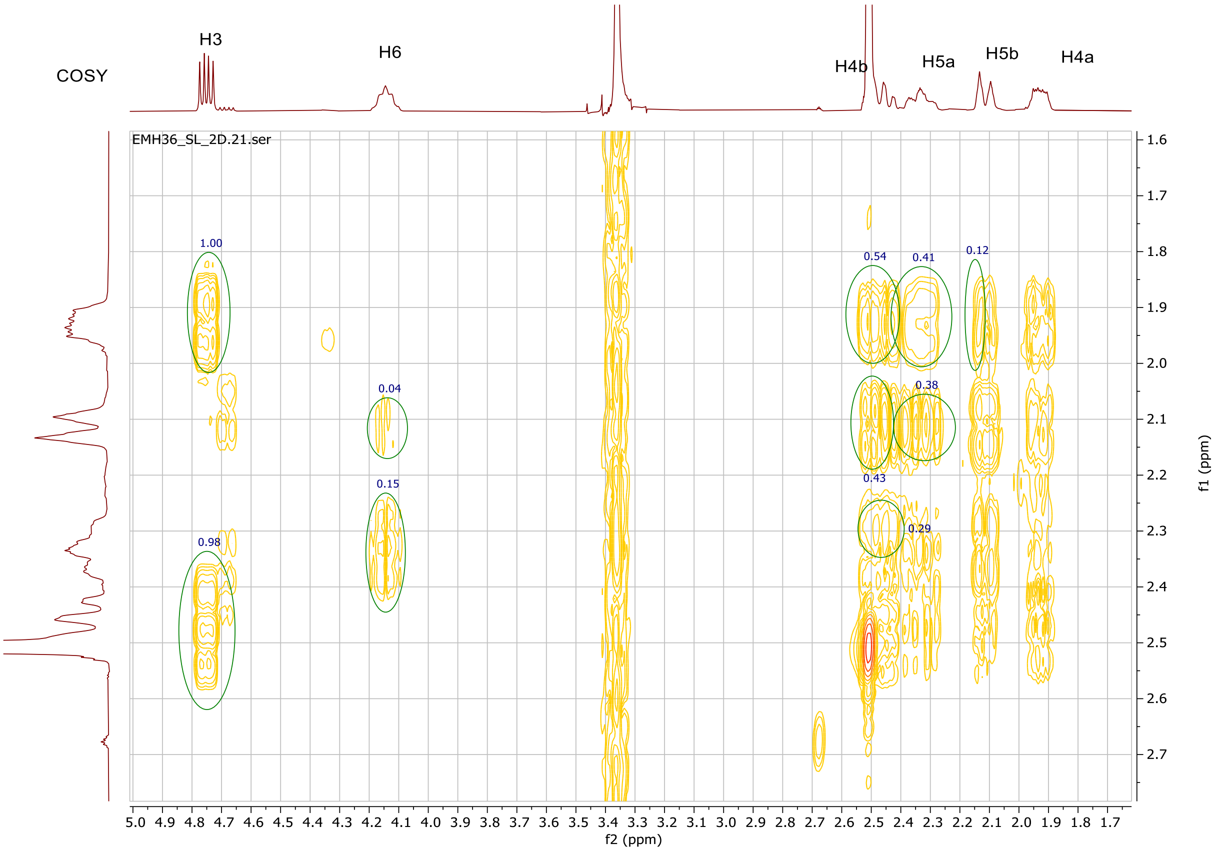

^{1}H–^{13}C HSQC

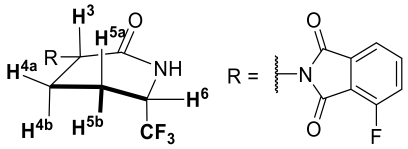

In the HSQC spectrum, H3–C3, H6–C6, H4b-C4, H5b-C5, H4a-C4 and H5a-C5 correlations confirm that these protons are directly attached to their respective carbons.

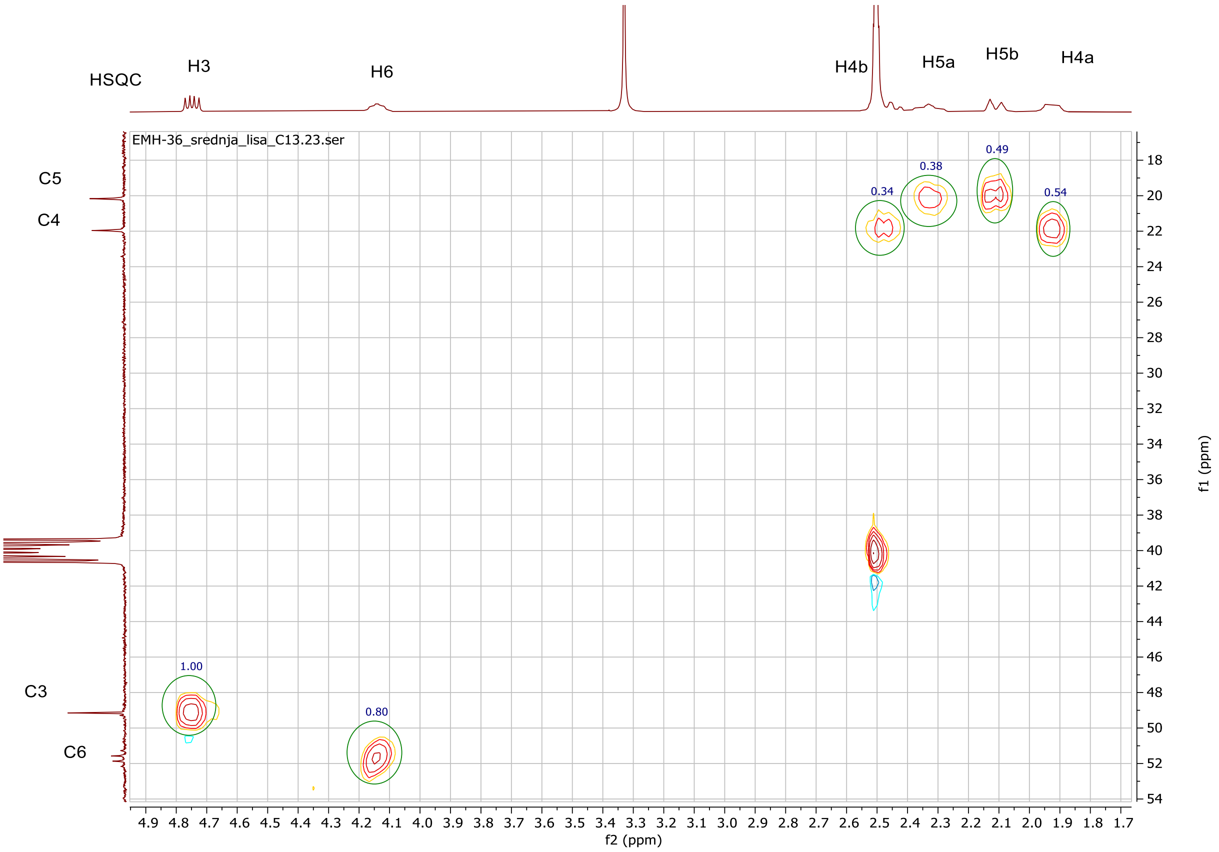

^{1}H–^{19}F HOESY

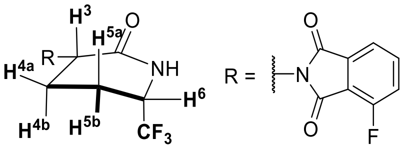

A strong correlation is observed between CF_3 and H6, CF_3 and H5b and CF_3 and H4b. There is also no correlation between CF_3 and H3, which confirms the *cis* relative configuration.

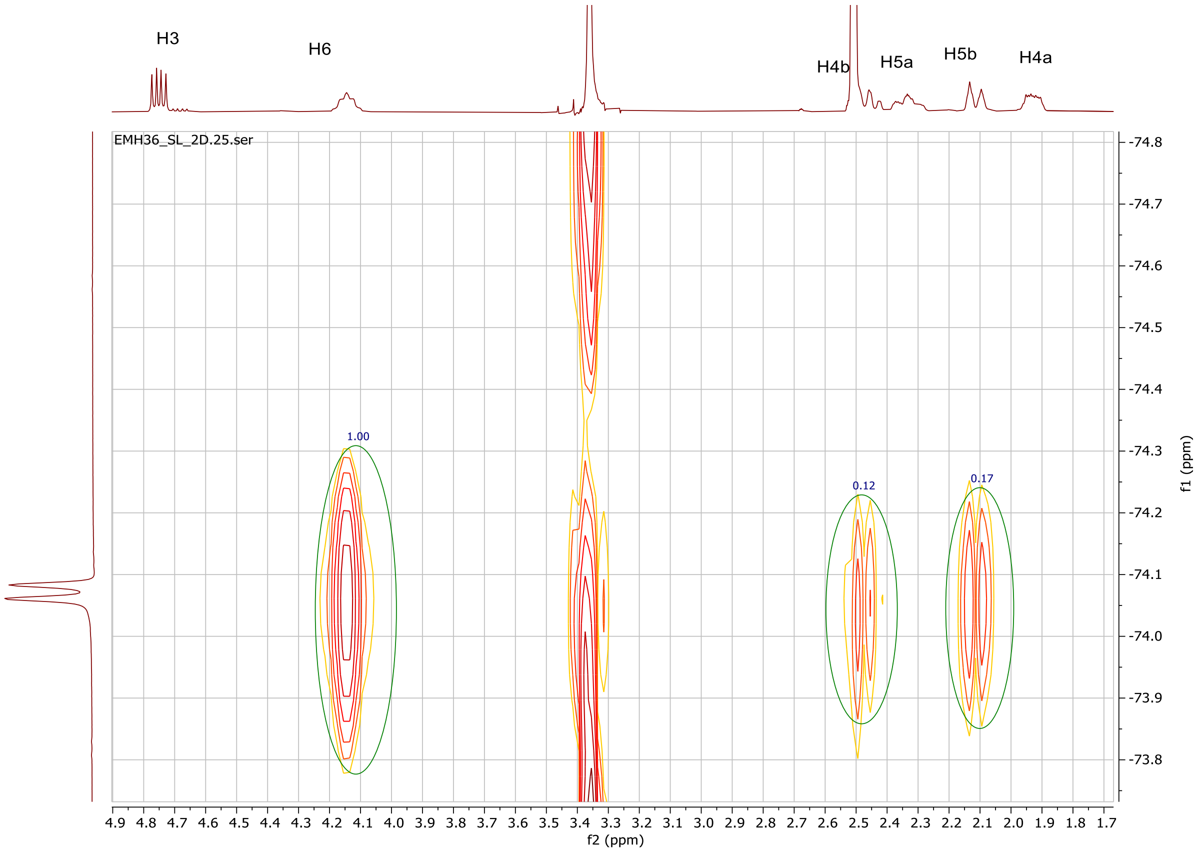

Compound 26

^{1}H–^{1}H COSY

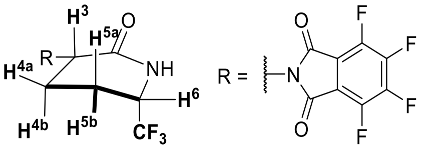

In the COSY spectrum, cross-peaks indicate scalar couplings ($^3J_{HH}$) between the following pairs of protons: H3–H4a, H3–H4b, H6–H5a, H6–H5b, H4a–H5a, H4a–H5b, H5b–H4b, H5a–H4b, and geminal couplings H4a–H4b, H5b–H5a.

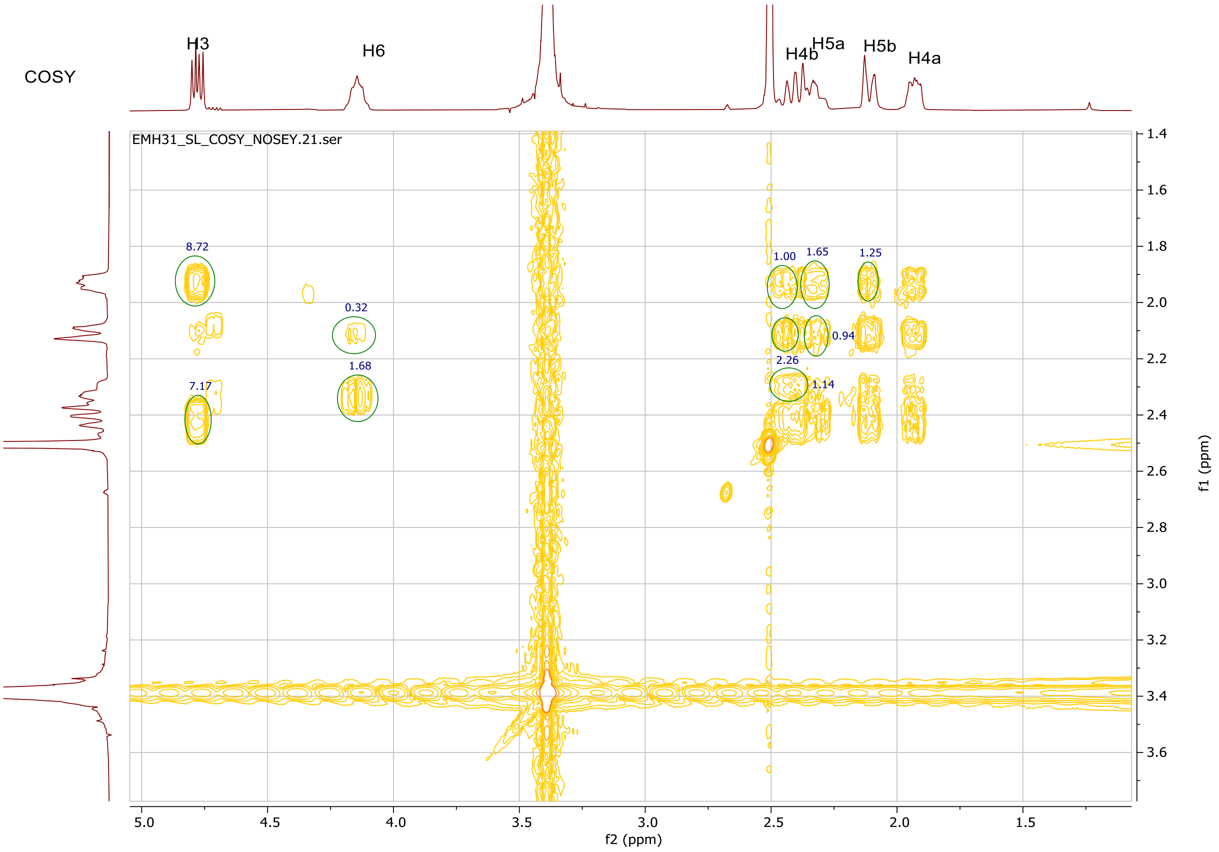

^{1}H–^{13}C HSQC

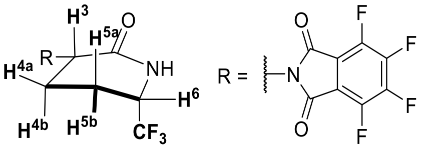

In the HSQC spectrum, H3–C3, H6–C6, H4b-C4, H5b-C5, H4a-C4 and H5a-C5 correlations confirm that these protons are directly attached to their respective carbons.

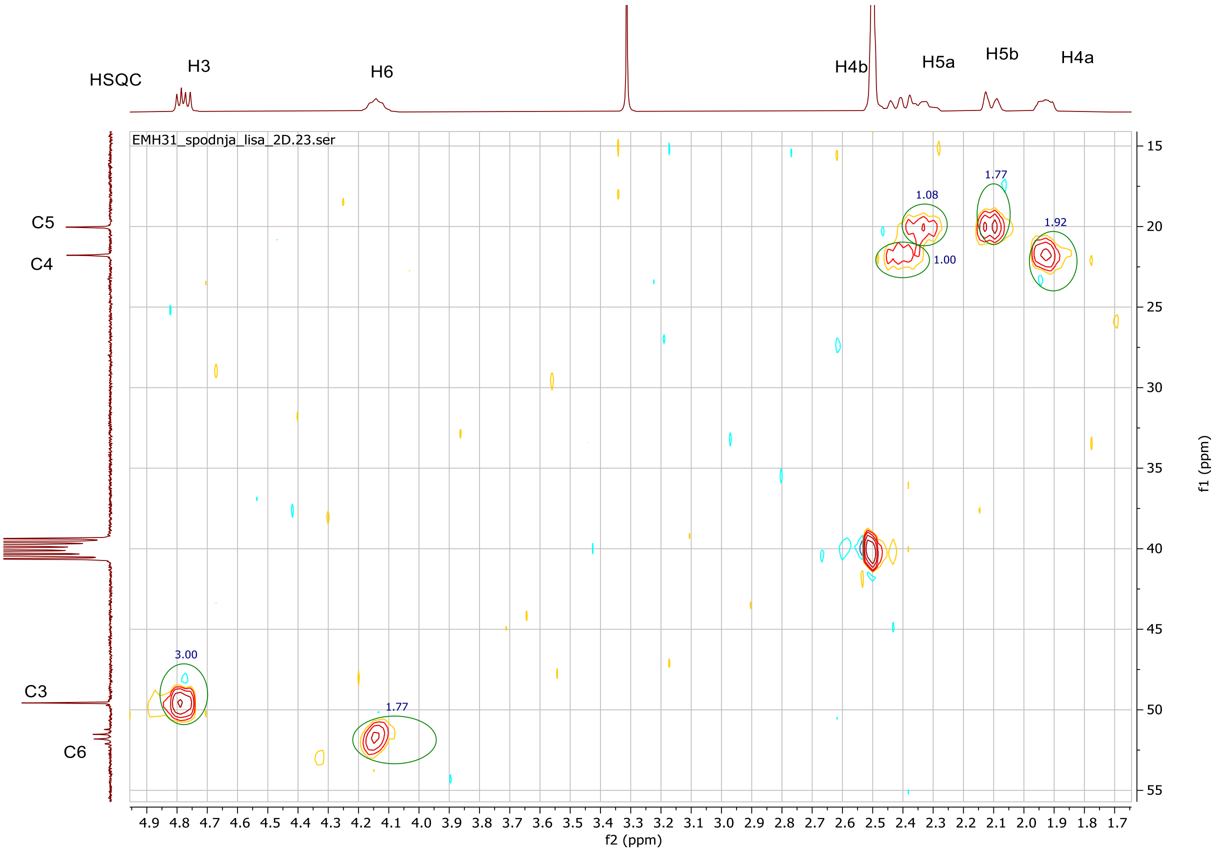

^{1}H–^{19}F HOESY

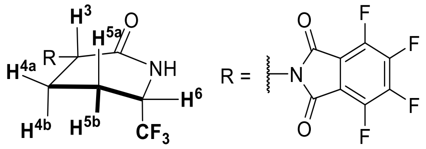

A strong correlation is observed between CF_3 and H6, CF_3 and H5b and CF_3 and H4b. There is also no correlation between CF_3 and H3, which confirms the *cis* relative configuration.

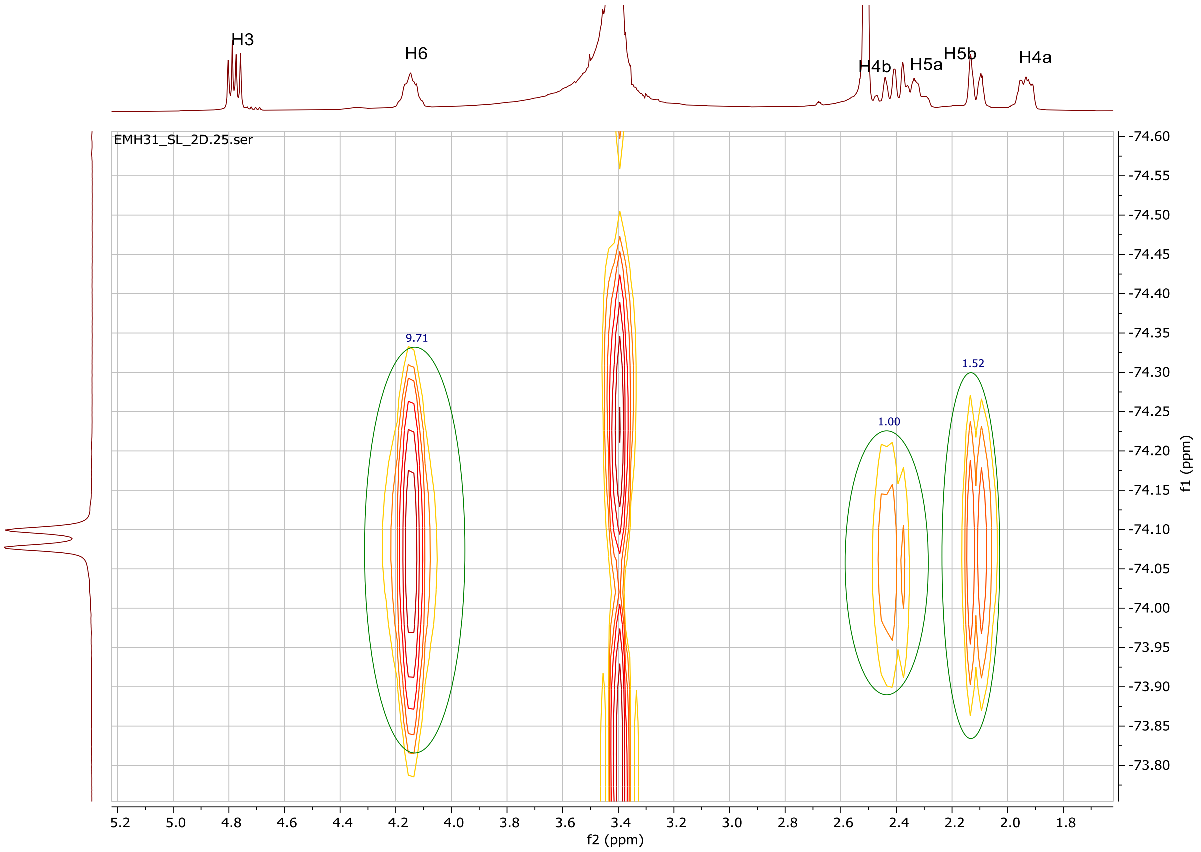

HPLC traces

Compound 6

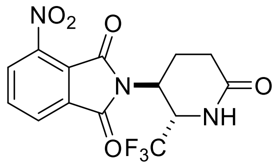

Chromatogram and Results

Injection Details

Injection Name:	**EBA-68**	*Run Time (min):*	**8,00**
Vial Number:	**RA2**	*Injection Volume:*	**1,00**
Injection Type:	**Unknown**	*Channel:*	**UV_VIS_2**
Calibration Level:		*Wavelength:*	**254**
Instrument Method:	**General method (0.1TFA_MeCN_10-90)**	*Bandwidth:*	**16**
Processing Method:	**Quantitative**	*Dilution Factor:*	**1,0000**
Injection Date/Time:	**30.okt..24 14:29**	*Sample Weight:*	**1,0000**

Chromatogram

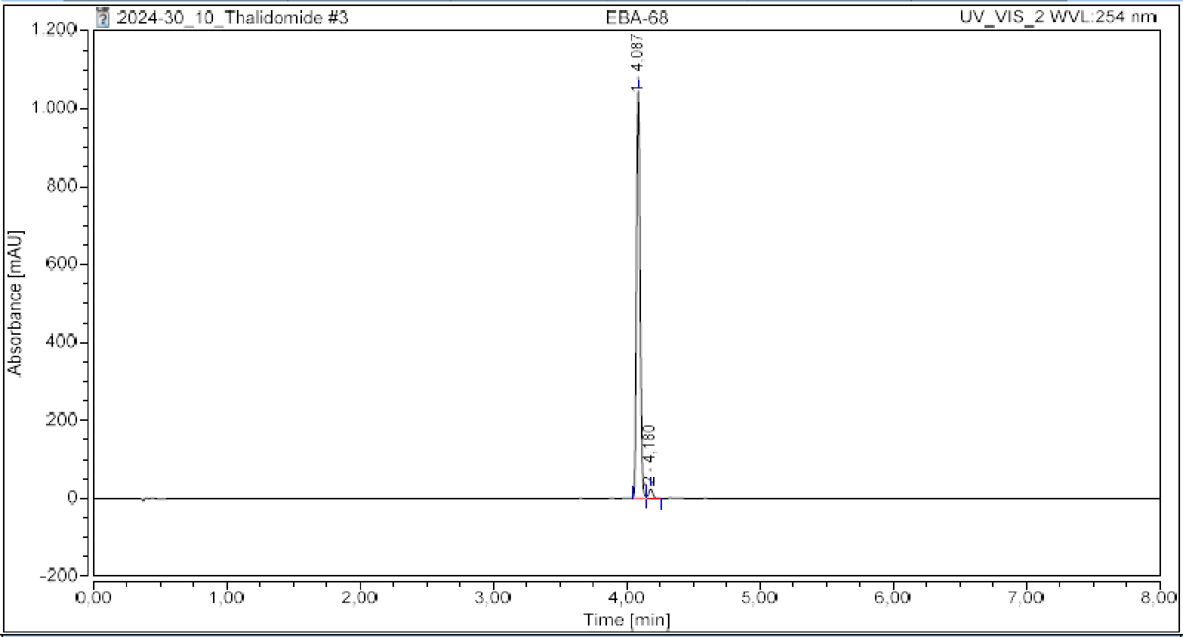

Integration Results

No.	Peak Name	Retention Time min	Area mAU*min	Height mAU	Relative Area %	Relative Height %	Amount n.a.
1		4,087	32,686	1044,746	97,54	97,60	n.a.
2		4,180	0,823	25,737	2,46	2,40	n.a.
**Total:**			**33,510**	**1070,482**	**100,00**	**100,00**	

Compound 7

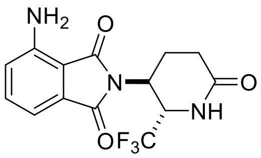

Chromatogram and Results

Injection Details

Injection Name:	**EBA-71**	*Run Time (min):*	**8,00**
Vial Number:	**RA3**	*Injection Volume:*	**1,00**
Injection Type:	**Unknown**	*Channel:*	**UV_VIS_2**
Calibration Level:		*Wavelength:*	**254**
Instrument Method:	**General method (0.1TFA_MeCN_10-90)**	*Bandwidth:*	**16**
Processing Method:	**Quantitative**	*Dilution Factor:*	**1,0000**
Injection Date/Time:	**30.okt..24 14:41**	*Sample Weight:*	**1,0000**

Chromatogram

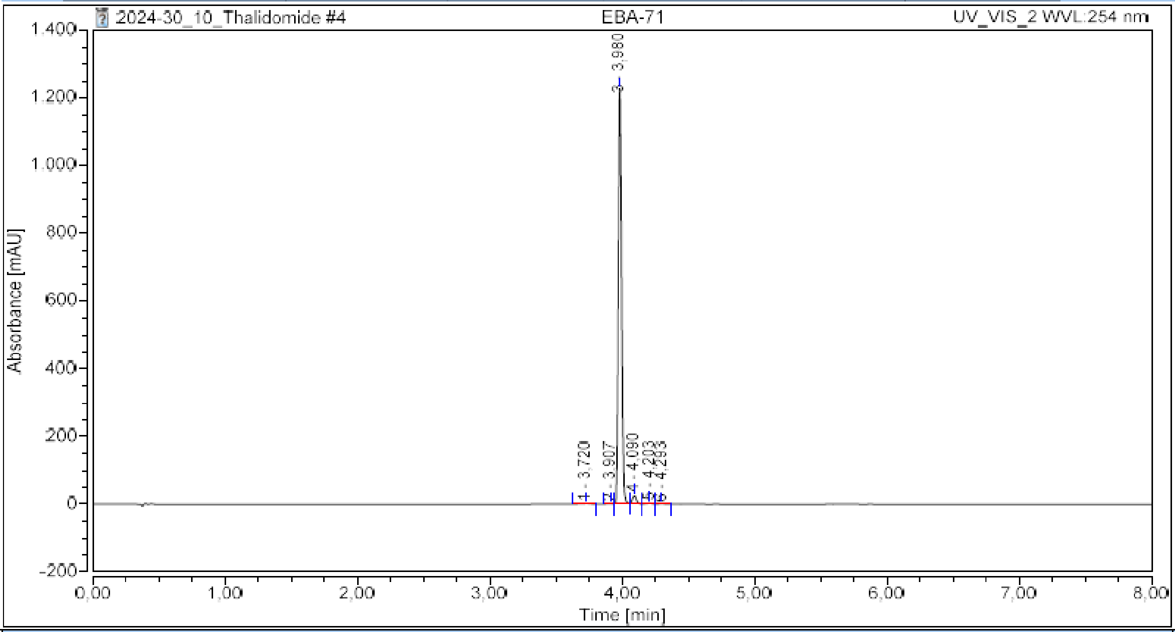

Integration Results

No.	Peak Name	Retention Time min	Area mAU*min	Height mAU	Relative Area %	Relative Height %	Amount n.a.
1		3,720	0,149	2,153	0,39	0,17	n.a.
2		3,907	0,073	1,940	0,19	0,15	n.a.
3		3,980	37,092	1228,182	96,59	97,21	n.a.
4		4,090	0,911	26,068	2,37	2,06	n.a.
5		4,203	0,122	3,489	0,32	0,28	n.a.
6		4,293	0,055	1,664	0,14	0,13	n.a.
**Total:**			**38,401**	**1263,496**	**100,00**	**100,00**	

Compound 8

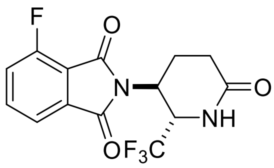

Chromatogram and Results

Injection Details

Injection Name:	**EBA-53**	*Run Time (min):*	**8,00**
Vial Number:	**RA5**	*Injection Volume:*	**1,00**
Injection Type:	**Unknown**	*Channel:*	**UV_VIS_2**
Calibration Level:		*Wavelength:*	**254**
Instrument Method:	**General method (0.1TFA_MeCN_10-90)**	*Bandwidth:*	**16**
Processing Method:	**Quantitative**	*Dilution Factor:*	**1,0000**
Injection Date/Time:	**30.okt..24 15:06**	*Sample Weight:*	**1,0000**

Chromatogram

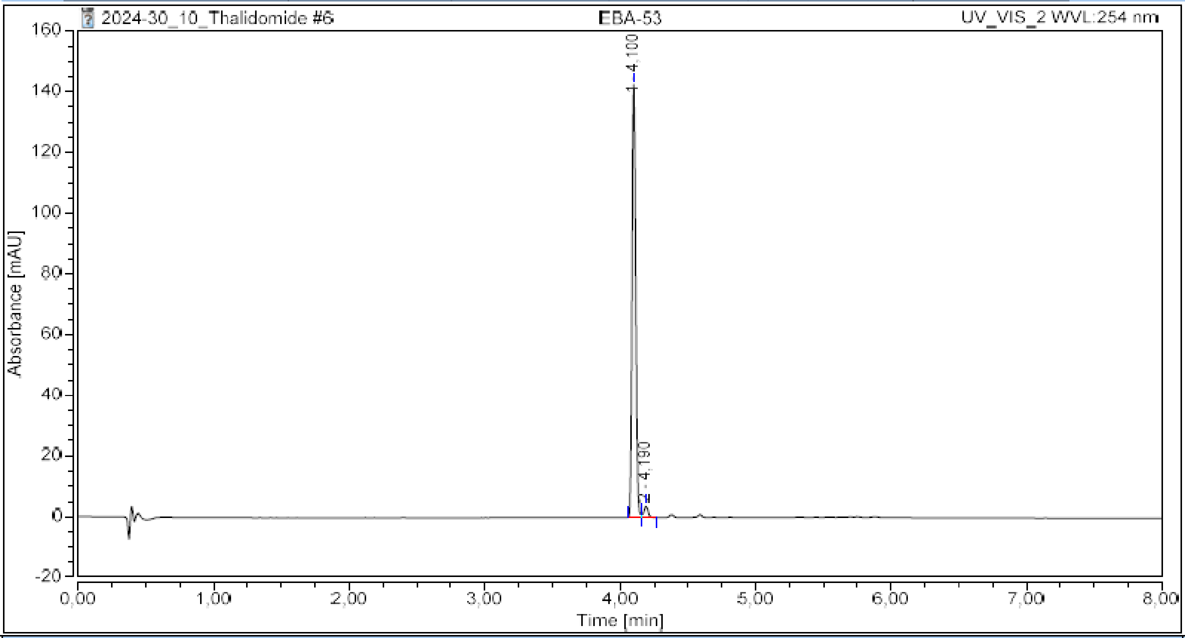

Integration Results

No.	Peak Name	Retention Time min	Area mAU*min	Height mAU	Relative Area %	Relative Height %	Amount n.a.
1		4,100	4,434	142,662	97,19	97,34	n.a.
2		4,190	0,128	3,894	2,81	2,66	n.a.
**Total:**			**4,562**	**146,556**	**100,00**	**100,00**	

Compound 9

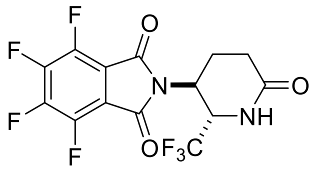

Chromatogram and Results

**Injection Details**			
Injection Name:	**EMH33_izolera**	*Run Time (min):*	**8,00**
Vial Number:	**GD2**	*Injection Volume:*	**5,00**
Injection Type:	**Unknown**	*Channel:*	**UV_VIS_2**
Calibration Level:		*Wavelength:*	**254**
Instrument Method:	**General method (0.1TFA_MeCN_10-90)**	*Bandwidth:*	**16**
Processing Method:	**Quantitative**	*Dilution Factor:*	**1,0000**
Injection Date/Time:	**21.nov..24 15:16**	*Sample Weight:*	**1,0000**

Chromatogram

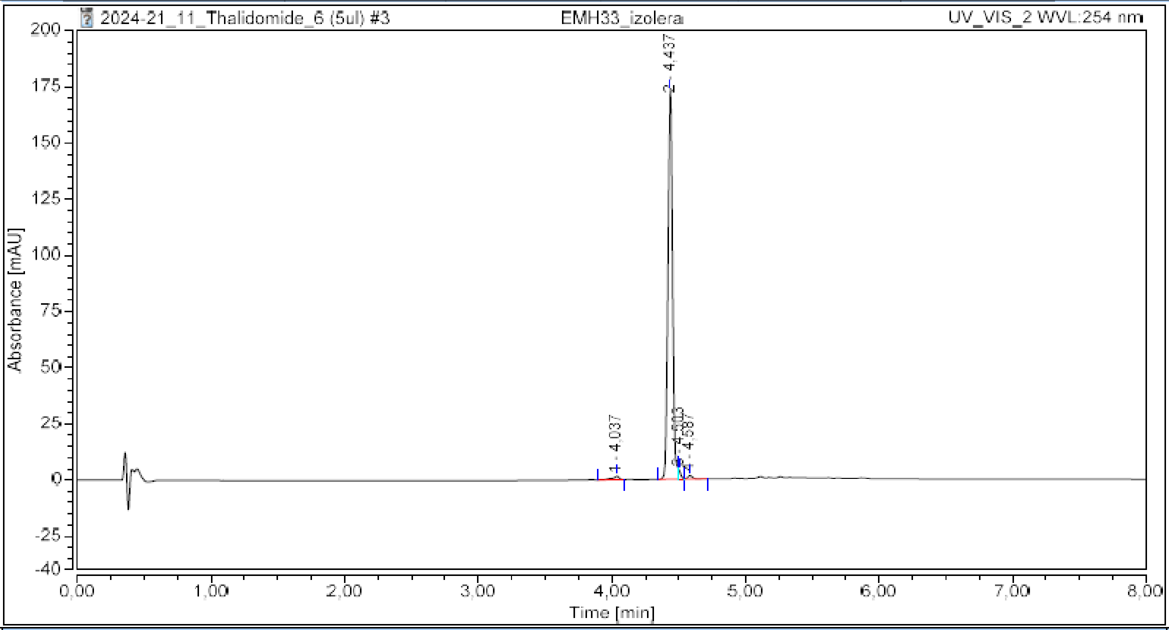

Integration Results

No.	Peak Name	Retention Time min	Area mAU*min	Height mAU	Relative Area %	Relative Height %	Amount n.a.
1		4,037	0,100	1,707	1,33	0,94	n.a.
2		4,437	7,253	172,959	96,24	95,71	n.a.
3		4,503	0,110	4,352	1,46	2,41	n.a.
4		4,587	0,073	1,689	0,97	0,93	n.a.
**Total:**			**7,536**	**180,707**	**100,00**	**100,00**	

Compound 19

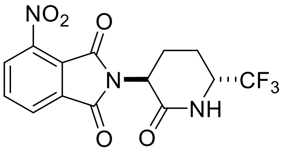

Chromatogram and Results

Injection Details

Injection Name:	**EMH24_zgornja_lisa**	*Run Time (min):*	**8,00**
Vial Number:	**BA3**	*Injection Volume:*	**1,00**
Injection Type:	**Unknown**	*Channel:*	**UV_VIS_2**
Calibration Level:		*Wavelength:*	**254**
Instrument Method:	**General method (0.1TFA_MeCN_10-90)**	*Bandwidth:*	**16**
Processing Method:	**Quantitative**	*Dilution Factor:*	**1,0000**
Injection Date/Time:	**09.nov..24 13:06**	*Sample Weight:*	**1,0000**

Chromatogram

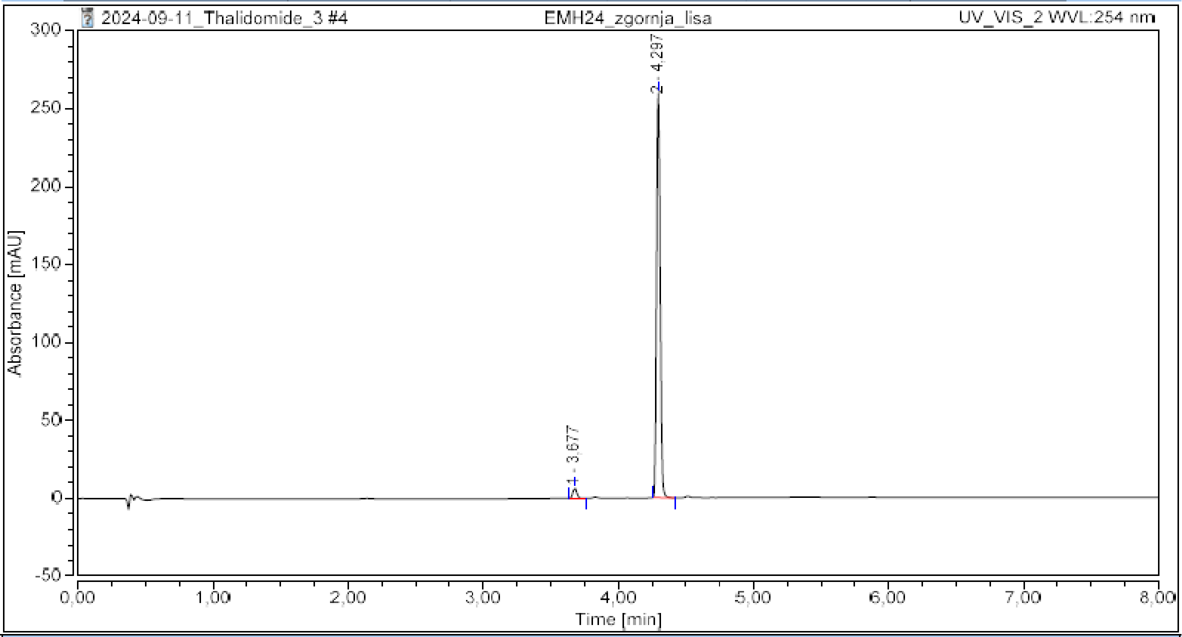

Integration Results

No.	Peak Name	Retention Time min	Area mAU*min	Height mAU	Relative Area %	Relative Height %	Amount n.a.
1		3,677	0,220	6,633	2,70	2,49	n.a.
2		4,297	7,920	259,487	97,30	97,51	n.a.
**Total:**			**8,140**	**266,120**	**100,00**	**100,00**	

Compound 20

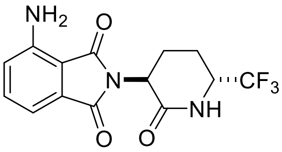

Chromatogram and Results

Injection Details

Injection Name:	**EMH28**	*Run Time (min):*	**8,00**
Vial Number:	**RA7**	*Injection Volume:*	**1,00**
Injection Type:	**Unknown**	*Channel:*	**UV_VIS_2**
Calibration Level:		*Wavelength:*	**254**
Instrument Method:	**General method (0.1TFA_MeCN_10-90)**	*Bandwidth:*	**16**
Processing Method:	**Quantitative**	*Dilution Factor:*	**1,0000**
Injection Date/Time:	**30.okt..24 18:24**	*Sample Weight:*	**1,0000**

Chromatogram

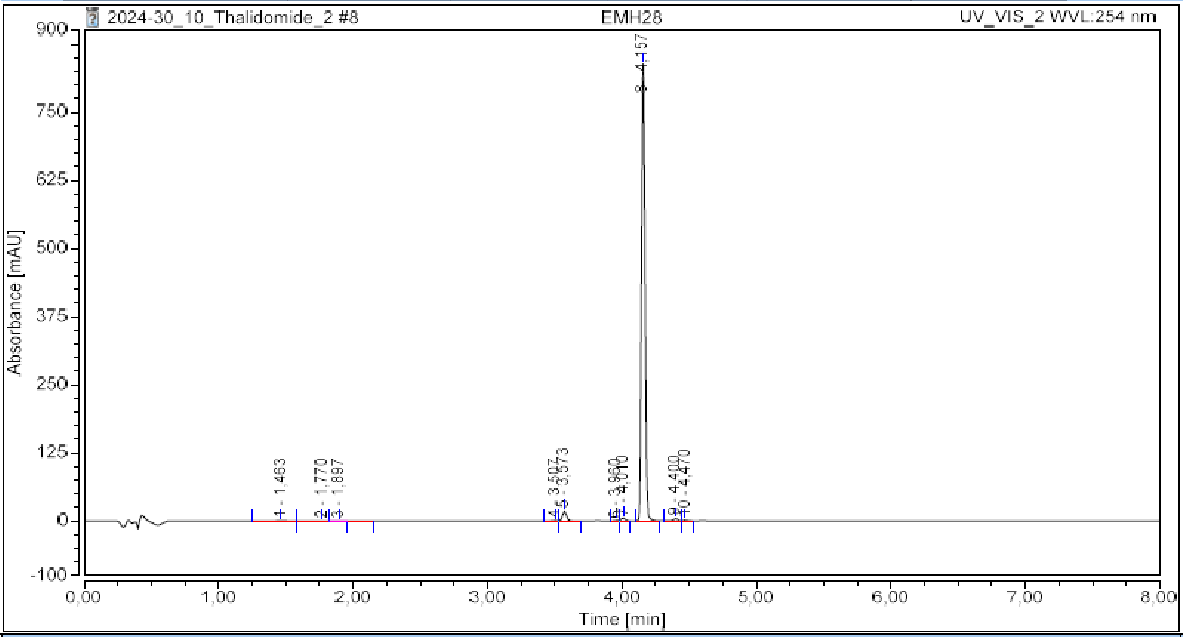

Integration Results

No.	Peak Name	Retention Time min	Area mAU*min	Height mAU	Relative Area %	Relative Height %	Amount n.a.
1		1,463	0,085	0,740	0,32	0,08	n.a.
2		1,770	0,049	0,189	0,19	0,02	n.a.
3		1,897	0,002	0,032	0,01	0,00	n.a.
4		3,507	0,046	0,919	0,17	0,11	n.a.
5		3,573	0,658	19,239	2,50	2,21	n.a.
6		3,960	0,032	0,972	0,12	0,11	n.a.
7		4,010	0,177	5,339	0,67	0,61	n.a.
8		4,157	25,089	837,819	95,23	96,15	n.a.
9		4,400	0,155	4,544	0,59	0,52	n.a.
10		4,470	0,053	1,614	0,20	0,19	n.a.
**Total:**			**26,345**	**871,409**	**100,00**	**100,00**	

Compound 21

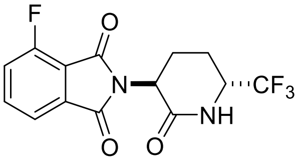

Chromatogram and Results

Injection Details

Injection Name:	**EMH36_zgornja_lisa**	*Run Time (min):*	**8,00**
Vial Number:	**RA6**	*Injection Volume:*	**1,00**
Injection Type:	**Unknown**	*Channel:*	**UV_VIS_2**
Calibration Level:		*Wavelength:*	**254**
Instrument Method:	**General method (0.1TFA_MeCN_10-90)**	*Bandwidth:*	**16**
Processing Method:	**Quantitative**	*Dilution Factor:*	**1,0000**
Injection Date/Time:	**30.okt..24 15:19**	*Sample Weight:*	**1,0000**

Chromatogram

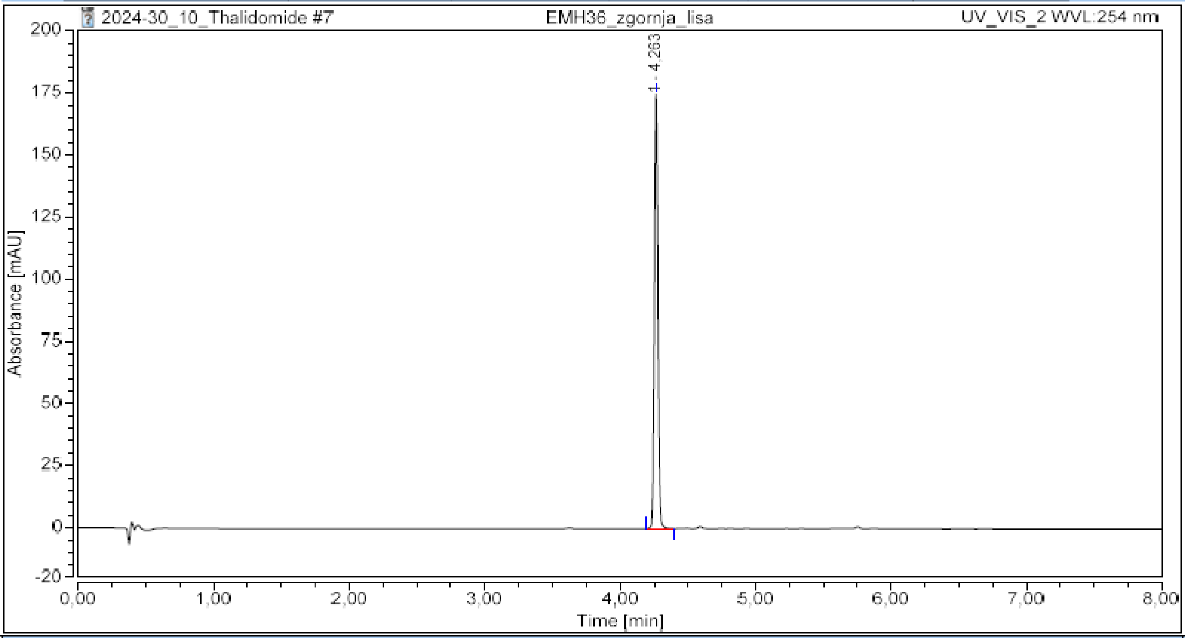

Integration Results

No.	Peak Name	Retention Time min	Area mAU*min	Height mAU	Relative Area %	Relative Height %	Amount n.a.
1		4,263	5,515	174,857	100,00	100,00	n.a.
**Total:**			**5,515**	**174,857**	**100,00**	**100,00**	

Compound 21

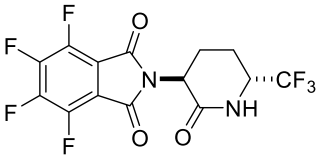

Chromatogram and Results

Injection Details

Injection Name:	**EMH31_zgornja_lisa**	*Run Time (min):*	**8,00**
Vial Number:	**RB2**	*Injection Volume:*	**1,00**
Injection Type:	**Unknown**	*Channel:*	**UV_VIS_2**
Calibration Level:		*Wavelength:*	**254**
Instrument Method:	**General method (0.1TFA_MeCN_10-90)**	*Bandwidth:*	**16**
Processing Method:	**Quantitative**	*Dilution Factor:*	**1,0000**
Injection Date/Time:	**30.okt..24 17:34**	*Sample Weight:*	**1,0000**

Chromatogram

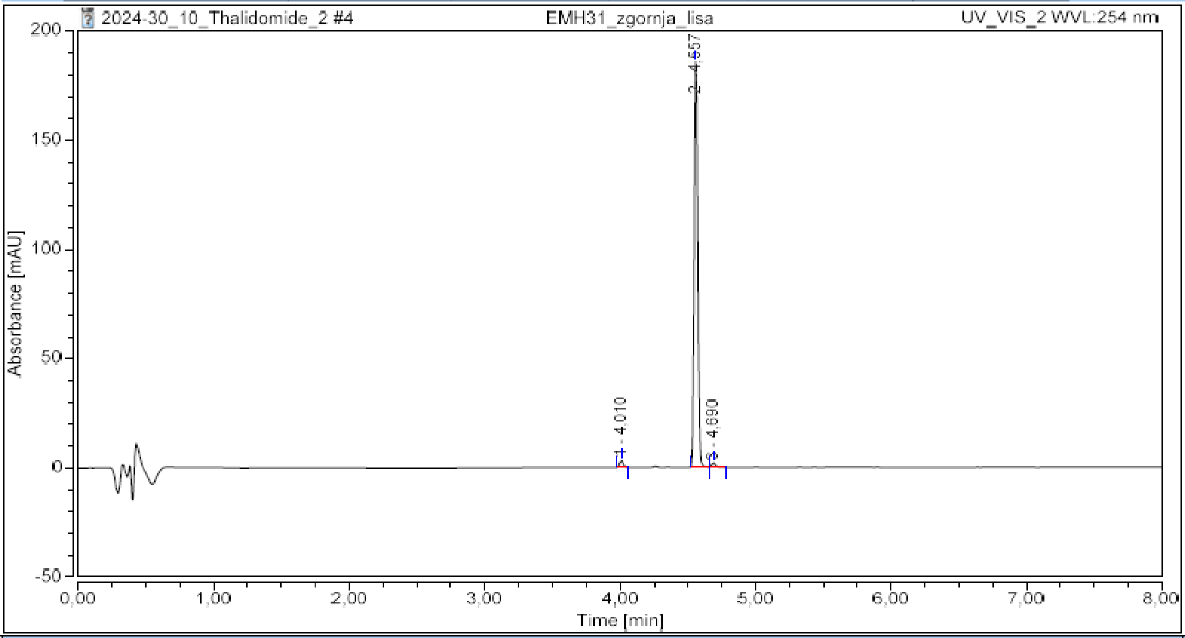

Integration Results

No.	Peak Name	Retention Time min	Area mAU*min	Height mAU	Relative Area %	Relative Height %	Amount n.a.
1		4,010	0,094	3,157	1,61	1,65	n.a.
2		4,557	5,645	185,675	97,18	97,22	n.a.
3		4,690	0,070	2,145	1,20	1,12	n.a.
**Total:**			**5,808**	**190,976**	**100,00**	**100,00**	

Compound 23

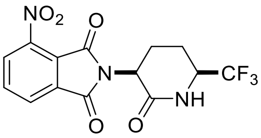

Chromatogram and Results

Injection Details

Injection Name:	**EMH24_f16**	*Run Time (min):*	**8,00**
Vial Number:	**BA4**	*Injection Volume:*	**1,00**
Injection Type:	**Unknown**	*Channel:*	**UV_VIS_2**
Calibration Level:		*Wavelength:*	**254**
Instrument Method:	**General method (0.1TFA_MeCN_10-90)**	*Bandwidth:*	**16**
Processing Method:	**Quantitative**	*Dilution Factor:*	**1,0000**
Injection Date/Time:	**09.nov..24 13:19**	*Sample Weight:*	**1,0000**

Chromatogram

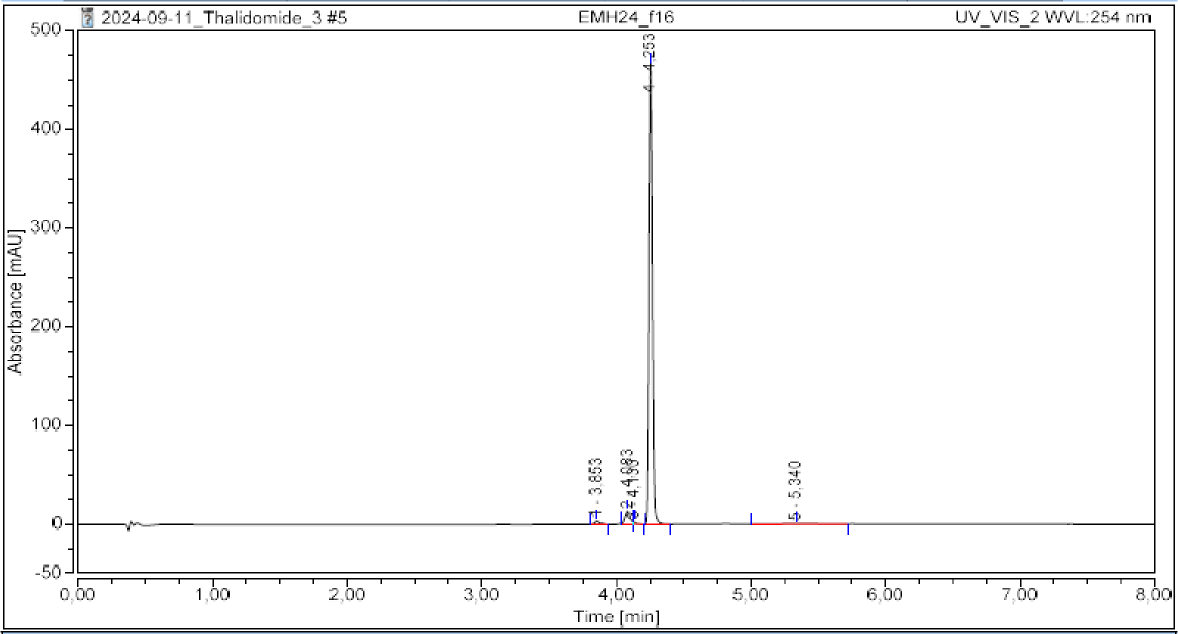

Integration Results

No.	Peak Name	Retention Time min	Area mAU*min	Height mAU	Relative Area %	Relative Height %	Amount n.a.
1		3,853	0,142	3,250	0,94	0,67	n.a.
2		4,083	0,516	12,878	3,41	2,66	n.a.
3		4,130	0,056	2,207	0,37	0,46	n.a.
4		4,253	14,282	465,036	94,46	96,15	n.a.
5		5,340	0,122	0,309	0,81	0,06	n.a.
**Total:**			**15,120**	**483,680**	**100,00**	**100,00**	

Compound 24

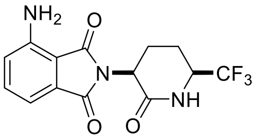

Chromatogram and Results

Injection Details			
Injection Name:	**EMH29**	*Run Time (min):*	**8,00**
Vial Number:	**RB1**	*Injection Volume:*	**1,00**
Injection Type:	**Unknown**	*Channel:*	**UV_VIS_2**
Calibration Level:		*Wavelength:*	**254**
Instrument Method:	**General method (0.1TFA_MeCN_10-90)**	*Bandwidth:*	**16**
Processing Method:	**Quantitative**	*Dilution Factor:*	**1,0000**
Injection Date/Time:	**30.okt..24 17:21**	*Sample Weight:*	**1,0000**

Chromatogram

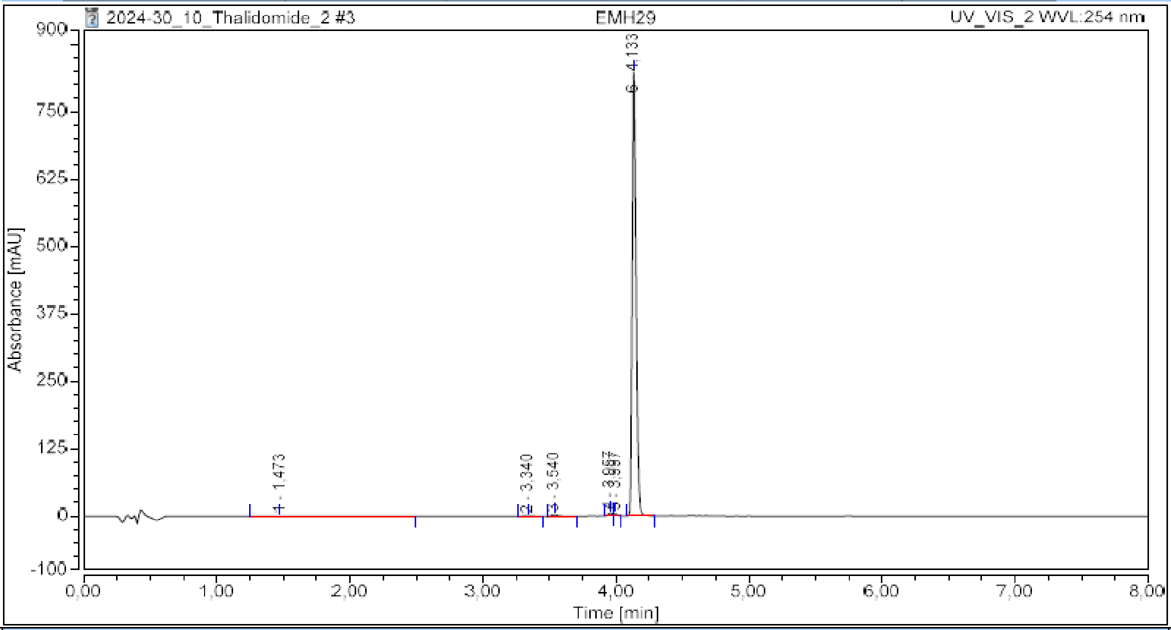

Integration Results

No.	Peak Name	Retention Time min	Area mAU*min	Height mAU	Relative Area %	Relative Height %	Amount n.a.
1		1,473	0,071	0,132	0,26	0,02	n.a.
2		3,340	0,053	0,742	0,19	0,09	n.a.
3		3,540	0,119	2,668	0,43	0,32	n.a.
4		3,957	0,178	5,781	0,64	0,69	n.a.
5		3,997	0,119	3,880	0,43	0,46	n.a.
6		4,133	27,202	823,521	98,06	98,42	n.a.
**Total:**			**27,742**	**836,723**	**100,00**	**100,00**	

Compound 25

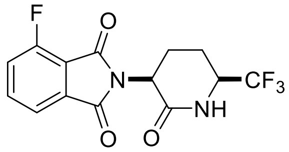

Chromatogram and Results

Injection Details

Injection Name:	**EMH36_srednja_lisa**	*Run Time (min):*	**8,00**
Vial Number:	**RB5**	*Injection Volume:*	**1,00**
Injection Type:	**Unknown**	*Channel:*	**UV_VIS_2**
Calibration Level:		*Wavelength:*	**254**
Instrument Method:	**General method (0.1TFA_MeCN_10-90)**	*Bandwidth:*	**16**
Processing Method:	**Quantitative**	*Dilution Factor:*	**1,0000**
Injection Date/Time:	**30.okt..24 18:11**	*Sample Weight:*	**1,0000**

Chromatogram

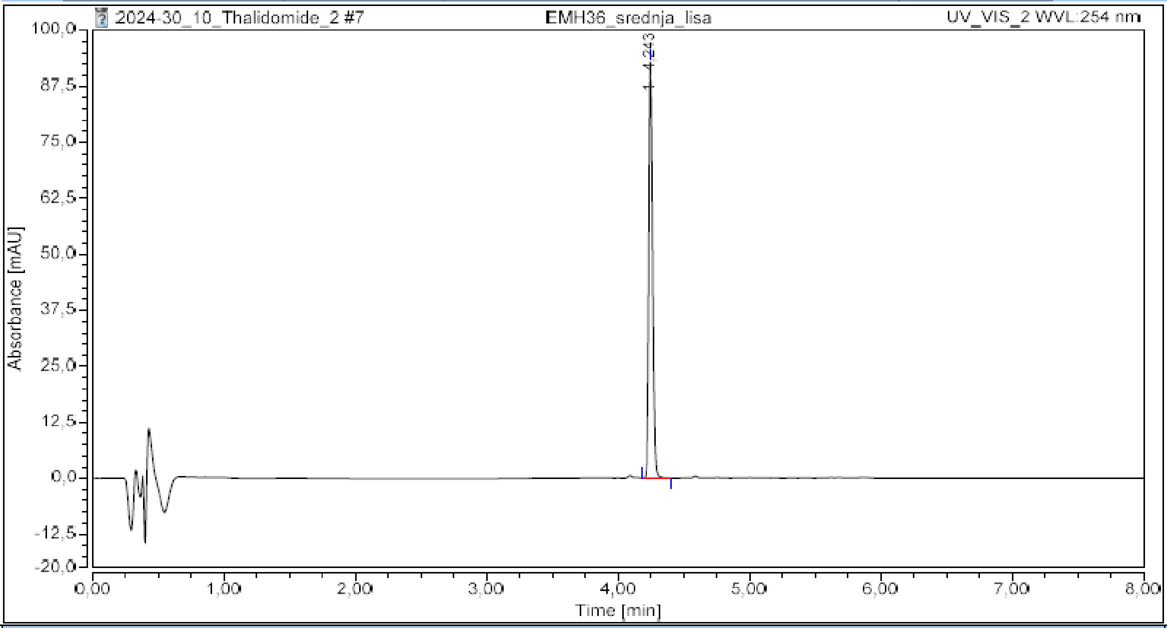

Integration Results

No.	Peak Name	Retention Time min	Area mAU*min	Height mAU	Relative Area %	Relative Height %	Amount n.a.
1		4,243	3,101	92,894	100,00	100,00	n.a.
**Total:**			**3,101**	**92,894**	**100,00**	**100,00**	

Compound 26

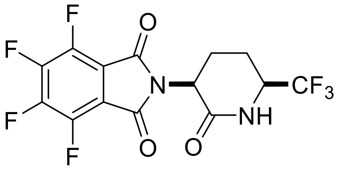

Chromatogram and Results

Injection Details

Injection Name:	**EMH31_spodnja_lisa**	*Run Time (min):*	**8,00**
Vial Number:	**RB4**	*Injection Volume:*	**1,00**
Injection Type:	**Unknown**	*Channel:*	**UV_VIS_2**
Calibration Level:		*Wavelength:*	**254**
Instrument Method:	**General method (0.1TFA_MeCN_10-90)**	*Bandwidth:*	**16**
Processing Method:	**Quantitative**	*Dilution Factor:*	**1,0000**
Injection Date/Time:	**30.okt..24 17:59**	*Sample Weight:*	**1,0000**

Chromatogram

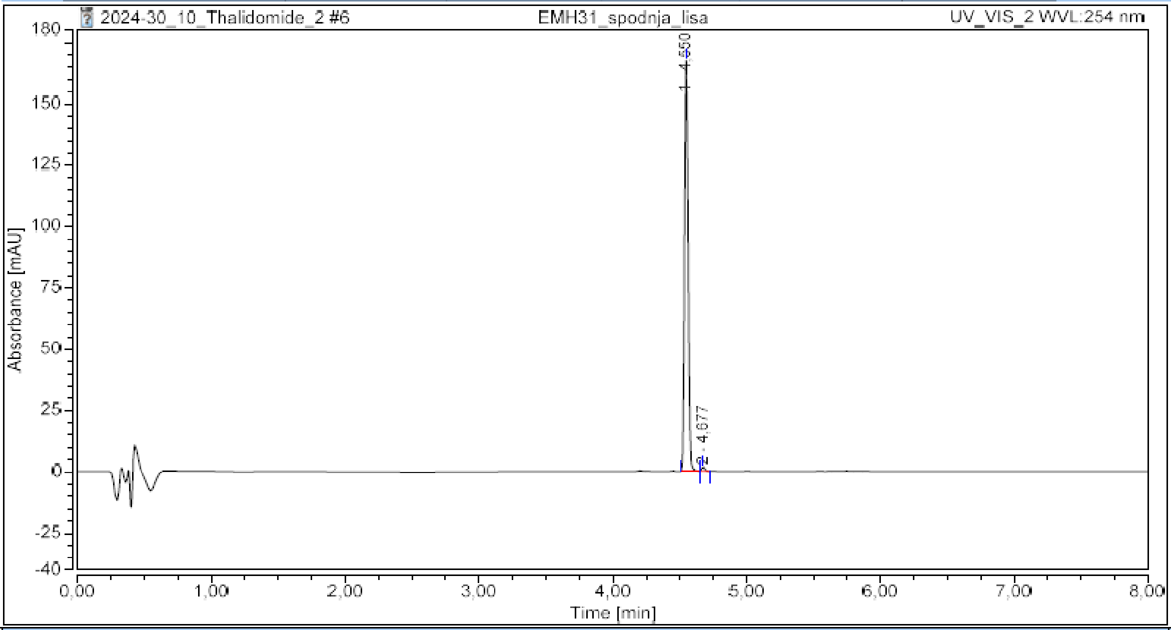

Integration Results

No.	Peak Name	Retention Time min	Area mAU*min	Height mAU	Relative Area %	Relative Height %	Amount n.a.
1		4,550	5,114	167,577	99,04	98,97	n.a.
2		4,677	0,050	1,746	0,96	1,03	n.a.
**Total:**			**5,163**	**169,323**	**100,00**	**100,00**	

Compound 12

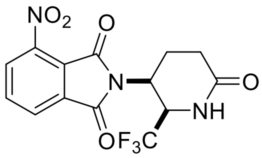

Chromatogram and Results

Injection Details

Injection Name:	**EBA-211_f4-7**	*Run Time (min):*	**8,00**
Vial Number:	**BA2**	*Injection Volume:*	**1,00**
Injection Type:	**Unknown**	*Channel:*	**UV_VIS_2**
Calibration Level:		*Wavelength:*	**254**
Instrument Method:	**General method (0.1TFA_MeCN_10-90)**	*Bandwidth:*	**16**
Processing Method:	**Quantitative**	*Dilution Factor:*	**1,0000**
Injection Date/Time:	**09.nov..24 12:54**	*Sample Weight:*	**1,0000**

Chromatogram

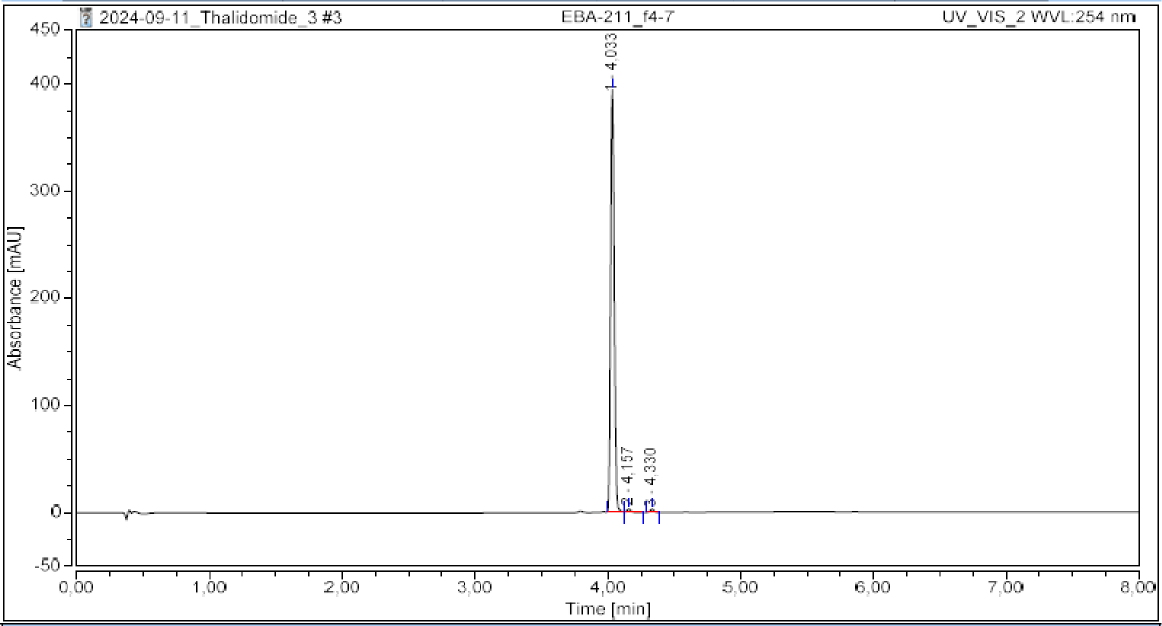

Integration Results

No.	Peak Name	Retention Time min	Area mAU*min	Height mAU	Relative Area %	Relative Height %	Amount n.a.
1		4,033	12,112	394,218	98,47	98,46	n.a.
2		4,157	0,101	3,185	0,82	0,80	n.a.
3		4,330	0,087	2,980	0,71	0,74	n.a.
**Total:**			**12,300**	**400,383**	**100,00**	**100,00**	

Compound 13

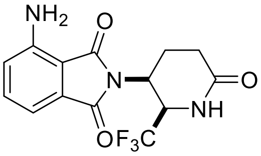

Chromatogram and Results

Injection Details

Injection Name:	**EBA-213_f2-5**	*Run Time (min):*	**8,00**
Vial Number:	**RB7**	*Injection Volume:*	**1,00**
Injection Type:	**Unknown**	*Channel:*	**UV_VIS_2**
Calibration Level:		*Wavelength:*	**254**
Instrument Method:	**General method (0.1TFA_MeCN_10-90)**	*Bandwidth:*	**16**
Processing Method:	**Quantitative**	*Dilution Factor:*	**1,0000**
Injection Date/Time:	**30.okt..24 18:48**	*Sample Weight:*	**1,0000**

Chromatogram

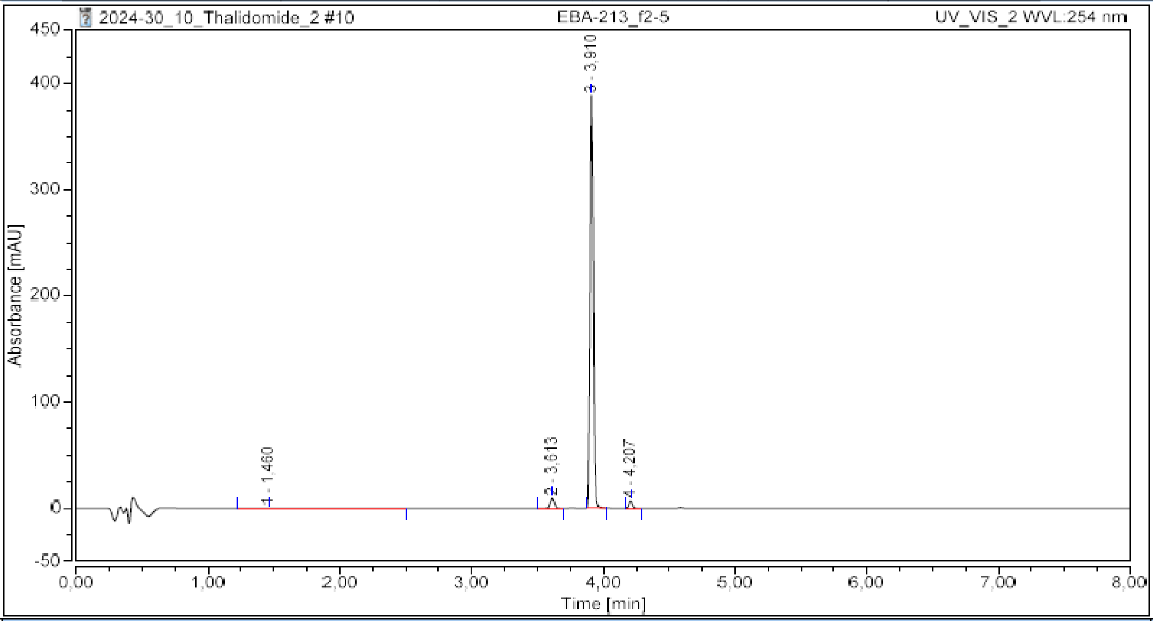

Integration Results

No.	Peak Name	Retention Time min	Area mAU*min	Height mAU	Relative Area %	Relative Height %	Amount n.a.
1		1,460	0,064	0,095	0,53	0,02	n.a.
2		3,613	0,341	9,931	2,83	2,45	n.a.
3		3,910	11,433	388,408	94,84	95,66	n.a.
4		4,207	0,217	7,584	1,80	1,87	n.a.
**Total:**			**12,055**	**406,018**	**100,00**	**100,00**	

Compound 14

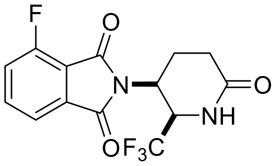

Chromatogram and Results

Injection Details

Injection Name:	**EMH37_izolera**	*Run Time (min):*	**8,00**
Vial Number:	**GD3**	*Injection Volume:*	**1,00**
Injection Type:	**Unknown**	*Channel:*	**UV_VIS_2**
Calibration Level:		*Wavelength:*	**254**
Instrument Method:	**General method (0.1TFA_MeCN_10-90)**	*Bandwidth:*	**16**
Processing Method:	**Quantitative**	*Dilution Factor:*	**1,0000**
Injection Date/Time:	**21.nov..24 11:24**	*Sample Weight:*	**1,0000**

Chromatogram

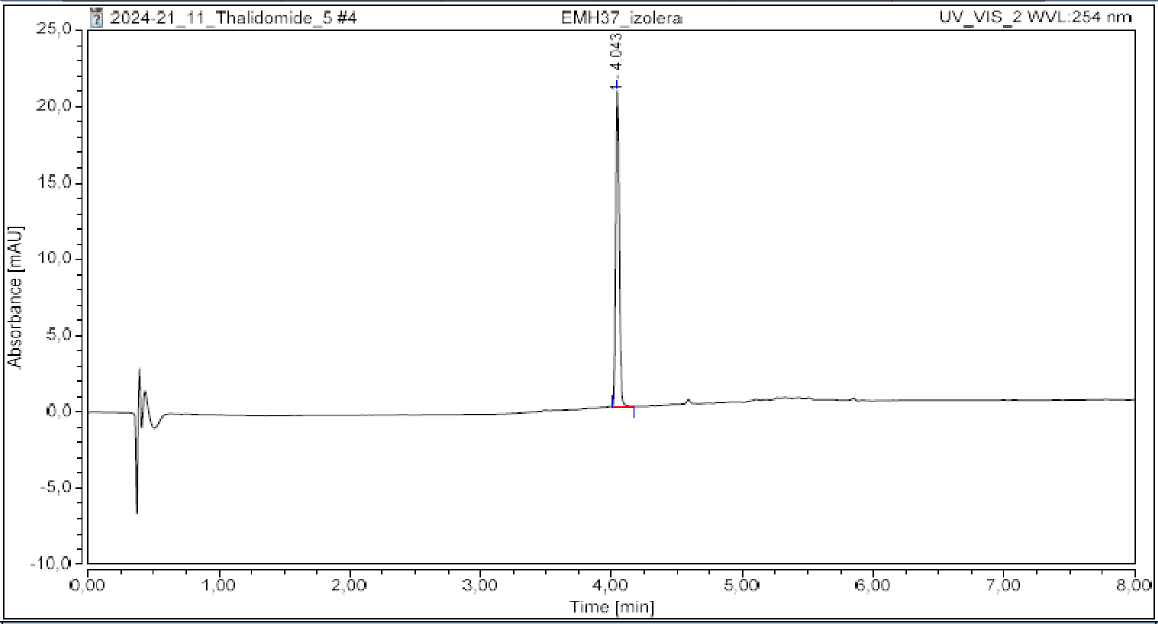

Integration Results

No.	Peak Name	Retention Time min	Area mAU*min	Height mAU	Relative Area %	Relative Height %	Amount n.a.
1		4,043	0,647	20,650	100,00	100,00	n.a.
**Total:**			**0,647**	**20,650**	**100,00**	**100,00**	

Compound 15

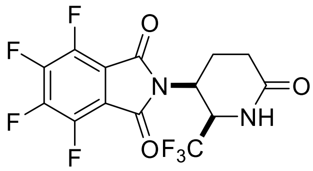

Chromatogram and Results

Injection Details

Injection Name:	**EBA-212**	*Run Time (min):*	**8,00**
Vial Number:	**RB8**	*Injection Volume:*	**1,00**
Injection Type:	**Unknown**	*Channel:*	**UV_VIS_2**
Calibration Level:		*Wavelength:*	**254**
Instrument Method:	**General method (0.1TFA_MeCN_10-90)**	*Bandwidth:*	**16**
Processing Method:	**Quantitative**	*Dilution Factor:*	**1,0000**
Injection Date/Time:	**30.okt..24 19:01**	*Sample Weight:*	**1,0000**

Chromatogram

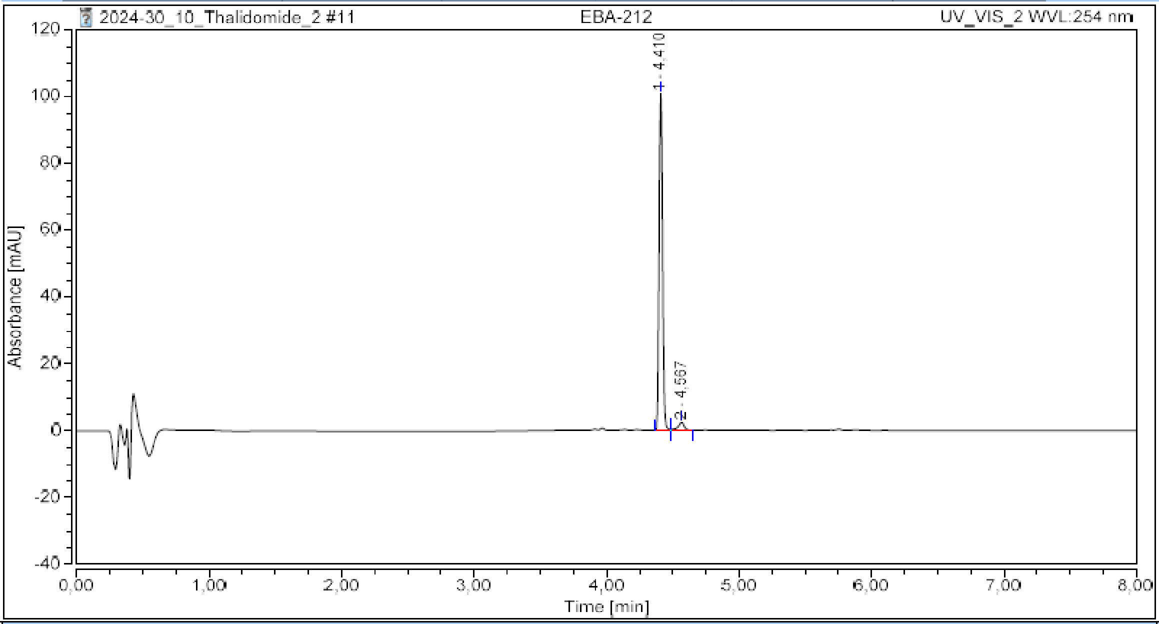

Integration Results

No.	Peak Name	Retention Time min	Area mAU*min	Height mAU	Relative Area %	Relative Height %	Amount n.a.
1		4,410	3,009	100,862	95,91	97,59	n.a.
2		4,567	0,128	2,487	4,09	2,41	n.a.
**Total:**			**3,138**	**103,349**	**100,00**	**100,00**	

Docking (Figure S1)

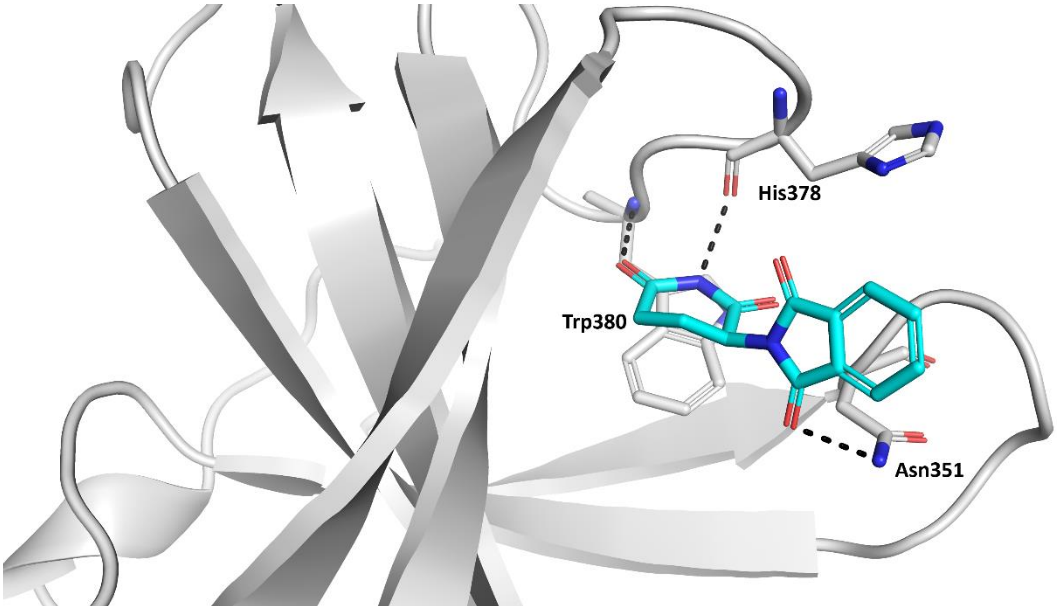

Figure S1: Co-crystal structure of thalidomide bound to cereblon (PDB entry: 7BQU), used as the reference for docking studies.

Angiogenesis assays (Figure S2–S7, Table S1–S2)

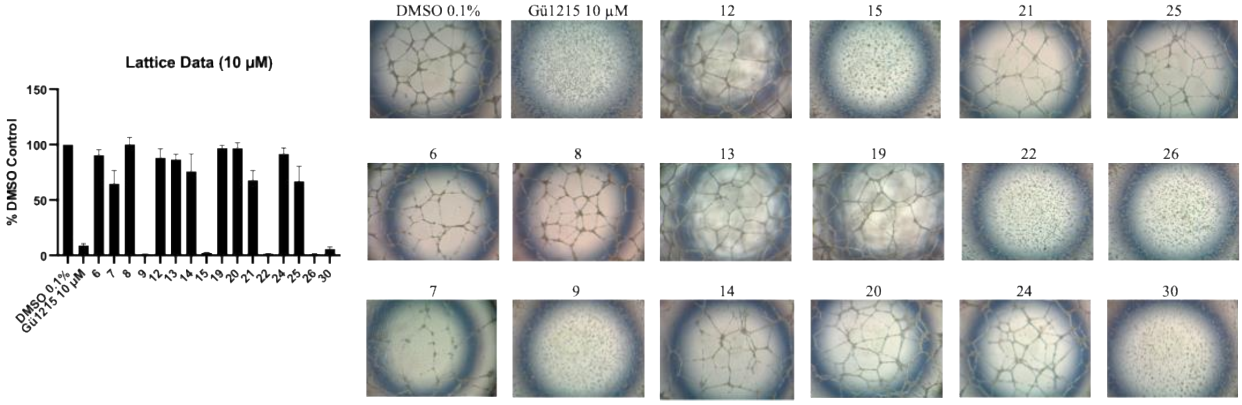

Figure S2. *Summary of tube formation assay results, tested at 10 µM.*

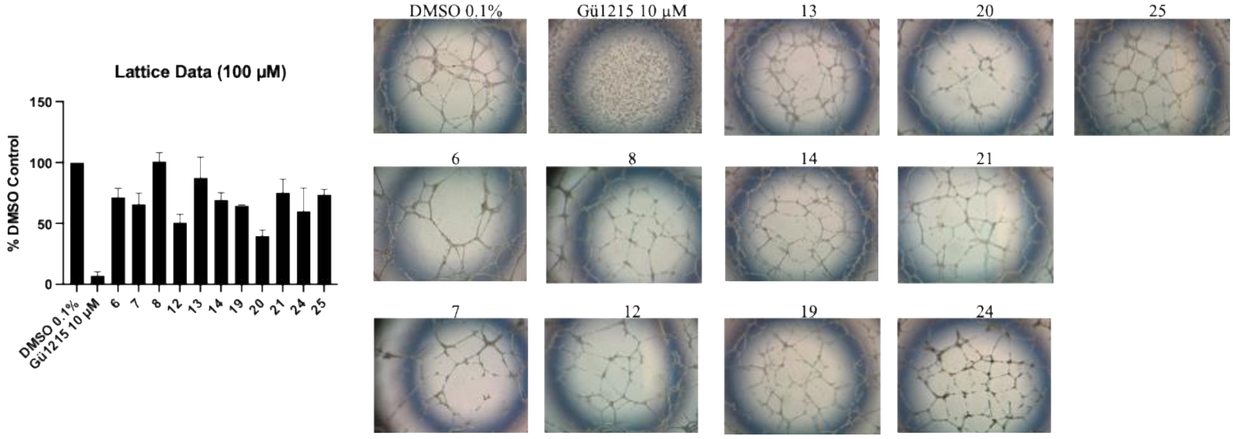

Figure S3. *Summary of tube formation assay results, tested at 100 µM.*

Table S1. *Anti-angiogenic activity in the tube formation assay, comprehensive results.*

Compounds	% Inhibition at 10 µM	% Inhibition at 100 µM
10 µM **Gü1215** (positive control)	91.2%	92.8%
**6**	9.5%	28.6%
**7**	35.3%	34.2%
**8**	n.i.	n.i.
**9**	99.1%	-
**12**	12.0%	49.3%
**13**	13.4%	12.6%
**14**	24.3%	30.8%
**15**	97.7%	-
**19**	3.2%	35.4%
**20**	3.3%	60.2%
**21**	32.1%	24.7%
**22**	98.5%	-
**24**	8.4%	40.1%
**25**	33.0%	26.5%
**26**	98.8%	-
**30**	94.5%	-

n.i. – no inhibition

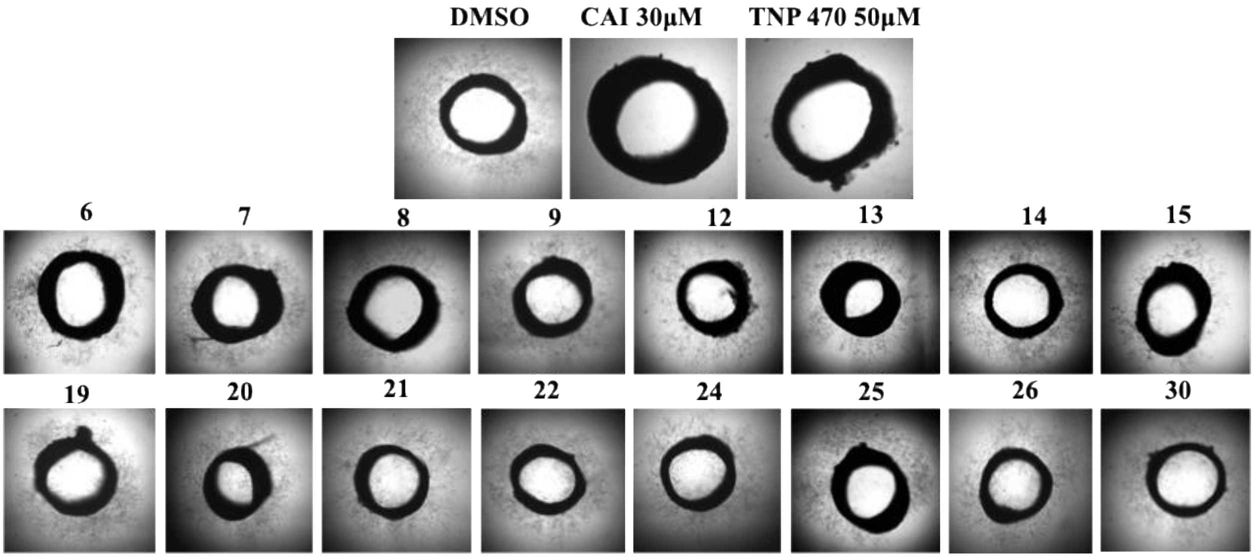

Figure S4. *Rat aortic ring assay at 50 µM. The images are representitive of 3 replicates using aortas from 3–4 different rats.*

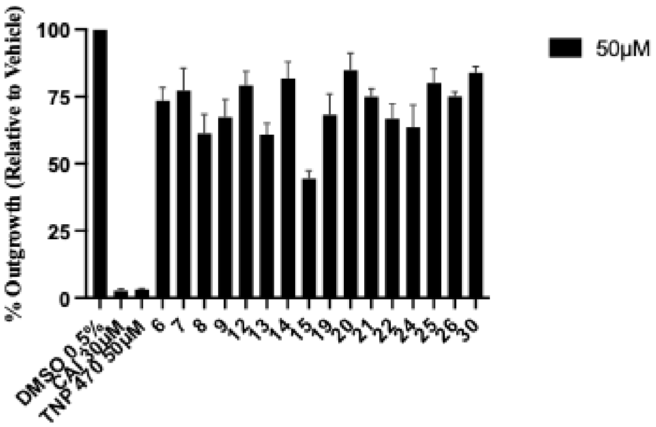

Figure S5. Relative area of angiogenic sprouting in the Rat aortic assay at 50 μM, reported in square pixels, quantified using Adobe Photoshop.

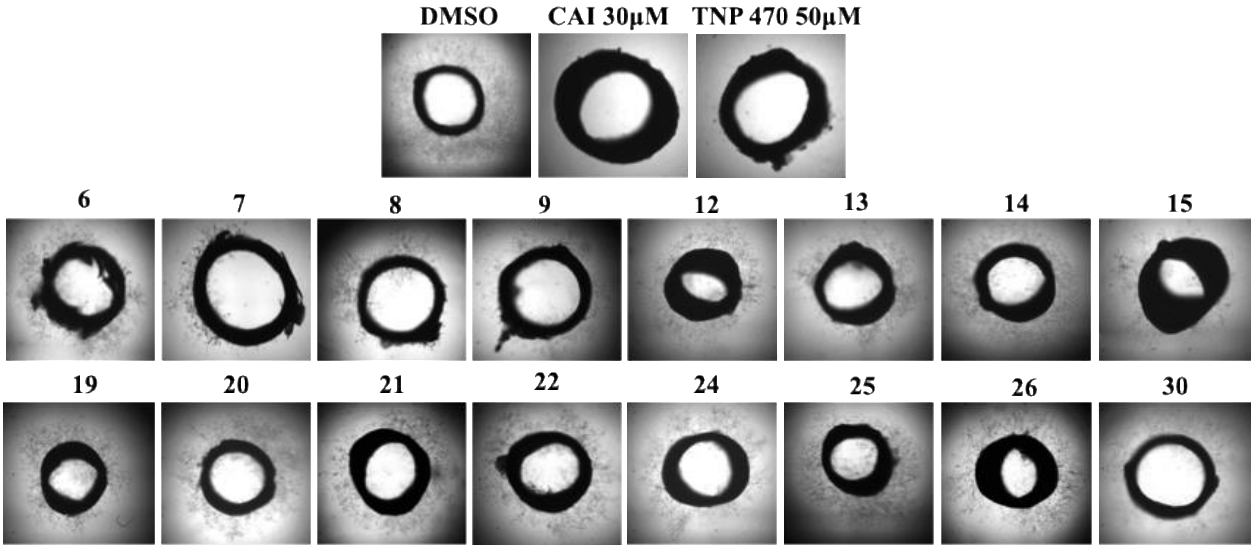

Figure S6. Rat aortic ring assay at 100 μM. The images are representitive of 3 replicates using aortas from 3–4 different rats.

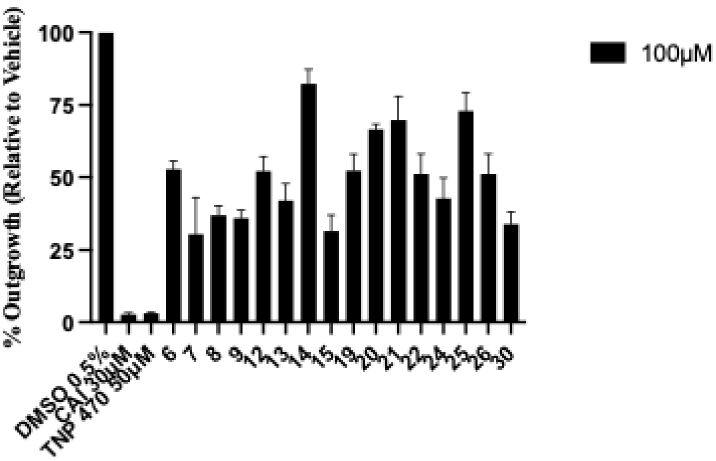

Figure S7. Relative area of angiogenic sprouting in the Rat aortic assay at 100 μM, reported in square pixels, quantified using Adobe Photoshop.

Table S2. *Antiangiogenic activity in the Rat aortic ring assay, comprehensive results.*

**Test Compounds**	**% Inhibition 50µM**	**% Inhibition 100µM**
**CAI** 30µM (Positive Control)	95.13%	
**TNP 470** 50µM (Positive Control)	98.13%	
**6**	26.56	47.24%
**7**	22.67 %	69.45%
**8**	38.76%	62.84%
**9**	32.59%	63.84%
**12**	20.86%	47.9%
**13**	39.24%	57.79%
**14**	18.32%	17.64%
**15**	55.54%	68.36%
**19**	31.76%	47.7%
**20**	15.13%	33.39%
**21**	24.95%	30.15%
**22**	33.18%	48.08%
**24**	36.45%	57.01%
**25**	19.85%	27.81%
**26**	24.91%	35.4%
**30**	16.11%	65.26%
